# Selectively Charged and Zwitterionic Analogues of the Smallest Immunogenic Structure of Streptococcus Pneumoniae Type 14

**DOI:** 10.3390/molecules24183414

**Published:** 2019-09-19

**Authors:** Tiziana Gragnani, Doretta Cuffaro, Silvia Fallarini, Grazia Lombardi, Felicia D’Andrea, Lorenzo Guazzelli

**Affiliations:** 1 Dipartimento di Farmacia, University of Pisa, Via Bonanno 6/33, 56126 Pisa, Italy; mastgragnani@inwind.it (T.G.); doretta.cuffaro@for.unipi.it (D.C.); 2 Dipartimento di Scienze del Farmaco, University of Piemonte Orientale Amedeo Avogadro, Largo Donegani 2, 28100 Novara, Italy; silvia.fallarini@uniupo.it (S.F.); grazia.lombardi@uniupo.it (G.L.)

**Keywords:** *Streptococcus pneumoniae* type 14, zwitterionic analogues, competitive ELISA

## Abstract

Zwitterionic polysaccharides (ZPs) have been shown in recent years to display peculiar immunological properties, thus attracting the interest of the carbohydrate research community. To fully elucidate the mechanisms underlying these properties and exploit the potential of this kind of structures, in depth studies are still required. In this context, the preparation of two cationic, an anionic, as well as two zwitterionic tetrasaccharide analogues of the smallest immunogenic structure of *Streptococcus pneumoniae* type 14 (SP14) capsular polysaccharide are presented. By exploiting a block strategy, the negative charge has been installed on the non-reducing end of the lactose unit of the tetrasaccharide and the positive charge either on the non-reducing end of the lactosamine moiety or on an external linker. These structures have then been tested by competitive ELISA, showing that the structural variations we made do not modify the affinity of the neutral compound to binding to a specific antibody. However, lower efficacies than the natural SP14 compound were observed. The results obtained, although promising, point to the need to further elongate the polysaccharide structure, which is likely too short to cover the entire epitopes.

## 1. Introduction

Vaccination represents one of public health’s most cost-effective interventions, deeply contributing to global health security and striving against antimicrobial resistance. In this context, carbohydrate-based vaccines have been studied and developed for many years [1,2,3,4]. The cells of several bacteria, virus, and fungi are surrounded by a complex, often specific, pattern of non-mammalian glycan structures, which represent their primary virulence factor and can protect them from the hosts’ immune defenses. These pathogen-specific glycan structures act as epitopes, able to elicit specific antibodies when in contact with the host immune cells, representing promising target structures for the development of vaccines.

In general, polysaccharides are characterized by low immunogenic activities, they are able to trigger B-cell-mediated immune responses without IgG class switching and memory development [5]. For this reason, the two polysaccharide vaccines currently on the market are both conjugated to an immunogenic carrier protein. The 10-valent (PCV10) vaccine is composed of capsular polysaccharides purified from 10 serotypes (1, 4, 5, 6B, 7F, 9V, 14, 18C, 19F and 23F). Each capsular polysaccharide is conjugated to a carrier protein, either protein D (an outer membrane protein from non-typeable *Haemophilus influenzae*), tetanus toxoid or diphtheria toxoid. PCV13 contains the capsular polysaccharides of serotypes 1, 3, 4, 5, 6A, 6B, 7F, 9V, 14, 18C, 19A, 19F and 23F, individually conjugated to a nontoxic diphtheria cross-reactive material (CRM197) carrier protein. Both PCV10 and PCV13 have been shown to be safe, effective and to have both direct (in vaccinated individuals) and indirect (in unvaccinated individuals living in communities with vaccinated children) effects against pneumococcal diseases [6]. Literature data have shown that particular polysaccharides, bearing on their structure both negatively and positively charged functionalities, and thus called zwitterionic polysaccharides (ZPs), are endowed with peculiar immunological properties [7]. ZPs, in fact, are able to be processed by the antigen presenting cells (APC) and loaded into the class-II major histocompatibility complex (MCH II), for presentation to T-cells and activation of immune responses [8].

Carbohydrate synthetic chemistry has become, during the years, a valuable tool for the preparation of complex structures both in the search of protective epitopes [9,10,11,12,13,14,15,16,17,18] and in the preparation of epitope analogues and mimics [19,20,21]. Due to the interest raised by ZPs in the carbohydrate-based vaccine research area, several recent synthetic efforts have been devoted to the preparation of both natural zwitterionic structures [21,22,23] and even to the transformation of otherwise neutral carbohydrate capsular fragments into their zwitterionic analogues [21]. From the synthetic perspective, the insertion of charged species in the target structures represents an extra-challenging aspect.

**Figure 1:**
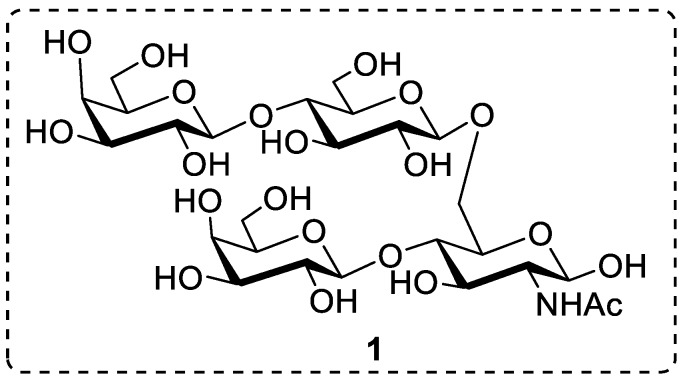
Identified smallest immunogenic fragment of S. *pneumoniae* type 14 capsular polysaccharide (CPS).

*Streptococcus pneumoniae* (S. *pneumoniae*) is a Gram-positive bacterium responsible for invasive and non-invasive infections in adults and children [24]. As mentioned previously, different carbohydrate-based vaccines against *S. pneumoniae* (Prevenar^®^, Synflorix™ (PCV10) [25] Prevenar 13^®^ (PCV 1)) have been licensed and commercialized. *S. pneumoniae* type 14 (SP14) is one of the serotypes with major worldwide clinical relevance. In 2008, Safari et al. [26] established that the synthetic branched tetrasaccharide *β-d-Galp-(1→4)-β-d-Glcp-(1→6)-[β-d-Galp-(1→4)]-β-d-GlcpNAc* (**1**) of the SP14 capsular polysaccharide (CPS) (Figure 1), previously identified by Mawas et al. [27], elicits a protective antibody response when conjugated with the immunogenic protein CRM197.

This fragment of SP14 CPS thus represents the smallest structure for the development of a synthetic vaccine and was further studied after conjugation with the bovine serum albumin (BSA) carrier protein [28] or as part of future potential synthetic glycoconjugate vaccines in the case of gold glyconanoparticles [29,30].

As part of an ongoing project aimed at further elucidating the molecular basis of the ZPS immunological properties, we selected this well-known model tetrasaccharide with the intention to study how its gradual zwitterionization affects the biological activity. Therefore, along with the synthesis of the methyl glycoside of **1** [26,27], needed as a benchmark for biological evaluation, here we report the preparation of an anionic, two cationic, and two zwitterionic tetrasaccharide analogues. The prepared structures were evaluated by competitive ELISA to understand whether the introduction of charges or the zwitterionization influence the ability to bind to specific antibodies.

## 2. Results and Discussion

**Scheme 1:**
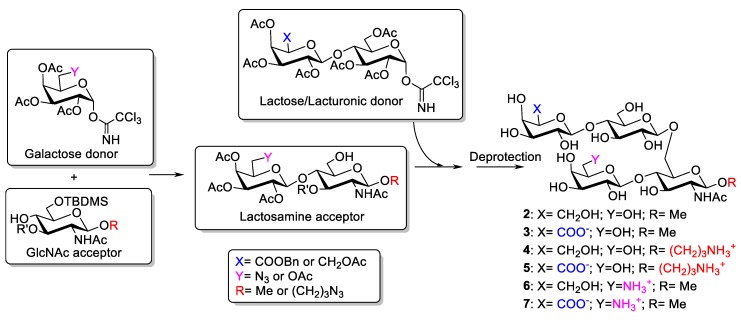
General approach for the synthesis of neutral tetrasaccharide **2** and of negatively/positively charged and zwitterionic target structures **3**–**7**.

Several different synthetic approaches have been explored thus far for preparing tetrasaccharide **1**, which is formally constituted by a lactose unit linked to an *N*-acetyl-lactosamine unit through a beta 1→6 glycosidic linkage. Thanks to a long-lasting experience in modifying the structure of lactose [31,32,33,34], we decided to use this natural disaccharide as the starting material for the preparation of lactose/lacturonic building block donors, while we built up lactosamine acceptors from suitably protected monosaccharide derivatives. The general design of the planned synthetic strategy is reported in Scheme 1, with the negative charge located on the galactose frame of the lactose unit (X) and the positive charge inserted either on the galactose part of the lactosamine unit (Y) or on the external linker (R).

### 2.1. Chemistry

#### 2.1.1. Preparation of Required *N*-acetyl-glucosamine Acceptors **20**–**23** and Lacturonic Donor **28**

**Scheme 2:**
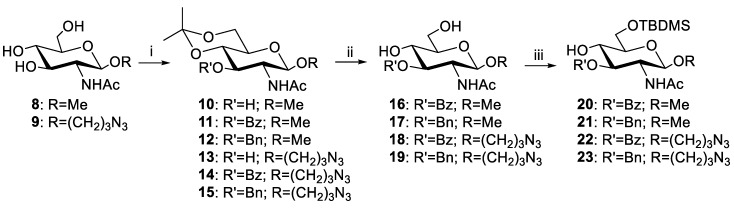
Synthesis of the target acceptors **20**–**23**. Reagents and conditions: (i) a) 2-MP, CSA, DMF, rt, 2–4 h (**10**: 91%; **13**: 88%); b) BzCl, Py, rt, 2–5 h (**11**: 88%) or BnBr, KOH, THF-H_2_O, 18-crown-6, rt, 4 h (**12**: 78%); (ii) 70% aq AcOH, 40 °C, 3–5 h (**16**: 96%; **17**: 96%; **18**: 89% from **13**; **19**: 91% from **13**); (iii) TBDMSCl, Py, rt, 2–4 h (**20**: 81%; **21**: 93%; **22**: 91%; **23**: 96%).

β-*d-*GlcNAc acceptors **20**–**23** (Scheme 2), characterized by the presence of an orthogonal protecting group on the 6-OH in view of their further use in the preparation of lactosamine acceptors, were synthesized starting from known **8** [35] and **9** [36]. The fully protected derivatives **10**–**15** (78–97%) were obtained by the introduction of a 4,6-*O*-isopropylidene acetal (2-methoxypropene/CSA) and either benzylation or benzoylation of OH-3.

The acid hydrolysis of the acetal group of **10**–**15** lead to the corresponding diols **16**–**19** (89–96%) which were submitted to a regioselective 6-OH silylation affording target acceptors **20**–**23** (91–96%, see Supporting Information File for full experimental details).

**Scheme 3:**
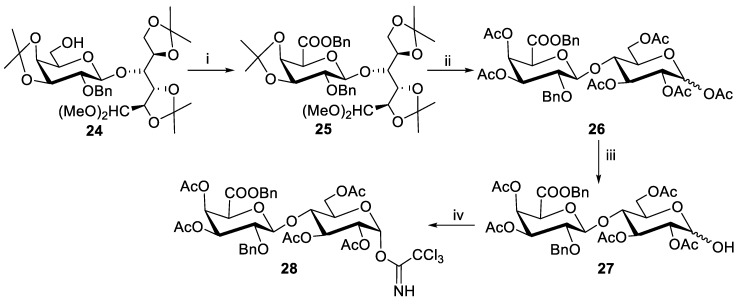
Synthesis of the lacturonic donor **28**. Reagents and conditions: (i) TEMPO, 5% aq NaHCO_3_, KBr, 13% aq NaOCl, Me_2_CO, rt, 15 min and then BnBr, KF, DMF, rt, overnight (93%); (ii) 80% aq ACOH, 80 °C, 4 h, and then 1:2 Ac_2_O-Py, rt, 12 h (98%); (iii): NH_2_NH_2_·AcOH, dry DMF, 60 °C, 30 min (79%); (iv) CCl_3_CN, DBU, dry CH_2_Cl_2_, rt, 30 min (91%).

Lacturonic donor **28** (Scheme 3) was synthesized starting from known lactose derivative **24** [37], prepared by benzylation at the C-2′ of known 2,3:5,6:3′,4′-tri-*O*-isopropylidene-6′-*O*-(1-methoxy-1-methylethyl)lactose dimethyl acetal [38] followed by mild acidic hydrolysis of the 6’-O-methoxyisopropyl acetal. Oxidation at the C-6′ position of **24** (TEMPO, NaOCl-NaHCO_3_-Me_2_CO) followed by protection of the carboxylic acid as benzyl ester (BnBr, KF, DMF) afforded **25** in an excellent yield (93%). This was converted into **26** (α/β ratio = 2:3, NMR) through acidic hydrolysis of acetal protections (80% aq AcOH, 80 °C) and consecutive acetylation with acetic anhydride in pyridine. Selective removal of the acetyl group at the anomeric position of **26** under mild basic condition (NH_2_NH_2_·AcOH, DMF) followed by treatment of **27** with trichloroacetonitrile in the presence of DBU gave pure α-d-trichloroacetimidate **28** (70% from **25**) (see Supporting Information File for full experimental details).

#### 2.1.2. Synthesis of the Target Tetrasaccharides **2**–**7**

**Scheme 4:**
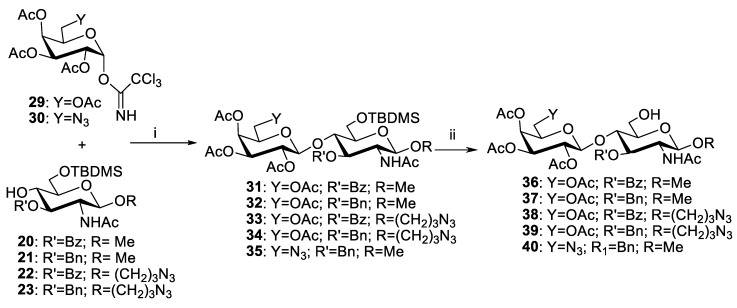
Synthesis of the β-(1→4)-lactosamine acceptors **36**–**40**. Reagents and conditions: (i) TMSOTf, CH_2_Cl_2_, 300-AW MS, −30 °C to rt, 17–24 h (**31**: 62%, **32**: 40%, **33**: 47%, **34**: 72%, **35**: 57%); (ii) 70% aq AcOH, 70 °C, 1–2 h (**36**: 83%, **37**: 83%, **38**: 96%, **39**: 81%, **40**: 92%).

Tetrasaccharide target structures were then built up from the prepared blocks. First, lactosamine acceptors **36**–**40** were synthetized (Scheme 4). Glycosylation of the OH-4 of *N*-acetyl-glucosamines is known to represent a challenging reaction [39,40]. Instead of changing the protecting group pattern on the acceptors, and in particular the protecting group on the amino function which would ultimately require the deprotection/acetylation sequence on larger structures, we decided to screen several reaction conditions on this kind of structure (supporting information file). 

The optimal conditions found were the following: trimethylsilyltriflate (TMSOTf, 0.5 eq) [41] as the catalyst of the glycosylation reaction which was added at −30 °C to a strictly anhydrous solution of acceptors **20**–**23** (1.0 eq) and known donors **29** [42] and **30** [43] (1.5 eq) in dry DCM. After 24–48 h the desired β-(1→4)-lactosamine derivatives **31**–**35** were obtained as the only products with satisfying yields (40%–72%) for all substrates (Scheme 4). NMR data confirmed the disaccharide structures and the high values (about 7.5 Hz) of the *J*_1′,2′_ coupling constants, in agreement with an axial-axial disposition of H-1′ and H-2′, ascertained the desired beta configuration of the formed glycosidic bonds. Disaccharide acceptors **36**–**40** were then prepared in good to excellent yields (83%–96%) through an easy acid cleavage of the silyl protecting group by treating **31**–**35** with a 70% aq AcOH solution at 70 °C.

**Scheme 5:**
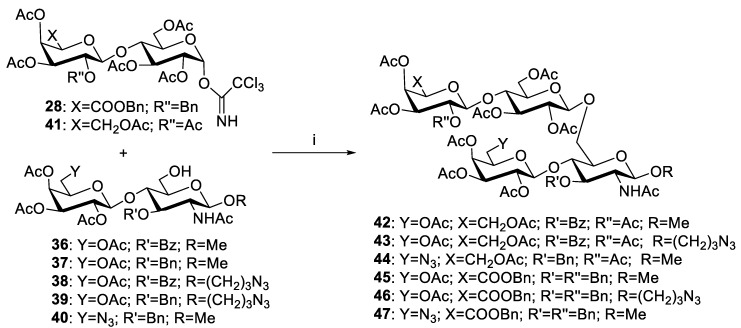
Synthesis of tetrasaccharides **42**–**47**. Reagents and conditions: (i): BF_3_·Et_2_O, AW-300 MS, CH_2_Cl_2_, −15 °C to rt, 17–24 h (**42**: 70%; **43**: 81%; **44**: 89%; **45**: 88%; **46**: 75%; **47**: 77%).

Lactose donors **28** and **41** [43,44] were employed for the glycosylation reaction with the prepared lactosamine acceptors **36**–**40** (Scheme 5). As expected, this glycosylation step was less problematic when compared to the previous β-(1→4)-galactosylation reaction due to the higher accessibility and reactivity of the primary 6-OH than the 4-OH in **20**–**23**.

Thus, lactosamine acceptors **36**–**40** (1.0 eq) were coupled with trichloroacetimidate donors **28** and **41** (1.5 eq) in CH_2_Cl_2_ using boron trifluoride etherate (BF_3_·Et_2_O, 1.3 eq) as the catalyst in the presence of acid-washed molecular sieves. Tetrasaccharides **42**–**47** (Scheme 5) were isolated in good yields (70%–89%) after purification by flash chromatography on silica gel of crude products. The presence of the acetate participating group on donors **28** and **41** allowed again for obtaining only the beta anomer. It is worth noticing that no differences in reactivity were observed between the peracetylated lactose trichloroacetimidate donor **41** and its C-6’ oxidized analogue **28**.

**Scheme 6:**
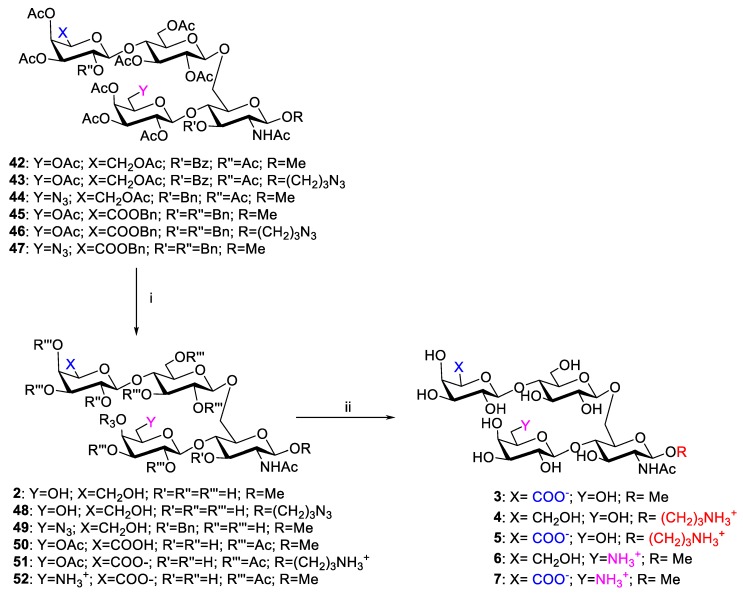
Synthesis of target tetrasaccharides **2–7**. Reagents and conditions: (i) for **2** and **48**: 0.33M MeONa-MeOH, rt, 12-48 h (**2**: 72%; **48**: 69%); for **49**: 3.5N NH_3_-MeOH, rt (65%); for **50**: H_2_, 10% Pd/C, 2.5:1 MeOH-EtOAc, 48 h (98%); for **51** and **52**: H_2_, 10% Pd/C, 3:1:0.5 MeOH-CH_2_Cl_2_-H_2_O, 48 h (**51**: 70%; **52**: 76%); (ii) for **3**: 3.5N NH_3_-MeOH, 48 h (89%); for **4** and **6**: H_2_, 10% Pd/C, 3:1 MeOH-H_2_O, 1% HCl-MeOH, 20 h (**4**: 71%; **6**: 96%); for **5** and **7**: 3.5N NH_3_-MeOH, rt, 48 h (**5**: 98%; **7**: 86%).

The target point-charged tetrasaccharide analogues (anionic **3**, and cationic **4** and **6**) of the neutral structure **2** [44], as well as zwitterionic analogues (**5** and **7**), were then prepared by the following removal of the protecting groups (Scheme 6). Deprotection of compound **42** required a simple Zemplen reaction (0.33M MeONa/MeOH) to afford the neutral tetrasaccharide **2** (72%) which, as mentioned before, was needed as a benchmark for the biological evaluation of the charged structures. The desired ammonium derivatives **4** (71%) and **6** (96%) were obtained by deprotection of compounds **43** and **44** respectively (Scheme 6), through a two-step procedure. After the basic hydrolysis of the ester groups either by using Zemplen conditions (0.33M MeONa/MeOH) or by treating with a methanolic ammonia solution (3.5N NH_3_-MeOH) [45], the partially deprotected disaccharides **48** and **49** were submitted to catalytic hydrogenolysis (H_2_, 10% Pd/C) in MeOH in the presence of 1% HCl-MeOH.

Unfortunately, unsatisfactory results were obtained when the same deprotection protocol was applied to tetrasaccharides **45** and **46**. In fact, during the basic hydrolysis of the acetyl groups a side trans-esterification reaction involving the 6’’’ position occurred, and the corresponding methyl esters of benzylic esters **45** and **46** were isolated. Therefore, a slightly different deprotection pathway was followed (Scheme 6) by reversing the order of the two deprotection steps. The catalytic hydrogenolysis (H_2_, 10% Pd/C) of **45** and **46** was performed in 2.5:1 MeOH-EtOAc or in a ternary solvent system (3:1:0.5 MeOH-CH_2_Cl_2_-H_2_O) and in all cases, the purification by chromatographic mean, afforded pure uronic acids **50** and **51** in good yields (70% and 98%, respectively). The following deacetylation reaction with 3.5N NH_3_-MeOH gave the desired deprotected target tetrasaccharides **3** and **5** (89% and 98%, respectively). As no drawbacks were encountered, this second protocol was also applied to **47** (Scheme 6), thus obtaining zwitterionic tetrasaccharide **7** (86%).

All compounds were characterized and their mono- and two-dimensional NMR analyses (^1^H, ^13^C, DEPT-135, COSY, HETCOR, HSQC) were consistent with their structures (please refer to the experimental section).

### 2.2. Biological Tests

**Figure 2:**
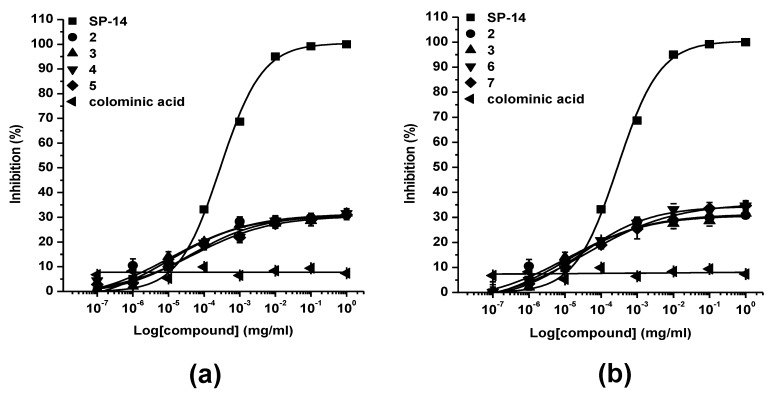
**a, b**. Concentration-response curves of SP14 CPS fragments on the inhibition of SP-14 CPS binding to an anti-SP-14 rabbit polyclonal antibody (competitive ELISA).

First of the all, we determined the biocompatibility of the newly synthesized compounds by calcein-AM viability assay on the RAW 264.7 cell line [46]. No compounds resulted toxic at all concentrations tested (1 × 10^−5^ –1 × 10^−1^ mg/mL), suggesting that the structural changes we have introduced into the CPS fragments did not modify cell viability (data not shown). Competitive ELISA were then performed to investigate the importance of the chain length, as well as of the structural charges, on the antigenic properties of the newly synthesized compounds [20]. Experiments performed with a specific rabbit anti-SP14 polyclonal antibody showed that the natural SP14 CPS (positive control) and all synthesized oligosaccharides are recognized by the antibody in a concentration-dependent manner. Colominic acid was always included as a negative control (Table 1 and Figure 2).

Data show that the relative affinities, expressed as IC_50_ values (mg/mL) resulted similar among the different charged fragments **3**–**7**. The introduction of either a positive **4** and **6** or a negative **3** charge into these molecules did not modify the potency of neutral tetrasaccharide **2** (IC_50_ calculated values at 10^−5^ order of magnitude for all compounds), suggesting that the presence of charged functionalities within the repeating unit do not improve the ability of compounds to bind to a specific antibody. The introduction of both positive and negative charges (ZPS compounds, **5** and **7**) gave the same results, independently of the charge positions within the tetrasaccharide structure. All analogues (**2**–**7**) exhibited similar efficacies (31 ± 3), which are lower (−70%) than the natural compound (100 ± 3). These results confirm that to obtain a high inhibition of the antibody binding to natural SP14 CPS, a higher number of different CPS epitopes is required [47].

## 3. Materials and Methods 

### 3.1. Chemistry

#### 3.1.1. General

Optical rotations were measured on a Perkin-Elmer 241 polarimeter at 20 ± 2 C. Melting points were determined with a Kofler hot-stage apparatus and are uncorrected. ^1^H NMR spectra were recorded in appropriate solvents with a Bruker Avance II operating at 250.13 MHz or a Bruker DRX 600 (biodrx600) spectrometer operating at 600 MHz. ^13^C NMR spectra were recorded with the above spectrometers operating at 62.9 or 150 MHz. The assignments were made, when possible, with the aid of DEPT, HETCOR, HSQC and COSY experiments. The first order proton chemical shifts δ are referenced to either residual CD_3_CN (δ_H_ 1.94, δ_C_ 1.28) or residual CD_3_OD (δ_H_ 3.31, δ_C_ 49.0) and *J*-values are given in Hz. All reactions were followed by TLC on Kieselgel 60 F_254_ or Silica gel 60 RP-18 F_254s_ or with detection by UV light and/or with ethanolic 10% phosphomolybdic or sulfuric acid, and heating. Kieselgel 60 (E. Merck, 70–230 and 230–400 mesh, respectively) or Biotage reverse phase C18 silica columns was used for column and flash chromatography. Some of the flash chromatography were conducted by the automated system Isolera Four (Biotage^®^, Uppsala, Sweden), equipped with a UV detector with variable wavelength (200–400 nm). Unless otherwise noted, solvents and reagents were obtained from commercial suppliers and were used without further purification. All reactions involving air- or moisture-sensitive reagents were performed under an argon atmosphere by using anhydrous solvents. Anhydrous dimethylformamide (DMF), dichloromethane (CH_2_Cl_2_) and methanol (CH_3_OH) were purchased from Sigma–Aldrich. Other dried solvents were obtained by distillation according to standard procedure [48] and stored over 4Å molecular sieves activated for at least 12 h at 200 °C. MgSO_4_ was used as the drying agent for solutions. Elemental analysis were obtained using an Elementar Vario MICRO cube equipment.

Compound **8** [35], **9** [36] and donors **29** [42], **30** [43], **28** [43,44] were prepared according to the reported procedure. The synthesis of compounds **10**–**15**, **18**–**23**, **25**–**28** and **31**–**40** is reported in the Electronic Supplementary Information.

#### 3.1.2. General Procedure for the *6-O-glycosylation*: Synthesis of the Tetrasaccharides **42**–**47**

A mixture of the appropriate acceptors **36**–**40** (1.0 eq), excess of opportune donors **28** or **41** (1.5 eq) and activated AW 300 MS (800 mg) in dry CH_2_Cl_2_ (20 mL), was stirred for 15 min at room temperature, cooled to −15 °C and BF_3_·Et_2_O (1.3 eq) was added. The reaction mixture was allowed to slowly attain room temperature with stirring until the appropriate acceptor was disappeared (17–24 h, TLC, EtOAc or 1:9 toluene-EtOAc) and the formation of a major UV visible spot had occurred. Et_3_N (1.0 mL) was added and after 30 min the mixture was filtered through a short pad of Celite, diluted with CH_2_Cl_2_, and concentrated under diminished pressure. Purification of crude product by flash chromatography on silica gel afforded pure tetrasaccharides **42**–**47**.

*Methyl 2-acetamido-3-O-benzoyl-4-O-(2,3,4,6-tetra-O-acetyl-β-d-galactopyranosyl)-6-O-[4-O-(2,3,4,6-tetra-O-acetyl-β-d-galactopyranosyl)-2,3,6-tri-O-acetyl-β-d-glucopyranosyl]-2-deoxy-β-d-glucopyranoside* (**42**). The glycosylation of lactosamine acceptor **36** (155 mg, 0.23 mmol, 1 eq) with lactose donor **41** (269 mg, 0.345 mmol, 1.5 eq) was performed in dry CH_2_Cl_2_ (5.5 mL) with AW-300 MS (300 mg) and BF_3_^.^Et_2_O (36 μL, 0.30 mmol, 1.3 eq) in dry CH_2_Cl_2_ (0.5 mL) in accordance with the general procedure. Purification of crude product by flash chromatography on silica gel (5:95 hexane-EtOAc + 0.1% Et_3_N) afforded pure tetrasaccharide **42** (208 mg, 70%) as a white foam, *R*_f_ 0.19 (5:95 hexane-EtOAc); (α)_D_ -16.1 (c 0.93 in CHCl_3_); ^1^H NMR (250.13 MHz, CD_3_CN): δ 8.00–7.96 (m, 2H, Ar-*H*), 7.58–7.55 (m, 1H, Ar-*H*), 7.49–7.42 (m, 2H, Ar-*H*), 6.54 (d,1H, J_2,NH_ 9.5 Hz, N*H*), 5.30 (dd, 1H, *J*_3′′′4′′′_3.4 Hz, *J*_4′″,5′″_ 1.0 Hz, H-4′′′), 5.25 (dd, 1H, *J*_2,3_ 10.2 Hz, *J*_3,4_ 8.5 Hz, H-3), 5.17 (dd, 1H, *J*_2″,3″_ 9.7 Hz, *J*_3″,4″_ 8.9 Hz, H-3″), 5.11 (dd, 1H, *J*_4__′__,5__′_ 1.1 Hz, *J*_3__′__,4__′_ 3.4 Hz, H-4′), 5.03 (dd, 1H, *J*_2′″,3′″_ 10.4 Hz, H-3′″), 4.96 (dd, 1H, *J*_2__′__,3__′_ 10.4 Hz, H-3′), 4.94 (dd, 1H, *J*_1_′″ ′,_2′″_ 7.8 Hz, H-2′″), 4.84 (dd, 1H, *J*_1__′__,2__′_ 7.6 Hz, H-2′), 4.83 (dd, 1H, *J*_1″,2″_ 7.9 Hz, H-2″), 4.70 (d, 1H, H-1′′), 4.58 (d, 1H, H-1′′′),4.53 (d, 1H, H-1′),4.46 (d, 1H, *J*_1,2_ 8.5 Hz, H-1), 4.39 (dd, 1H, *J*_6″a,6″b_ 12.2 Hz, *J*_5″,6″b_ 2.2 Hz, H-6″b), 4.18-3.98 (m, 5H, H-4, H-5′′′, H-6′′a, H-6′′′a, H-6′′′b), 3.97-3.81 (m, 2 H, H-2, H-4′′), 3.80-3.63 (m, 5H, H-5, H-5′, H-5′′, H-**6**′**a**, H-6′b), 3.42 (s, 3H, OMe), 3.39 (dd, 1H, *J*_6a,6b_ 11.1 Hz, *J*_5,6b_ 7.4 Hz, H-6b), 3.22 (dd, 1H, *J*_5,6a_ 6.0 Hz, H-6a), 2.08, 2.07, 2.03, 2.02, 2.01, 2.00, 1.99, 1.94, 1.89, 1.87, 1.86 (11s, each 3H, 11 × MeCOO), 1.68 (s, 3H, *Me*CON); ^13^C NMR (62.9 MHz, CD_3_CN): δ 171.5-170.4 (12 × MeCO), 166.5 (PhCO), 134.1, 130.3, 129.4 (Ar-*C*H), 131.1 (Ar-*C*), 102.3 (C-1), 101.4 (C-1′, C-1″, C-1′′′), 77.6 (C-5′′), 77.0 (C-4), 74.5 (C-3), 75.0 (C-5), 73.4 (C-5′′′), 73.1 (C-3′′), 72.3 (C-2′′), 71.6 (C-3′′′), 71.5 (C-3′), 71.4 (C-4′′), 71.1 (C-5′), 69.9 (C-2′, C-2′′′), 68.8 (C-6), 68.0 (C-4′′′), 67.7 (C-4′), 62.9 (C-6′′), 61.9, 61.1 (C-6′, C-6′′′), 57.2 (*Me*O), 54.5 (C-2), 23.0 (*Me*CON), 21.1-20.6 (11 × *Me*COO). Elemental Analysis Found: C, 52.25; H, 5.75; N, 1.13. Calculated for C_56_H_73_NO_33_ (1288.17): C, 52.21; H, 5.71; N, 1.09.

*3-Azidopropyl 2-acetamido-3-O-benzoyl-4-O-(2,3,4,6-tetra-O-acetyl-β-d-galactopyranosyl)-6-O-[4-O-(2,3,4,6-tetra-O-acetyl-β-d-galactopyranosyl)-2,3,6-tri-O-acetyl-β-d-glucopyranosyl]-2-deoxy-β-d-glucopyranoside* (**43**). The glycosylation of lactosamine acceptor **38** (338 mg, 0.457 mmol, 1 eq) with lactose donor **41** (535 mg, 0.685 mmol, 1.5 eq) was performed in dry CH_2_Cl_2_ (10 mL) with AW-300 MS (550 mg) and BF_3_^.^Et_2_O (69 μL, 0.594 mmol, 1.3 eq) in dry CH_2_Cl_2_ (1.0 mL), as described in the general procedure. Purification of crude product by flash chromatography on silica gel (15:85 hexane-EtOAc + 0.1% Et_3_N) afforded pure tetrasaccharide **43** (505 mg, 81%) as a white foam, *R*_f_ 0.34 (1:9 toluene-EtOAc); (α)_D_ -16.5 (*c* 0.98 in CHCl_3_); ^1^H NMR (250.13 MHz, CD_3_CN): δ 8.02–7.96 (m, 2H, Ar-*H*), 7.62–7.55 (m, 1H, Ar-*H*), 7.50–7.43 (m, 2H, Ar-*H*), 6.46 (d,1H, *J*_2,NH_ 9.6 Hz, N*H*), 5.30 (dd, 1H, *J*_4′″,5′″_ 1.0 Hz, *J*_3′″,4′″_ 3.5 Hz, H-4′″), 5.26 (dd, 1H, *J*_2″,3″_ 10.4 Hz, *J*_3″,4″_ 8.8 Hz, H-3″), 5.18 (dd, 1H, *J*_2,3_ 9.6 Hz, *J*_3,4_ 9.1 Hz, H-3), 5.11 (dd, 1H, *J*_3__′__,4__′_ 3.4 Hz, *J*_4__′__,5__′_ 1.1 Hz, H-4′), 5.02 (dd, 1H, *J*_2′″,3′″_ 9.3 Hz, H-3′″), 5.00 (dd, 1H, *J*_2__′__,3__′_ 9.7 Hz, H-3′), 4.89 (dd, 1H, *J*_1″,2″_ 7.7 Hz, H-2″), 4.84 (dd, 1H, *J*_1′″,2′″_ 7.8 Hz, H-2′″), 4.83 (dd, 1H, *J*_1__′__,2__′_ 7.9 Hz, H-2′), 4.70 (d, 1H, H-1′), 4.56 (d, 1H, H-1′″), 4.55 (d, 1H, *J*_1,2_ 8.4, H-1), 4.53 (d, 1H, H-1″), 4.39 (dd, 1H, *J*_6″a,6″b_ 12.4 Hz, *J*_5″,6″b_ 2.1 Hz, H-6b″), 4.18-3.95 (m, 5H, H-6′a, H-6^′^b, H-6″a, H-6′″a, H-6′″b), 3.94- 3.81 (m, 4 H, H-2, H-4, H-4″, H-5′″), 3.80-3.50 (m, 5H, H-5, H-5′, H-5″, CH_2_O), 3.39 (dd, 1H, *J*_6a,6b_ 11.1 Hz, *J*_5,6b_ 7.4 Hz, H-6b), 3.36 (t, 2H, *J*_vic_ 6.8 Hz, C*H*_2_N_3_), 3.24 (m, 1H, *J*_5,6a_ 5.9 Hz, H-6a), 2.08, 2.02, 2.01, 2.00, 1.99, 1.97, 1.94, 1.93, 1.89, 1.87, 1.86 (11s, each 3H, 11 × *Me*COO), 1.81 (m, 2H, C*H*_2_), 1.70 (s, 3H, *Me*CON); ^13^C NMR (62.9 MHz, CD_3_CN): δ 171.5-170.4 (12 × *MeC*O), 166.5 (Ph*C*O), 134.1, 130.4, 129.4 (Ar-*C*H), 131.1 (Ar-*C*), 101.5 (C-1, C-1″), 101.4 (C-1′, C-1′″), 74.7 (C-3), 77.5 (C-5), 77.0 (C-4), 75.1 (C-5′), 75.0 (C-5′″), 73.5 (C-4″), 73.2 (C-3″), 71.6 (C-3′″), 71.4 (C-3′, C-2″), 69.9 (C-2′, C-2′″), 68.8 (C-6), 68.0 (C-4′″), 67.7 (C-4′), 54.7 (C-2), 67.1 (*C*H_2_O), 62.9, 61.9, 61.1 (C-6′, C-6″, C-6′″), 48.8 (*C*H_2_N_3_), 29.4 (*C*H_2_), 23.0 (*MeC*ON), 21.1-20.7 (11 × *Me*COO). Elemental Analysis Found: C, 51.37; H, 5.67; N, 4.16. Calculated for C_58_H_76_N_4_O_33_ (1357.24): C, 51.33; H, 5.64; N, 4.13.

*Methyl 2-acetamido-3-O-benzyl-4-O-(2,3,4-tri-O-acetyl-6-azido-6-deoxy-β-d-galactopyranosyl)-6-O-[4-O-(2,3,4,6-tetra-O-acetyl-β-d-galactopyranosyl)-2,3,6-tri-O-acetyl-β-d-glucopyranosyl]-2-deoxy-β-d-glucopyranoside* (**44**). The glycosylation of lactosamine acceptor **40** (97 mg, 0.152 mmol, 1 eq) with lactose donor **41** (178 mg, 0.228 mmol, 1.5 eq) was performed in dry CH_2_Cl_2_ (5.0 mL) with AW-300 MS (270 mg) and BF_3_^·^Et_2_O (27 μL, 0.196 mmol, 1.3 eq) in dry CH_2_Cl_2_ (0.5 mL), as described in the general procedure. Purification of crude product by flash chromatography on silica gel (1:9 hexane-EtOAc + 0.1% Et_3_N) afforded pure tetrasaccharide **44** (170 mg, 89%) as a white foam, *R*_f_ 0.32 (1:9 hexane-EtOAc); (α)_D_ -27.3 (c 1.2 in CHCl_3_); ^1^H NMR (250.13 MHz, CD_3_CN): δ 7.35–7.29 (m, 5H, Ar-*H*), 6.52 (d,1H, *J*_2,NH_ 9.3 Hz, N*H*), 5.28 (m, 2H, H-4′, H-4′″), 5.16 (dd, 1H, *J*_2″,3″_ 8.8 Hz, *J*_3″,4″_ 9.7 Hz, H-3″), 5.01 (m, 3H, H-3′, H-2′″, H-3′″), 4.93 (dd, 1H, *J*_1__′__,2__′_ 7.8 Hz, *J*_2__′__,3__′_ 10.4 Hz, H-2′), 4.86, 4.54 (AB system, 2H, *J*_A,B_ 11.1 Hz, C*H*_2_Ph), 4.81 (dd, 1H, *J*_1″,2″_ 7.9 Hz, H-2″), 4.68 (d, 1H, *J*_1′″,2′″_ 7.9 Hz, H-1′″),4.65 (d, 1H, H-1′), 4.56 (d, 1H, H-1″), 4.26 (d, 1H, *J*_1,2_ 7.8 Hz, H-1), 4.39 (dd, 1H, *J*_6″a,6″b_ 12.1 Hz, *J*_5″,6″b_ 1.9 Hz, H-6″b), 4.15-3.97 (m, 5H, H-6b, H-6″a, H-5′″, H-6′″a, H-6′″b), 3.86 (m, 2H, H-5′, H-4″), 3.77–3.65 (m, 4H, H-2, H-4, H-6a, H-5″), 3.51 (m, 2H, H-3, H-5), 3.37 (s, 3H, OMe), 3.26 (dd, 1H, *J*_6__′__a,6__′__b_ 12.9 Hz, *J*_5__′__,6__′__b_ 7.4 Hz, H-6′b), 3.13 (dd, 1H, *J*_5__′__,6__′__a_ 5.4 Hz, H-6′a), 2.10, 2.08, 2.06, 2.03, 2.02, 2.01, 2.00, 1.99, 1.92, 1.89 (10s, each 3H, 10 × *Me*COO), 1.83 (1s, 3H, *Me*CON); ^13^C NMR (62.9 MHz, CD_3_CN): δ 171.4-170.4 (11 × *C*=O), 139.9 (Ar-*C*), 129.0–128.2 (Ar-*C*H), 102.8 (C-1), 101.4 (C-1″, C-1′″), 100.7 (C-1′), 80.5 (C-3), 77.7 (C-4), 76.6 (C-4″), 76.9 (C-5), 74.2 (*C*H_2_Ph), 73.3 (C-5″), 73.1 (C-3″), 72.6 (C-5′), 72.3 (C-2″), 71.3 (C-5′″), 71.6 (C-3′, C-3′″), 70.1 (C-2′″), 69.2 (C-6), 68.6 (C-4′″), 68.0 (C-4′), 68.8 (C-2′), 62.9 (C-6″), 61.9 (C-6′″), 56.9 (*Me*O), 54.6 (C-2), 50.7 (C-6′), 23.2 (*Me*CON), 21.1-20.7 (10 × *Me*COO). Elemental Analysis Found C, 51.63; H, 5.80; N, 4.49. Calculated for C_54_H_72_N_4_O_30_ (1257.17): C, 51.59; H, 5.77; N, 4.46.

*Methyl 2-acetamido-3-O-benzyl-4-O-(2,3,4,6-tetra-O-acetyl-β-d-galactopyranosyl)-6-O-[4-O-(benzyl-(2-O-benzyl-3,4-di-O-acetyl-β-d-galactopyranosyl) uronate)-2,3,6-tri-O-acetyl-β-d-glucopyranosyl]-2-deoxy-β-d-glucopyranoside* (**45**). The glycosylation of lactosamine acceptor **37** (159 mg, 0.24 mmol, 1 eq) with lacturonic donor **38** (321 mg, 0.36 mmol, 1.5 eq) was performed in dry CH_2_Cl_2_ (5.5 mL) with AW-300 MS (310 mg) and BF_3_^.^Et_2_O (36.4 μL, 0.315 mmol, 1.3 eq) in dry CH_2_Cl_2_ (0.5 mL), as described in the general procedure. Purification of crude product by flash chromatography on silica gel (2:8 hexane-EtOAc + 0.1% Et_3_N) afforded pure tetrasaccharide **45** (292 mg, 88%) as a white foam, *R*_f_ 0.24 (2:8 hexane-EtOAc); (α)_D_ -15.1 (*c* 1.1 in CHCl_3_); ^1^H NMR (250.13 MHz, CD_3_CN): δ 7.67–7.23 (m, 15H, Ar-*H*), 6.48 (d, 1H, *J*_2,NH_ 9.4 Hz, N*H*), 5.47 (dd, 1H, *J*_3′″,4′″_ 3.7 Hz, *J*_4′″,5′″_ 1.4 Hz, H-4′″), 5.30 (dd, 1H, *J*_2″,3″_ 10.3 Hz, *J*_3″,4″_ 8.9 Hz, H-3″), 5.32 (m, 1H, H-4′), 5.15, 5.08 (AB system, 2H, *J*_A,B_ 12.1 Hz, COOC*H*_2_Ph), 5.08 (m, 2H, H-3′, H-2′), 5.00 (dd, 1H, *J*_′″3′″_ 10.0 Hz, H-3′″), 4.84 (dd, 1H, *J*_1″,2″_ 7.9 Hz, H-2″), 4.73 (d, 1H, H-1″), 4.77, 4.54 (AB system, 2H, *J*_A,B_ 11.0 Hz, C*H*_2_Ph), 4.73, 4.57 (AB system, 2H, *J*_A,B_ 11.3 Hz, C*H*_2_Ph), 4.68 (d, 1H, *J*_1__′__,2__′_ 7.8 Hz, H-1′), 4.48 (d, 1H, H-5′″), 4.46 (d, 1H, *J*_1′″,2′″_ 7.9 Hz, H-1′″), 4.35 (dd, 1H, *J*_6__′__a,6__′__b_ 12.1 Hz, *J*_5__′__,6__′__b_ 2.0 Hz, H-6′b), 4.28 (d, 1H, *J*_1,2_ 7.9 Hz, H-1), 4.20 (dd, 1H, *J*_5__′__,6__′__a_ 4.9 Hz, H-6′a), 4.08-3.79 (m, 6H, H-6b, H-5′, H-5″, H-4″, H-6″a, H-6″b), 3.77-3.65 (m, 3H, H-2, H-4, H-6a), 3.53 (dd, 1H, *J*_2,3_ 9.5 Hz, *J*_3,4_ 8.2 Hz, H-3), 3.50 (m, 1H, H-5), 3.49 (dd, 1H, H-2′″), 3.41 (s, 3H, *Me*O), 2.10, 2.07, 2.03, 2.02, 1.98, 1.92, 1.88, 1.87, 1.85 (9s, each 3H, 9 × *Me*COO), 1.83 (s, 3H, *Me*CON); ^13^C NMR (62.9 MHz, CD_3_CN): δ 171.3–170.5 (10 × C=O), 167.0 (C-6′″), 139.8, 138.9, 136.4 (3 × Ar-*C*), 129.5-129.3 (Ar-*C*H), 103.3 (C-1′″), 102.8 (C-1′), 101.4 (C-1″), 101.1 (C-1), 80.6 (C-3), 78.2 (C-4), 77.3 (C-5), 77.0 (C-4″), 75.8, 74.0 (*C*H_2_Ph), 75.4 (C-5), 73.7 (C-5″), 72.9, 72.9, 72.8, 72.5, 72.4 (C-5′, C-2″, C-3″, C-5′″, C-3′″), 71.6, 71.4 (C-3′, C-2′″), (70.2 (C-2′), 69.5 (C-4′″), 69.2 (C-6), 68.1 (C-4′), 67.8 (COO*C*H_2_Ph), 62.9 (C-6′), 61.9 (C-6″), 57.0 (*Me*O), 54.6 (C-2), 23.3 (*MeC*ON), 21.0–20.5 (9 × *Me*COO). Elemental Analysis Found: C, 57.29; H, 5.94; N, 1.05. Calculated for C_66_H_81_NO_31_ (1384.35) C, 57.26; H, 5.90; N, 1.01.

*3-Azidopropyl 2-acetamido-3-O-benzyl-4-O-(2,3,4,6-tetra-O-acetyl-β-d-galactopyranosyl)-6-O-[4-O-(benzyl-(3,4-di-O-acetyl-2-O-benzyl-β-d-galactopyranosyl) uronate)-2,3,6-tri-O-acetyl-β-d-glucopyranosyl]-2-deoxy-β-d-glucopyranoside* (**46**). The glycosylation of lactosamine acceptor **39** (71 mg, 0.098 mmol, 1 eq) with lacturonic donor **28** (131 mg, 0.147 mmol, 1.5 eq) was performed in dry CH_2_Cl_2_ (3.0 mL) with AW-300 MS (175 mg) and BF_3_^.^Et_2_O (14.6 μL, 0.26 mmol, 1.3 eq) in dry CH_2_Cl_2_ (0.5 mL), as described in the general procedure. Purification of crude product by flash chromatography on silica gel (3:7 hexane-EtOAc + 0.1% Et_3_N) afforded pure tetrasaccharide **46** (107 mg, 75%) as a clear syrup, *R*_f_ 0.23 (3:7 hexane-EtOAc); (α)_D_ -14.9 (*c* 1.2 in CHCl_3_); ^1^H NMR (250.13 MHz, CD_3_CN): δ 7.39-7.24 (m, 15H, Ar-*H*), 6.60 (d, 1H, *J*_2,NH_ 9.3 Hz, N*H*), 5.48 (dd, 1H, *J*_3′″,4′″_ 3.7 Hz, *J*_4′″,5′″_ 1.4 Hz, H-4′″), 5.33 (m, 1H, H-4′), 5.29 (dd, 1H, *J*_2″,3″_ 9.4 Hz, *J*_3″,4″_ 8.0 Hz, H-3″), 5.16, 5.07 (AB system, 2H, *J*_A,B_ 12.1 Hz, COOC*H*_2_Ph), 5.10- 5.05 (m, 2H, H-3′, H-2′), 5.00 (dd, 1H, *J*_2′″,3′″_ 10.2 Hz, H-3′″), 4.82 (dd, 1H, *J*_1″,2″_ 7.4 Hz, H-2″), 4.78, 4.57 (AB system, 2H, *J*_A,B_ 11.7 Hz, C*H*_2_Ph), 4.73, 4.55 (AB system, 2H, *J*_A,B_ 11.3 Hz, C*H*_2_Ph), 4.69 (d, 1H, H-1″), 4.67 (d, 1H, *J*_1__′__,2__′_ 7.9 Hz, H-1′), 4.5 (d, 1H, H-5′″), 4.46 (d, 1H, *J*_1′″,2′″_ 7.9 Hz, H-1′″), 4.37 (d, 1H, *J*_1,2_ 8.2 Hz, H-1), 4.36 (dd, 1H, *J*_6″a,6″b_ 12.1 Hz, *J*_5″,6″b_ 1.7 Hz, H-6″b), 4.20 (dd, 1H, *J*_5″,6″a_ 5.1 Hz, H-6″a), 4.08–3.86 (m, 9H, H-5, H-5′, H-5″, H-4, H-4″, H-6a, H-6′a, H-6b, H-6′b), 3.85 (m, 2H, C*H*_2_O), 3.78–3.66 (m, 3H, H-2, H-4, H-4″), 3.55 (dd, 1H, *J*_2,3_ 9.8 Hz, *J*_3,4_ 8.4 Hz, H-3), 3.51 (m, 1H, H-5), 3.43 (dd, 1H, H-2′″), 3.36 (t, 1H, *J*_vic_ 6.7 Hz, C*H*_2_N_3_), 2.10, 2.07, 2.04, 2.03, 1.98, 1.92, 1.89, 1.87, 1.86 (9s, each 3H, 9 × *Me*COO), 1.84 (s, 3H, *Me*CON), 1.77 (m, 2H, C*H*_2_); ^13^C NMR (62.9 MHz, CD_3_CN,): δ 171.4-170.3 (10 × *C*=O), 167.0 (C-6′″), 139.9, 139.0, 136.4 (3 × Ar-*C*), 129.5-128.3 (Ar-*C*H), 103.3 (C-1′″), 101.9 (C-1), 101.4 (C-1″), 101.2 (C-1′), 80.6 (C-3), 78.2 (C-4″), 78.1 (C-4), 77.3 (C-2′″), 75.6 (C-5′″), 75.8, 74.1 (*C*H_2_Ph), 73.7 (C-5′), 72.9 (C-3″), 72.9 (C-5″), 72.6 (C-3′″), 72.4 (C-2″), 71.6 (C-3′), 71.4 (C-5), 70.3 (C-2′), 69.5 (C-4′″), 69.0 (C-6), 68.1 (C-4′), 67.9 (COO*C*H_2_Ph), 66.9 (*C*H_2_O), 62.9 (C-6″), 61.9 (C-6′), 54.9 (C-2), 48.8 (*C*H_2_N_3_), 29.5 (*C*H_2_), 23.3 (*MeC*ON), 21.1–20.5 (9 × *Me*COO). Elemental Analysis Found: C, 56.23; H, 5.84; N, 3.88. Calculated for C_68_H_84_N_4_O_31_ (1453.42): C, 56.20; H, 5.83; N, 3.85.

*Methyl 2-acetamido-3-O-benzyl-4-O-(2,3,4-tri-O-acetyl-6-azido-6-deoxy-β-d-galactopyranosyl)-6-O-[4-O-(benzyl-(3,4-di-O-acetyl-2-benzyl-β-d-galactopyranosyl) uronate)-2,3,6-tri-O-acetyl-β-d-glucopyranosyl]-2-deoxy-β-d-glucopyranoside* (**47**). The glycosylation of lactosamine acceptor **40** (145 mg, 0.227 mmol, 1 eq) with lacturonic donor **28** (303 mg, 0.34 mmol, 1.5 eq) was performed in dry CH_2_Cl_2_ (7.5 mL) with AW-300 MS (400 mg) and BF_3_^.^Et_2_O (33.8 μL, 0.292 mmol, 1.3 eq) in dry CH_2_Cl_2_ (0.5 mL), as described in the general procedure. Purification of crude product by flash chromatography on silica gel (2:8 hexane-EtOAc + 0.1% Et_3_N) afforded pure tetrasaccharide **47** (238 mg, 77%) as a white foam, *R*_f_ 0.35 (2:8 hexane-EtOAc); (α)_D_ -18.64 (*c* 1.2 in CHCl_3_); ^1^H NMR (250.13 MHz, CD_3_CN): δ 7.38–7.23 (m, 15H, Ar-*H*), 6.68 (d, 1H, *J*_2,NH_ 9.4 Hz, N*H*), 5.47 (dd, 1H, *J*_3′″,4′″_ 3.7 Hz, *J*_4′″, 5′″_ 1,5 Hz, H-4′″), 5.31 (m, 1H, H-4′), 5.27 (dd, 1H, *J*_2″,3″_ 9.6 Hz, *J*_3″,4″_ 8.8 Hz, H-3″), 5.16–5.06 (AB system, 2H, *J*_A,B_ 12.1 Hz, COOCH_2_Ph), 5.09 (m, 2H, H-3′, H-2′), 5.00 (dd, 1H, H-3′″), 4.84 (dd, 1H, *J*_1″,2″_ 7.9 Hz, H-2″), 4.84, 4.55 (AB system, 2H, *J*_A,B_ 11.0 Hz, C*H*_2_Ph), 4.75, 4.56 (AB system, 2H, *J*_A,B_ 11.3 Hz, C*H*_2_Ph), 4.73 (d, 1H, H-1″), 4.67 (d, 1H, *J*_2__′__,3__′_ 7.9 Hz, H-1′), 4.49 (d, 1H, H-5′″), 4.46 (d, 1H, *J*_1′″,2′″_ 7.9 Hz, H-1′″), 4.35 (dd, 1H, *J*_6″a,6″b_ 12.1 Hz, *J*_5″,6″b_ 1.5 Hz, H-6″b), 4.20 (dd, 1H, *J*_5″,6″a_ 4.9 Hz, H-6″a), 4.27 (d, 1H, *J*_1,2_ 7.8 Hz, H-1), 4.01 (dd, 1H, *J*_6a,6b_ 10.9 Hz, *J*_5,6b_ 2.2 Hz, H-6b), 3.93 (dd, 1H, *J*_5,6a_ 6.8 Hz, H-6a), 3.90 (m, 1H, H-5″), 3.90–3.65 (m, 3H, H-2, H-4, H-5), 3.87 (m, 1H, H-5′), 3.84 (m, 1H, H-4″), 3.53 (dd, 1H, *J*_2,3_ 9.4 Hz, *J*_3,4_ 8.1 Hz, H-3), 3.43 (dd, 1-H, *J*_2′″,3′″_ 10.1 Hz, H-2′″), 3.41 (s, 3H, OMe), 3.27 (dd, 1H, *J*_6__′__a,6__′__b_ 12.8 Hz, *J*_5__′__,6__′__b_ 7.3 Hz, H-6′b), 3.12 (dd, 1H, *J*_5__′__,6__′__a_ 5.6 Hz, H-6′a), 2.10, 2.06, 2.04, 2.03, 1.98, 1.91, 1.87, 1.85 (8s, each 3H, 8 × *Me*COO), 1.83 (1s, 3H, *Me*CON); ^13^C NMR (62.9 MHz, CD_3_CN): δ 171.6-170.5 (9 × *C*=O), 167.1 (C-6′″), 139.9, 138,9, 136.4 (Ar-*C*), 129.5-128.3 (Ar-*CH*), 103.3 (C-1′″), 102.9 (C-1), 101.5 (C-1″), 100.9 (C-1′), 80.6 (C-3), 77.9 (C-4″), 77.4 (C-2′″), 77.0 (C-4), 75.9, 74.4 (*C*H_2_Ph), 75.4 (C-5″), 73.7 (C-5′), 73.0 (C-5′″), 72.9 (C-3″), 72.6 (C-5, C-3′″), 72.4 (C-2″), 71.6 (C-3′), 70.2 (C-2′), 69.6 (C-4′″), 69.3 (C-6), 68.6 (C-4′), 67.9 (COO*C*H_2_Ph), 63.0 (C-6″), 57.0 (*Me*O), 54.7 (C-2), 50.8 (C-6′), 23.3 (*Me*CON), 21.1–20.5 (8 × *Me*COO). Elemental Analysis Found: C, 56.26; H, 5.78; N, 4.14. Calculated for C_64_H_78_N_4_O_29_ (1367.33): C, 56.22; H, 5,75; N, 4.10.

#### 3.1.3. Synthesis of 3-Azidopropyl 2-acetamido-4-O-β-d-galactopyranosyl-6-O-[4-O-(β-d-galactopyranosyl)-β-d-glucopyranosyl]-2-deoxy-β-d-glucopyranoside (**48**)

A solution of tetrasaccharide **43** (200 mg, 0.147 mmol) in MeOH (1 mL) was cooled at 0 °C, treated with a methanolic solution of MeONa (0.33M, 3 mL), and the reaction mixture was stirred at 0 °C until TLC analysis (1:1 CHCl_3_-MeOH) showed the complete disappearance of the starting material (48 h). The solution was neutralized with resin acid (Amberlist-15), filtered on a cotton pad, washed with MeOH and concentrated under diminished pressure. Purification of crude product by reversed-phase flash chromatography on C-18 silica gel (1:1 MeOH-H_2_O) followed by trituration with Et_2_O afforded pure tetrasaccharide **48** (80 mg, 69%) as a white solid, *R*_f_ 0.20 (4:6 CHCl_3-_MeOH); mp 159–161 °C (from MeOH-Isopropanol); (α)_D_ -6.05 (*c* 1.19 in MeOH); ^1^H NMR (250.13 MHz, CD_3_OD-D_2_O): δ 4.53 (d, 1H, *J*_1__″__,2″_ 8.0 Hz, H-1″), 4.47 (d, 1H, *J*_1′,2′_ 7.8 Hz, H-1′), 4.43 (d, 1H, *J*_1,2_ 8.3 Hz, H-1), 4.35 (d, 1H, *J*_1__′″__,2′″_ 7.7 Hz, H-1′″), 3.95-3.70 (m, 13H, H-2, H-3, H-4, H-4′, H-4′″, H-5a, H-6b, H-6′a, H-6′b, H-6″a, H-6″b, H-6′″a, H-6′″b), 3.67-3.54 (m, 11H, CH_2_O, H-2′, H-2′″, H-4″, H-5, H-5′, H-5″, H-5′″, H-2″, H-3″), 3.51–3.3.34 (m, 2H, H-3′, H-3′″), 3.37 (t, 2H, *J*_vic_ 6.7 Hz, C*H_2_*N_3_), 2.00 (s, 3H, *Me*CON), 1.81 (m, 2H, C*H_2_*); ^13^C NMR (62.9 MHz, CD_3_OD-D_2_O): δ 170.7(*C=*O), 104.7 (C-1′″), 104.4 (C-1′), 104.1 (C-1″), 102.8 (C-1), 80.1 (C-4″), 79.6 (C-4), 76.8 (C-5′), 76.6 (C-5′″), 76.2 (C-5″), 75.9 (C-5), 74.9 (C-2″), 74.3–73.7 (C-3′, C-3′″, C-3, C-3″), 72.4 (C-2′, C-2′″), 70.1 (C-4′, C-4′″), 68.6 (C-6), 67.7 (*C*H_2_O), 62.4 (C-6′, C-6′″), 61.5 (C-6″), 56.4 (C-2), 48.6 (*C*H_2_N_3_), 29.8 (*C*H_2_), 23.1 (*Me*CON). Elemental Analysis Found: C, 44.09; H, 6.41; N, 7.13. Calculated for C_29_H_50_N_4_O_21_ (790.73): C, 44.05; H, 6.37; N, 7.09.

#### 3.1.4. Synthesis of Methyl 2-acetamido-3-O-benzyl-4-O-(6-azido-6-deoxy-β-d-galactopyranosyl)-6-O-[4-O-(β-d-galactopyranosyl)-β-d-glucopyranosyl]-2-deoxy-β-d-glucopyranoside (**49**)

A solution of tetrasaccharide **44** (170 mg, 0.135 mmol) in MeOH (1.35 mL) was treated with 7N NH_3_-MeOH (1.35 mL) and the solution was stirred at room temperature until TLC analysis (3:7 MeOH-H_2_O, reversed-phase) revealed the complete disappearance of the starting material (20 h). The solution was concentrated under diminished pressure and the purification of crude product by reversed-phase flash chromatography on C-18 silica gel (3:7 MeOH-H_2_O) afforded pure tetrasaccharide **49** (73 mg, 65%) as a white foam, *R*_f_ 0.47 (3:7 MeOH-H_2_O); (α)_D_ -10.86 (*c* 1.04 in MeOH); ^1^H NMR (250.13 MHz, CD_3_OD-D_2_O): δ 7.36–7.19 (m, 5H, Ar-*H*), 4.95 (m, 2H, C*H*_2_Ph), 4.60 (d, 1H, *J*_1″,2″_ 7.9 Hz, H-1″), 4.42 (d, 1H, *J*_1′,2′_ 7.8 Hz, H-1′), 4.36 (d, 1H, *J*_1,2_ 8.1 Hz, H-1), 4.34 (d, 1H, *J*_1′″,2′″_ 7.9 Hz, H-1′″), 4.25 (m, 1H, H-6b), 4.08-3.42 (m, 21H, H-2, H-3, H-4, H-5, H-6a, H-2′, H-3′, H-4′, H-5′, H-2″, H-3″, H-4″, H-5″, H-6″a, H-6″b, H-2′″, H-3′″, H-4′″, H-5′″, H-6′″b, H-6′″a), 3.38-3.27 (m, 2H, H-6′a, H-6′b), 3.42 (s, 3H, *Me*O), 1.85 (s, 3H, *Me*CON); ^13^C NMR (62.9 MHz, CD_3_OD-D_2_O): δ 173.5 (*C*=O), 140.2 (Ar-*C*), 129.1–128.4 (Ar-*C*H), 104.8 (C-1), 104.4, 104.2 (C-1′, C-1″), 103.6 (C-1′″), 81.9 (C-3), 80.3 (C-4″), 78.0 (C-4), 76.9 (C-5), 76.3, 76.2, 75.5, 74.5, 74.4, 74.3, 74.0, 72.6, 72.4, 69.9, 69.8, (C-2′, C-3′, C-4′, C-5′, C-2″, C-3″, C-5″, C-2′″,C-3′″, C-4′″, C-5′″), 75.1 (CH_2_Ph), 68.1 (C-6), 62.4 (C-6″), 61.6 (C-6′″), 57.4 (O*Me*), 55.8 (C-2), 51.6 (C-6′), 23.3 (*MeC*ON). Elemental Analysis Found: C, 48.84; H, 6.27; N, 6.74. Calculated for C_34_H_52_N_4_O_20_ (836.80): C, 48.80; H, 6.23; N, 6.70.

#### 3.1.5. Synthesis of Methyl 2-acetamido-4-O-(2,3,4,6-tetra-O-acetyl-β-d-galactopyranosyl)-6-O-[4-O-(3,4-di-O-acetyl-β-d-galactopyranoyl uronic acid)-2,3,6-tri-O-acetyl-β-d-glucopyranosyl]-2-deoxy-β-d-glucopyranoside (**50**)

Compound **45** (70.6 mg, 0.051 mmol) in 2.5:1 MeOH-EtOAc (3.5 mL) was treated with 10% Pd on activated charcoal (8 mg). The suspension was stirred at room temperature under H_2_ atmosphere until TLC analysis (8:2 CHCl_3_-MeOH) revealed the complete disappearance of the starting material (48 h) and the formation of a product at *R*_f_ 0.16 (no UV visible spot). The suspension was filtered on a cotton pad, washed with MeOH and concentrated under diminished pressure. Purification of crude product by flash chromatography on silica gel (8:2 CHCl_3_-MeOH) gave the uronic acid pure **50** (55.7 mg, 98%) as a white foam, *R*_f_ 0.16 (8:2 CHCl_3_-MeOH); (α)_D_ +4.74 (*c* 1.16 in CHCl_3_); ^1^H NMR (250.13 MHz, CDCl_3_-CD_3_OD): δ 5.61 (bd, 1H, H-4′″), 5.38 (m, 1H, H-4′), 5.20-4.90 5.11-4.90 (m, 4H, H-3″, H-3′″, H-2′, H-3′), 4.80 (m, 3H, H-1′, H-1″, H-2″), 4.40–4.20 (m, 3H, H-1′″, H-1, H-6′b,), 4.10–4.02 (m, 2H, H-6′a, H-6″b H-5′″, H-6″a), 4.00-3.88 (m, 2H, H-5′, H-6a), 3.77–3.15 (m, 8H, H-6b, H-5″, H-4, H-4″, H-2, H-3, H-5, H-2′″), 3.43 (s, 3H, *Me*O), 2.13, 2.07, 2.06, 1.98 (4s, each 3H, 4 × *Me*COO), 2.02 (s, 9H, 3 × *Me*COO), 1.95 (s, 6H, 2 × *Me*COO), 1.64 (s, 3H, *Me*CON); ^13^C NMR (62.9 MHz, CDCl_3_-CD_3_OD): δ 172.4-170.2 (10 × *C*=O, C-6′″), 104.3 (C-1′″), 101.8 (C-1), 101.5 (C-1′), 100.9 (C-1″), 81.9 (C-3), 77.0 (C-4″), 75.2 (C-5), 74.2 (C-3″), 74.1 (C-5″), 74.0 (C-3′), 73.6 (C-3′″), 73.1 (C-4), 72.6 (C-5′), 72.2 (C-2″), 71.3 (C-5′″), 71.2 (C-2′), 69.2 (C-2′″), 69.8 (C-4′″), 69.0 (C-6), 67.4 (C-4′), 63.0 (C-6′), 61.7 (C-6″), 56.7 (*Me*O), 55.2 (C-2), 22.9 (*MeC*ON), 20.9-20.6 (9 × *Me*COO). Elemental Analysis Found: C, 48.55; H, 5.74; N, 1.29. Calculated for C_45_H_63_NO_31_ (1113.98): C, 48.52; H, 5.70; N, 1.26.

#### 3.1.6. Synthesis of 3-Ammoniumpropyl 2-acetamido-4-O-(2,3,4,6-tetra-O-acetyl-β-d-galactopyranosyl)-6-O-[4-O-(3,4-di-O-acetyl-2-benzyl-β-d-galactopyranosyl uronate)-2,3,6-tri-O-acetyl-β-d-glucopyranosyl]-2-deoxy-β-d-glucopyranoside (**51**)

A solution of **46** (142.4 mg, 0.098 mmol) in 3:1:0.5 MeOH-CH_2_Cl_2_-H_2_O (14 mL) was treated with 10% Pd on activated charcoal (74.4 mg). The suspension was stirred at room temperature under H_2_ atmosphere until TLC analysis (7:3 CHCl_3_-MeOH) revealed the complete disappearance of the starting material (48 h). The suspension was filtered on a cotton pad, washed with MeOH and concentrated under diminished pressure. Purification of crude product by flash chromatography on silica gel (7:3 CHCl_3_-MeOH) gave pure zwitterionic tetrasaccharide **51** (80 mg, 70%) as a white solid, *R*_f_ 0.23 (7:3 CHCl_3_-MeOH); mp 193–195 °C (after trituration with Et_2_O); (α)_D_ +2.57 (*c* 1.13 in MeOH); ^1^H NMR (250.13 MHz, CD_3_OD) led to identify only some signals as: δ 4.72 (m, 2H, H-1′, H-1″), 4.46 (d, 1H, *J*_1′″,2′″_ 7.9 Hz, H-1′″), 4.36 (d, 1H, *J*_1,2_ 8.1 Hz, H-1), 4.01 and 3.63 (2m, each 1H, C*H*_2_O), 3.15 (bt, 1H, *J*_vic_ 6.8 Hz, C*H*_2_NH_3_^+^), 2.15, 2.11, 2.10, 2.07, 2.05, 2.03, 1.98, 1.95, 1.94 (9s, each 3H, 9 × *Me*COO), 1.91 (s, 3H, *Me*CON), 1.88 (m, 2H, C*H_2_*); ^13^C NMR (62.9 MHz, CD_3_OD): δ 174.1 (C-6′″), 173.0-171.2 (10 × *C*=O), 104.9 (C-1′″), 102.4 (C-1), 101.9 (C-1′), 101.8 (C-1″), 82.1 (C-4″), 78.0 (C-4), 75.0 (C-5″), 74.7 (C-5, C-2″, C-3″, C-5′″), 74.2 (C-5′), 73.3 (C-3), 72.2, 72.1, 70.9, 70.4 (C-2′″, C-3′″, C-3′, C-2′), 69.9 (C-4′″), 69.7 (C-6), 68.6 (C-4′), 68.5 (*C*H_2_O), 63.9 (C-6″), 62.7 (C-6′), 56.4 (C-2), 39.3 (*C*H_2_NH_3_^+^), 28.1 (*C*H_2_), 23.0 (*MeC*ON), 21.3-20.5 (9 × *Me*COO). Elemental Analysis Found: C, 48.82; H, 5.95; N, 2.44. Calculated for C_47_H_68_N_2_O_31_ (1157.04): C, 48.79; H, 5.92; N, 2.42.

#### 3.1.7. Synthesis of Methyl 2-acetamido-4-O-(6-ammonium-2,3,4-tri-O-acetyl-6-deoxy-β-d-galactopyranosyl)-6-O-[4-O-(3,4-di-O-acetyl-β-d-galactopyranosyl uronate)-2,3,6-tri-O-acetyl-β-d-glucopyranosyl]-2-deoxy-β-d-glucopyranoside (**52**).

A solution of **47** (238 mg, 0.174 mmol) in 3:1:0.5 MeOH-CH_2_Cl_2_-H_2_O (13.5 mL) was treated with 10% Pd on activated charcoal (131 mg). The suspension was stirred at room temperature under H_2_ atmosphere until TLC analysis (7:3 CHCl_3_-MeOH) revealed the complete disappearance of the starting material (48 h). The suspension was filtered on a cotton pad, washed with MeOH and concentrated under diminished pressure. Purification of crude product by reversed-phase flash chromatography on C-18 silica gel (4:6 MeOH-H_2_O) gave pure zwitterionic tetrasaccharide **52** (141 mg, 76%) as a white solid, *R*_f_ 0.40 (4:6 MeOH-H_2_O, reversed-phase); mp 195–197 °C (after trituration with Et_2_O); (α)_D_ –1.75 (*c* 1.03 in MeOH); ^1^H NMR (250.13 MHz, CD_3_OD-D_2_O): δ 5.49 (m, 1H, H-4′″), 5.31 (m, 1H, H-4′), 5.23 (m, 1H, H-3″), 5.12-4.91 (m, 2H, H-3′, H-2′), 4.90–4.70 (m, 2H, H-3′″, H-2″), 4.75 (bd, 2H, *J* 7.5 Hz, H-1″, H-1′), 4.54-4.20 (m, 5H, H-1′″, H-5′″, H-1, H-6″b, H-6″a), 4.13- 3.88 (m, 4H, H-6b, H-6a, H-5″, H-5′), 3.82- 3.40 (m, 6H, H-2, H-4, H-5, H-4″, H-3, H-2′″), 3.47 (s, 3H, OMe), 3.10-2.95 (m, 2H, H-6′a, H-6′b), 2.10-1.88 (m, 27H, 9 × *Me*CO); ^13^C NMR (62.9 MHz, CD_3_OD-D_2_O): δ 174.6-172.3 (9 × C=O, C-6′″), 104.3 (C-1′″), 103.0 (C-1), 101.9 (C-1″), 101.3 (C-1′), 79.9 (C-3), 77.0 (C-4″), 75.4 (C-2″), 74.6 (C-4, C-5′, C-5″), 73.8, 73.5, 73.2, 72.0 (C-5′″, C-3″, C-3′″, C-5), 71.0 (C-3′, C-2′″), 70.5 (C-2′), 69.9 (C-6), 69.7 (C-4′″), 69.5 (C-4′), 64.0 (C-6″), 57.5 (O*Me*), 56.5 (C-2), 40.8 (C-6′), 23.1 (*Me*CON), 21.5-20.7 (8 ×*Me*COO). Elemental Analysis Found: C, 48.26; H, 5.87; N, 2.65. Calculated for C_43_H_62_N_2_O_29_ (1070.95): C, 48.23; H, 5.84; N, 2.62.

#### 3.1.8. Synthesis of Methyl 2-acetamido-4-O-β-d-galactopyranosyl-6-O-[4-O-(β-d-galactopyranosyl)-β-d-glucopyranosyl]-2-deoxy-β-d-glucopyranoside (**2**)

A solution of **42** (99.7 mg, 0.0774 mmol) in MeOH (1 mL) was treated with a methanolic solution of MeONa (0.33 M, 3 mL) and the reaction mixture was stirred at 0°C until TLC analysis (9:1 CHCl_3_-MeOH) showed the complete disappearance of the starting material (12 h). The solution was neutralized with resin acid (Amberlist-15), filtered on a cotton pad, washed with MeOH and concentrated under diminished pressure. Crystallization of the crude product with EtOH-H_2_O afforded pure tetrasaccharide **2** (40 mg, 72%) as a white solid, *R*_f_ 0.10 (9:1 CHCl_3_-MeOH); mp 158-160 °C (from EtOH-H_2_O); (α)_D_ –14.42 (*c* 1.2 in H_2_O); Lit [44] (α)_D_ +1 (*c* 1 in H_2_O); Lit [49] (α)_D_ –4.40 (*c* 2.75 in H_2_O); ^1^H NMR (600 MHz, D_2_O): δ 4.45 (d, 1H, *J*_1″,2″_ 8.02 Hz, H-1″), 4.43 (d, 1H, *J*_1__′,2__′_ 7.8 Hz, H-1′), 4.36 (d, 1H, *J*_1,2_ 8.3 Hz, H-1), 4.34 (d, 1H, *J*_1′″,2′″_ 8.2 Hz, H-1′″), 4.19 (dd, 1H, *J*_6a,6b_ 11.1 Hz, *J*_5,6b_ 1.2 Hz, H-6b), 3.85 (dd, 1H, *J*_5,6a_ 3.7 Hz, H-6a), 3.87 (m, 1H, H-6″a), 3.82 (m, 1H, H-4′″), 3.81 (m, 1H, H-4′), 3.71 (m, 1H, H-6″b), 3.65 (m, 4H, H-6′a, H-6′b, H-6′″a, H-6′″b), 3.74 (m, 1H, H-4), 3.63 (m, 1H, H-2), 3.61 (m, 1H, H-5), 3.59 (m, 1H, H-5′), 3.57 (m, 1H, H-3″), 3.567 (m, 1H, H-5′″), 3.56 (m, 4H, H-3, H-4′″, H-3′″, H-3′), 3.49 (m, 1H, H-5″), 3.43 (m, 2H, H-2′, H-2′″), 3.40 (s, 3H, O*Me*), 3.27 (m, 1H, H-2″), 1.93 (s, 3H, *Me*CON); ^13^C NMR (D_2_O): δ 174.3 (*C*=O), 102.8 (C-1′″), 102.6 (C-1′), 102.2 (C-1″), 102.0 (C-1), 78.3 (C-4″), 77.7 (C-4), 75.2 (C-5′), 74.6 (C-5″), 74.4 (C-5′″), 74.2 (C-3′, C-3′″), 73.4 (C-5), 72.6 (C-2″), 72.4 (C-3), 72.3 (C-3″), 70.9 (C-2′, C-2′″), 68.5 (C-4′, C-4′″), 67.1 (C-6), 61.0 (C-6′, C-6′″), 59.9 (C-6″), 57.7 (*Me*O), 54.9 (C-2), 23.1 (*Me*CON). Elemental Analysis Found: C, 44.98; H, 6.59; N, 1.97. Calculated for C_27_H_47_NO_21_ (721.67): C, 44.94; H, 6.56; N, 1.94.

#### 3.1.9. Synthesis of Methyl 2-acetamido-4-O-β-d-galactopyranosyl-6-O-[4-O-(β-d-galactopyranosyl uronate)-β-d-glucopyranosyl]-2-deoxy-β-d-glucopyranoside Ammonium Salt (**3**)

A solution of **50** (40.7 mg, 0.0365 mmol) in MeOH (0.5 mL) was treated with 7N NH_3_-MeOH (0.5 mL) and the reaction mixture was stirred at room temperature until TLC analysis (6:4 CHCl_3_-MeOH) showed the complete disappearance of the starting material (48 h). The solution was concentrated under diminished pressure and purification of crude product by reversed-phase flash chromatography on C-18 silica gel (1:3 MeOH-H_2_O) gave pure tetrasaccharide **3** (24.4 mg, 89%) as a white solid, *R*_f_ 0.0 (8:2 CHCl_3_-MeOH); mp 241–243 °C (from isopropanol-MeOH); (α)_D_ –50.6 (*c* 0.9 in MeOH); ^1^H NMR (600 MHz, D_2_O): δ 4.46 (d, 1H, *J*_1__″__,2″_ 7.99 Hz, H-1″), 4.43 (d, 1H, *J*_1′,2′_ 7.87 Hz, H-1′), 4.36 (d, 1H, *J*_1,2_ 8.37 Hz, H-1), 4.34 (d, 1H, *J*_1__′″__,2′″_ 7.76 Hz, H-1′″), 4.20 (dd, 1H, *J*_6a,6b_ 10.9 Hz, *J*_5,6b_ 1.1 Hz, H-6b), 4.11 (m, 1H, H-4′″), 3.99 (m, 1H, H-5′″), 3.85 (dd, 1H, *J*_5,6a_ 3.1 Hz, H-6a), 3.81 (m, 1H, H-4′), 3.74 (m, 1H, H-4), 3.72 (m, 1H, H-6″b), 3.66 (m, 1H, H-6″a), 3.64 (m, 1H, H-2), 3.63 (m, 2H, 6′a, H-6′b), 3.62 (m, 1H, H-5), 3.60 (m, 1H, H-3′″), 3.60 (m, 1H, H-5′), 3.59 (m, 1H, H-3″), 3.58 (m, 2H, H-3, H-3′), 3.52 (m, 1H, H-4″), 3.507 (m, 1H, H-5″), 3.48 (m, 1H, H-2′″), 3.45 (m, 1H, H-2′), 3.44 (s, 3H, *Me*O), 3.29 (m, 1H, H-2″), 1.93 (s, 3H, *Me*CON); ^13^C NMR (150 MHz, D_2_O): δ 178.2, 175.4 (*MeC*O, C-6′″), 102.5 (C-1′), 102.5 (C-1′″), 102.3 (C-1″), 102.0 (C-1), 78.9 (C-4″), 77.6 (C-4), 75.5 (C-4′″), 75.2 (C-5′), 75.1 (C-5′″), 74.6 (C-5″), 74.4 (C-3′), 73.5 (C-2′″), 73.4 (C-5), 73.4 (C-2′), 72.6 (C-2″), 72.4 (C-3, C-3″), 70.0 (C-3′″), 68.6 (C-4′), 67.2 (C-6), 61.0 (C-6′), 59.9 (C-6″), 54.9 (C-2), 58.2 (*Me*O), 22.2 (*MeC*ON). Elemental Analysis Found: C, 43.12; H, 6.46; N, 3.75. Calculated for C_27_H_48_N_2_O_22_ (752.67): C, 43.09; H, 6.43; N, 3.72.

#### 3.1.10. Synthesis of 3-Aminopropyl 2-acetamido-4-O-β-d-galactopyranosyl-6-O-[4-O-(β-d-galactopyranosyl)-β-d-glucopyranosyl]-2-deoxy-β-d-glucopyranoside Hydrochloride (**4**)

A solution of **48** (59.3 mg, 0.075 mmol) in 3:1 MeOH-H_2_O (4 mL) was turned to pH 3 adding few drops of 1% methanolic HCl (0.46 mL) and treated with 10% Pd on activated charcoal (42.2 mg). The suspension was stirred at room temperature under H_2_ atmosphere until TLC analysis (2:8 MeOH-H_2_O, reversed-phase) revealed the complete disappearance of the starting material (20 h). The suspension was filtered on a cotton pad, washed with MeOH and concentrated under diminished pressure. Crystallization of the crude product with EtOH afforded pure tetrasaccharide **4** (43 mg, 71%) as a white solid, *R*_f_ 0.98 (2:8 MeOH-H_2_O, reversed-phase); mp 183–185 °C (from EtOH); (α)_D_ –4.3 (*c* 0.72 in MeOH); ^1^H NMR (600 MHz, D_2_O): δ 4.45 (d, 1H, *J*_1″,2″_ 8.1 Hz, H-1″), 4.43 (d, 1H, *J*_1′,2′_ 8.03 Hz, H-1′), 4.42 (d, 1H, *J*_1,2_ 8.3 Hz, H-1), 4.34 (d, 1H, *J*_1′″,2′″_ 7.85 Hz, H-1′″), 4.19 (dd, 1H, *J*_6a,6b_ 10.9 Hz, *J*_5,6b_ 1.1 Hz, H-6b), 3.87 (m, 1H, H-6″b), 3.85 (dd, 1H, *J*_5,6b_ 3.1 Hz, H-6a), 3.82 (m, 1H, H-4′″), 3.81 (m, 1H, H-4′), 3.75 (m, 1H, H-4), 3.71 (m, 1H, H-6″a), 3.68 (m, 2H, C*H_2_*O), 3.65 (m, 2H, H-6′a, H-6′b), 3.64 (m, 3H, H-2, H-6′″a, H-6′″b,), 3.61 (m, 2H, H-5, H-5′″), 3.60 (m, 1H, H-5′), 3.59 (m, 1H, H-3), 3.57 (m, 2H, H-4″, H-3″), 3.56 (m, 1H, H-3′), 3.55 (m, 1H, H-3′″), 3.50 (m, 1H, H-5″), 3.44 (m, 2H, H-2′, H-2′″), 3.27 (m, 1H, H-2″), 3.01 (bt, 2H, *J*_vic_ 6.8 Hz, C*H_2_*N_3_), 2.01 (s, 3H, *Me*CON), 1.91 (m, 2H, C*H_2_*); ^13^C NMR (150 MHz, D_2_O): δ 175.7 (*C=*O), 102.9 (C-1′″), 102.6 (C-1′), 102.3 (C-1″), 101.3 (C-1), 78.3 (C-4″), 77.5 (C-4), 75.3 (C-5′, C-5′″), 74.6 (C-5″), 74.2 (C-3′, C-3′″), 73.3 (C-5), 72.6 (C-2″), 72.5 (C-3″), 72.2 (C-3), 70.9 (C-2′″, C-2′), 68.5 (C-4′″, C-4′), 68.3 (*C*H_2_O), 67.1 (C-6), 61.0 (C-6′″), 60.9 (C-6′), 59.8 (C-6″), 55.1 (C-2), 38.6 (*C*H_2_N_3_), 27.6 (*C*H_2_), 23.2 (*Me*CON). Elemental Analysis Found: C, 43.51; H, 6.69; N, 3.54. Calculated for C_29_H_52_ClN_2_O_21_ (801.19): C, 43.48; H, 6.67; N, 3.50.

#### 3.1.11. Synthesis of 3-Ammoniumpropyl 2-acetamido-4-O-β-d-galactopyranosyl-6-O-[4-O-(β-d-galactopyranosyl uronate)-β-d-glucopyranosyl]-2-deoxy-β-d-glucopyranoside (**5**)

A solution of **51** (60.1 mg, 0.052 mmol) in MeOH (1.0 mL) was treated with 7N NH_3_-MeOH (1.0 mL) and the solution was stirred at room temperature until TLC analysis (4:6 MeOH-H_2_O, reversed-phase) revealed the complete disappearance of the starting material (48 h). The solution was concentrated under diminished pressure and the purification of crude product by reversed-phase flash chromatography on C-18 silica gel (H_2_O) afforded pure tetrasaccharide **5** (39.8 mg, 98%) as a white solid, *R*_f_ 0.95 (H_2_O); mp 194–195 °C (after trituration with MeOH); (α)_D_ –38.8 (*c* 0.99 in H_2_O); ^1^H NMR (600 MHz, D_2_O): δ 4.47 (d, 1H, *J*_1″,2″_ 7.97 Hz, H-1″), 4.43 (d, 1H, *J*_1′,2′_ 7.76 Hz, H-1′), 4.42 (d, 1H, *J*_1,2_ 8.3 Hz, H-1), 4.33 (d, 1H, *J*_1′″,2′″_ 7.86 Hz, H-1′″), 4.19 (dd, 1H, *J*_6a,6b_ 10.9 Hz, *J*_5,6b_ 1.2 Hz, H-6b), 4.12 (m, 1H, H-4′″), 3.98 (m, 1H, H-5′″), 3.85 (dd, 1H, *J*_5,6a_ 3.7 Hz, H-6a), 3.86 (m, 1H, H-6″b), 3.81–3.72 (m, 3H, H-4′, H-4, H-6″a), 3.67–3.51 (m, 13H, H-3′″, H-2, H-6′a, H-6′b, C*H*_2_O, H-5, H-3, H-5′, H-3″, H-3′, H-4″, H-5″), 3.47 (m, 1H, H-2′″), 3.43 (m, 1H, H-2′), 2.98 (bt, 1H, *J*_vic_ 6.8 Hz, C*H*_2_NH_3_^+^), 3.27 (m, 1H, H-2″), 1.91 (s, 3H, *Me*CON), 1.82 (m, 2H, C*H_2_*); ^13^C NMR (150 MHz, D_2_O): δ 175.7 (*C=*O), 175.6 (C-6′″), 102.6 (C-1′), 102.4 (C-1′″), 102.2 (C-1″), 101.4 (C-1), 78.7 (C-4″), 77.5 (C-4), 75.6 (C-5′″), 75.0 (C-5′), 74.6 (C-5″), 73.3 (C-5), 72.7 (C-2″), 72.4 (C-3″, C-3′″), 72.3 (C-3, C-3′), 70.9 (C-2′), 70.4 (C-2′″), 69.9 (C-4′″), 68.4 (C-4′), 68.3 (*C*H_2_O), 67.1 (C-6), 60.9 (C-6′), 59.9 (C-6″), 55.0 (C-2), 38.6 (*C*H_2_NH_3_^+^), 27.6 (*C*H_2_), 23.1 (*MeC*ON). Elemental Analysis Found: C, 44.76; H, 6.49; N, 3.64. Calculated for C_29_H_50_N_2_O_22_ (778.71): C, 44.73; H, 6.47; N, 3.60.

#### 3.1.12. Synthesis of Methyl 2-acetamido-4-O-(6-amino-6-deoxy-β-d-galactopyranosyl)-6-O-[4-O-(β-d-galactopyranosyl)-β-d-glucopyranosyl]-2-deoxy-β-d-glucopyranoside Hydrochloride (**6**)

A solution of **49** (52 mg, 0.062 mmol) in 3:1 MeOH-H_2_O (2.5 mL) was turned to pH 3 adding few drops of 1% methanolic HCl (0.51 mL) and treated with 10% Pd on activated charcoal (50 mg). The suspension was stirred at room temperature under H_2_ atmosphere until TLC analysis (1:9 MeOH-H_2_O, reversed-phase) revealed the complete disappearance of the starting material (20 h). The suspension was filtered on a cotton pad, washed with MeOH and concentrated under diminished pressure. Purification of crude product by reversed-phase flash chromatography on C-18 silica gel (1:9 MeOH-H_2_O) afforded pure tetrasaccharide **6** (45 mg, 96%) as a white solid, *R*_f_ 0.47 (7:3 MeOH-H_2_O, reversed-phase); mp 183–186 °C (from MeOH-isopropanol); (α)_D_ –19.9 (*c* 1.0 in MeOH); ^1^H NMR (250.13 MHz, CD_3_OD-D_2_O): δ 4.67 (d, 1H, *J*_1″,2″_ 7.6 Hz, H-1″), 4.42 (d, 1H, *J*_1′,2′_ 7.8 Hz, H-1′), 4.39 (d, 1H, *J*_1′″,2′″_ 7.9 Hz, H-1′″), 4.34 (d, 1H, *J*_1,2_ 8.2 Hz, H-1), 4.28 (m, 1H, H-6b), 4.10-3.51 (m, 21H, H-2, H-3, H-4, H-5, H-6a, H-2′, H-3′, H-4′, H-5′, H-2″, H-3″, H-4″, H-5″, H-6″a, H-6″b, H-2′″, H-3′″, H-4′″, H-5′″, H-6′″b, H-6′″a), 3.45 (s, 3H, *Me*O), 3.38–3.21 (m, 2H, H-6′a, H-6′b), 1.99 (s, 3H, *Me*CON); ^13^C NMR (62.9 MHz, CD_3_OD-D_2_O): δ 173.3 (*MeC*O), 104.8 (C-1), 103.8, 103.5, 103.4 (C-1′, C-1″, C-1′″), 80.2 (C-4″), 77.8 (C-4), 76.7 (C-3, C-5), 76.1, 75.0, 74.4, 74.4, 74.0, 73.9, 72.2, 71.9, 71.6, 70.9, 69.9 (C-2′, C-3′, C-4′, C-5′, C-2″, C-3″, C-5″, C-2′″, C-3′″, C-4′″, C-5′″), 67.7 (C-6), 62.2 (C-6″), 61.4 (C-6′″), 57.1 (O*Me*), 56.2 (C-2), 41.7 (C-6′), 22.6 (*MeC*ON). Elemental Analysis Found: C, 42.86; H, 6.55; N, 3.72. Calculated for C_27_H_49_ClN_2_O_20_ (757.13): C, 42.83; H, 6.52; N, 3.70.

#### 3.1.13. Synthesis of Methyl 2-acetamido-4-O-(6-ammonium-6-deoxy-β-d-galactopyranosyl)-6-O-[4-O-(β-d-galactopyranosyl uronate)-β-d-glucopyranosyl]-2-deoxy-β-d-glucopyranoside (**7**)

A solution of **52** (39.1 mg, 0.0365 mmol) in MeOH (1.0 mL) was treated with 7N NH_3_-MeOH (1.0 mL) and the solution was stirred at room temperature until TLC analysis (H_2_O, reversed-phase) revealed the complete disappearance of the starting material (48 h). The solution was concentrated under diminished pressure and the purification of crude product by reversed-phase flash chromatography on C-18 silica gel (H_2_O) afforded pure tetrasaccharide **7** (23 mg, 86%) as a white solid, *R*_f_ 0.95 (H_2_O, reversed-phase); mp 226–228 °C (from MeOH-EtOH); (α)_D_ –16.67 (*c* 1.05 in H_2_O), ^1^H NMR (250.13 MHz, D_2_O): δ 4.46 (d, 1H, *J*_1″,2″_ 7.9 Hz, H-1″), 4.45 (d, 1H, *J*_1′,2′_ 7.3 Hz, H-1′), 4.40 (d, 1H, *J*_1,2_ 8.4 Hz, H-1), 4.37 (d, 1H, *J*_1′″,2′″_ 8.1 Hz, H-1′″), 4.12 (m, 1H, H-6b), 4.03 (m, 1H, H-4′″), 3.91 (m, 1H, H-5′″), 3.85-3.63 (m, 4H, H-4′, H-4, H-5, H-6b), 3.60-3.41 (m, 12H, H-2′″, H-3′, H-4″, H-3′″, H-3″, H-6″a, H-6″b, H-5′, H-5″, H-2, H-3, H-6a,), 3.40-3.10 (m, 5H, H-2′, H-2″, H-2, H-6′a, H-6′b), 3.33 (s, 3H, O*Me*), 1.85 (s, 3H, *Me*CON); ^13^C NMR (62.9 MHz, D_2_O): δ 175.7, 175.6 (*C=*O, C-6′″), 103.9 (C-1′), 103.7(C-1′″), 103.4 (C-1″), 103.0 (C-1), 80.1 (C-4″), 79.3 (C-4), 75.7 (C-5′″), 75.5 (C-5′), 74.5 (C-5″, C-2′″), 73.9 (C-2″), 73.8-73.7 (C-5, C-3″, C-3), 73.4 (C-3′″), 73.3 (C-3′), 71.8 (C-2′), 71.4 (C-4′″), 69.9 (C-4′), 68.4 (C-6), 61.1 (C-6″), 58.3 (O*Me*), 56.0 (C-2), 40.8 (C-6′), 23.2 (*MeC*ON). Elemental Analysis Found: C, 44.17; H, 6.34; N, 3.84. Calculated for C_27_H_46_N_2_O_21_ (734.66): C, 44.14; H, 6.31; N, 3.81.

### 3.2. Biological Methods

#### 3.2.1. Test ELISA

96-Well flat-bottomed plates were incubated overnight at 4–8 °C with a mixture of SP14 CPS (1 mg/mL) (American Type Culture Collection, ATCC, Manassas, VA, USA) and methylated human serum albumin (1 mg/mL). A solution of fetal calf serum (5%) in phosphate-buffered saline supplemented with Brij-35 (0.1%) and sodium azide (0.05%) was applied to the plates for blocking of nonspecific binding sites. The plates were incubated overnight at 4–8 °C with a solution (1:200) of rabbit anti-SP14 (Thermo Fisher Scientific, Rockford, IL, USA), used as reference serum. When SP14 analogous were tested, they were added to each well immediately before the addition of the reference serum. The plates were then incubated with alkaline phosphatase conjugate goat anti-rabbit IgG stained with *p*-nitrophenylphosphate (Sigma Aldrich, Milan Italy), and the absorbance was measured at 405 nm with an Ultramark microplate reader.

#### 3.2.2. Statistical Analysis

Results are expressed as the mean + SEM of at least five different experiments run in triplicate. Statistical significance was evaluated by Student′s *t*-test for unpaired varieties. Differences were considered statistically significant when *p* ≤ 0.05.

## 4. Conclusions

A synthetic approach for the synthesis of charged analogues of the smallest immunogenic structure of *Streptococcus pneumoniae* type 14 (SP14) capsular polysaccharide was explored. Suitable lacturonic and lactose donors were coupled with modified lactosamine acceptors which allowed, after the final deprotection, to obtain two cationic, an anionic as well as two zwitterionic tetrasaccharide target structures. From the synthetic perspective, the proposed block strategy proved to be feasible, permitting to build up several point-charged structures. The biological data we reported for the prepared tetrasaccharides clearly demonstrate that the introduction of positive and/or negative charges into the basic unit of SP14 CPS do not modify the affinity of a neutral compound to binding to a specific antibody. In our opinion, these results would represent an encouraging starting point for future studies on the ability of these charged compounds to stimulate immune cell responses. All analogues exhibited similar efficacies, which are lower than the natural SP-14 compound. This might be related to the length of synthetic fragments, too short to cover the entire SP14 CPS epitopes.

## Figures and Tables

**Table 1:** Results of the competitive ELISA assay.

Compound	IC_50_ (mg/mL)	Maximum Inhibition^a^ (%)
**SP-14**	28 × 10^−5^ ± 1.2	100 ± 3
**2**	1.2 × 10^−5^ ± 0.5	30 ± 5
**3**	1.4 × 10^−5^ ± 0.4	31 ± 3
**4**	4.1 × 10^−5^ ± 0.3	31 ± 2
**5**	5.1 × 10^−5^ ± 0.4	31 ± 3
**6**	2.8 × 10^−5^ ± 0.2	33 ± 4
**7**	3.8 × 10^−5^ ± 0.3	34 ± 5

^a^ The maximum inhibition elicited by each compound at 1 mg/mL.

## Supplementary Materials

The following are available online, experimental procedures and characterization data of compound **10–23**, **25–28** and **31–40**. NMR spectra of new compounds.
