# Supplementary material for: Selectively Charged and Zwitterionic Analogues of the Smallest Immunogenic Structure of Streptococcus Pneumoniae Type 14
Source: Molecules. 2019 Sep 19;24(18):3414. doi: 10.3390/molecules24183414 (PMC6767069; doi:10.3390/molecules24183414)
Supplement: Supplementary file 1 [file molecules-24-03414-s001.pdf]

Supporting Information

for

Selectively charged and zwitterionic analogues of the smallest immunogenic structure of Streptococcus pneumoniae type 14

Tiziana Gragnani[1], Doretta Cuffaro[1], Silvia Fallarini[2], Grazia Lombardi[2], Felicia D'Andrea[1,*], Lorenzo Guazzelli[1,*]

[1]Dipartimento di Farmacia, University of Pisa, Via Bonanno 6/33, 56126, Pisa, Italy

[2]Dipartimento di Scienze del Farmaco, University of Piemonte Orientale "Amedeo Avogadro", Largo Donegani 2, 28100 Novara, Italy

Corresponding Authors:
Lorenzo Guazzelli, E-mail: lorenzo.guazzelli@unipi.it;
Felicia D'Andrea, E-mail: felicia.dandrea@unipi.it.

Contents

Experimental procedures, characterization data of **10-23**	S2-S9
Experimental procedures, characterization data of **25-28**	S9-S12
Experimental procedures, characterization data of **31-40**	S12-S19
References	S19
NMR spectra of new compounds	S21-S83

Experimental procedures and characterization data of 10-23.

General procedure A for the de-*O*-isopropylidenation of 11-12 and 14-15:

The appropriate protected sugar (1 mmol) was dissolved in 70% aq AcOH (13 mL) and heated to 40 °C while stirring until TLC analysis (TLC, EtOAc or 9:1 $CHCl_3$-MeOH, 3-5 h) revealed the complete disappearance of the starting material and formation of a more retained product. The solution was then cooled to room temperature and coevaporated with toluene (4×20 mL) under diminished pressure. The crude product was purified by flash chromatography on silica gel.

General procedure B for the regioselective protection with *tert*-butyldimethylsilyl chloride (TBDMSCl) at C-6 of 16-19:

A solution of the approproate diol (**16-19**) (1 eq) in dry pyridine (7.0 mL) treated with TBDMSCl (2 eq) and the solution was left to react at room temeperature until TLC analysis (TLC, 1:1 hexane- EtOAc or EtOAc) showed complete disappearance of the starting material (2-4 h) and formation of a slower moving product. The reaction was diluted with $CHCl_3$, washed with satd aq $NaHCO_3$ and then brine. The organic phase was dried, filtered and concentrated under diminished pressure. The crude product was purified by flash chromatography on silica gel.

Methyl 2-acetamido-2-deoxy-4,6-*O*-isopropylidene-β-D-glucopyranoside (10).

A solution of known **8** [1] (5.37 g, 22.8 mmol) in dry DMF (83 mL) was cooled at 0 °C and treated with freshly distilled 2-methoxypropene (6.6 mL, 68.4 mmol, 3 eq) and camphorsulfonic acid (CSA, 530 mg, 2.28 mmol, 0.1 eq). The reaction mixture was stirred at room temperature until TLC analysis (8:2 $CHCl_3$-MeOH, 4 h) revealed the complete disappearance of the starting material. Et_3N (3 mL) was added, stirring was pursued for 30 min and concentrated under diminished pressure. Purification of the crude product by flash chromatography on silica gel (8:2 $CHCl_3$-MeOH) gave pure **10** (5.71 g, 91%) as a white foam, R_f 0.47 (8:2 $CHCl_3$-MeOH). Physic-chemical properties and NMR data were in agreement with those reported in the literature [2].

3-azidopropyl 2-acetamido-2-deoxy-4,6-*O*-isopropylidene-β-D-glucopyranoside (13).

A solution of known **9** [3] (2.96 g, 9.73 mmol) in dry DMF (33 mL) was cooled at 0 °C and treated with freshly distilled 2-methoxypropene (2.8mL, 29.2 mmol, 3 eq) and camphorsulfonic acid (CSA, 226 mg, .973 mmol, 0.1 eq). The reaction mixture was stirred at room temperature until TLC analysis (8:2 $CHCl_3$-MeOH, 2 h) revealed the complete disappearance of the starting material. Et_3N (3 mL) was added, stirring was pursued for 30 min and concentrated under diminished pressure. Purification of the crude product by flash chromatography on silica gel (EtOAc + 0.1% Et_3N) gave pure **13** (2.95 g, 88%) as a white foam, R_f 0.37 (9:1 $CHCl_3$-MeOH), $[\alpha]_D$ -46.5 (*c* 1.11 in $CHCl_3$), ^{1}H NMR (250.13 MHz, CD_3CN): δ 6.61 (d,1H, $J_{2,NH}$ 9.2 Hz, N*H*), 4.40 (d, 1H, $J_{1,2}$ 8.3 Hz, H-1), 3.84-3.68 (m, 4H, H-6a, H-6b, C*H*2O, OH-3), 3.63 (dd, 1H, $J_{2,3}$ 8.7 Hz, $J_{3,4}$ 9.5 Hz, H-3), 3.57-3.40 (m, 3H, H-2, H-4, C*H*2O), 3.34 (t, 2H, J_{vic} 6.7 Hz, CH_2N_3), 3.18 (dt, 1H, $J_{4,5}$ 9.8 Hz, $J_{5,6a}$ $J_{5,6b}$ = 5.6 Hz, H-5), 1.90 (s, 3H, *Me*CO), 1.76 (m, 2H, CH_2), 1.46, 1.34 (2s, each 3H, CMe_2); ^{13}C NMR (62.9 MHz, CD_3CN): δ 171.6 (*C*=O), 102.6 (C-1), 100.2 (CMe_2), 74.8 (C-4), 73.1 (C-3), 68.1 (C-5), 67.1 (CH_2O), 62.6 (C-6), 57.6 (C-2), 48.8 (CH_2N_3), 29.6 (CH_2), 29.4, 19.4 (CMe_2), 23.3 (*Me*CO). Anal. Found: C, 48.84; H, 7.05; N, 16.29. Cal for $C_{14}H_{24}N_4O_6$ (344.37): C, 48.83; H, 7.03; N, 16.27.

Methyl 2-acetamido-3-*O*-benzoyl-2-*deoxy*-β-D-glucopyranoside (16)

A solution of known **10** [2] (520 mg, 1.89 mmol) in dry pyridine (6 mL) was cooled to 0°C, treated with BzCl (580 μL, 5.08 mmol, 2.7 eq) and stirred at 0°C until TLC analysis (EtOAc, 2.5 h) revealed the complete disappearance of the starting material and the formation of a faster moving product UV visible (R_f 0.56). The mixture was poured into icy water, stirred for 30 min and extracted with $CHCl_3$ (3×50 mL). The combined extracts were collected, washed with 1M HCl (10 mL) then satd aq $NaHCO_3$ (10 mL), dried and concentrated under diminished pressure. Purification of the crude product by flash chromatography on silica gel (25:75 hexane-EtOAc + 0.1% Et_3N) gave pure methyl 2-acetamido-3-*O*-benzoyl-2-deoxy-4,6-*O*-isopropylidene-β-D-glucopyranoside (**11**) (631 mg, 88%) as a white foam, R_f 0.23 (25:75 hexane-EtOAc), $[\alpha]_D$ -27.5 (c 1.1 in $CHCl_3$); ^{1}H NMR (250.13 MHz, CD_3CN): δ 7.98 (m, 2H, Ar-*H*), 7.63 (m, 1H, Ar-*H*), 7.48 (m, 2H, Ar-*H*), 6.56 (d, 1H, $J_{2,NH}$ 9.7 Hz, N*H*), 5.27 (dd, 1H, $J_{2,3}$ 10.2 Hz, $J_{3,4}$ 9.4 Hz, H-3), 4.56 (d, 1H, $J_{1,2}$ 8.5 Hz, H-1), 4.01 (dd, 1H, H-2), 3.96 (dd, 1H, $J_{4,5}$ 9.7 Hz, H-4), 3.95-3.79 (m, 2H, H-6a, H-6b), 3.42 (m, 1H, H-5), 3.42 (s, 3H, OMe), 1.70 (s, 3H, *Me*CO), 1.44-1.27 (2s, each 3H, CMe_2); ^{13}C NMR (62.9 MHz, CD_3CN): δ 170.8

(CONHH), 166.8 (PhCO), 134.3-129.5 (Ar-CH), 130.8 (Ar-C), 103.3 (C-1), 100.4 (*C*Me$_2$), 74.1 (C-3), 72.7 (C-4), 67.9 (C-5) , 62.6 (C-6), 57.3 (OMe), 55.2 (C-2), 29.3, 19.4 (C*Me$_2$*) 23.0 (MeCO). Anal. Found: C, 60.18; H, 6.67; N, 3.72. Calc for $C_{19}H_{25}NO_7$ (379.41): C, 60.15; H, 6.64; N, 3.69.

Hydrolysis of **11** (202 mg, 0.533 mmol) performed according to the general procedure A. The crude product was purified by flash chromatography (EtOAc + 0.1% Et_3N) to give pure the known diol **16** (174 mg, 96%) as a white solid, R_f 0.22 (9:1 $CHCl_3$-MeOH). Physic-chemical properties and NMR data were in agreement with those reported in the literature [4].

Methyl 2-acetamido-3-*O*-benzyl-2-*deoxy*-β-D-glucopyranoside (17)

Compound **10** (6.30 g, 22.9 mmol) was dissolved in THF-H_2O (99.5:0.5, 150 mL). Powdered KOH (5.10 g, 91.2 mmol, 4 eq) and 18-crown-6 (150 mg, 0.57 mmol, 0.025 eq) were added, and the resulting mixture was vigorously stirred at room temperature for 30 min at 0°C. Benzyl bromide (5.24 ml, 45.62 mmol, 2 eq) was added, and the reacting mixture was stirred at room temperature until TLC analysis (EtOAc, 4 h) revealed the complete disappearance of the starting material and the formation of a major faster-moving product (R_f 0.41). MeOH (2 mL) was added, the mixture was stirred for an additional 10 min, solvents were evaporated under diminished pressure and the residue was portioned between CH_2Cl_2 (40 mL) and H_2O (40 mL). The aqueous phase was extracted with CH_2Cl_2 (3×40 mL) and the collected organic layers were dried, filtered and concentrated under diminished pressure. Purification of the crude product by flash chromatography on silica gel (4:6 hexane-EtOAc + 0.1% Et_3N) gave pure methyl 2-acetamido-3-*O*-benzyl-2-deoxy-4,6-*O*-isopropylidene-β-D-glucopyranoside (**12**) (6.53 g, 78%) as a white solid, R_f 0.41 (EtOAc), $[\alpha]_D$ +22.6 (*c* 1.1 in $CHCl_3$), mp 172-174 °C (from EtOAc-hexane), ^{1}H NMR (250.13 MHz, CD_3CN): δ 7.39-7.22 (m, 5H, Ar-*H*), 6.63 (d, 1H, $J_{2,NH}$ 8.2 Hz, N*H*), 4.73, 4.56 (AB system, 2H, $J_{A,B}$ 11.7 Hz, C*H$_2$*Ph), 4.35 (d, 1H, $J_{1,2}$ 8.5 Hz, H-1), 3.85 (dd, 1H, $J_{6a,6b}$ 10.2 Hz, $J_{5,6b}$ 5.6, H-6b), 3.76 (dd, 1H, $J_{5,6a}$=$J_{5,6b}$ 5.6 Hz, H-6a), 3.75 (m, 2H, H-2, H-4), 3.49 (dd, 1H, $J_{2,3}$ 8.9 Hz, $J_{3,4}$ 10 Hz, H-3), 3.37 (s, 3H, O*Me*), 3.22 (dt, 1H, $J_{4,5}$ 10.9 Hz, H-5), 1.85 (s, 3H, *Me*CON), 1.49, 1.37 (2s, each 3H, C*Me$_2$*); ^{13}C NMR (62.9 MHz, CD_3CN): δ 170.9 (C=O), 139.9 (Ar-C), 129.1, 128.6, 128.3 (Ar-CH), 103.7 (C-1), 100.1 (*C*Me$_2$), 80.0 (C-3), 75.3 (C-4), 74.2 (*C*H$_2$Ph), 67.7 (C-5), 62.7 (C-6), 57.1 (O*Me*), 55.5 (C-2), 29.5, 19.5 (C*Me$_2$*), 23.3

(*Me*CON). Anal. Found: C, 62.41; H, 7.43; N, 3.80. Calc for $C_{19}H_{27}NO_6$ (365.43): C, 62.45; H, 7.45; N, 3.83.

Hydrolysis of **12** (3.97 g, 10.86 mmol) performed according to the general procedure A. The crude product was purified by flash chromatography (EtOAc + 0.1% Et_3N) to give pure the known diol **17** (3.39 g, 96%) as a white solid, R_f 0.18 (9:1 $CHCl_3$- MeOH), mp 205-209 °C (from EtOAc), $[\alpha]_D$ -25.9 (*c* 1.1 in $CHCl_3$), Lit [5] $[\alpha]_D$ -26.6 (*c* 0.74 in CH_3OH). NMR data were in agreement with those reported in the literature [5].

3-Azidopropyl 2-acetamido-3-*O*-benzoyl-2-deoxy-β-D-glucopyranoside (18)

A solution of **13** (412 mg, 1.20 mmol) in dry pyridine (4.5 mL) was cooled to 0°C, treated with BzCl (0.43 mL, 3.71 mmol, 3.1 eq) and stirred at 0°C until TLC analysis (EtOAc, 2.5 h) revealed the complete disappearance of the starting material and the formation of a faster moving product UV visible (R_f 0.62). The mixture was poured into icy water, stirred for 15 min and extracted with $CHCl_3$ (3×10 mL). The combined extracts were collected, washed with 1M HCl (5 mL) then satd aq $NaHCO_3$ (5 mL), dried and concentrated under diminished pressure. The crude residue (520 mg, 97%) was constituted (NMR) exclusively by a 3-azidopropyl 2-acetamido-3-*O*-benzoyl-2-deoxy-4,6-*O*-isopropylidene-β-D-glucopyranoside (**14**) as a white foam, R_f 0.27 (1:1 hexane-EtOAc), ^{13}C NMR (62.9 MHz, $CDCl_3$): δ 170.4 (Me*C*O), 167.0 (Ph*C*O), 133.4, 129.7, 128.4 (Ar-*C*H), 129.0 (Ar-*C*), 102.0 (C-1), 99.6 (*C*Me_2), 73.3 (C-3), 71.7 (C-4), 66.7 (C-5), 66.0 (CH_2O), 61.8 (C-6), 53.6 (C-2), 47.7 (CH_2N_3), 28.7 (CH_2), 28.6, 18.7 (Me_2C), 23.0 (*Me*CON). Hydrolysis of crude **14** (493 mg, 1.10 mmol) performed according to the general procedure A. The crude product was purified by flash chromatography (EtOAc + 0.1% Et_3N) to give pure the diol **18** (413 mg, 89% calculated from **13**) as a white foam, R_f 0.29 (EtOAc), $[\alpha]_D$ -3.54 (*c* 1.0 in MeOH); ^1H NMR (250.13 MHz, CD_3CN): δ 8.01 (m, 2H, Ar-*H*), 7.61 (m, 1H, Ar-*H*), 7.48 (m, 2H, Ar-*H*), 6.98 (d, 1H, $J_{2,NH}$ 8.5 Hz, N*H*), 5.23 (dd, 1H, $J_{2,3}$ 9.4 Hz, H-3), 4.62 (d, 1H, $J_{1,2}$ 8.5 Hz, H-1), 4.38 (bt, 1H, OH-6), 3.96 (dd, 1H, H-2), 3.90-3.51 (m, 6H, H-4, H-6a, H-6b, OH-4, CH_2O), 3.45 (ddd, 1H, $J_{4,5}$ 10.3 Hz, $J_{5,6a}$ 2.6 Hz, $J_{5,6b}$ 6.8 Hz, H-5), 4.38 (bt, 1H, OH-6), 3.35 (t, 2H, J_{vic} 6.8 Hz, CH_2N_3), 1.79 (m, 2H, CH_2), 1.70 (s, 3H, *Me*CO); ^{13}C NMR (62.9 MHz, CD_3CN): δ 171.3 (Me*C*O), 167.2 (Ph*C*O), 134.1, 130.4, 129.4 (Ar*C*H), 130.9 (Ar-*C*), 101.6 (C-1), 77.5 (C-3), 77.0 (C-5), 69.5 (C-4), 66.9 (CH_2O), 62.3 (C-6), 54.7 (C-2),

48.8 (CH_2N_3), 29.5 (CH_2), 23.0 (*Me*CON). Anal. Found: C, 52.97; H, 5.95; N, 13.75. Calc for $C_{18}H_{24}N_4O_7$ (408.41): C, 52.94; H, 5.92; N, 13.72.

3-Azidopropyl 2-acetamido-3-*O*-benzyl-2-deoxy-β-D-glucopyranoside (19)

Compound **13** (300 mg, 0.87 mmol) was dissolved in THF-H_2O (99.5:0.5, 6.0 mL). Powdered KOH (195 mg, 3.49 mmol, 4 eq) and 18-crown-6 (5.75 mg, 0.022 mmol, 0.025 eq) were added, and the resulting mixture was vigorously stirred at room temperature for 30 min at 0°C. Benzyl bromide (0.21 ml, 1.74 mmol, 2 eq) was added, and the reacting mixture was stirred at room temperature until TLC analysis (EtOAc, 4 h) revealed the complete disappearance of the starting material and the formation of a major faster-moving product (R_f 0.57). MeOH (1 mL) was added, the mixture was stirred for an additional 10 min, solvents were evaporated under diminished pressure and the residue was portioned between CH_2Cl_2 (20 mL) and H_2O (20 mL). The aqueous phase was extracted with CH_2Cl_2 (4×20 mL) and the collected organic layers were dried, filtered and concentrated under diminished pressure. The crude residue (359 mg, 95%) was constituted (NMR) exclusively by a 3-azidopropyl 2-acetamido-3-*O*-benzyl-2-deoxy-4,6-*O*-isopropylidene-β-D-glucopyranoside (**15**) as a white solid, R_f 0.23 (1:1 hexane-EtOAc), δ ^{13}C NMR (62.9 MHz, CD_3CN): δ 170.7 (*C*=O), 140.1 (Ar-*C*), 129.1-128.3 (Ar-*C*H), 102.9 (C-1), 100.1 (*C*Me_2), 79.9 (C-3), 75.3 (C-4), 74.2 (*C*H_2Ph), 67.8 (C-5), 67.0 (*C*H_2*O*), 62.8 (C-6), 55.7 (C-2), 48.7 (*C*H_2N_3), 29.5 (*C*H_2), 29.3, 19.5 (*Me*$_2$C), 23.3 (*Me*CO).

Hydrolysis of crude **15** (359 mg, 0.826 mmol) performed according to the general procedure A. The crude product was purified by flash chromatography (EtOAc + 0.1% Et_3N) to give pure the diol **19** (313 mg, 91% calculated from **13**) as a white solid, R_f 0.15 (EtOAc), mp 156-158 °C (from EtOAc), $[\alpha]_D$ +1.68 (*c* 0.95 in MeOH), ^{1}H NMR (250.13 MHz, CD_3CN): δ 7.34-7.28 (m, 5H, Ar-*H*), 6.60 (d, 1H, $J_{2,NH}$ 9.1 Hz, N*H*), 4.79, 4.63 (AB system, 2H, $J_{A,B}$ 11.3 Hz, C*H*$_2$Ph), 4.40 (d, 1H, $J_{1,2}$ 8.4 Hz, H-1), 3.84 (dt, 1H, J_{vic} 5.8 Hz, J_{gem} 10.2 Hz, C*H*$_2$O), 3.75-3.60 (m, 4H, H-2, H-6a, H-6b, OH-4), 3.53 (dt, 1H, J_{vic} 6.3 Hz, J_{gem} 10.2 Hz, C*H*$_2$O), 3.45 (m, 2H, H-3, H-4), 3.28 (ddd, 1H, $J_{4,5}$ 9.3 Hz, $J_{5,6a}$ 3.0 Hz, $J_{5,6b}$ 5.5 Hz, H-5), 3.35 (t, 2H, J_{vic} 6.8 Hz, C*H*$_2N_3$), 3.00 (bt, 1H, OH-6), 1.84 (s, 3H, *Me*CO), 1.77 (m, 2H, C*H*$_2$); ^{13}C NMR (62.9 MHz, CD_3CN): δ 170.9 (*C*=O), 140.1 (Ar-*C*), 129.1, 128.8, 128.3 (Ar-*C*H), 102.2 (C-1), 83.4 (C-3), 77.0 (C-5), 74.9 (*C*H_2Ph), 71.9 (C-4), 66.7 (*C*H_2*O*), 62.7 (C-6), 55.5 (C-2), 48.8 (*C*H_2N_3), 29.6 (*C*H_2), 23.3 (*Me*CON). Anal.

Found: C, 54.85; H, 6.67; N, 14.24. Calc for $C_{18}H_{26}N_4O_6$ (394.43): C, 54.81; H, 6.64; N, 14.20.

Methyl 2-acetamido-3-*O*-benzoyl-6-*O*-*tert*-butyldimethylsilyl-2-deoxy-β-D-glucopyranoside (20)

Silylation of **16** (159 mg, 0.468 mmol) performed according to the general procedure B. The crude product was purified by flash chromatography (35:65 hexane-EtOAc + 0.1 % Et_3N) to give pure the acceptor **20** (172 mg, 81%) as a white solid, R_f 0.70 (9:1 $CHCl_3$-MeOH), mp 165-172 °C (from EtOAc-hexane), $[\alpha]_D$ -18.8 (*c* 1.0 in $CHCl_3$), ^{1}H NMR (250.13 MHz, CD_3CN): δ 8.00 (m, 2H, Ar-*H*), 7.61 (m, 1H, Ar-*H*), 7.49 (m, 2H, Ar-*H*), 6.58 (d, 1H, $J_{2,NH}$ 9.6 Hz, N*H*), 5.17 (dd, 1H, $J_{2,3}$ 10.6 Hz, $J_{3,4}$ 9.0 Hz, H-3), 4.48 (d, 1H, $J_{1,2}$ 8.5 Hz, H-1), 3.94 (dd, 1H, $J_{5,6b}$ 2.5 Hz, $J_{6a,6b}$ 11.3 Hz, H-6b), 3.90 (dd, 1H, $J_{5,6a}$ 4.7 Hz, H-6a), 3.89 (m, 1H, H-2), 3.82 (d, 1H, $J_{4,OH}$ 5.2 Hz, OH-4), 3.70 (ddd, 1H, $J_{4,5}$ 9.7 Hz, H- 4), 3.43 (ds, 3H, OMe), 3.42 (ddd, 1H, H-5), 1.67 (s, 3H, *Me*CO), 0.92 (s, 9H, SiC*Me*$_3$), 0.11, 0.10 (2s, each 3H, *Me*$_2$Si); ^{13}C NMR (62.9 MHz, CD_3CN): δ 170.8 (Me*C*O), 167.2 (Ph*C*O), 134.1, 130.4, 129.4 (Ar-*C*H), 131.1 (Ar-*C*), 102.5 (C-1), 77.8 (C-3), 77.1 (C-5), 69.4 (C-4), 63.5 (C-6), 56.9 (OMe), 54.6 (C-2), 26.2 (C*Me*$_2$), 23.0 (*Me*CO), 19.0 (Si*C*Me$_3$), -5.02, -5.12 (*Me*$_2$Si). Anal. Found: C, 58.28; H, 7.81; N, 3.12. Calc for $C_{22}H_{35}NO_7Si$ (453.61): C, 58.25; H, 7.78; N, 3.09.

Methyl 2-acetamido-3-*O*-benzyl-6-*O*-*tert*-butyldimethylsilyl-2-deoxy-β-D-glucopyranoside (21)

Silylation of **17** (3.38 g, 10.4 mmol) performed according to the general procedure B. The crude product was purified by flash chromatography (1:9 hexane- EtOAc + 0.1 % Et_3N) to give pure the acceptor **21** (4.24 g, 93%) as a white solid, R_f 0.52 (EtOAc), $[\alpha]_D$ +1.01 (*c* 1.0 in $CHCl_3$); ^{1}H NMR (250.13 MHz, CD_3CN): δ 7.35-7.20 (m, 5H, Ar-*H*), 6.69 (d, 1H, $J_{2,NH}$ 9.4 Hz, N*H*), 4.79, 4.62 (AB system, J_{AB} 11.3 Hz, CH_2Ph), 4.28 (d, 1H, $J_{1,2}$ 8.4 Hz, H-1), 3.91 (dd, 1H, $J_{6a,6b}$ 11.3 Hz, $J_{5,6b}$ 2.6 Hz, H-6b), 3.79-3.63 (m, 1H, H-2, H-6a, OH-4), 3.50-3.40 (m, 2H, H-3, H-4), 3.38 (s, 3H, O*Me*), 3.22 (ddd, 1H, $J_{4,5}$ 9.4 Hz, $J_{5,6a}$ 4.6 Hz, H-5), 1.83 (s, 3H, CH_3CON), 0.90 (s, 9H, C*Me*$_3$), 0.088, 0.086 (2s, each 3H, *Me*$_2$Si); ^{13}C NMR (62.9 MHz, CD_3CN): δ 171.0 (C=O), 140.2 (Ar-C), 129.1, 128.7, 128.3 (*Ar*-CH), 102.9 (C-1), 83.7 (C-3), 77.1 (C-5), 74.9 (*C*H$_2$Ph), 71.6 (C-4), 63.8 (C-6), 56.7 (O*Me*), 55.3 (C-2), 26.2 (C*Me*$_3$), 23.4 (*Me*CON), 18.9 (SiC*Me*$_3$), -5.06, -5.13

(Me_2Si). Anal. Found: C, 60.14; H, 8.45; N, 3.22. Calc for $C_{22}H_{37}NO_6Si$ (439.62): C, 60.11; H, 8.48; N, 3.19.

3-Azidopropyl 2-acetamido-3-*O*-benzoyl-6-*O*-*tert*-butyldimethylsilyl-2-deoxy-β-D-glucopyranoside (22)

Silylation of **18** (1.54 g, 3.77 mmol) performed according to the general procedure B. The crude product was purified by flash chromatography (1:1 hexane-EtOAc + 0.1 % Et_3N) to give the acceptor pure **22** (1.80 g, 91%) as a white foam, R_f 0.76 (EtOAc), $[\alpha]_D$ -11.2 (*c* 1.0 in $CHCl_3$), ^{1}H NMR (250.13 MHz, CD_3CN): δ 8.03-7.98 (m, 2H, Ar-*H*), 7.65-7.58 (m, 1H, Ar-*H*), 7.52-7.45 (m, 2H, Ar-*H*), 6.73 (d, 1H, $J_{2,NH}$ 9.6 Hz, N*H*), 5.19 (dd, 1H, $J_{2,3}$ 10.6 Hz, $J_{3,4}$ 9.0 Hz, H-3), 4.57 (d, 1H, $J_{1,2}$ 8.5 Hz, H-1), 3.98-3.78 (m, 5H, H-2, H-6a, H-6b, OH, CH_2O), 3.69 (bt, 1H, $J_{4,5}$ 9.5 Hz, H-4), 3.43 (ddd, 1H, $J_{5,6a}$ 2.4 Hz, $J_{5,6b}$ 4.3 Hz, H-5), 3.57 (dt, 1H, J_{vic} 6.2 Hz, J_{gem} 10.2 Hz, CH_2O), 3.35 (t, 2H, J_{vic} 6.8 Hz, CH_2N_3), 1.79 (m, 2H, CH_2), 1.69 (s, 3H, *Me*CO), 0.92 (s, 9H, CMe_3), 0.10, 0.09 (2s, each 3H, Me_2Si); ^{13}C NMR (62.9 MHz, CD_3CN): δ 171.0 (Me*C*O), 167.2 (Ph*C*O), 134.1 130.4,129.4 (Ar*C*H), 131.0 (Ar-*C*), 101.6 (C-1), 77.6 (C-3), 77.1 (C-5), 69.4 (C-4), 66.9 (*C*H_2O), 63.5 (C-6), 54.7 (C-2), 48.8 (*C*H_2N_3), 29.5 (*C*H_2), 26.2 (CMe_3), 23.0 (*Me*CON), 18.9 (*C*Me_3), -5.02, -5.09 (Me_2Si). Anal. Found: C, 55.19; H, 7.36; N, 10.76. Calc for $C_{24}H_{38}N_4O_7Si$ (522.67): C, 55.15; H, 7.33; N, 10.72.

3-Azidopropyl 2-acetamido-3-*O*-benzyl-6-*O*-*tert*-butyldimethylsilyl-2-deoxy-β-D-glucopyranoside (23)

Silylation of **19** (925 mg, 2.34 mmol) performed according to the general procedure B. The crude product was purified by flash chromatography (1:1 hexane- EtOAc + 0.1 % Et_3N) to give pure the acceptor **23** (1.14 g, 96%) as a white foam, R_f 0.25 (1:1 hexane-EtOAc), $[\alpha]_D$ +11.2 (*c* 1.3 in $CHCl_3$), ^{1}H NMR (250.13 MHz, CD_3CN): δ 7.34-7.24 (m, 5H, Ar-*H*), 6.73 (m, 1H, N*H*), 4.80, 4.64 (AB system, 2H, $J_{A,B}$ 11.3 Hz, CH_2Ph), 4.37 (d, 1H, $J_{1,2}$ 8.4 Hz, H-1), 3.90 (dd, 1H, $J_{6a,6b}$ 11.0 Hz, $J_{5,6b}$ 4.1 Hz, H-6b), 3.85-3.63 (m, 4H, H-2, H-6a, OH-4, CH_2O), 3.54-3.45 (m, 3H, H-3, H-4, CH_2O), 3.33 (t, 2H, J_{vic} 6.8 Hz, CH_2N_3), 3.26 (ddd, 1H, $J_{4,5}$ 9.4 Hz, $J_{5,6a}$ 5.3 Hz, H-5), 1.86 (s, 3H, *Me*CO), 1.76 (m, 2H, CH_2), 0.91 (s, 9H, SiCMe_3), 0.093 (s, 6H, Me_2Si); ^{13}C NMR (62.9 MHz, CD_3CN): δ 170.9 (*C*O), 140.0 (Ar-*C*), 129.1, 128.7, 128.3 (Ar-*C*H), 102.1 (C-1), 83.6 (C-3), 77.1 (C-5), 71.6 (C-4), 66.6 (*C*H_2O), 63.8 (C-6), 55.4 (C-2), 48.8 (*C*H_2N_3), 29.6 (*C*H_2), 26.2 (CMe_3), 23.4

(*Me*CON), 18.9 (*C*Me$_3$), -5.03, -5.09 (*Me*$_2$Si). Anal. Found: C, 56.71; H, 7.96; N, 11.05. Calc for $C_{24}H_{40}N_4O_6Si$ (508.69): C, 56.67; H, 7.93; N, 11.01.

Experimental procedures and characterization data of 25-28

4-*O*-[Benzyl 2-*O*-benzyl-3,4-*O*-isopropylidene-β-D-galactopyranosyl uronate]-2,3:5,6-di-*O*-isopropylidene-*aldehydo*-D-glucose dimethyl acetal (25)

A solution of **24** [6] (700 mg, 1.17 mmol) in acetone (117 mL) was treated with 5% aq. $NaHCO_3$ (58.45 mL), KBr (278.3, 2.34 mmol, 2 eq) and TEMPO (255.8, 1.64 mmol, 1.4 eq) and the mixture was stirred at 0°C. After 10 min 13% aq. NaOCl (4.9 mL) was added and the mixture was stirred at room temperature until the starting material was completely reacted (15 min, TLC, EtOAc). The reaction mixture was repeatedly co-evaporated with toluene (5×30 mL) under diminished pressure. A solution of the crude product in dry DMF (30.7 mL) KF (680 mg, 11.6 mmol, 10 eq) was added, after 10 min at 0°C was treated with BnBr (497 µL, 5.85 mmol, 5 eq) and the reaction was stirred overnight at room temperature. The suspension was concentrated and the residue was portioned between CH_2Cl_2 (35 mL) and H_2O (35 mL). The reaction was concentrated under diminished pressure and the residue was portioned between CH_2Cl_2 (30 mL) and satd aq NaCl (30 mL). The aqueous phase was extracted with CH_2Cl_2 (3×50 mL) and the collected organic layers were dried over $MgSO_4$, filtered and concentrated under diminished pressure. Purification of the crude product by flash chromatography on silica gel (7:3 hexane-EtOAc) gave pure **25** (763.5 mg, 93%) as a colorless syrup, R_f 0.22 (7:3 hexane-EtOAc), $[\alpha]_D$ -12.0 (*c* 1.16 in $CHCl_3$), ^1H NMR (250.13 MHz, CD_3CN): δ 7.39-7.27 (m, 10H, Ar-*H*), 5.28, 5.13 (AB system, 2H, $J_{A,B}$ 12.6 Hz, COOC*H*$_2$Ph), 4.79, 4.69 (AB system, 2H, $J_{A,B}$ 12.0 Hz, OC*H*$_2$Ph), 4.68 (d, 1H, $J_{1',2'}$ 8.1 Hz, H-1'), 4.52 (m, 2H, H-5', H-2), 4.44 (dd, 1H, $J_{3',4'}$ 5.5 Hz, $J_{4',5'}$ 2.4 Hz, H-4'), 4.35 (d, 1H, $J_{1,2}$ 6.0 Hz, H-1), 4.23 (m, 1H, H-5), 4.20 (dd, 1H, $J_{2',3'}$ 7.1 Hz, H-3'), 4.13 (dd, 1H, $J_{2,3}$ 7.0 Hz, $J_{3,4}$ 1.2 Hz, H-3), 4.05 (dd, 1H, $J_{5,6b}$ 5.9 Hz, $J_{6a,6b}$ 8.7 Hz, H-6b), 3.94 (dd, 1H, $J_{5,6a}$ 6.4 Hz, H-6a), 3.82 (dd, 1H, $J_{4,5}$ 6.1 Hz, H-4), 3.34, 3.33 (2s, each 3H, O*Me*), 3.31 (dd, 1H, H-2'), 1.39, 1.30 (2s, each 3H, C*Me*$_2$), 1.27, 1.34 (2s, each 6H, 2×C*Me*$_2$); ^{13}C NMR (62.9 MHz, CD_3CN): δ 167.7 (C-6'), 139.4, 136.9 (2×Ar-*C*), 129.4-128.4 (Ar-*C*H), 110.9, 110.6, 109.2 (3×*C*Me$_2$), 106.1 (C-1), 103.1 (C-1'), 80.6 (C-2'), 79.6 (C-3'), 78.3 (C-3), 77.8 (C-4), 77.5 (C-5), 76.4 (C-2), 75.1 (C-4'), 74.2 (O*C*H$_2$Ph), 72.5 (C-5'), 67.1 (COO*C*H$_2$Ph),

66.5 (C-6), 55.8, 54.5 (2×O*Me*), 28.0, 27.7, 27.3, 26.9, 26.4, 25.5 (3×Me_2C). Anal Found: C, 63.26; H, 7.19. Calc for $C_{37}H_{50}O_{13}$ (702.79): C, 63.23; H, 7.17.

4-*O*-[Benzyl 2-*O*-benzyl-3,4-di-*O*-acetyl-β-D-galactopyranosyl uronate]-1,2,3,6-tetra-*O*-acetyl-α,β-D-glucopyranoside (26)

A solution of protected disaccharide **25** (810.4 mg, 1.153 mmol) in 80% aq AcOH (4 mL) was stirred at 80 °C until the starting material was completely reacted (TLC, 9:1 $CHCl_3$-MeOH, 4 h) with formation of a slower moving product. The solution was concentrated under diminished pressure by co-evaporation with toluene (4×30 mL), a mixture of 1:2 Ac_2O-Py was added, the solution was stirred overnight at room temperature and was concentrated under diminished pressure by co-evaporation with toluene (4×35 mL). Flash chromatographic purification on silica gel, eluting with 6:4 hexane- EtOAc, afforded pure **26** (808 mg, 89%) as an 1:1 α/β anomeric mixture, as established on the basis of the integration of the H-1 signals (^{1}H NMR), a white foam, R_f 0.76 (6:4 hexane-EtOAc), ^{1}H NMR (250.13 MHz, CD_3CN) of **α-26**: δ 6.18 (d, 1H, $J_{1,2}$ 3.8 Hz, H-1); **β-26**: δ 5.81 (d, 1H, $J_{1,2}$ 8.3 Hz, H-1); cluster of signals for both anomers: δ 7.44-7.21 (m, 10H, Ar-*H*), 5.53 (m, 1H, H-4'), 5.40 (m, 1H, H-3), 5.12, 5.07 (AB system, 2H, $J_{A,B}$ 12.1 Hz, COOCH_2Ph), 4.92 (m, 2H, H-2, H-3'), 4.73, 4.71, 4.58, 4.56 (2×AB systems, each 2H, $J_{A,B}$ 11.2, $J_{A,B}$ 11.5 Hz, CH_2Ph), 4.52-4.45 (m, 2H, H-1', H-5'), 4.40-4.31 (m, 2H, H-6a, H-6b), 4.12-3.95 (m, 2H, H-5, H-4), 3.46 (m, 1H, H-2'), 2.18, 2.12, 2.09, 2.06, 2.02 (5s, each 3H, 5×*Me*CO), 2.00, 1.98 (2s, each 6H, 4×*Me*CO), 1.89 (m, 9H, 3×*Me*CO); ^{13}C NMR (62.9 MHz, CD_3CN): of **α-26**: δ 89.5 (C-1); **β-26**: δ 92.2 (C-1); cluster of signals for both anomers: δ 171.4-170.0 (C=O), 167.9 (C-6'), 139.0, 138.9, 136.4 (Ar-*C*), 129.5-128.7 (Ar-*C*H), 103.4, 103.2 (C-1'), 77.4, 77.3 (C-2'), 76.6, 76.4 (C-4), 75.9 (CH_2Ph), 74.4, 71.8 (C-5), 72.9 (C-5'), 72.6 (C-3'), 72.5, 69.9 (C-3), 71.0, 70.2 (C-2), 69.5 (C-4'), 67.9 (COOCH_2Ph), 62.5, 62.2 (C-6), 21.1-20.5 (*Me*CO).

4-*O*-[Benzyl 2-*O*-benzyl-3,4-di-*O*-acetyl-β-D-galactopyranosyl uronate]-2,3,6-tri-*O*-acetyl-α,β-D-glucopyranose (27)

A solution of **26** in dry DMF (804.5 mg, 1.02 mmol) was treated with NH_2NH_2·AcOH (110.6 mg, 1.23 mmol, 1.2 eq) and the mixture was stirred at 60°C until the starting material was completely disappeared (TLC, 2:8 hexane-EtOAc, 30 min). The reaction was concentrated under diminished pressure and the residue was portioned between

CH_2Cl_2 (30 mL) and satd aq NaCl (30 mL). The aqueous phase was extracted with CH_2Cl_2 (3×50 mL) and the collected organic layers were dried over $MgSO_4$, filtered and concentrated under diminished pressure. Purification of the crude product by flash chromatography on silica gel (7:3 hexane-EtOAc) gave pure **27** (602 mg, 79%) as an 7:3 α/β anomeric mixture, as established on the basis of the integration of the C1 signals (^{13}C NMR), as a white foam, R_f 0.22 (1:1 hexane-EtOAc); ^{1}H NMR (250.13 MHz, CD_3CN): of **α-27**: δ 5.42 (dd, 1H, $J_{2,3}$ 9.3 Hz, $J_{3,4}$ 10.4 Hz, H-3), 5.23 (m, 1H, H-1), 3.44 (d, 1H, $J_{2',3'}$ 10.0 Hz, H-2'), 2.08, 2.02, 1.98, 1.86, 1.85 (5s, each 3H, 5×*Me*CO); **β-27**: δ 3.42 (d, 1H, $J_{2',3'}$ 9.9 Hz, H-2'), 2.06, 2.01, 1.99, 1.97, 1.87 (5s, each 3H, 5×*Me*CO); cluster of signals for both anomers: δ 7.37-7.24 (m, 10H, Ar-*H*), 5.47 (d, 1H, $J_{4',5'}$ 1.6 Hz, $J_{3',4'}$ 3.7 Hz, H-4'), 5.16, 5.08 (AB system, 2H, $J_{A,B}$ 12.1 Hz, COOCH_2Ph), 5.00 (d, 1H, H-3'), 4.78-4.68 (m, 3H, β-H-1, β-H-3, α,β-H-2), 4.73, 4.55 (AB system, 2H, $J_{A,B}$ 11.5, CH_2Ph), 4.48 (d, 1H, H-5'), 4.46 (m, 1H, H-1'), 4.38-4.11 (m, 3H, H-5, H-6a, H-6b), 3.92-3.79 (m, 1H, H-4); ^{13}C NMR (62.9 MHz, CD_3CN): of **α-27**: δ 103.4 (C-1'), 90.4 (C-1), 77.4 (C-4), 69.2 (C-5), 72.0, (C-2), 70.0 (C-3); **β-27**: δ 103.3 (C-1'), 95.2 (C-1), 77.1 (C-4), 73.6, 73.4, 73.0 (C-2, C-3, C-4); cluster of signals for both anomers: δ 171.5-170.6 (*C*=O), 167.1 (C-6'), 139.0, 136.4 (Ar-*C*), 129.5-128.7 (Ar-*C*H), 77.3 (C-2'), 75.9 (*C*H$_2$Ph), 72.9 (C-5'), 72.6 (C-3'), 69.6 (C-4'), 67.9 (COO*C*H$_2$Ph), 62.9 (C-6), 21.1-20.6 (*Me*CO).

4-*O*-[Benzyl 2-*O*-benzyl-3,4-di-*O*-acetyl-β-D-galactopyranosyl uronate]-2,3,6-tri-*O*-acetyl-α-D-glucopyranosyl trichloroacetimidate (28)

A solution of **27** (350 mg, 0.469 mmol) in dry CH_2Cl_2 (2.20 mL) was cooled at 0 °C and a large excess of CCl_3CN (282 mL, 2.81 mmol, 6 eq) and a catalytic amount of DBU (0.054 eq) were added. The reaction mixture was stirred at room temperature until TLC analysis (1:1 hexane-EtOAc, 30 min) showed the disappearance of the starting product and then was concentrated under diminished pressure. Purification of the crude by flash chromatography on silica gel (6:4 hexane-EtOAc) gave pure the donor **28** (380 mg, 91%) as a white foam, R_f 0.25 (6:4 hexane-EtOAc), $[\alpha]_D$ +59.6 (*c* 1.14 in $CHCl_3$), ^{1}H NMR (250.13 MHz, $CDCl_3$): δ 8.63 (s, 1H, NH), 7.34-7.15 (m, 10H, Ar-*H*), 6.46 (d, 1H, $J_{1,2}$ 3.7 Hz, H-1), 5.58 (dd, 1H, $J_{4',5'}$ 1.4 Hz, $J_{3',4'}$ 3.5 Hz, H-4'), 5.60 (dd, 1H, $J_{2,3}$ 10.3 Hz, $J_{3,4}$ 9.1 Hz, H-3), 5.13, 5.04 (AB system, 2H, $J_{A,B}$ 11.9 Hz, COOCH_2Ph), 5.04 (dd, 1H, H-2), 4.92 (dd, 1H, $J_{2',3'}$ 10.2 Hz, H-3'), 4.67, 4.57 (AB system, 2H, $J_{A,B}$ 11.6, CH_2Ph),

4.40 (dd, 1H, $J_{6a,6b}$ 12.3 Hz, $J_{5,6b}$ 1.9 Hz, H-6b), 4.39 (dd, 1H, $J_{1',2'}$ 7.6 Hz, H-1'), 4.19 (d, 1H, H-5'), 4.13 (dd, 1H, H-6a), 4.07 (ddd, 1H, $J_{4,5}$ 10.1 Hz, $J_{5,6a}$ 3.7 Hz, H-5), 3.54 (dd, 1H, H-2'), 2.14, 1.99, 1.98, 1.85, 1.82 (5s, each 3H, 5×*Me*CO); ^{13}C NMR (62.9 MHz, $CDCl_3$): δ 170.7, 170.0, 169.9, 169.8, 169.5 (5×*C*=O), 165.4 (C-6'), 160.7 (*C*=NH), 137.7, 134.7 (2×Ar-*C*), 128.9-127-5 (Ar-*C*H), 102.5 (C-1'), 92.9 (C-1), 90.7 (*C*Cl_3), 76.4 (C-2'), 75.3 (*C*H_2Ph), 75.1 (C-4), 72.1 (C-5'), 71.9 (C-3'), 71.3 (C-5), 69.8, (C-2), 68.9 (C-3), 68.4 (C-4'), 67.3 (COO*C*H_2Ph, 61.0 (C-6), 20.8-20.2 (5×*Me*CO). Anal. Found: C, 51.25; H, 4.78; N, 1.59. Calc for $C_{38}H_{42}Cl_3NO_{17}$ (891.10): C, 51.22; H, 4.75; N, 1.57.

Experimental procedures and characterization data of 31-40

General procedure A for the 4-*O*-glycosylation. A mixture of the appropriate acceptors **20-23** (1.0 eq), excess of opportune donors **39,30** (1.5 eq) and activated AW-300 MS (800 mg) in dry CH_2Cl_2 (17 mL), was stirred for 30 min at room temperature. The suspension was then cooled to -30 °C and a solution of TMSOTf (0.5 eq) in dry CH_2Cl_2 was added. The reaction mixture was allowed to slowly attain room temperature with stirring until the appropriate acceptor was disappeared (17-24 h, TLC) and the formation of a higher major UV visible spot. Et_3N (0.5 mL) was added and after 30 min the mixture was filtered through a short pad of Celite, diluted with CH_2Cl_2 and concentrated under diminished pressure. Purification of crude product by flash chromatography on silica gel afforded pure the disaccharide **31-35**.

Methyl 4-*O*-(2,3,4,6-tetra-*O*-acetyl-β-D-galactopyranosyl)-2-acetamido-3-*O*-benzoyl-6-*O*-*t*-butyldimethylsilyl-2-*deoxy*-β-D-glucopyranoside (31).

A solution of acceptor **20** (161 mg, 0.355 mmol, 1 eq) and donor **29** (262 mg, 0.532 mmol, 1.5 eq) in dry CH_2Cl_2 (5.5 mL) was treatment with TMSOTf (32 μL, 0.177 mmol, 0.5 eq) in dry CH_2Cl_2 (0.5 mL) in accordance with the general procedure A. The reaction was stirred until the acceptor was disappeared (12 h, TLC,). Purification of the crude product by flash chromatography on silica gel (3:7 hexane-EtOAc + 0.1% Et_3N) gave pure disaccharide **31** (173 mg, 62%) as a white foam, R_f 0.23 (3:7 hexane-EtOAc), $[\alpha]_D$ -11.95 (*c* 0.94 in $CHCl_3$), ^{1}H NMR (250.13 MHz, CD_3CN-$CDCl_3$): δ 8.01-7.98 (m, 2H, Ar-*H*), 7.61-7.54 (m, 1H, Ar-*H*), 7.49-7.41 (m, 2H, Ar-*H*), 6.48 (d, 1H, $J_{2,NH}$ 9.5 Hz, N*H*), 5.24 (dd, 1H, $J_{2,3}$ 10.5 Hz, $J_{3,4}$ 9.2 Hz, H-3), 5.11 (dd, 1H, $J_{3',4'}$ 3.1 Hz, $J_{4',5'}$ 1.1 Hz, H-4'), 4.86 (dd, 1H, $J_{2',3'}$ 10.3 Hz, H-3'), 4.82 (dd, 1H, $J_{1',2'}$ 7.3 Hz, H-2'), 4.67 (d,

1H, H-1'), 4.47 (d, 1H, $J_{1,2}$ 8.5, H-1), 4.10-3.98 (m, 2H, H-4, H-6b), 3.95-3.83 (m, 2H, H-2, H-6a), 3.69 (dt, 1H, $J_{5',6'a}$=$J_{5',6'b}$ 6.5 Hz, H-5'), 3.52 (dd, 1H, $J_{6'a,6'b}$ 11.2 Hz, H-6'b), 3.44 (m, 1H, H-5), 3.42 (s, 3H, OMe), 3.37 (dd, 1H, H-6'a), 2.01, 1.94, 1.86, 1.86 (4s, each 3H, 4×*Me*COO), 1.72 (s, 3H, *Me*CON), 0.94 (s, 9H, *Me*$_3$C), 0.13, 0.12 (2s, each 3H, *Me*$_2$Si); ^{13}C NMR (62.9 MHz, CD_3CN-$CDCl_3$): δ 170.7-170.2 (*C*=O), 166.5 (Ph*C*O), 133.9, 130.3, 129.2 (Ar-*C*H), 131.2 (Ar-*C*), 102.3 (C-1), 100.7 (C-1'), 75.8 (C-5), 75.5 (C-4), 74.4 (C-3), 71.5 (C-3'), 71.3 (C-5'), 69.9 (C-2'), 67.8 (C-4'), 61.9 (C-6), 61.5 (C-6'), 56.8 (O*Me*), 54.5 (C-2), 26.2 (*Me*$_3$C), 23.3 (*Me*CON), 21.1-20.7 (4×*Me*COO), 18.8 (Me$_3$*C*), -4.86, -5.09 (*Me*$_2$Si). Anal. Found: C, 55.18; H, 6.86; N, 1.82. Calc for $C_{36}H_{53}NO_{16}Si$ (783.90): C, 55.16; H, 6.82; N, 1.79.

Methyl 4-*O*-(2,3,4,6-tetra-*O*-acetyl-β-D-galactopyranosyl)-2-acetamido-3-*O*-benzyl-6-*O*-*t*-butyldimethylsilyl-2-*deoxy*-β-D-glucopyranoside (32).

A solution of acceptor **21** (773 mg, 1.76 mmol, 1 eq) and donor **29** (1.26 g, 2.56 mmol, 1.5 eq) in dry CH_2Cl_2 (28 mL) was treatment with AW-300 MS (1.45 g) and TMSOTf (154 μL, 0.852 mmol, 0.5 eq) in dry CH_2Cl_2 (1.0 mL) in accordance with the general procedure A. The reaction was stirred until the acceptor was disappeared (12 h, TLC, 3:7 hexane-EtOAc). Purification of the crude product by flash chromatography on silica gel (3:7 hexane-EtOAc) gave pure disaccharide **32** (542 mg, 40%) as a white foam, R_f 0.38 (3:7 esano-EtOAc), $[\alpha]_D$ -8.75 (*c* 1.12 in $CHCl_3$), ^{1}H NMR (250.13 MHz, CD_3CN): δ 7.38-7.26 (m, 5H, Ar-*H*), 6.55 (d, 1H, $J_{2,NH}$ 9.3 Hz, N*H*), 5.32 (dd, 1H, $J_{4',5'}$ 0.8 Hz, $J_{3',4'}$ 3.3 Hz, H-4'), 5.09 (dd, 1H, $J_{1',2'}$ 7.5 Hz, $J_{2',3'}$ 10.4 Hz, H-2'), 5.01 (dd, 1H, H-3'), 4.86, 4.57 (AB system, 2H, $J_{A,B}$ 10.7 Hz, C*H*$_2$Ph), 4.85 (d, 1H, H-1'), 4.28 (d, 1H, $J_{1,2}$ 8.2 Hz, H-1), 4.03-3.85 (m, 6H, H-5', H-6'a, H-6'b, H-4, H-6a, H-6b), 3.69 (ddd, 1H, $J_{2,3}$ 8.7 Hz, H-2), 3.50 (dd, 1H, $J_{3,4}$ 10.1 Hz, H-3), 3.37 (s, 3H, O*Me*), 2.09, 2.04, 1.93, 1.91 (4s, each 3H, 4×*Me*COO), 1.84 (s, 3H, *Me*CON), 0.93 (s, 9H, *Me*$_3$C), 0.12 (s, 6H, *Me*$_2$Si); ^{13}C NMR (62.9 MHz, CD_3CN): δ 171.2-170.5 (5×C=O), 140.1 (Ar-*C*), 128.9-128.3 (Ar-*C*H), 102.8 (C-1), 100.7 (C-1'), 80.8 (C-3), 76.3 (C-4), 76.2 (C-5), 73.9 (CH_2Ph), 71.7 (C-5'), 71.7 (C-3'), 70.3 (C-2'), 68.3 (C-4'), 62.8 (C-6), 62.3 (C-6'), 56.6 (O*Me*), 26.3 (*Me*$_3$C), 23.3 (*Me*CON), 21.0-20.1 (4×*Me*COO), 18.9 (Me$_3$*C*), -4.80, -5.07 (Me$_2$Si). Anal. Found: C, 56.14; H, 7.18; N, 1.80. Calc for $C_{36}H_{55}NO_{15}Si$ (769.91): C, 56.16; H, 7.20; N, 1.82.

3-Azidopropyl 4-*O*-(2,3,4,6-tetra-*O*-acetyl-β-D-galactopyranosyl)-2-acetamido-3-*O*-benzoyl-6-*O*-*t*-butyldimethylsilyl-2-*deoxy*-β-D-glucopyranoside (33).

A solution of acceptor **22** (215 mg, 0.41 mmol, 1 eq) and donor **29** (306 mg, 0.62 mmol, 1.5 eq) in dry CH_2Cl_2 (5.5 mL) was treatment with AW-300 MS (350 mg) and TMSOTf (38 μL, 0.21 mmol, 0.5 eq) in dry CH_2Cl_2 (0.5 mL) in accordance with the general procedure A. The reaction was stirred until the acceptor was disappeared (16 h, TLC, 1:1 hexane-EtOAc). Purification of the crude product by chromatography on silica (first 65:35 hexane-EtOAc + 0.1% Et_3N then 1:1 hexane-EtOAc + 0.1% Et_3N) gave pure disaccharide **33** (165 mg, 47%) as a white foam, R_f 0.27 (1:1 toluene-EtOAc), $[\alpha]_D$ -3.28 (*c* 0.97 in $CHCl_3$), ^1H NMR (250.13 MHz, CD_3CN): δ 8.02-7.95 (m, 2H, Ar-*H*), 7.62-7.56 (m, 1H, Ar-*H*), 7.50-7.42 (m, 2H, Ar-*H*), 6.52 (d, 1H, $J_{2,NH}$ 9.3 Hz, N*H*), 5.25 (dd, 1H, $J_{2,3}$ 10.5 Hz, $J_{3,4}$ 9.6 Hz, H-3), 5.11 (dd, 1H, $J_{3',4'}$ 3.1 Hz, $J_{4',5'}$ 1.0 Hz, H-4'), 4.90 (dd, 1H, $J_{2',3'}$ 10.3 Hz, H-3'), 4.82 (dd, 1H, $J_{1',2'}$ 7.5 Hz, H-2'), 4.69 (d, 1H, H-1'), 4.57 (d, 1H, $J_{1,2}$ 8.4 Hz, H-1), 4.00 (t, 1H, $J_{4,5}$ 9.6 Hz, H-4), 3.92-3.79 (m, 4H, H-2, H-6a, H-6b, H-6'b), 3.90 (m, 1H, CH_2O), 3.71 (dt, 1H, $J_{5',6'a}$=$J_{5',6'b}$ 6.6 Hz, H-5'), 3.59-3.43 (m, 2H, H-5, H-6'a), 3.52 (dt, 1H, J_{vic} 6.7 Hz, J_{gem} 11.0 Hz, CH_2O), 3.35 (t, 2H, J_{vic} 6.7 Hz, CH_2N_3), 2.00, 1.93, 1.86, 1.85 (4s, each 3H, 4×*Me*COO), 1.79 (m, 2H, CH_2), 1.72 (s, 3H, *Me*CON), 0.94 (s, 9H, Me_3C), 0.14, 0.12 (2s, each 3H, Me_2Si); ^{13}CNMR (62.9 MHz, CD_3CN): δ 171.0-170.2 (5×*C*=O), 166.6 (Ph*C*O), 133.9, 130.4, 129.3 (Ar-*C*H), 131.3 (Ar-*C*), 101.5 (C-1), 100.8 (C-1'), 76.0 (C-5), 75.5 (C-4), 74.4 (C-3), 71.5 (C-3'), 71.4 (C-5'), 70.0 (C-2'), 67.9 (C-4'), 66.8 (*C*H_2O), 62.0 (C-6), 61.6 (C-6'), 54.7 (C-2), 48.8 (*C*H_2N_3), 29.5 (*C*H_2), 26.2 (Me_3C), 23.0 (*MeC*ON), 20.9-20.5 (4×*Me*COO), 18.8 (Me_3*C*), -4.86, -5.07 (Me_2Si). Anal. Found: C, 53.54; H, 6.65; N, 6.60. Calc for $C_{38}H_{56}N_4O_{16}Si$ (852.96): C, 53.51; H, 6.62; N, 6.57.

3-Azidopropyl 4-*O*-(2,3,4,6-tetra-*O*-acetyl-β-D-galactopyranosyl)-2-acetamido-3-*O*-benzyl-6-*O*-*t*-butyldimethylsilyl-2-*deoxy*-β-D-glucopyranoside (34).

A solution of acceptor **23** (412 mg, 0.81 mmol, 1 eq) and donor **29** (599 mg, 1.22 mmol, 1.5 eq) in dry CH_2Cl_2 (12.5 mL) was treatment with AW-300 MS (621 mg) and TMSOTf (73 μL, 0.405 mmol, 0.5 eq) in accordance with the general procedure A. The reaction was stirred until the acceptor was disappeared (4 h, TLC, 7:3 CH_2Cl_2-EtOAc). Purification of the crude product by chromatography on silica (1:1 hexane-EtOAc +

0.1% Et_3N) gave pure disaccharide **34** (489 mg, 72%) as a white foam, R_f 0.16 (1:1 toluene-EtOAc), $[\alpha]_D$ -10.63 (*c* 1.05 in $CHCl_3$), ^{1}H NMR (250.13 MHz, CD_3CN): δ_H 7.38-7.24 (m, 5H, Ar-*H*), 6.51 (d, 1H, $J_{2,NH}$ 9.2 Hz, N*H*), 5.32 (dd, 1H, $J_{3',4'}$ 3.3 Hz, $J_{4',5'}$ 0.9 Hz, H-4'), 4.09 (dd, 1H, $J_{1',2'}$ 7.5 Hz, $J_{2',3'}$ 10.4 Hz, H-2'), 5.00 (dd, 1H, H-3'), 4.86-4.56 (AB system, 2H, $J_{A,B}$ 10.7 Hz, CH_2Ph), 4.85 (d, 1H, H-1'), 4.37 (d, 1H, $J_{1,2}$ 8.2 Hz, H-1), 4.04 (dd, 1H, $J_{5',6'b}$ 4.8 Hz, $J_{6'a,6'b}$ 12.5 Hz, H-6'b), 3.98 (dd, 1H, $J_{3,4}$ 10.2 Hz, $J_{4,5}$ 9.4 Hz, H-4), 3.94-3.83 (m, 4H, H-6a, H-6b, H-5', H-6'a), 3.80 (dt, 1H, J_{vic} 5.8 Hz, J_{gem} 10.2 Hz, CH_2O), 3.67 (ddd, 1H, $J_{2,3}$ 10.1 Hz, H-2), 3.52 (dt, 1H, J_{vic} 6.2 Hz, J_{gem} 10.2 Hz, CH_2O), 3.50 (dd, 1H, H-3), 3.34 (t, 2H, J_{vic} 6.8 Hz, CH_2N_3), 3.28 (dt, 1H, $J_{5,6a}$=$J_{5,6'b}$ 2.2 Hz, H-5), 1.95, 1.94, 1.92, 1.91 (4s, each 3H, 4×*Me*COO), 1.85 (s, 3H, *Me*CON), 1.79 (m, 2H, CH_2), 0.93(s, 9H, Me_3C), 0.12 (s, each 3H, Me_2Si); ^{13}C NMR (62.9 MHz, CD_3CN): δ 171.1, 170.9, 170.7, 170.6, 170.4 (5×*C*=O), 140.1 (Ar-*C*), 128.9, 128.2 (Ar-*C*H), 102.0 (C-1), 100.7 (C-1'), 80.6 (C-3), 76.3 (C-5), 76.2 (C-4), 73.8 (*C*H_2Ph), 71.7 (C-3'), 71.6 (C-5'), 70.3 (C-2'), 68.3 (C-4'), 66.6 (*C*H_2O), 62.3 (C-6'), 62.2 (C-6), 55.0 (C-2), 48.8 (*C*H_2N_3), 29.6 (*C*H_2), 26.3 (Me_3C), 23.3 (*MeC*ON), 21.0-20.8 (4×*Me*COO), 18.9 (Me_3*C*), -4.82, -5.08 (Me_2Si). Anal. Found: C, 54.44; H, 7.01; N, 6.72. Calc for $C_{38}H_{56}N_4O_{15}Si$ (838.98): C, 54.40; H, 6.97; N, 6.68.

Methyl 4-*O*-(2,3,4-tetra-*O*-acetyl-6-azido-6-*deoxy*-β-D-galactopyranosyl)-2-acetamido-3-*O*-benzyl-6-*O*-*t*-butyldimethyl-silyl-2-*deoxy*-β-D-glucopyranoside (35).

A solution of acceptor **21** (193 mg, 0.44 mmol, 1 eq) and donor **30** (315 mg, 0.66 mmol, 1.5 eq) in dry CH_2Cl_2 (7.5 mL) was treatment with AW-300 MS (380 mg) and TMSOTf (40 μL, 0.22 mmol, 0.5 eq) in dry CH_2Cl_2 (0.5 mL) in accordance with the general procedure A. The reaction was stirred until the acceptor was disappeared (12 h, TLC, 4:6 CH_2Cl_2-EtOAc). Purification of the crude product by chromatography on silica (7:3 AcOEt-CH_2Cl_2) gave pure disaccharide **35** (189 mg, 57%) as a white foam, R_f 0.39 (6:4 CH_2Cl_2-EtOAc), $[\alpha]_D$ -26.4 (*c* 1.0 in $CHCl_3$), ^{1}H NMR (250.13 MHz, CD_3CN): δ 7.37-7.31 (m, 5H, Ar-*H*), 6.70 (d, 1H, $J_{2,NH}$ 9.3 Hz, N*H*), 5.29 (dd, 1H, $J_{3',4'}$ 3.3 Hz, $J_{4',5'}$ 1.1 Hz, H-4'), 5.09 (dd, 1H, $J_{2',3'}$ 10.4 Hz, $J_{1',2'}$ 7.6 Hz, H-4'), 5.01 (dd, 1H, H-4'), 4.91, 4.58 (AB system, 2H, $J_{A,B}$ 10.9 Hz, CH_2Ph), 4.86 (d, 1H, H-1'), 4.29 (d, 1H, $J_{1,2}$ 8.1 Hz, H-1), 3.95 (dd, 1H, $J_{3,4}$ 10.0 Hz, $J_{4,5}$ 8.6 Hz, H-4), 3.64 (ddd, $J_{2,3}$ 8.4 Hz, 1H, H-2), 3.92-3.84 (m, 3H, H-5, H-6a, H-6b), 3.83 (m, 1H, H-5'), 3.48 (dd, 1H, H-3), 3.37 (s, 3H, O*Me*), 3.30 (dd, 1H, $J_{6'a,6'b}$ 12.3 Hz, $J_{5',6'b}$ 5.2 Hz, H-6'b), 3.09 (dd, 1H, $J_{5',6'a}$ 4.7 Hz, H-6'a), 2.09,

2.04, 1.92, (3s, each 3H, 3×*Me*COO), 1.85 (s, 3H, *Me*CON), 0.94 (s, 9H, Me_3C), 0.12, 0.11 (2s, each 3H, Me_2Si); ^{13}C NMR (62.9 MHz, CD_3CN): δ 171.2-170.5 (4×C=O), 140.1 (Ar-*C*), 128.9-128.2 (Ar-CH), 102.8 (C-1), 100.5 (C-1'), 80.8 (C-3), 76.2 (C-4), 76.1 (C-5), 74.6 (CH_2Ph), 73.1 (C-5'), 71.6 (C-3'), 70.3 (C-2'), 68.8 (C-4'), 62.1 (C-6), 56.6 (O*Me*), 55.1 (C-2), 50.9 (C-6'), 26.2 (Me_3C), 23.3 (*Me*CON), 21.0-20.7 (3×*Me*COO), 18.8 (Me_3C), -4.87, -5.12 (Me_2Si). Anal. Found: C, 54.27; H, 6.99; N, 7.49. Calc for $C_{34}H_{54}N_4O_{13}Si$ (752.89): C, 54.24; H, 6.96; N, 7.44.

General procedure B for the preparation of lactosamine acceptor 36-40.

A solution of appropriate protected disaccharide **31-35** (1 mmol) in 70% aq AcOH (58 mL) was stirred at 70 °C until the TLC analysis revealed the complete reaction of the starting material with formation of slower moving products. The solution was then cooled to room temperature and repeatedly co-evaporated with toluene (4×30 mL) under diminished pressure. Purification of the crude residue by flash chromatography on silica gel affording the lactosamine acceptors pure **36-40**.

Methyl 4-*O*-(2,3,4,6-tetra-*O*-acetyl-β-D-galactopyranosyl)-2-acetamido-3-*O*-benzoyl-2-*deoxy*-β-D-glucopyranoside (36).

Selective hydrolysis of **31** (220 mg, 0.28 mmol) was performed according to the general procedure B. The reaction was stopped after 1 h (TLC, EtOAc) and the crude product was purified by flash chromatography (95:5 CH_3Cl-MeOH) to give acceptor pure **36** (156 mg, 83%) as a white foam, R_f 0.23 (95:5 $CHCl_3$-MeOH), $[\alpha]_D$ -8.13 (*c* 1.0 in $CHCl_3$), ^{1}H NMR (250.13 MHz, CD_3CN-$CDCl_3$): δ 8.03-7.96 (m, 2H, Ar-*H*), 7.61-7.54 (m, 1H, Ar-*H*), 7.48-7.40 (m, 2H, Ar-*H*), 6.64 (d,1H, $J_{2,NH}$ 9.5 Hz, N*H*), 5.26 (dd, 1H, $J_{2,3}$ 10.6 Hz, $J_{3,4}$ 9.1 Hz, H-3), 5.10 (dd, 1H, $J_{3',4'}$ 3.3 Hz, $J_{4',5'}$ 1.2 Hz, H-4'), 4.93 (dd, 1H, $J_{2',3'}$ 10.3 Hz, H-3'), 4.85 (dd, 1H, $J_{1',2'}$ 7.6 Hz, H-2'), 4.66 (d, 1H, H-1'), 4.51 (d, 1H, $J_{1,2}$ 8.5 Hz, H-1), 4.02-3.90 (m, 2H, H-4, H-2), 3.82 (dd, 1H, $J_{6'a,6'b}$ 12.1 Hz, $J_{5',6'b}$ 2.0 Hz, H-6'b), 3.71-3.63 (m, 2H, H-6'a, H-6b), 3.51-3.40 (m, 3H, H-5', H-5, OH-6), 3.44 (s, 3H, OMe), 3.31 (dd, 1H, $J_{6a,6b}$ 11.0 Hz, $J_{5,6a}$ 6.0 Hz, H-6a), 2.02, 1.91, 1.88, 1.86 (4s, each 3H, *Me*COO), 1.72 (s, 3H, *Me*CON); ^{13}C NMR (62.9 MHz, CD_3CN-$CDCl_3$): δ 170.8-170.2 (5×*Me*CO), 166.4 (Ph*C*O), 133.8, 130.2, 129.1 (Ar-*C*H), 130.9 (Ar-*C*), 102.2 (C-1), 101.1 (C-1'), 76.2 (C-4), 75.7 (C-5), 74.6 (C-3), 71.3 (C-3'), 70.9 (C- 5'), 69.8 (C-2'), 67.5 (C-4'), 61.0 (C-6), 60.7 (C-6'), 56.0 (*Me*O), 54.5 (C-2), 23.1 (*MeC*ON), 20.9, 20.7,

20.6, 20.5 (4×*Me*COO). Anal. Found: C, 53.83; H, 5.89; N, 2.12. Calc for $C_{30}H_{39}NO_{16}$ (669.63): C, 53.81; H, 5.87; N, 2.09.

Methyl 4-O-(2,3,4,6-tetra-*O*-acetyl-β-D-galactopyranosyl)-2-acetamido-3-*O*-benzyl-2-*deoxy*-β-D-glucopyranoside (37).

Selective hydrolysis of **32** (525 mg, 0.68 mmoli) was performed according to the general procedure B. The reaction was stopped after 2 h (TLC, EtOAc) and the crude product was purified by flash chromatography (95:5 CH_3Cl-MeOH) to give acceptor pure **37** (371 mg, 83%) as a white foam, R_f 0.22 (3:7 hexane-EtOAc), $[\alpha]_D$ -6.13 (*c* 1.06 in $CHCl_3$), ^{1}H NMR (250.13 MHz, CD_3CN): δ 7.37-7.21 (m, 5H, Ar-*H*), 6.59 (d, 1H, $J_{2,NH}$ 9.3 Hz, N*H*), 5.30 (dd, 1H, $J_{3',4'}$ 3.1 Hz, $J_{4',5'}$ 0.9 Hz, H-4'), 5.13-5.01 (m, 2H, H-2', H-3'), 4.86, 4.56 (AB system, 2H, $J_{A,B}$ 10.8 Hz, CH_2Ph), 4.80 (d, 1H, $J_{1',2'}$ 7.9 Hz, H-1'), 4.32 (d, 1H, $J_{1,2}$ 8.2 Hz, H-1), 4.02-3.83 (m, 4H, H-5', H-6'a, H-6'b, H-4), 3.81-3.62 (m, 3H, H-2, H-6a, H-6b), 3.53 (dd, 1H, $J_{2,3}$ 8.7 Hz, $J_{3,4}$ 10.1 Hz, H-3), 3.40 (s, 3H, OMe), 3.30 (ddd, 1H, $J_{4,5}$ 9.5 Hz, $J_{5,6a}$ 2.3 Hz, $J_{5,6a}$ 4.3 Hz, H-5), 3.10 (bt, 1H, OH-6), 2.09, 2.04, 1.91, 1.89 (4s, each 3H, 4×*Me*COO), 1.84 (s, 3H, *Me*CON); ^{13}C NMR (62.9 MHz, CD_3CN): δ 171.0, 170.9, 170.8, 170.7, 170.4 (5×C=O), 139.9 (Ar-*C*), 128.9-128.1 (Ar-*C*H), 102.8 (C-1), 101.0 (C-1'), 80.9 (C-3), 77.1 (C-4), 76.2 (C-5), 73.9 (*C*H_2Ph), 71.6 (C-3'), 71.3 (C-5'), 70.3 (C-2'), 68.1 (C-4'), 61.9 (C-6'), 61.1 (C-6), 56.8 (O*Me*), 54.9 (C-2), 23.3 (*Me*CON), 20.9-20.8 (4×*Me*COO). Anal. Found: C, 54.99; H, 6.34; N, 2.18. Calc for: $C_{30}H_{41}NO_{15}$ (655.65): C, 54.96; H, 6.30; N, 2.14.

3-Azidopropyl 4-*O*-(2,3,4,6-tetra-*O*-acetyl-β-D-galactopyranosyl)-2-acetamido-3-*O*-benzoyl-2-*deoxy*-β-D-glucopyranoside (38).

Selective hydrolysis of **33** (290 mg, 0.34 mmol) was performed according to the general procedure B. The reaction was stopped after 2 h (TLC, EtOAc) and the crude product was purified by flash chromatography (1:9 hexane-EtOAc) to give acceptor pure **38** (242 mg, 96%) as a white foam, R_f 0.34 (EtOAc), $[\alpha]_D$ -15.58 (*c* 1.2 in $CHCl_3$), ^{1}H NMR (250.13 MHz, CD_3CN): δ 8.04-8.00 (m, 2H, Ar-*H*), 7.62-7.54 (m, 1H, Ar-*H*), 7.51-7.42 (m, 2H, Ar-*H*), 6.58 (d,1H, $J_{2,NH}$ 9.5 Hz, N*H*), 5.27 (dd, 1H, $J_{2,3}$ 10.6 Hz, $J_{3,4}$ 9.2 Hz, H-3), 5.10 (dd, 1H, $J_{3',4'}$ 3.4 Hz, $J_{4',5'}$ 1.2 Hz, H-4'), 4.95 (dd, 1H, $J_{2',3'}$ 10.4 Hz, H-3'), 4.84 (dd, 1H, $J_{1',2'}$ 7.7 Hz, H-2'), 4.64 (d, 1H, H-1'), 4.60 (d, 1H, $J_{1,2}$ 8.5 Hz, H-1), 3.96 (dd, 1H, $J_{4,5}$ 9.8 Hz, H-4), 3.89 (m, 1H, H-2), 3.84 (m, 1H, CH_2O), 3.78 (m, 1H, H-6'b), 3.69

(m, 1H, H-6'a), 3.69 (m, 1H, H-5'), 3.57 (dt, 1H, J_{vic} 6.3 Hz, J_{gem} 10.3 Hz, C*H2*O), 3.46 (ddd, 1H, $J_{6a,6b}$ 11.1 Hz, $J_{5,6b}$ 7.4 Hz, H-6b), 3.44 (m, 1H, H-5), 3.28 (m, 1H, H-6a), 3.36 (t, 2H, J_{vic} 6.8 Hz, CH_2N_3), 3.10 (bt, 1H, $J_{OH,6a}$=$J_{OH,6b}$ 5.0 Hz, OH-6), 2.02, 1.91, 1.89, 1.86 (4s, each 3H, 4×*Me*COO), 1.78 (m, 2H, CH_2), 1.71 (s, 3H, *Me*CON); ^{13}C NMR (62.9 MHz, CD_3CN): δ 171.0-170.5 (5×*C*=O), 166.6 (Ph*C*O), 134.1, 130.4, 129.4 (Ar-*C*H), 131.2 (Ar-*C*), 101.5 (C-1), 101.3 (C-1'), 76.5 (C-4), 76.0 (C-5), 74.7 (C-3), 71.5 (C-3'), 71.1 (C-5'), 70.0 (C-2'), 67.8 (C-4'), 67.0 (*C*H_2O), 61.2 (C-6), 60.9 (C-6'), 54.8 (C-2), 48.8 (*C*H_2N_3), 29.5 (*C*H_2), 23.0 (*Me*CON), 20.9-20.6 (4×*Me*COO). Anal. Found: C, 52.06; H, 5.76; N, 7.62. Calc for $C_{32}H_{42}N_4O_{16}$ (738.70): C, 52.03; H, 5.73; N, 7.58.

3-Azidopropyl 4-*O*-(2,3,4,6-tetra-*O*-acetyl-β-D-galactopyranosyl)-2-acetamido-3-*O*-benzyl-2-*deoxy*-β-D-glucopyranosyde (39).

Selective hydrolysis of **34** (135 mg, 0.161 mmol) was performed according to the general procedure B. The reaction was stopped after 1.5 h (TLC, EtOAc) and the crude product was purified by flash chromatography (1:9 hexane-EtOAc) to give acceptor pure **39** (94 mg, 81%) as a white foam, R_f 0.28 (EtOAc), $[\alpha]_D$ -9.9 (*c* 1.10 in $CHCl_3$), ^{1}H NMR (250.13 MHz, CD_3CN): δ 7.36-7.25 (m, 5H, Ar-*H*), 6.60 (d,1H, $J_{2,NH}$ 9.3 Hz, N*H*), 5.30 (dd, 1H, $J_{3',4'}$ 3.1 Hz, $J_{4',5'}$ 0.9 Hz, H-4'), 5.12-5.03 (m, 2H, H-2', H-3'), 4.86, 4.57 (AB system, 2H, $J_{A,B}$ 10.8 Hz, *CH2*Ph), 4.81 (d, 1H, $J_{1',2'}$ 7.9 Hz, H-1'), 4.40 (d, 1H, $J_{1,2}$ 8.3 Hz, H-1), 4.01 (M, 1H, C*H2*O), 3.98 (m, 1H, H-5'), 3.91-380 (m, 3H, H-4, H-6'a, H-6'b), 3.79-3.65 (m, 2H, H-6a, H-6b), 3.71 (m, 1H, $J_{2,3}$ 8.7 Hz, H-2), 3.53 (dd, 1H, $J_{3,4}$ 10.2 Hz, H-3), 3.52 (dt, 1H, J_{vic} 6.4 Hz, J_{gem} 10.2 Hz, C*H2*O), 3.34 (t, 2H, J_{vic} 6.7 Hz, CH_2N_3), 3.30 (ddd, 1H, $J_{4,5}$ 9.5 Hz, $J_{5,6a}$ 2.3 Hz, $J_{5,6b}$ 4.4 Hz, H-5), 2.09, 2.04, 1.91, 1.89 (4s, each 3H, 4×*Me*COO), 1.84 (s, 3H, *Me*CON), 1.77 (m, 2H, CH_2); ^{13}C NMR (62.9 MHz, CD_3CN): δ 171.1-170.5 (5×*C*=O), 140.O (Ar-*C*), 128.9-128.2 (Ar-*C*H), 102.0 (C-1), 101.1 (C-1'), 80.9 (C-3), 77.2 (C-4), 76.5 (C-5), 74.0 (*C*H_2Ph), 71.6 (C-3'), 71.4 (C-5'), 70.3 (C-2'), 68.2 (C-4'), 66.8 (*C*H_2O), 62.0 (C-6'), 61.2 (C-6), 55.1 (C-2), 48.8 (*C*H_2N_3), 29.6 (*C*H_2), 23.3 (*Me*CON), 21.0-20.8 (4×*Me*CO). Anal. Found: C, 52.06; H, 6.15; N, 7.76. Calc for $C_{32}H_{44}N_4O_{15}$ (724.28): C, 52.03; H, 6.12; N, 7.73.

Methyl 4-*O*-(2,3,4-tetra-*O*-acetyl-6-azido-6-*deoxy*-β-D-galactopyranosyl)-2-acetamido-3-*O*-benzyl-2-*deoxy*-β-D-glucopyranoside (40).

Selective hydrolysis of **35** (158 mg, 0.21 mmol) was performed according to the general procedure B. The reaction was stopped after 1 h (TLC, EtOAc) and the crude product was purified by flash chromatography (1:9 hexane-EtOAc) to give acceptor pure **40** (123 mg, 92%) as a white foam, R_f 0.27 (95:5 EtOAc-MeOH), $[\alpha]_D$ -14.5 (*c* 1.0 in $CHCl_3$), ^1H NMR (250.13 MHz, CD_3CN): δ 7.36-7.30 (m, 5H, Ar-*H*), 6.73 (d, 1H, $J_{2,NH}$ 9.3 Hz, NH), 5.28 (dd, 1H, $J_{3',4'}$ 3.0 Hz, $J_{4',5'}$ 1.1 Hz, H-4'), 5.12-5.02 (m, 2H, H-2', H-3'), 4.90, 4.57 (AB system, 2H, $J_{A,B}$ 10.9 Hz, C*H2*Ph), 4.81 (d, 1H, $J_{1',2'}$ 7.9 Hz, H-1'), 4.30 (d, 1H, $J_{1,2}$ 8.2 Hz, H-1), 3.86 (dd, 1H, $J_{3,4}$ 10.1 Hz, $J_{4,5}$ 8.9 Hz, H-4), 3.84 (m, 1H, H-5'), 3.80 (dd, 1H, $J_{6a,6b}$ 12.2 Hz, $J_{5,6b}$ 2.2 Hz, H-6b), 3.67 (dd, 1H, $J_{5,6a}$ 8.8 Hz, H-6a), 3.65 (m, 1H, H-2), 3.50 (dd, 1H, $J_{2,3}$ 8.6 Hz, H-3), 3.31 (ddd, 1H, H-5), 3.28 (dd, 1H, $J_{6'a,6'b}$ 12.8 Hz, $J_{5',6'b}$ 7.4 Hz, H-6'b), 3.12 (dd, 1H, $J_{5',6'a}$ 5.5 Hz, H-6'a), 3.39 (s, 3H, O*Me*), 2.10, 2.04, 1.92 (3s, each 3H, 3×*Me*COO), 1.84 (s, 3H, *Me*CON); ^{13}C NMR (62.9 MHz, CD_3CN): δ 171.3, 171.2, 170.9, 170.7 (4×C=O), 140.1 (Ar-*C*), 129.0-128.2 (Ar-CH), 102.9 (C-1), 100.8 (C-1'), 81.1 (C-3), 77.1 (C-4), 76.3 (C-5), 74.5 (CH_2Ph), 72.6 (C-3'), 72.5 (C-5'), 70.4 (C-2'), 68.8 (C-4'), 61.1 (C-6), 56.9 (O*Me*), 55.2 (C-2), 50.8 (C-6'), 23.3 (*Me*CON), 21.0-20.8 (3×*Me*COO). Anal. Found: C, 52.75; H, 6.08; N, 8.82. Calc for $C_{28}H_{38}N_4O_{13}$ (638.63): C, 52.71; H, 6.05; N, 8.79.

References

[1] Gudmundsdottir, A.V.; Nitz, M. Protecting Group Free Glycosidations Using p-Toluenesulfonohydrazide Donors. *Org. Lett.* **2008**, *10*, 3461-3463. doi: 10.1021/ol801232f.

[2] Mulard, L.A.; Costachel C.; Sansonetti P.J. Synthesis of the Methyl Glycosides of a Di- and Two Trisaccharide Fragments Specific for the *Shigella flexneri* Serotype 2a *O*-Antigen. *J. Carbohydr. Chem.* **2000**, *19*, 849-877. doi: 10.1080/07328300008544123.

[3] Willems, M.M.J.H.P.; Zom, G.G.; Meeuwenoord, N.; Ossendorp, F.A.; Overkleeft, H.S.; van der Marel, G.A.; Codée J.D.C.; Filippov, D.V. Design, automated synthesis and immunological evaluation of NOD2-ligand-antigen conjugates. *Beilstein J. Org. Chem.* **2014**, *10*, 1445-1453. doi: 10.3762/bjoc.10.148.

[4] Umezawa, S.; Tsuchiya, T.; Tatsuta, K. Studies of Aminosugars. XI. Configurational Studies of Aminosugar Glycosides and Aminocyclitols by a Copper Complex Method. *Bull. Chem. Soc. Jap.* **1966**, *39*, 1235-1243. doi: 10.1246/bcsj.39.1235

[5] Cai, Y.; Ling C.-C.; Bundle, D.R. Concise and Efficient Synthesis of 2-Acetamido-2-deoxy-β-D-hexopyranosides of Diverse Aminosugars from 2-Acetamido-2-deoxy-β-D-glucose. *J. Org. Chem.* **2009**, *74*, 580-589. doi:10.1021/jo801927k

[6] Yoshino, T.; Reuter, G.; Kelm S.; Schauer, R. Facile synthesis of 2'-substituted lactoses. *Glycoconjugate J.,***1986**, *3*, 7-14. doi: 10.1007/BF01108607.

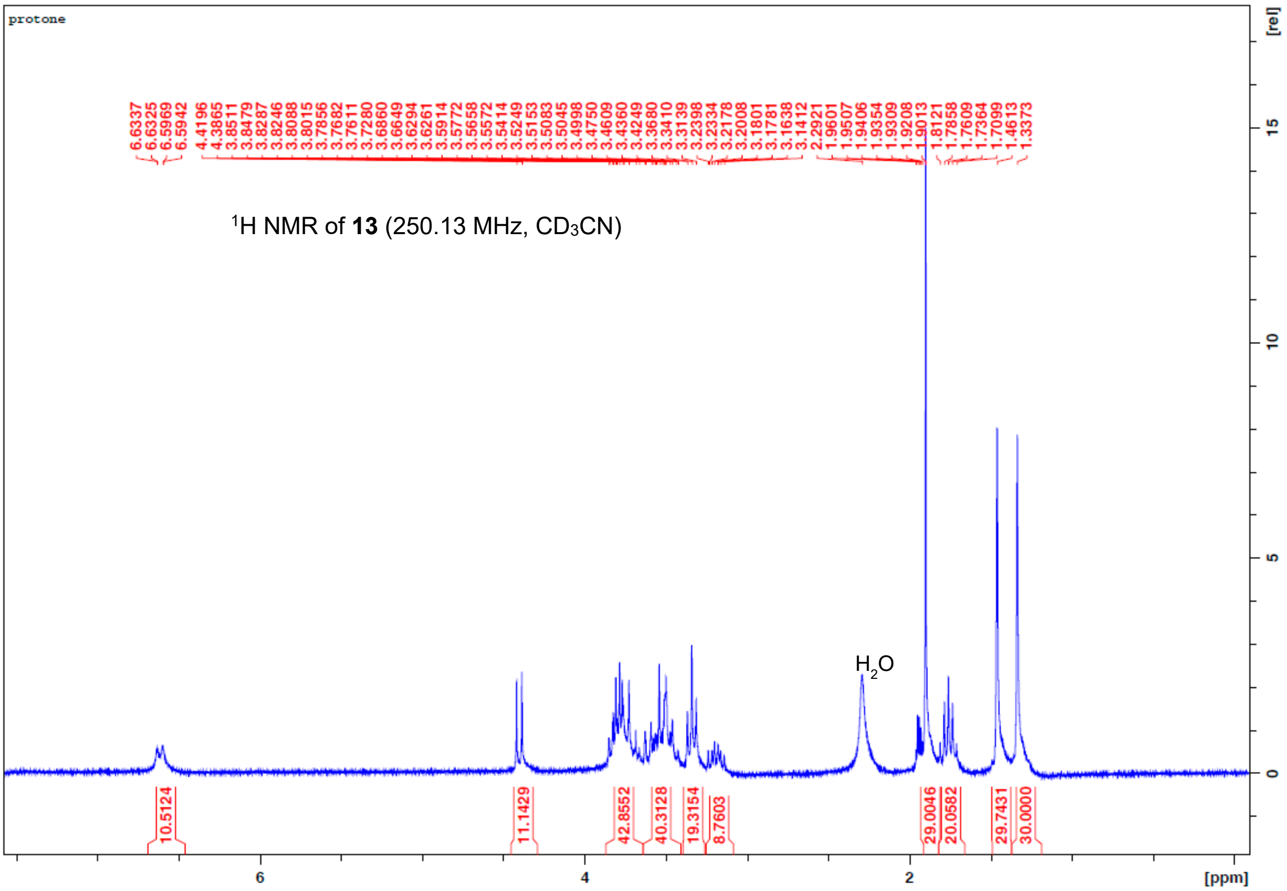
protone
^{1}H NMR of **13** (250.13 MHz, CD_3CN)
6.6337 6.6325 6.5969 6.5942 4.4196 4.3865 3.8511 3.8479 3.8287 3.8246 3.8088 3.8015 3.7856 3.7682 3.7611 3.7280 3.6860 3.6649 3.6294 3.6261 3.5914 3.5772 3.5658 3.5572 3.5414 3.5249 3.5153 3.5083 3.5045 3.4998 3.4750 3.4609 3.4360 3.4249 3.3680 3.3410 3.3139 3.2398 3.2334 3.2178 3.2008 3.1801 3.1781 3.1638 3.1412 2.2921 1.9601 1.9507 1.9406 1.9354 1.9309 1.9208 1.9013 1.8121 1.7858 1.7609 1.7364 1.7099 1.4613 1.3373
H_2O
10.5124 11.1429 42.8552 40.3128 19.3154 8.7603 29.0046 20.0582 29.7431 30.0000
6 4 2 [ppm]
[rel] 0 5 10 15

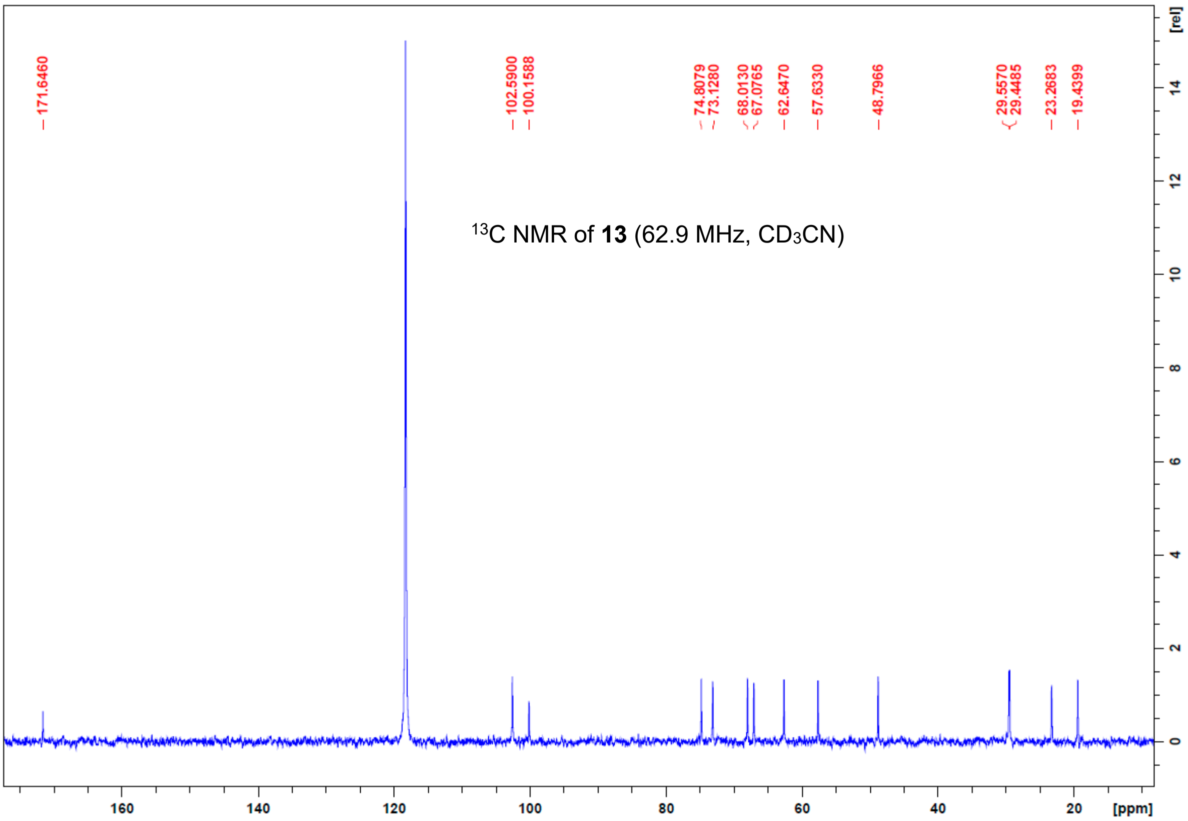
^{13}C NMR of **13** (62.9 MHz, CD_3CN)
171.6460 102.5900 100.1588 74.8079 73.1280 68.0130 67.0765 62.6470 57.6330 48.7966 29.5570 29.4485 23.2683 19.4399
160 140 120 100 80 60 40 20 [ppm]
[rel] 0 2 4 6 8 10 12 14

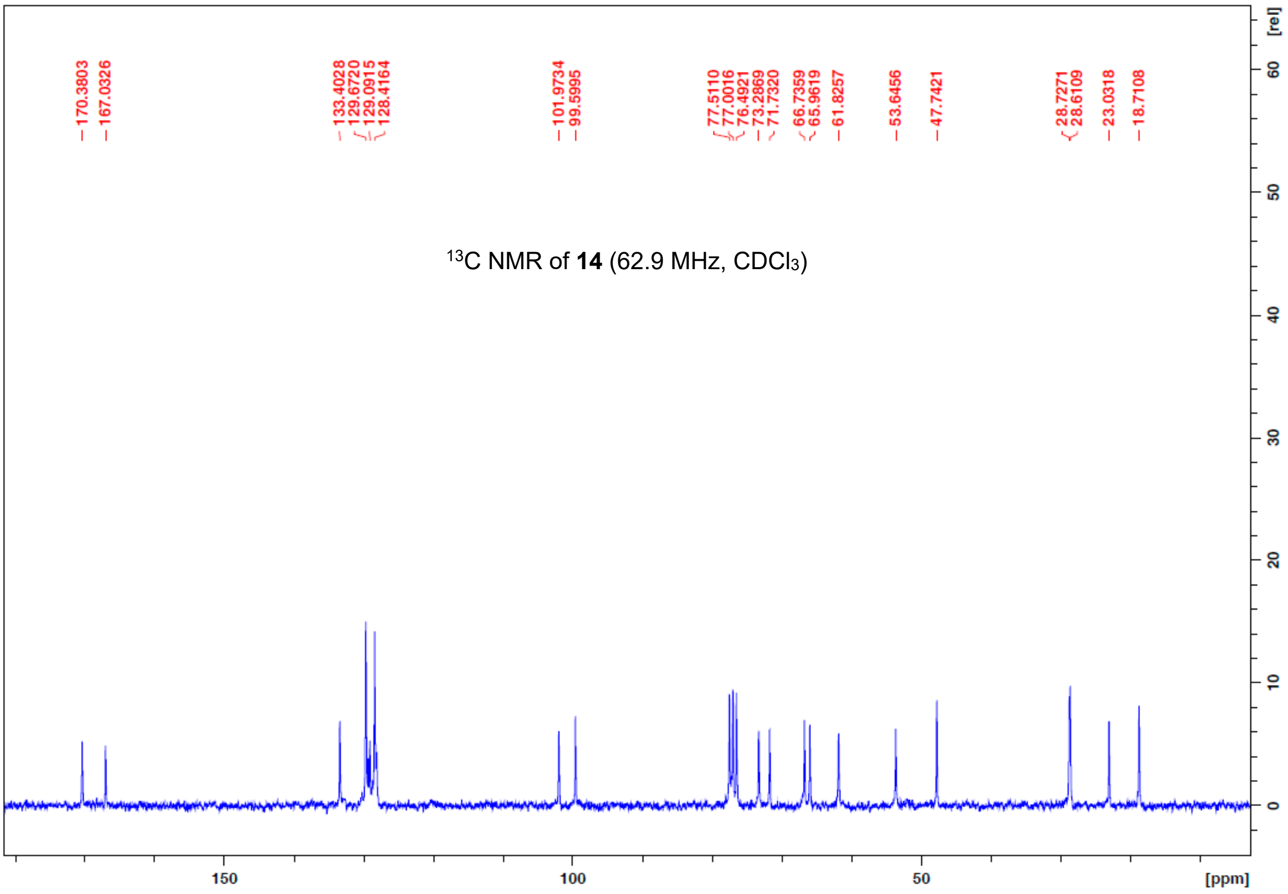
170.3803
167.0326
133.4028
129.6720
129.0915
128.4164
101.9734
99.5995
77.5110
77.0016
76.4921
73.2869
71.7320
66.7359
65.9619
61.8257
53.6456
47.7421
28.7271
28.6109
23.0318
18.7108
13C NMR of 14 (62.9 MHz, CDCl3)
[rel]
[ppm]

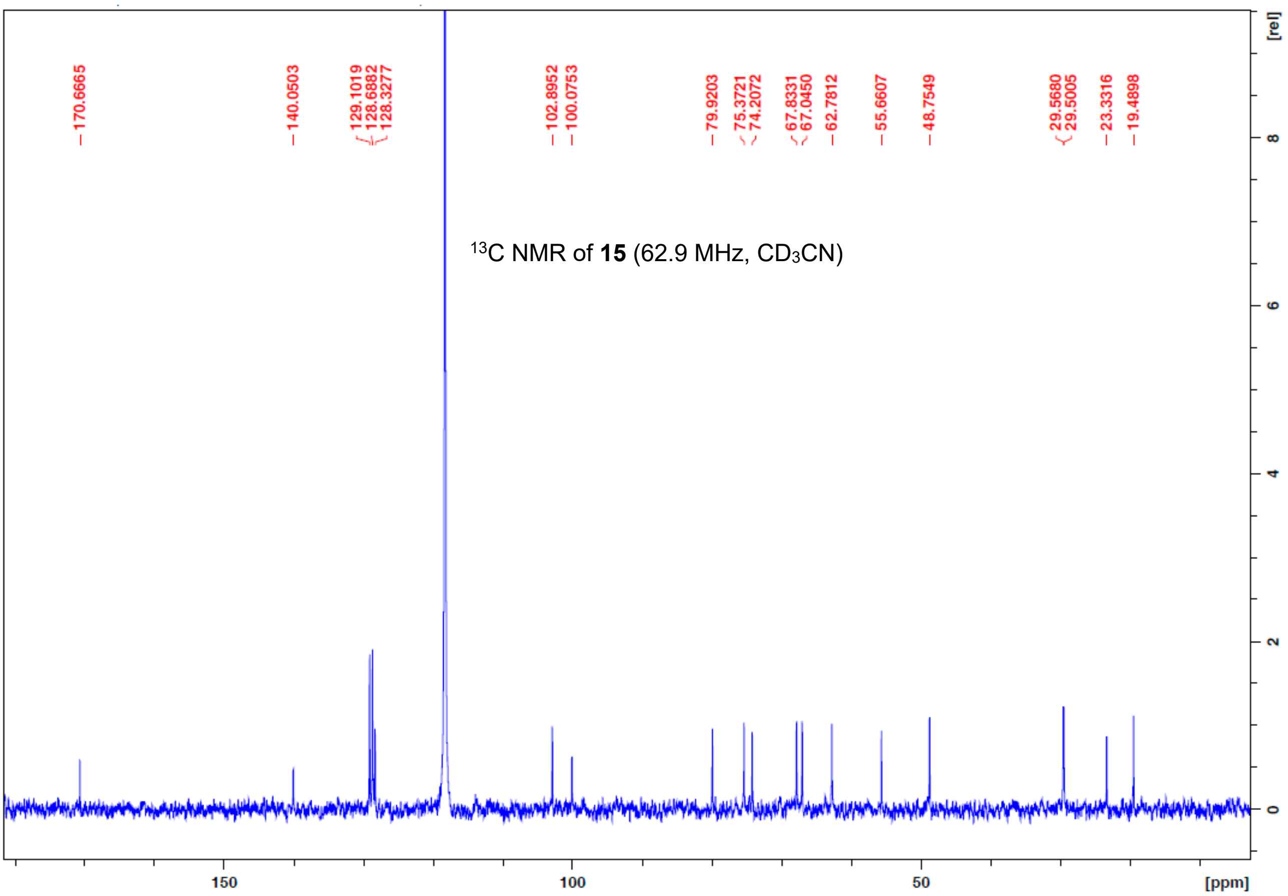
170.6665
140.0503
129.1019
128.6882
128.3277
102.8952
100.0753
79.9203
75.3721
74.2072
67.8331
67.0450
62.7812
55.6607
48.7549
29.5680
29.5005
23.3316
19.4898
13C NMR of 15 (62.9 MHz, CD3CN)
[rel]
[ppm]

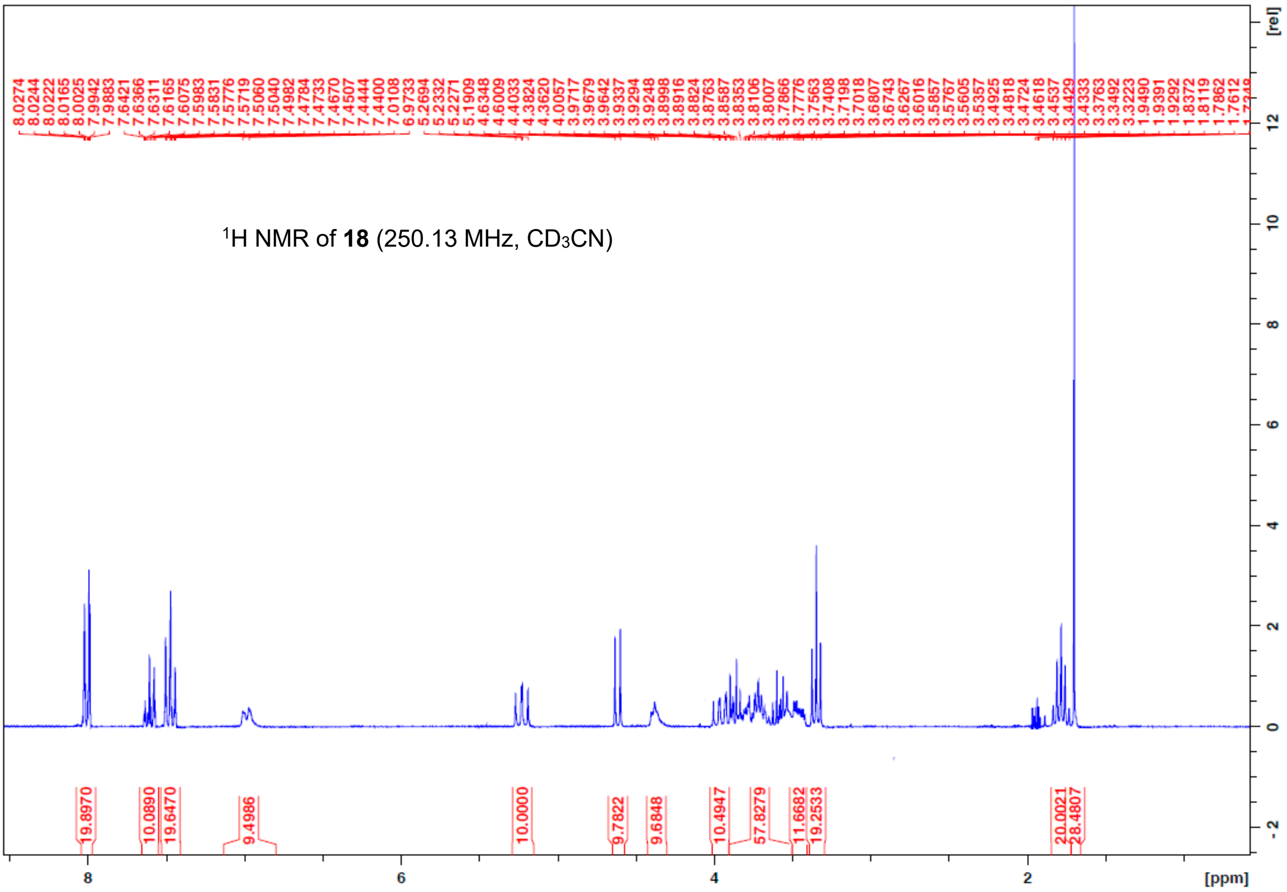
8.0274
8.0244
8.0222
8.0165
8.0025
7.9942
7.9883
7.6421
7.6366
7.6311
7.6165
7.6075
7.5983
7.5831
7.5776
7.5719
7.5060
7.5040
7.4982
7.4784
7.4733
7.4670
7.4507
7.4444
7.4400
7.0108
6.9733
5.2694
5.2332
5.2271
5.1909
4.6348
4.6009
4.4033
4.3824
4.3620
4.0057
3.9717
3.9679
3.9642
3.9337
3.9294
3.9248
3.8998
3.8916
3.8824
3.8763
3.8587
3.8353
3.8106
3.8007
3.7866
3.7776
3.7563
3.7408
3.7198
3.7018
3.6807
3.6743
3.6267
3.6016
3.5857
3.5767
3.5605
3.5357
3.4925
3.4818
3.4724
3.4618
3.4537
3.4429
3.4333
3.3763
3.3492
3.3223
1.9490
1.9391
1.9292
1.8372
1.8119
1.7862
1.7612
[illegible]
[rel]
^{1}H NMR of **18** (250.13 MHz, CD_3CN)
19.8970
10.0890
19.6470
9.4986
10.0000
9.7822
9.6848
10.4947
57.8279
11.6682
19.2533
20.0021
28.4807
[ppm]

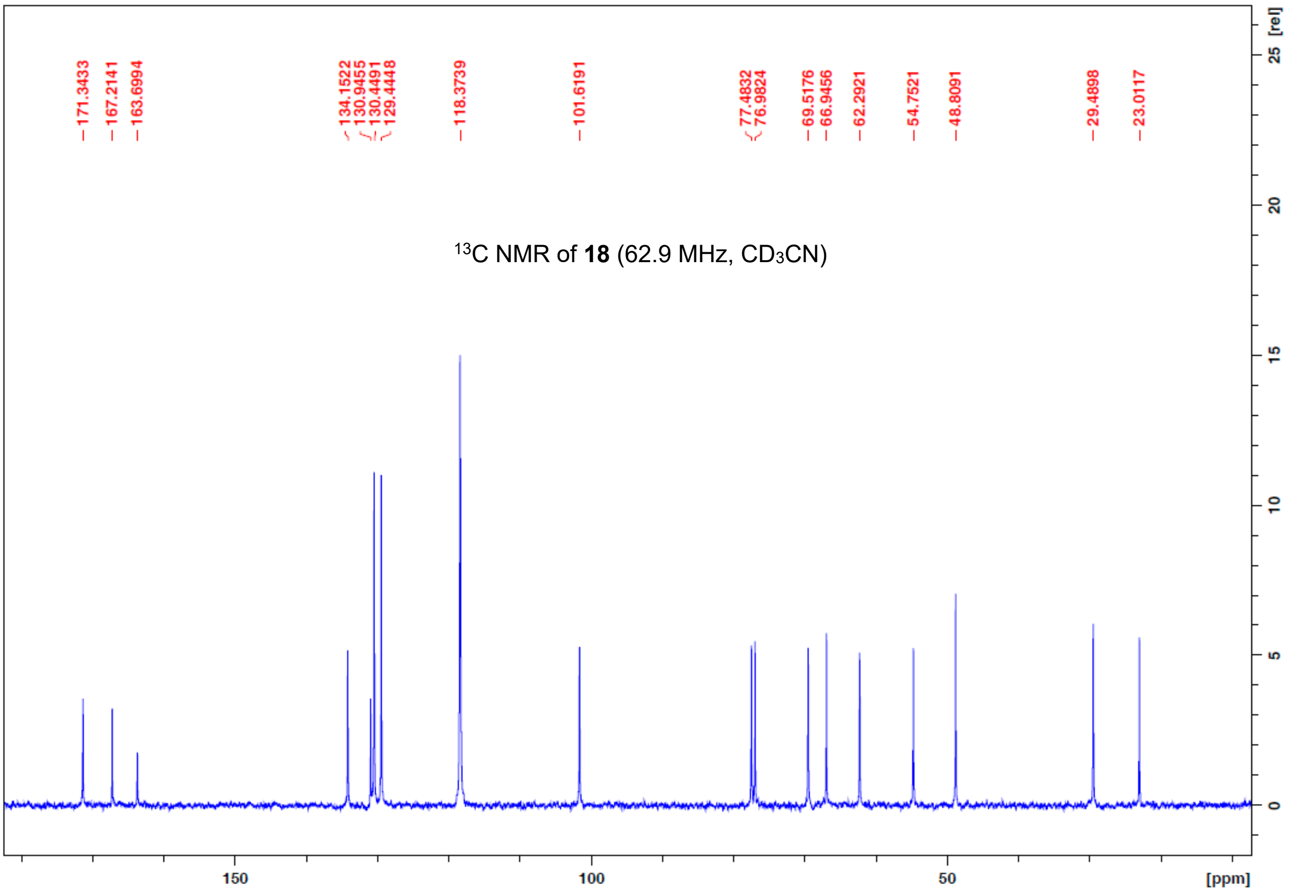
171.3433
167.2141
163.6994
134.1522
130.9455
130.4491
129.4448
118.3739
101.6191
77.4832
76.9824
69.5176
66.9456
62.2921
54.7521
48.8091
29.4898
23.0117
[rel]
^{13}C NMR of **18** (62.9 MHz, CD_3CN)
[ppm]

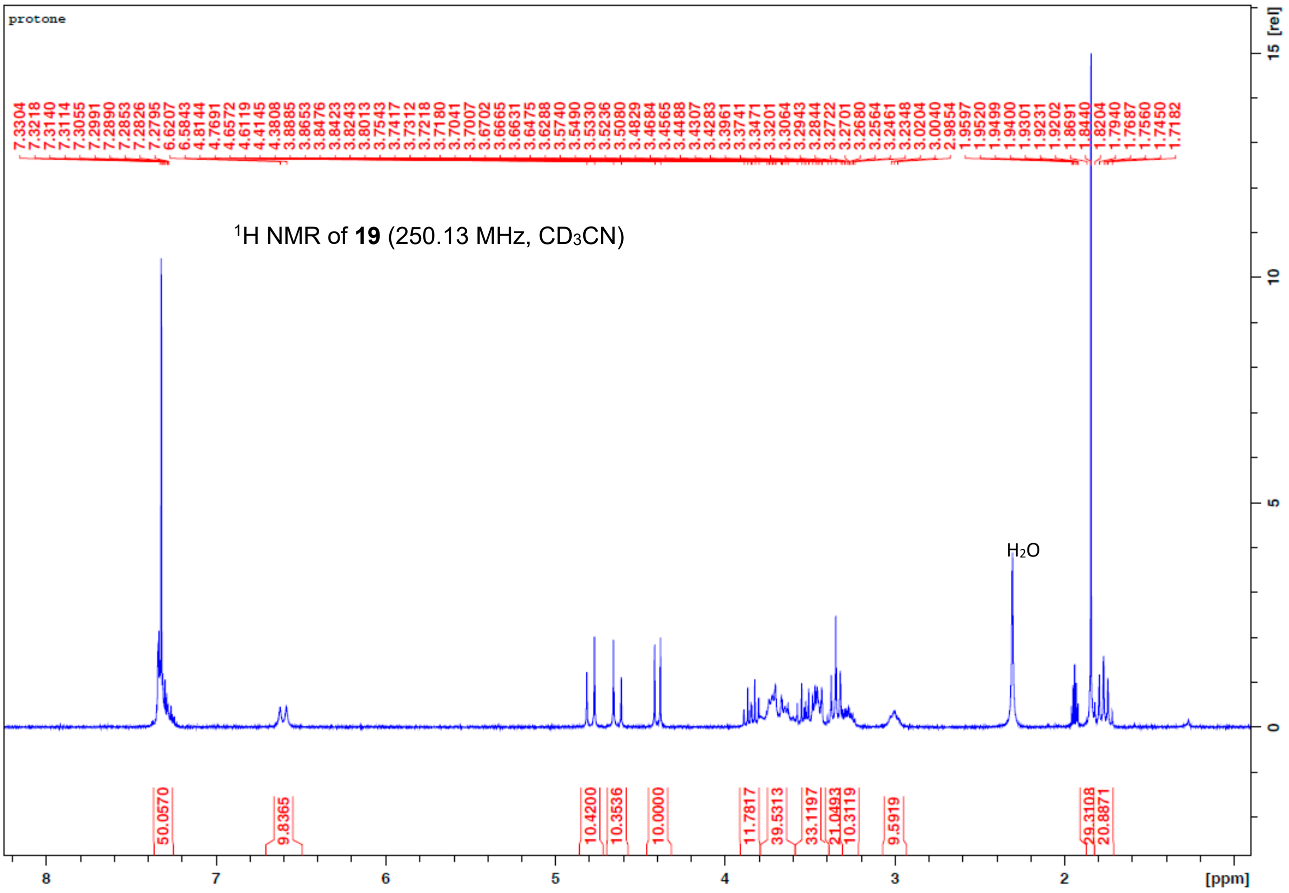
protone
^{1}H NMR of **19** (250.13 MHz, CD_3CN)
H_2O
[ppm]
[rel]

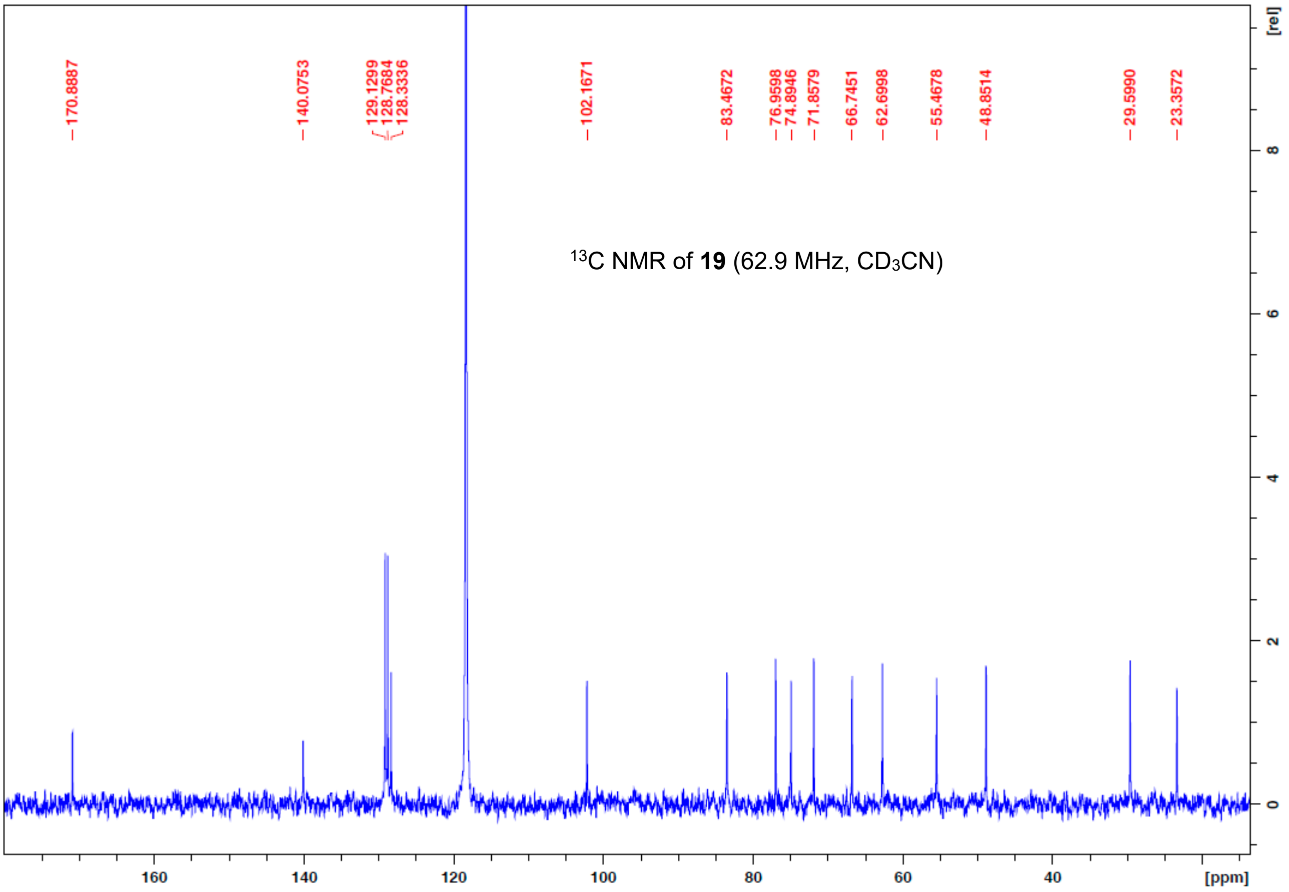
^{13}C NMR of **19** (62.9 MHz, CD_3CN)
170.8887
140.0753
129.1299
128.7684
128.3336
102.1671
83.4672
76.9598
74.8946
71.8579
66.7451
62.6998
55.4678
48.8514
29.5990
23.3572
[ppm]
[rel]

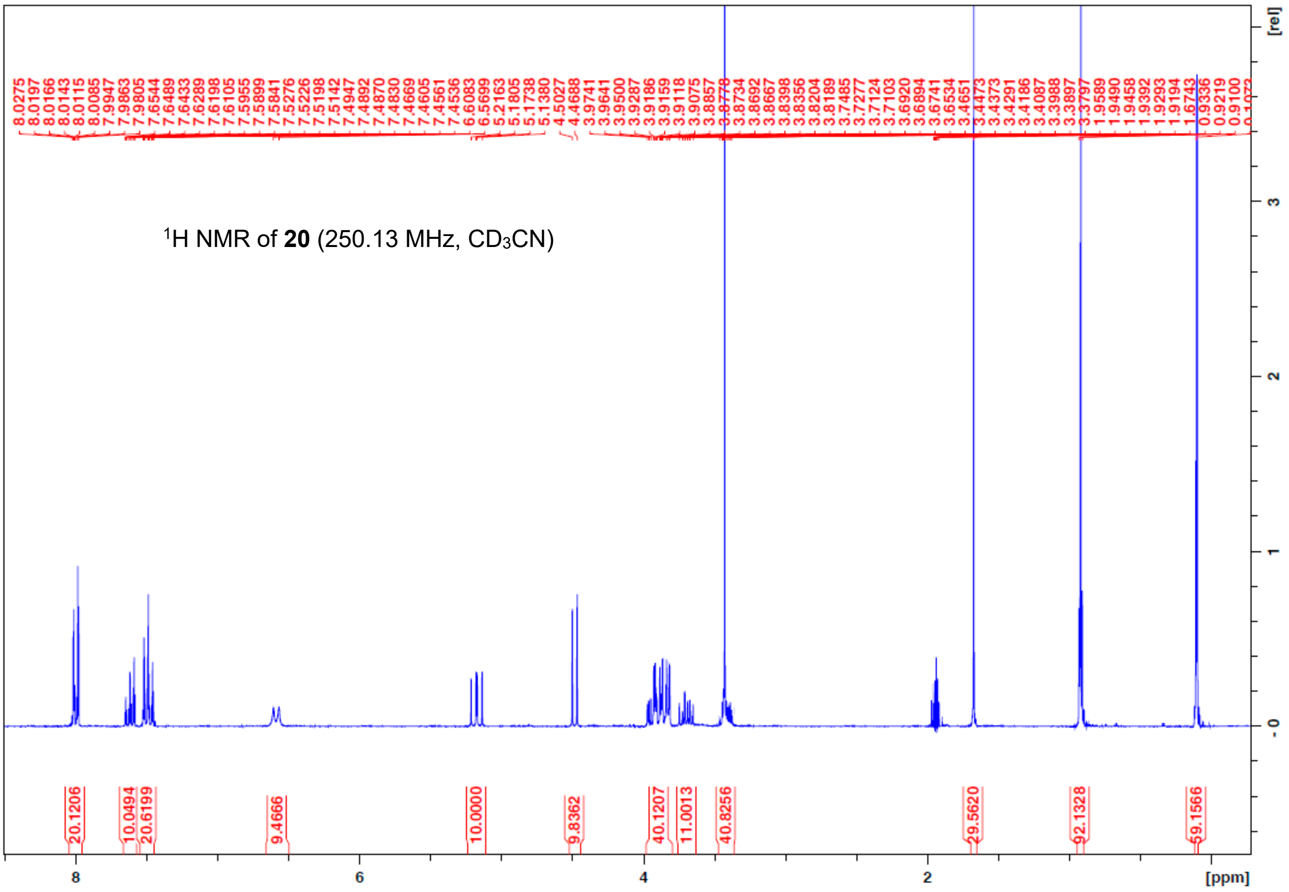

^{1}H NMR of **20** (250.13 MHz, CD_3CN)

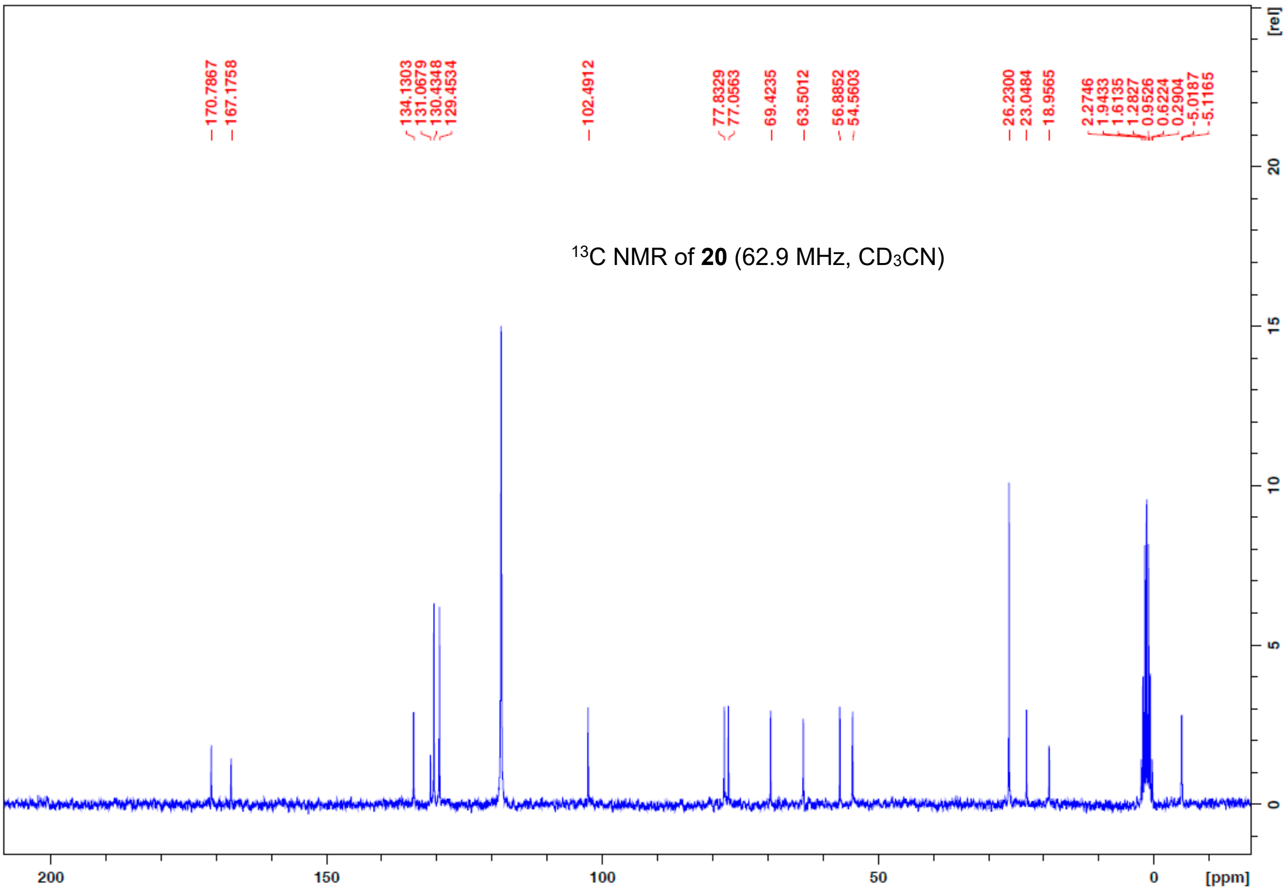

^{13}C NMR of **20** (62.9 MHz, CD_3CN)

COSY spectra of **20** (250.13 MHz, CD_3CN)

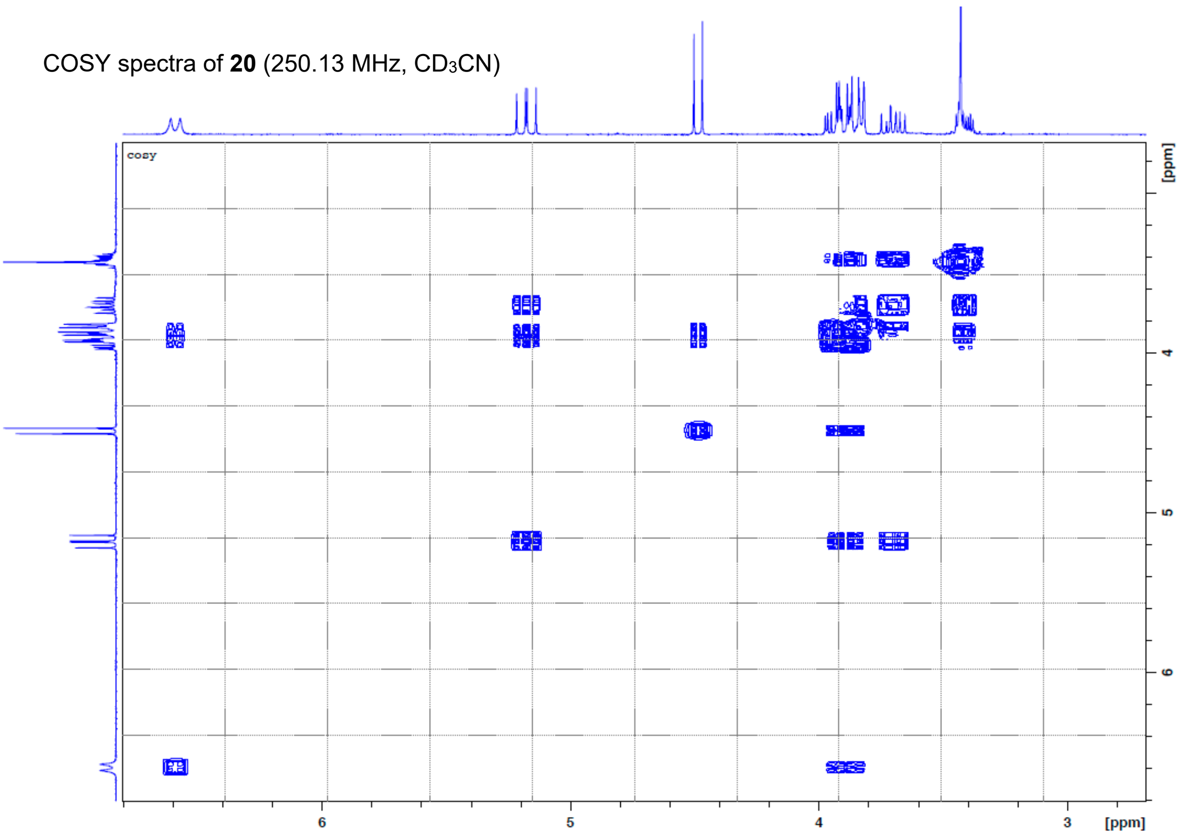

HETCOR spectra of **20** (62.9 MHz, CD_3CN)

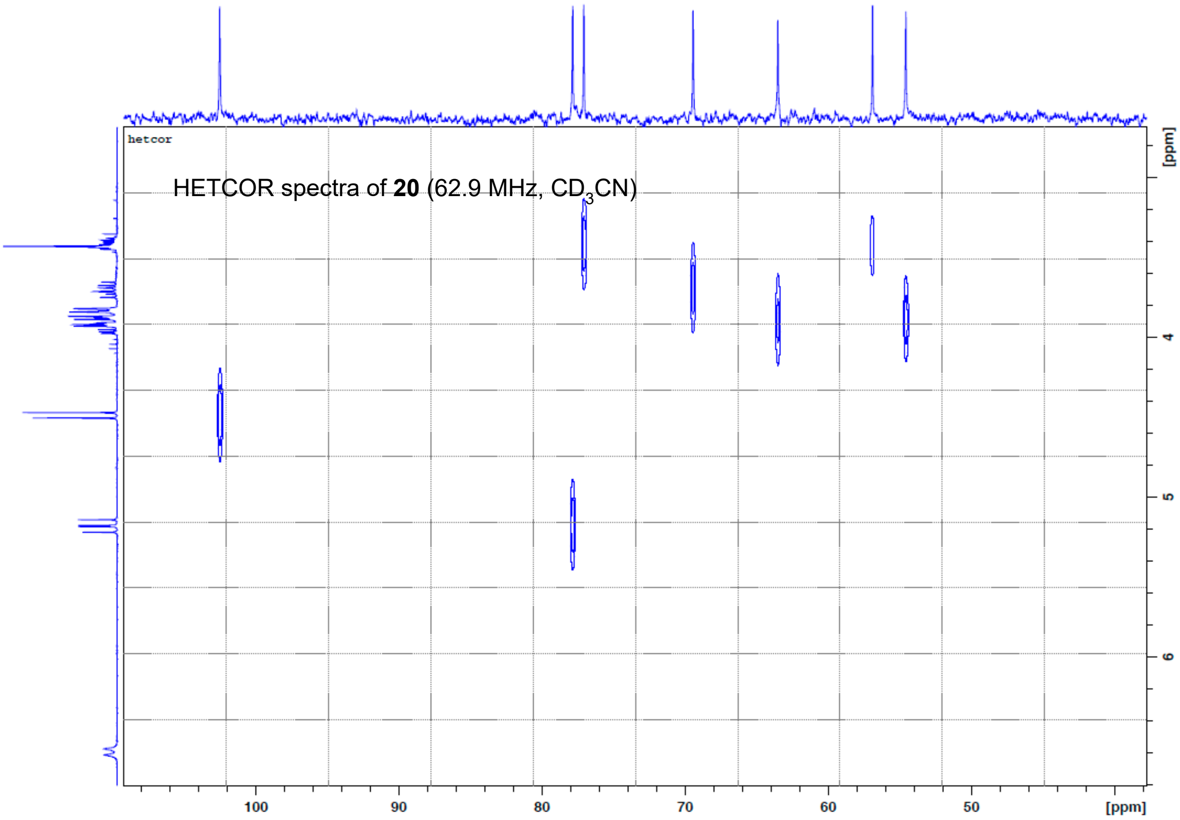

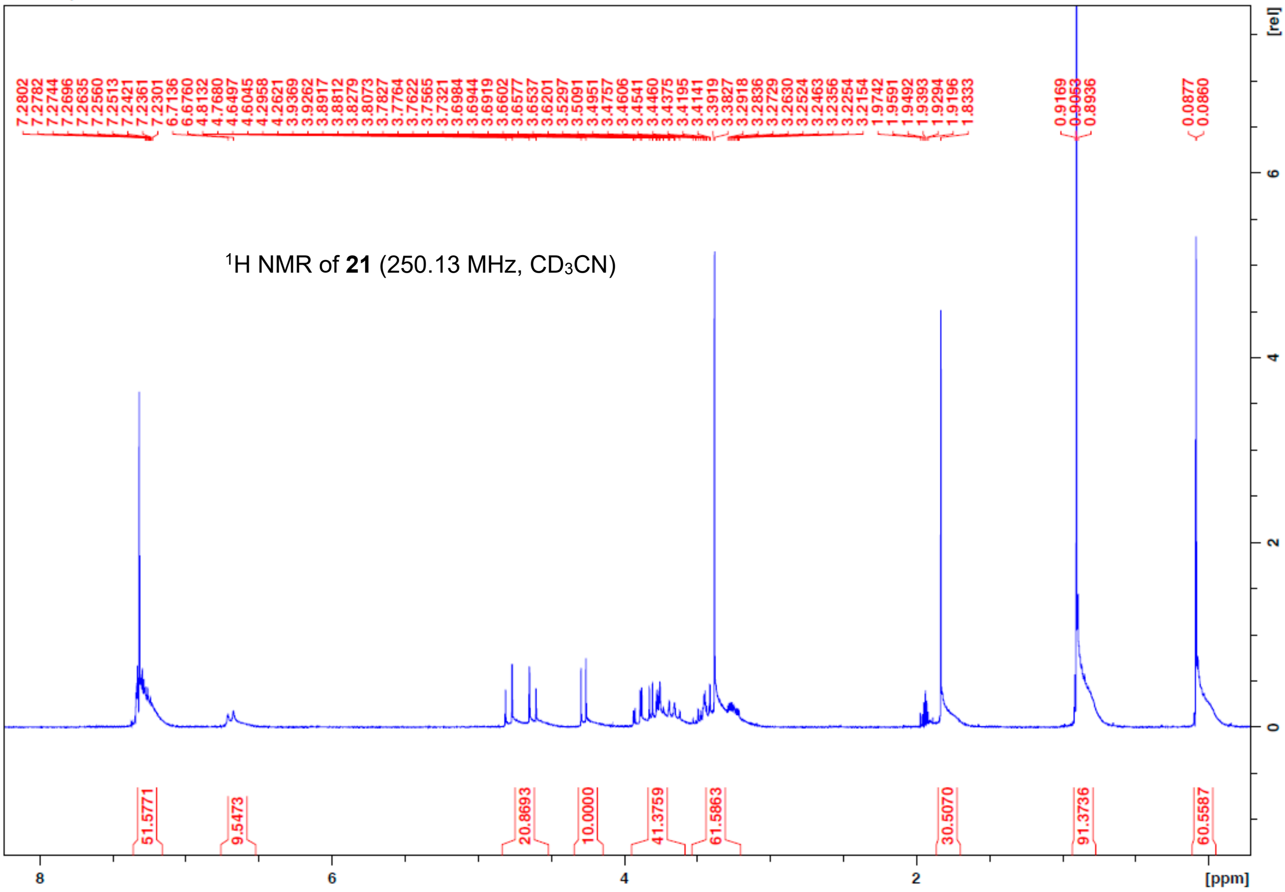
1H NMR of 21 (250.13 MHz, CD3CN)
7.2802 7.2782 7.2744 7.2696 7.2635 7.2560 7.2513 7.2421 7.2361 7.2301 6.7136 6.6760 4.8132 4.7680 4.6497 4.6045 4.2958 4.2621 3.9369 3.9262 3.8917 3.8812 3.8279 3.8073 3.7827 3.7764 3.7622 3.7565 3.7321 3.6984 3.6944 3.6919 3.6602 3.6577 3.6537 3.6201 3.5297 3.5091 3.4951 3.4757 3.4606 3.4541 3.4460 3.4375 3.4195 3.4141 3.3919 3.3827 3.2918 3.2836 3.2729 3.2630 3.2524 3.2463 3.2356 3.2254 3.2154 1.9742 1.9591 1.9492 1.9393 1.9294 1.9196 1.8333
0.9169 0.9053 0.8936
0.0877 0.0860
51.5771
9.5473
20.8693
10.0000
41.3759
61.5863
30.5070
91.3736
60.5587
[rel]
[ppm]

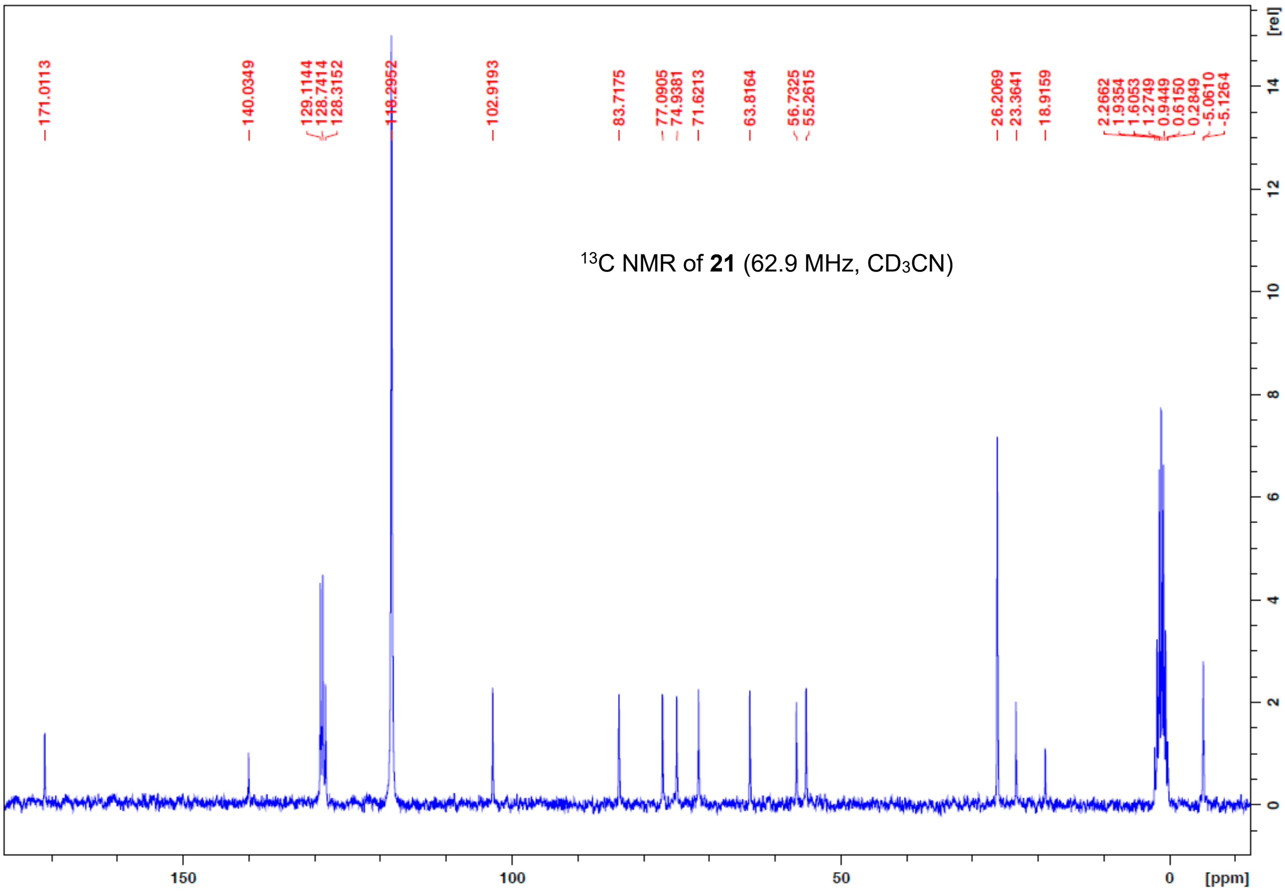
13C NMR of 21 (62.9 MHz, CD3CN)
171.0113
140.0349
129.1144 128.7414 128.3152
118.2952
102.9193
83.7175
77.0905 74.9381 71.6213
63.8164
56.7325 55.2615
26.2069 23.3641 18.9159
2.2662 1.9354 1.6053 1.2749 0.9449 0.6150 0.2849 -5.0610 -5.1264
[rel]
[ppm]

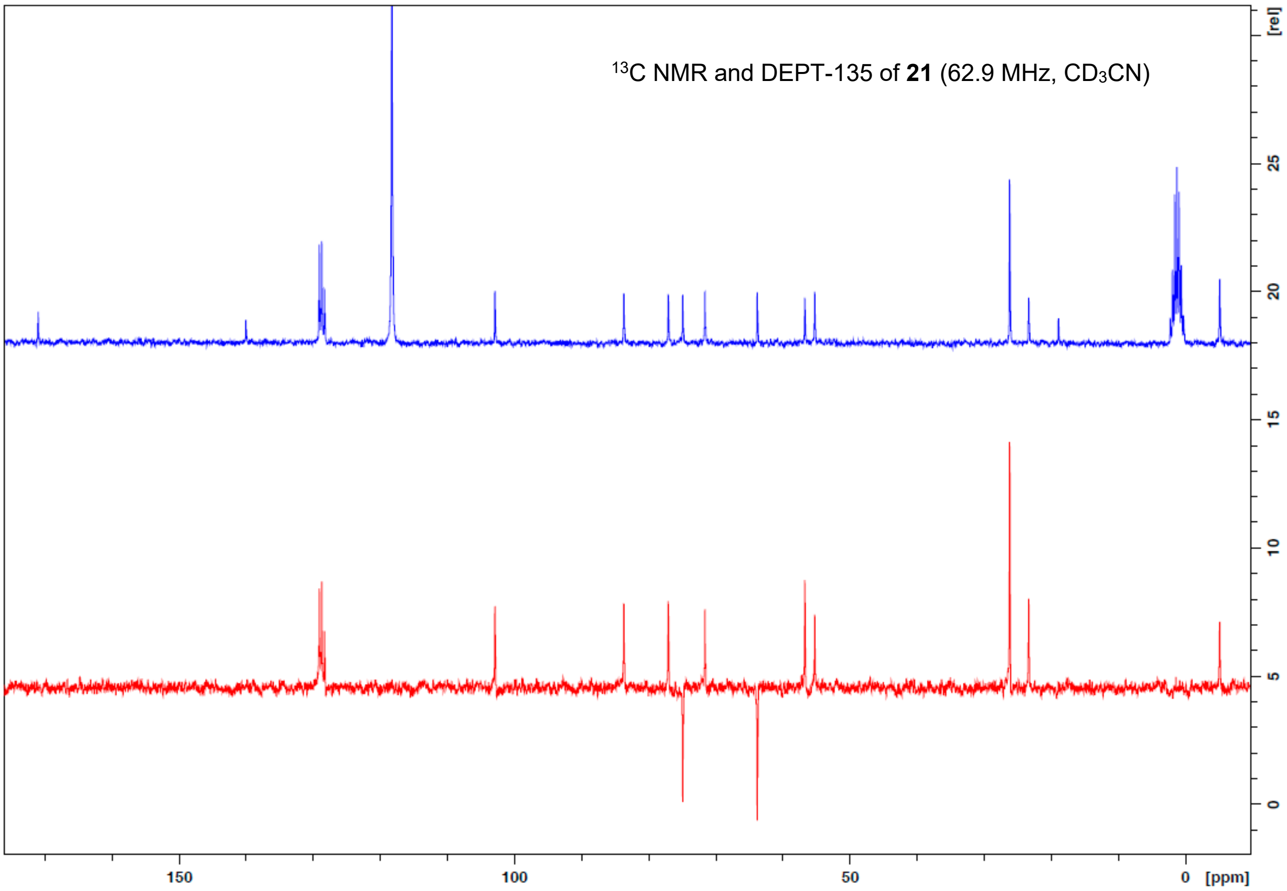

13C NMR and DEPT-135 of 21 (62.9 MHz, CD3CN)

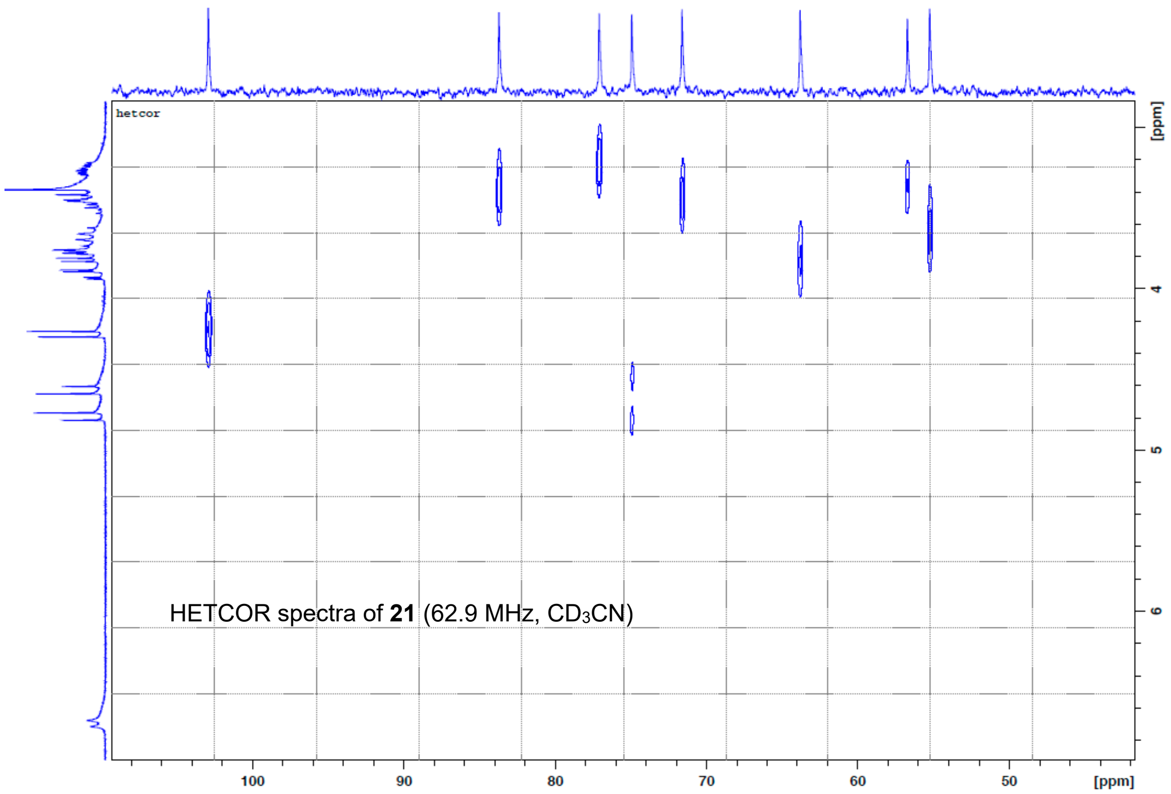

hetcor
HETCOR spectra of 21 (62.9 MHz, CD3CN)

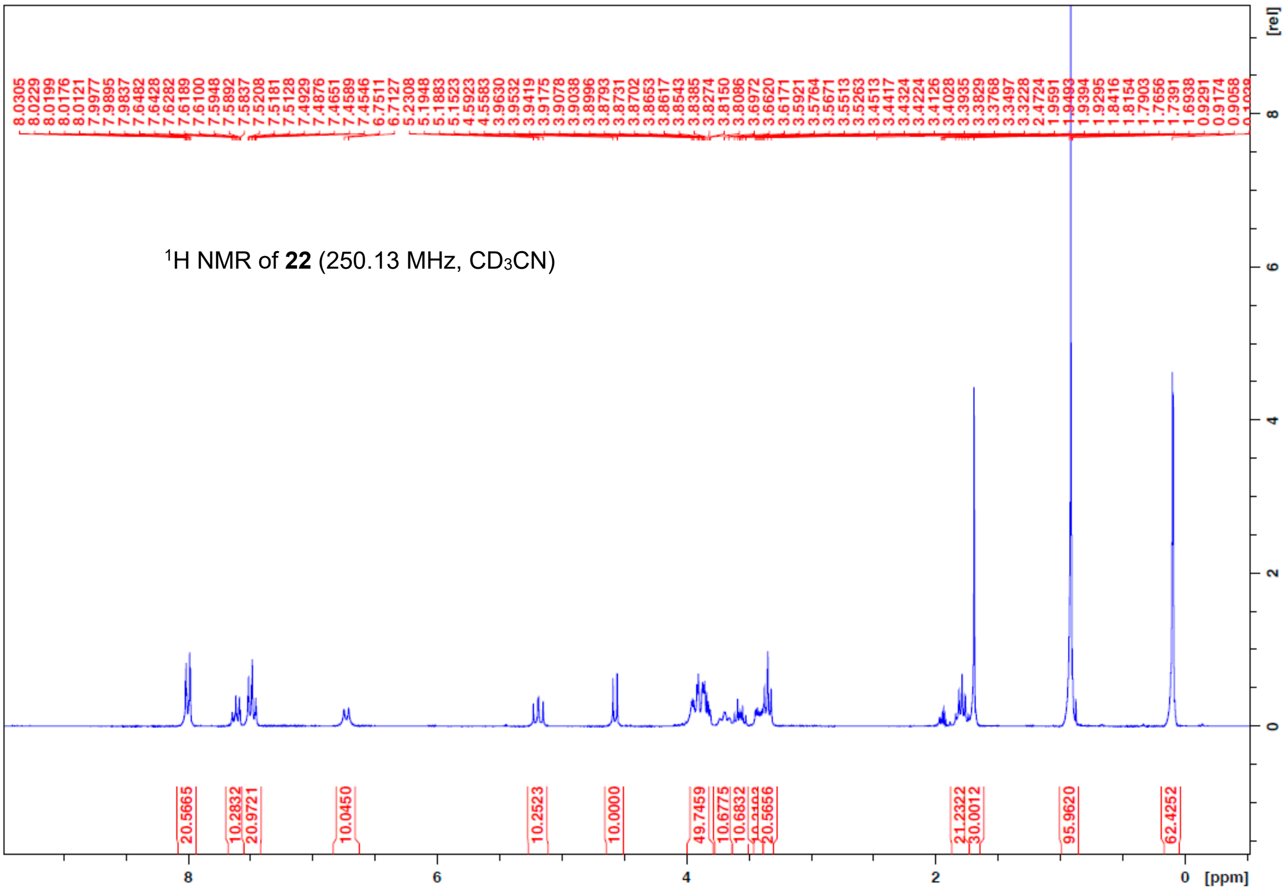

^{1}H NMR of **22** (250.13 MHz, CD_3CN)

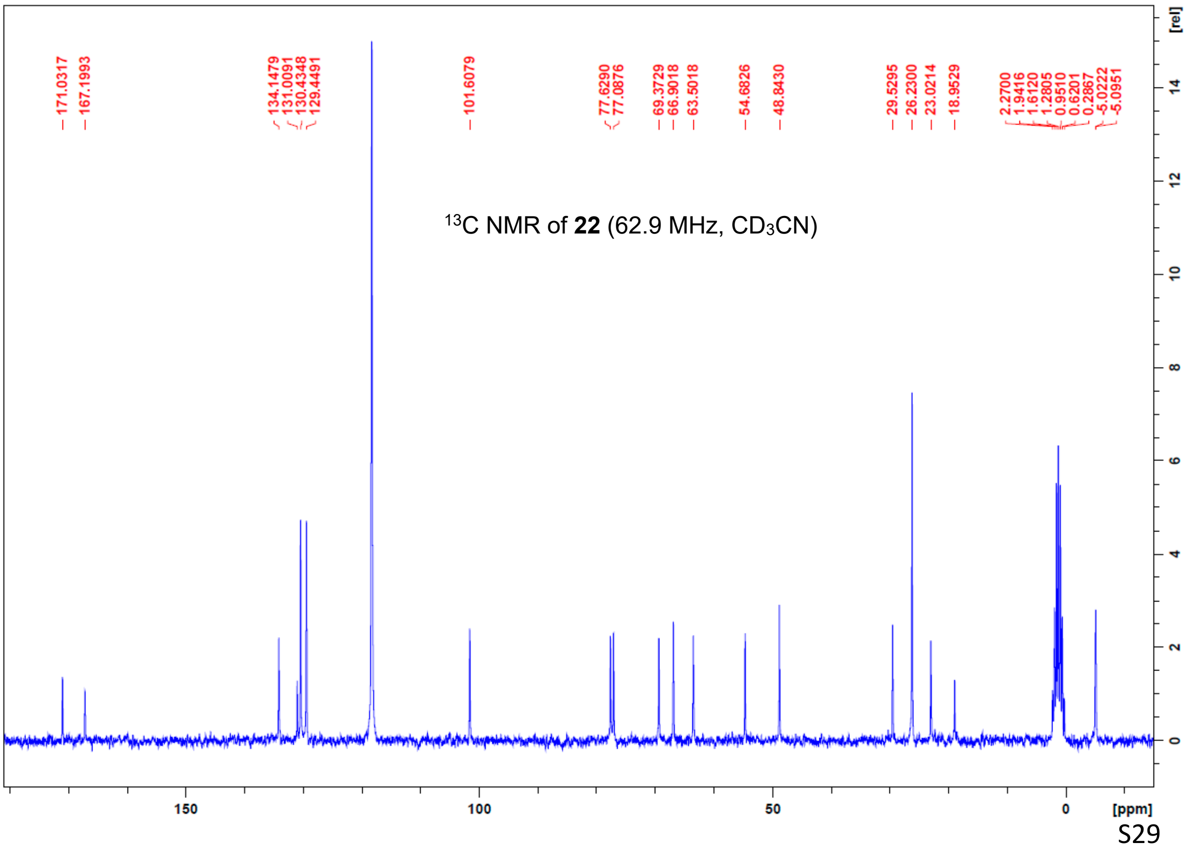

^{13}C NMR of **22** (62.9 MHz, CD_3CN)

COSY spectra of **22** (250.13 MHz, CD_3CN)

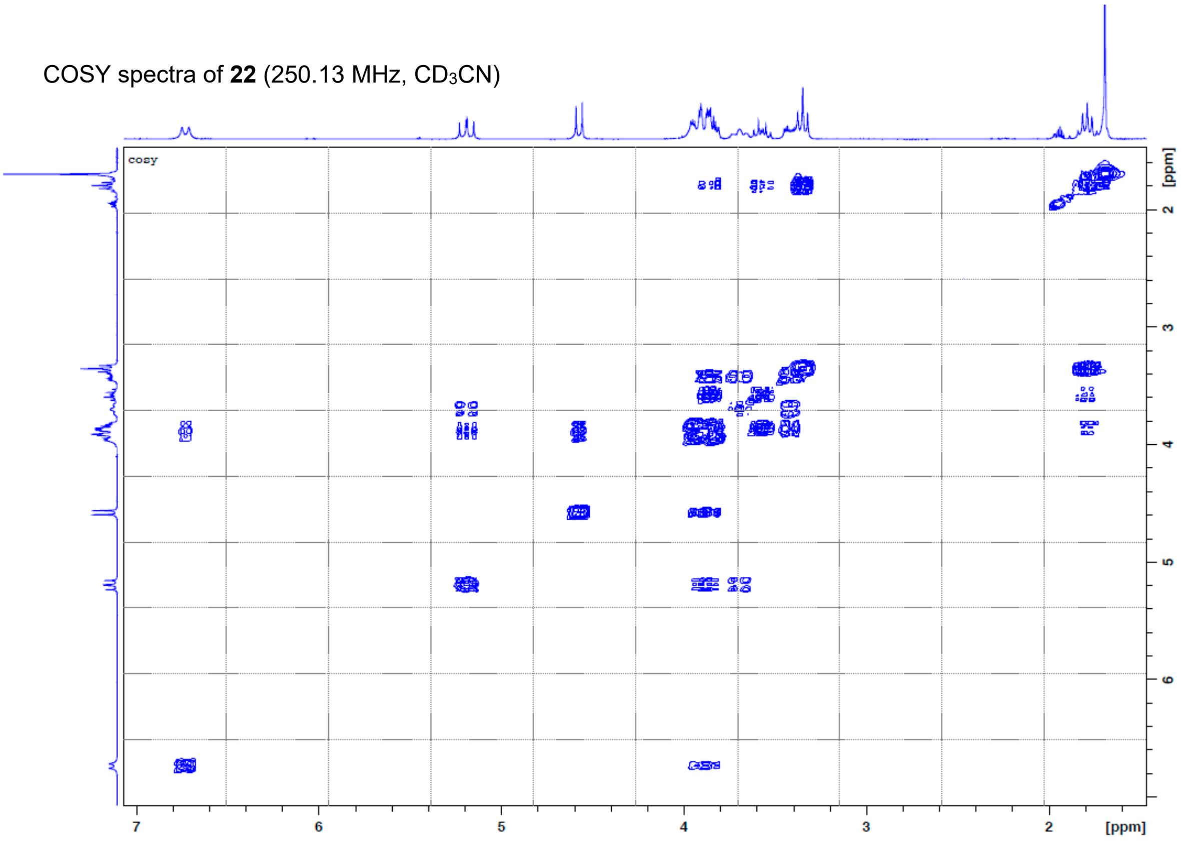

HETCOR spectra of **22** (62.9 MHz, CD_3CN)

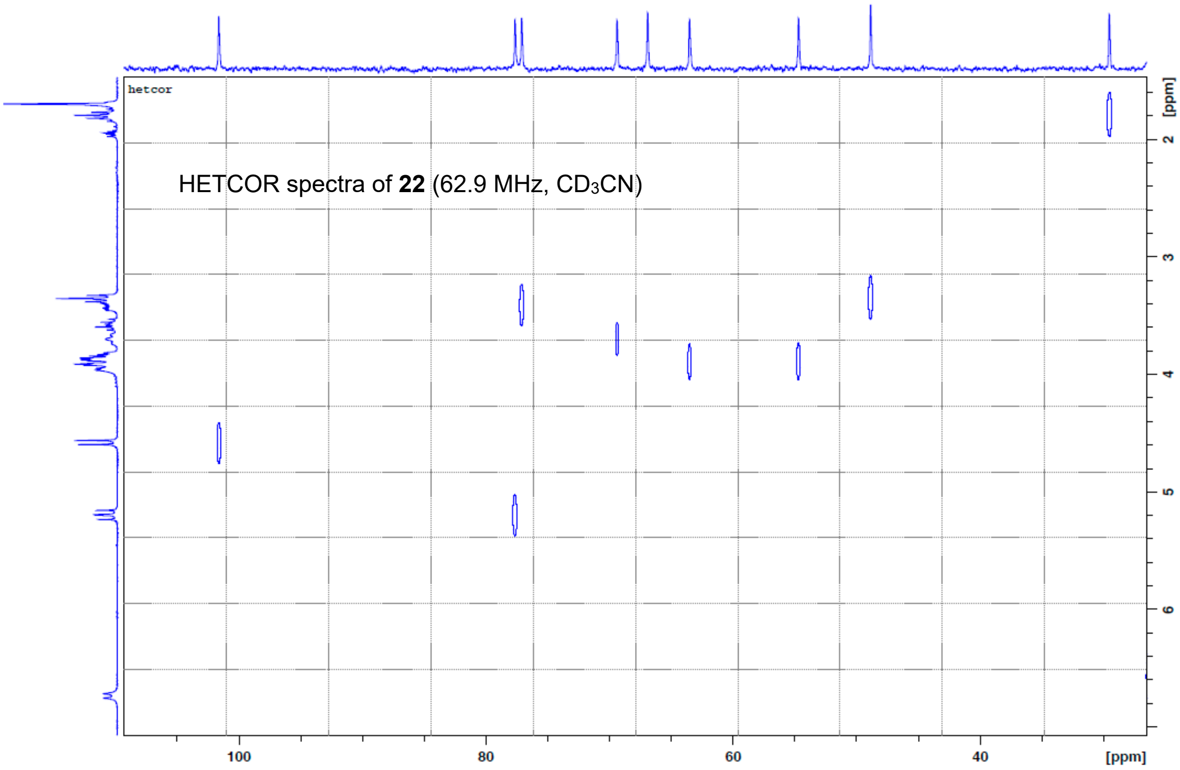

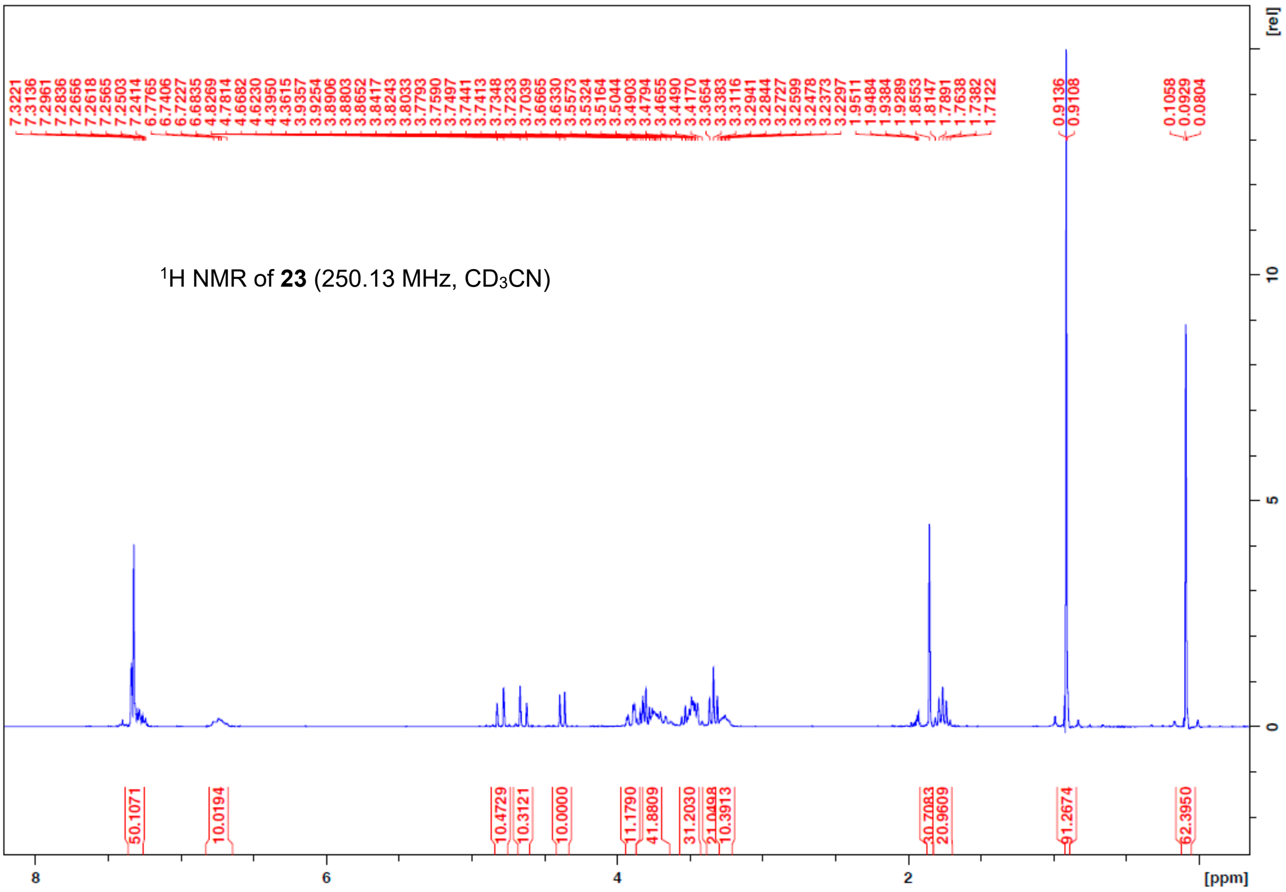
^{1}H NMR of 23 (250.13 MHz, CD3CN)
[ppm]
[rel]

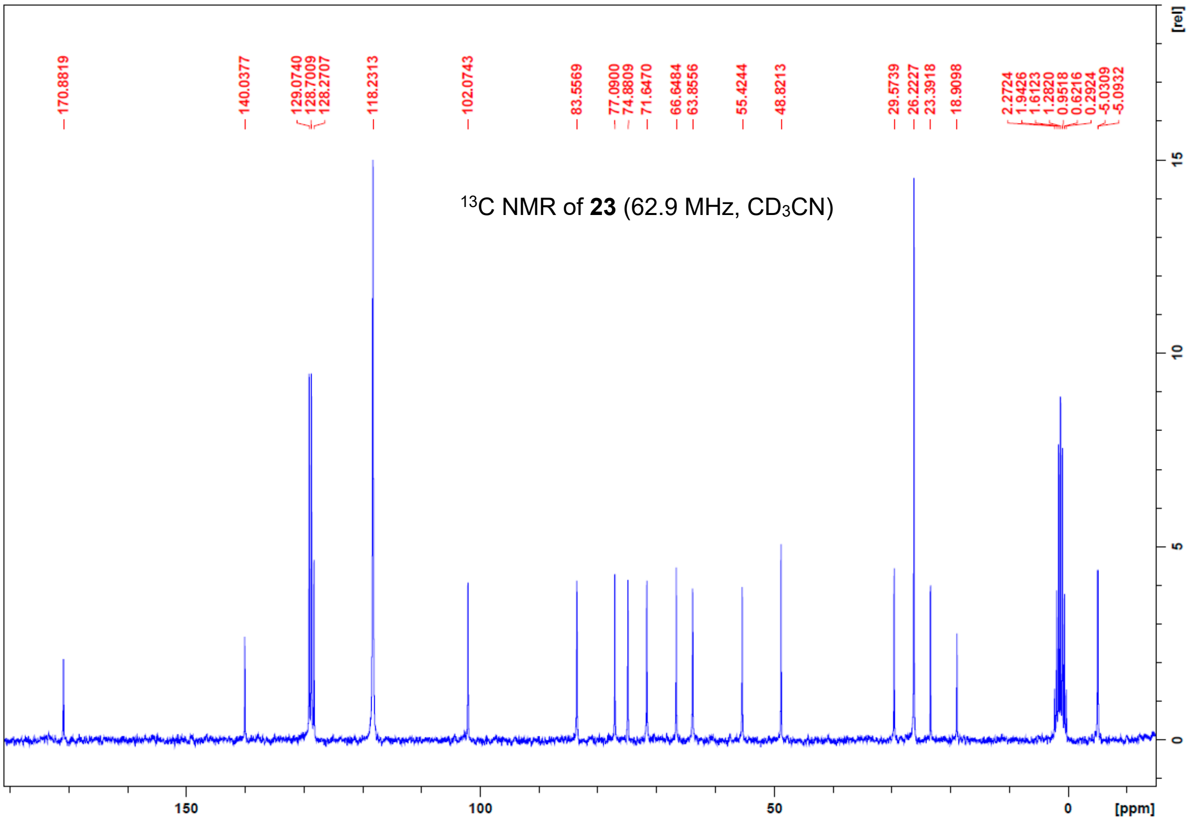
^{13}C NMR of 23 (62.9 MHz, CD3CN)
170.8819
140.0377
129.0740
128.7009
128.2707
118.2313
102.0743
83.5569
77.0900
74.8809
71.6470
66.6484
63.8556
55.4244
48.8213
29.5739
26.2227
23.3918
18.9098
[ppm]
[rel]

COSY spectra of **23** (250.13 MHz, CD_3CN)

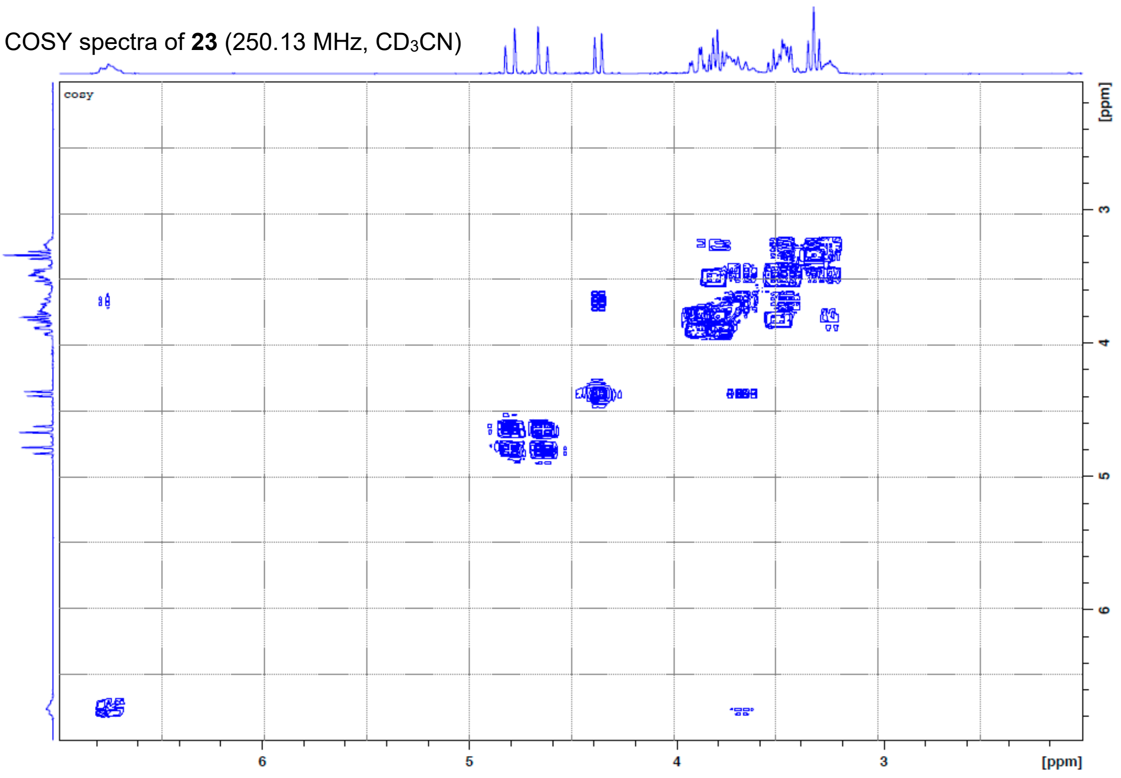

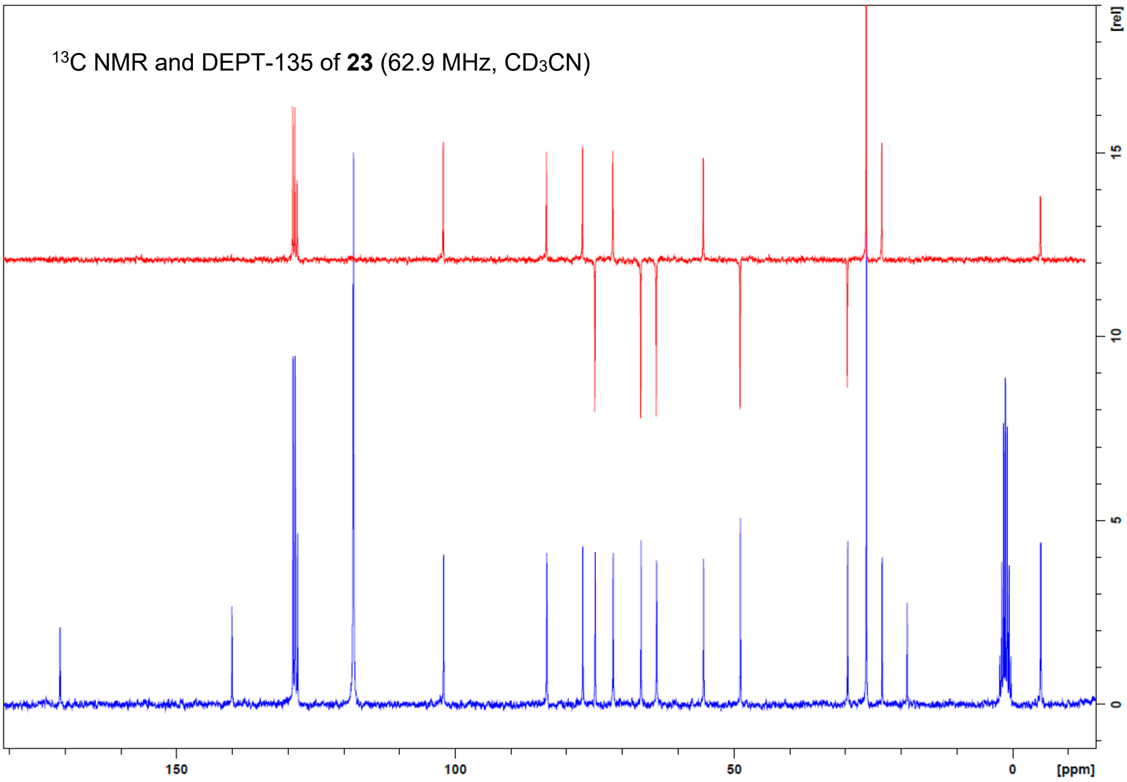

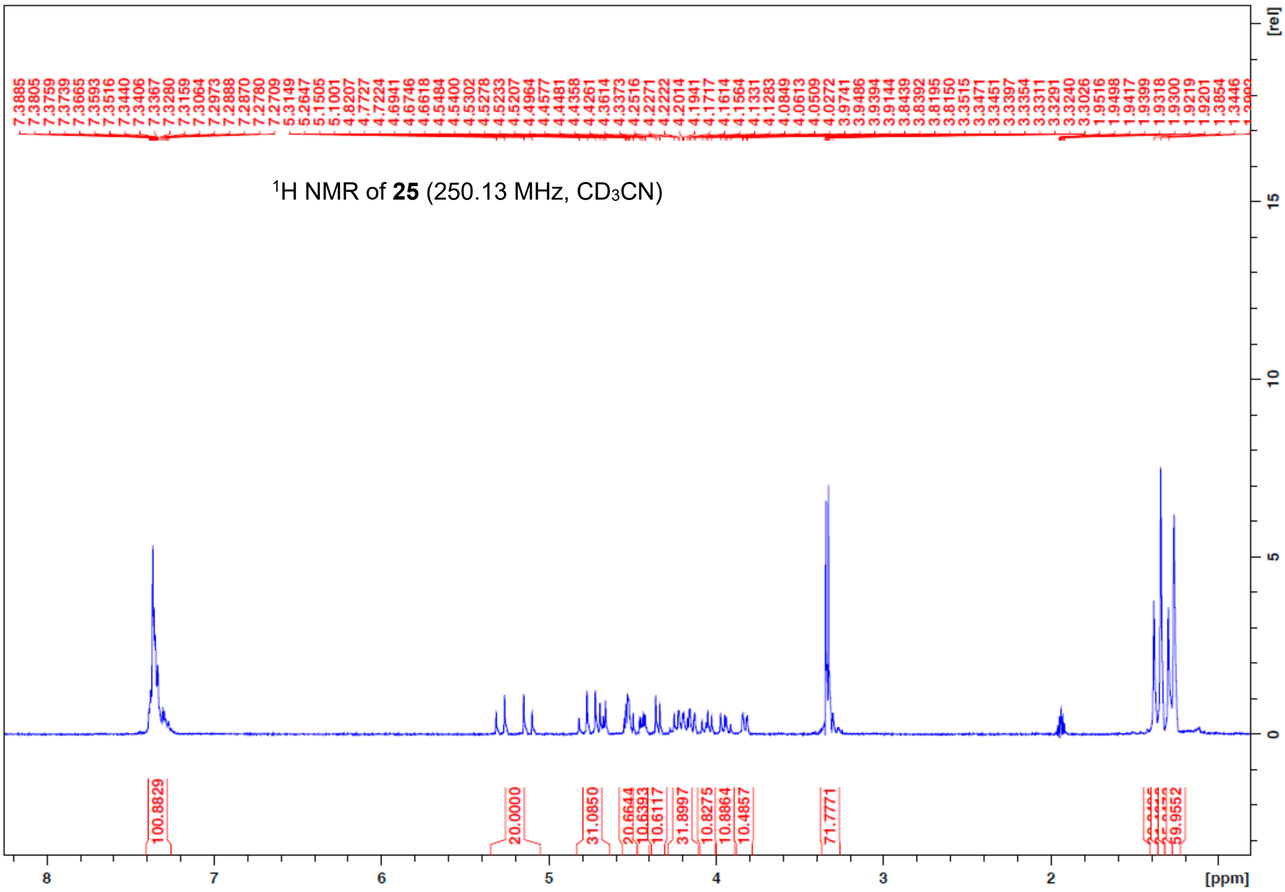

^{1}H NMR of **25** (250.13 MHz, CD_3CN)

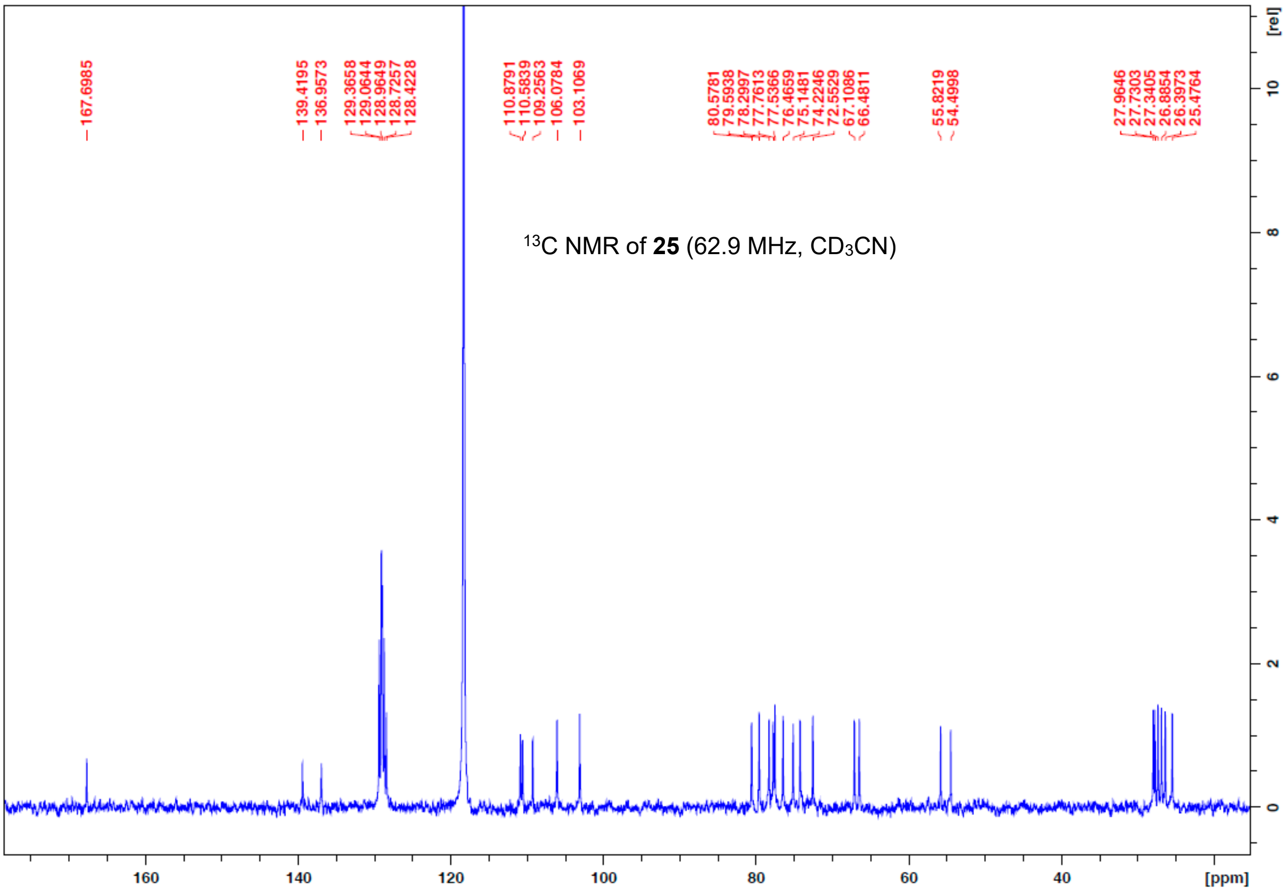

^{13}C NMR of **25** (62.9 MHz, CD_3CN)

HETCOR spectra of **25** (62.9 MHz, CD_3CN)

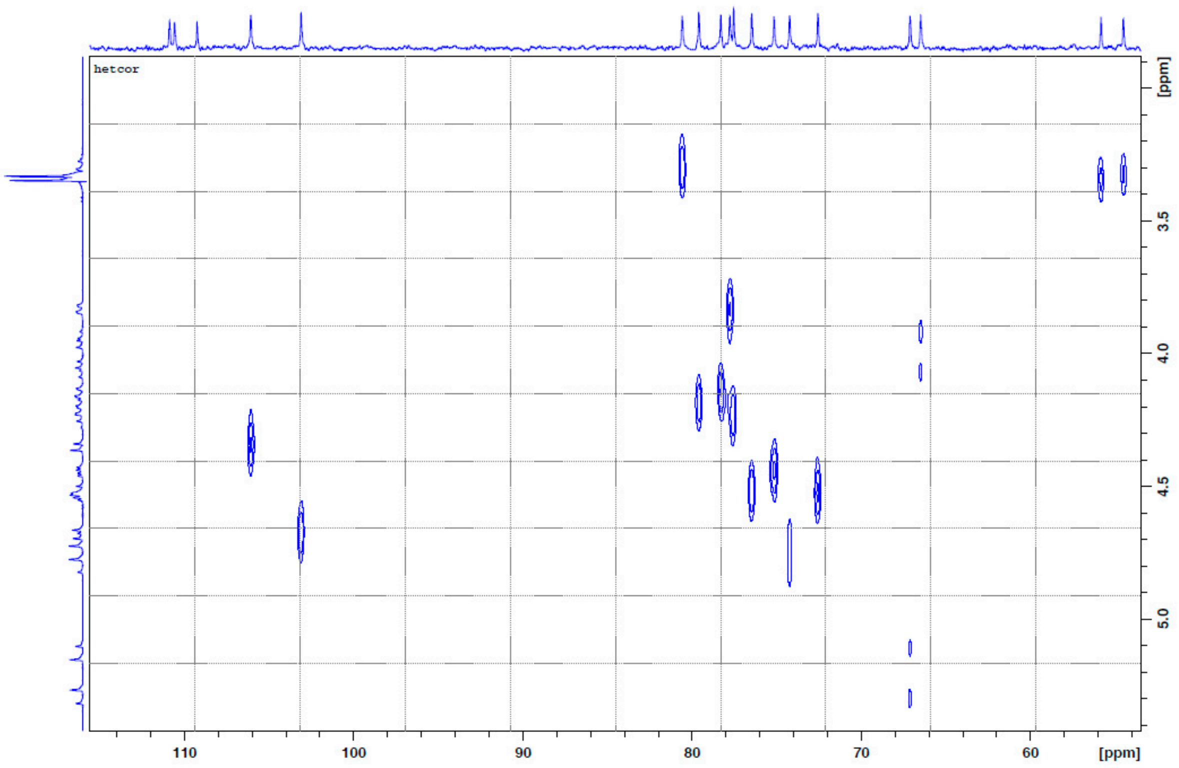

COSY spectra of **25** (250.13 MHz, CD_3CN)

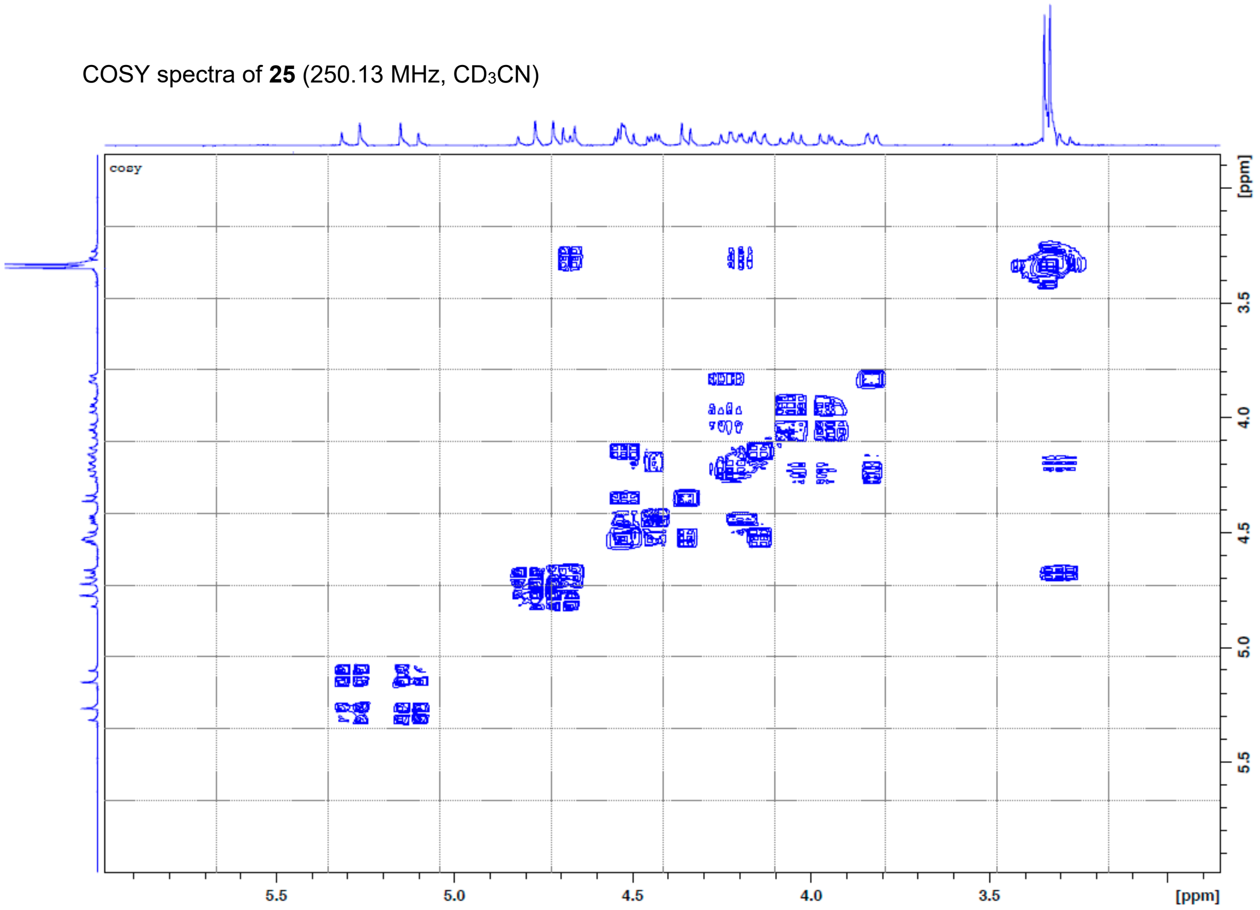

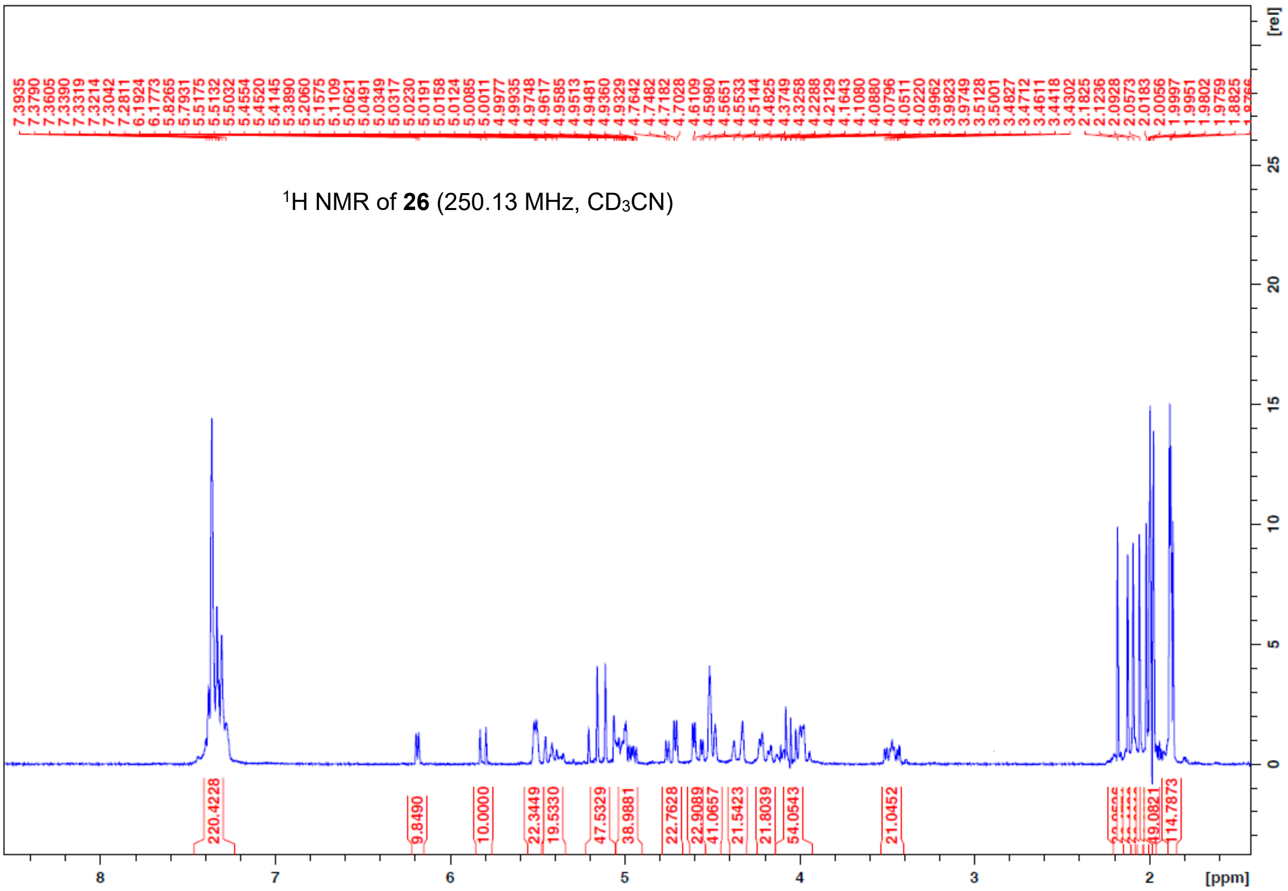
1H NMR of 26 (250.13 MHz, CD3CN)

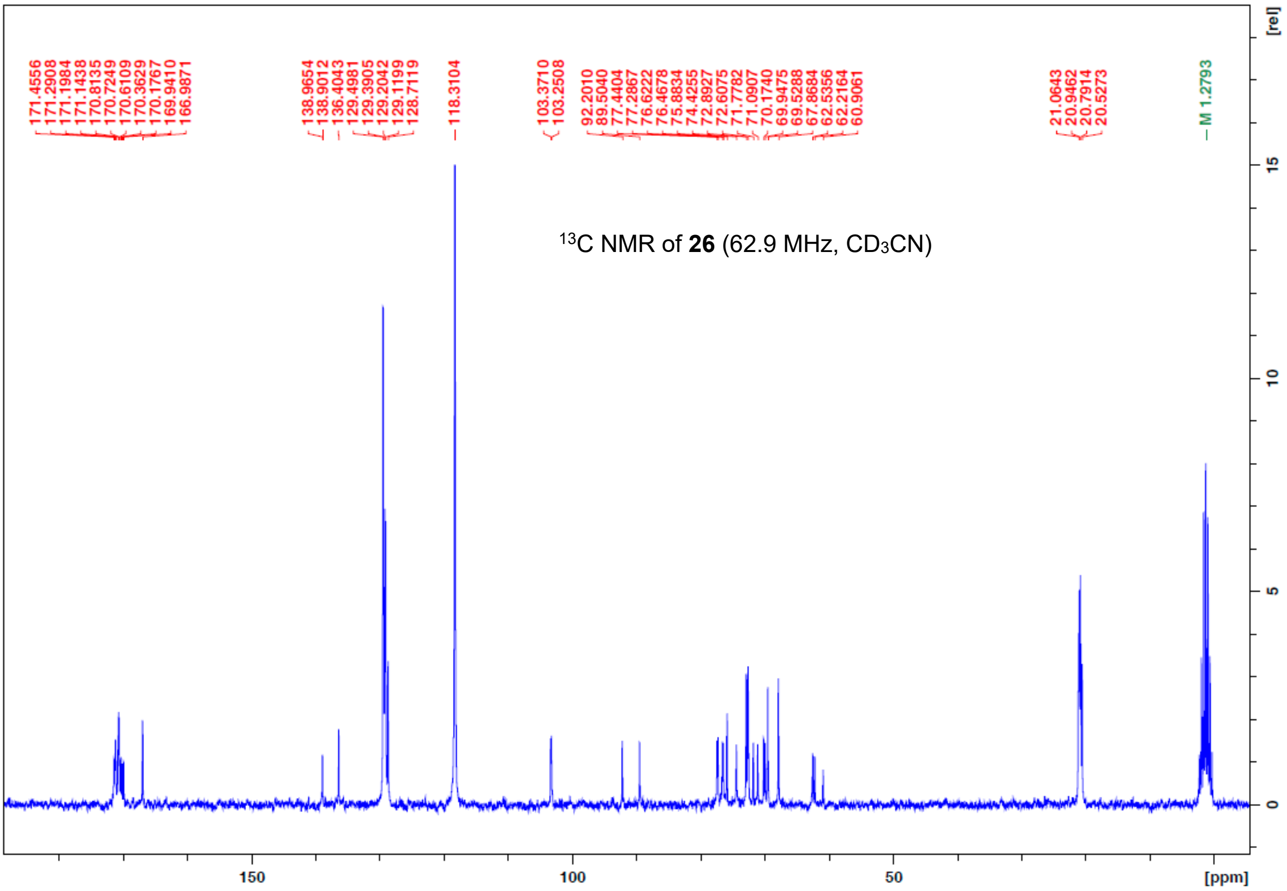
13C NMR of 26 (62.9 MHz, CD3CN)

COSY spectra of **26** (250.13 MHz, CD_3CN)

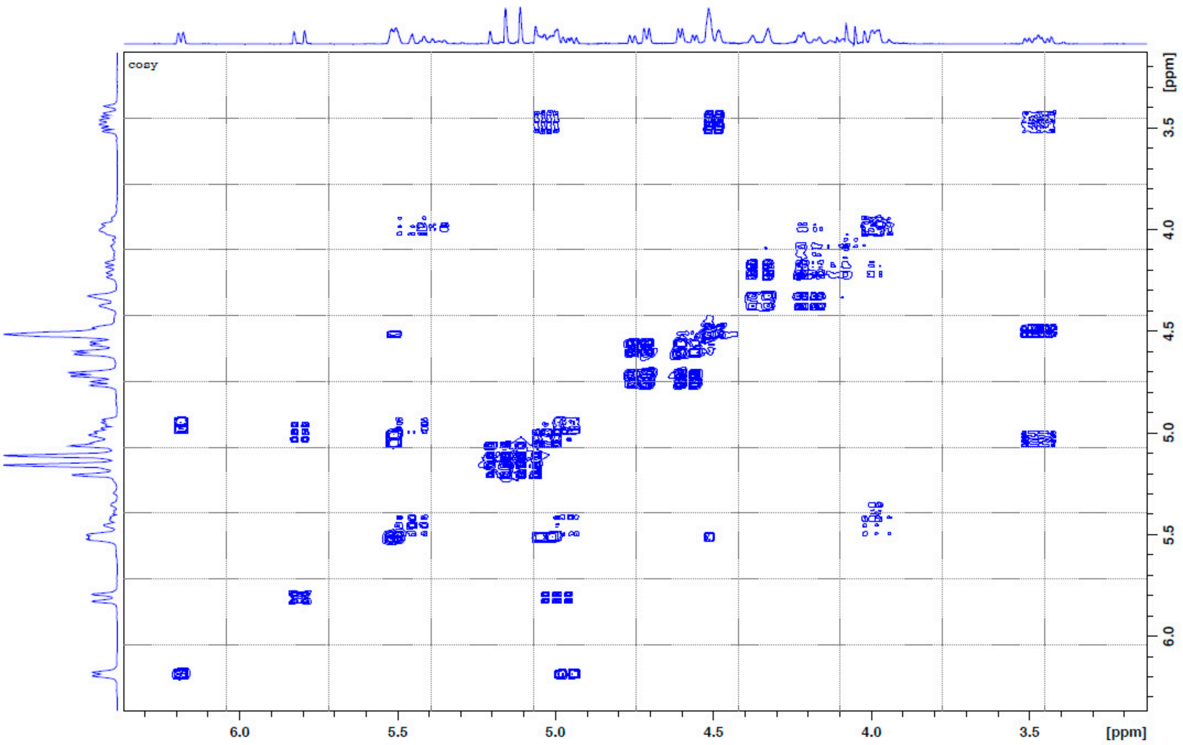

^{1}H NMR of **27** (250.13 MHz, CD_3CN)

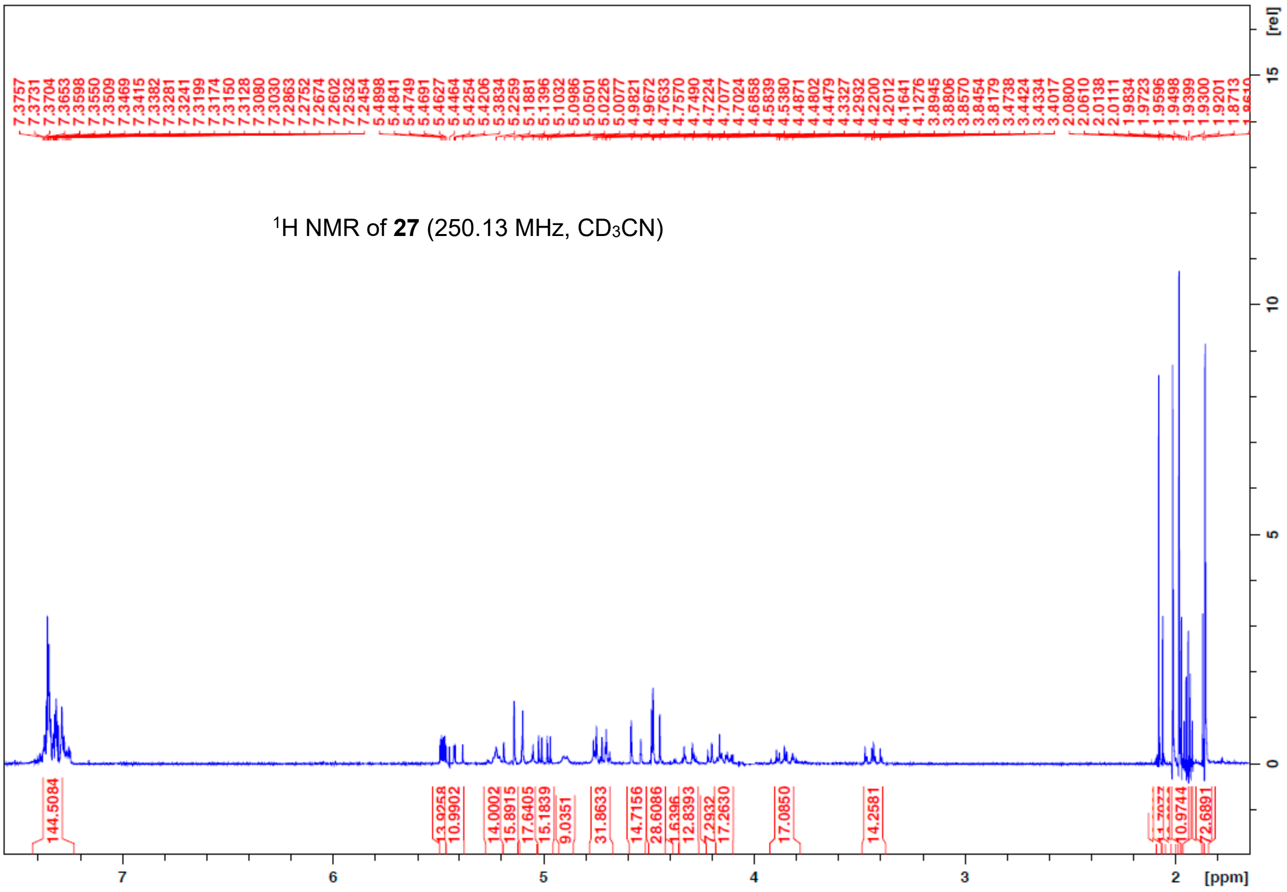

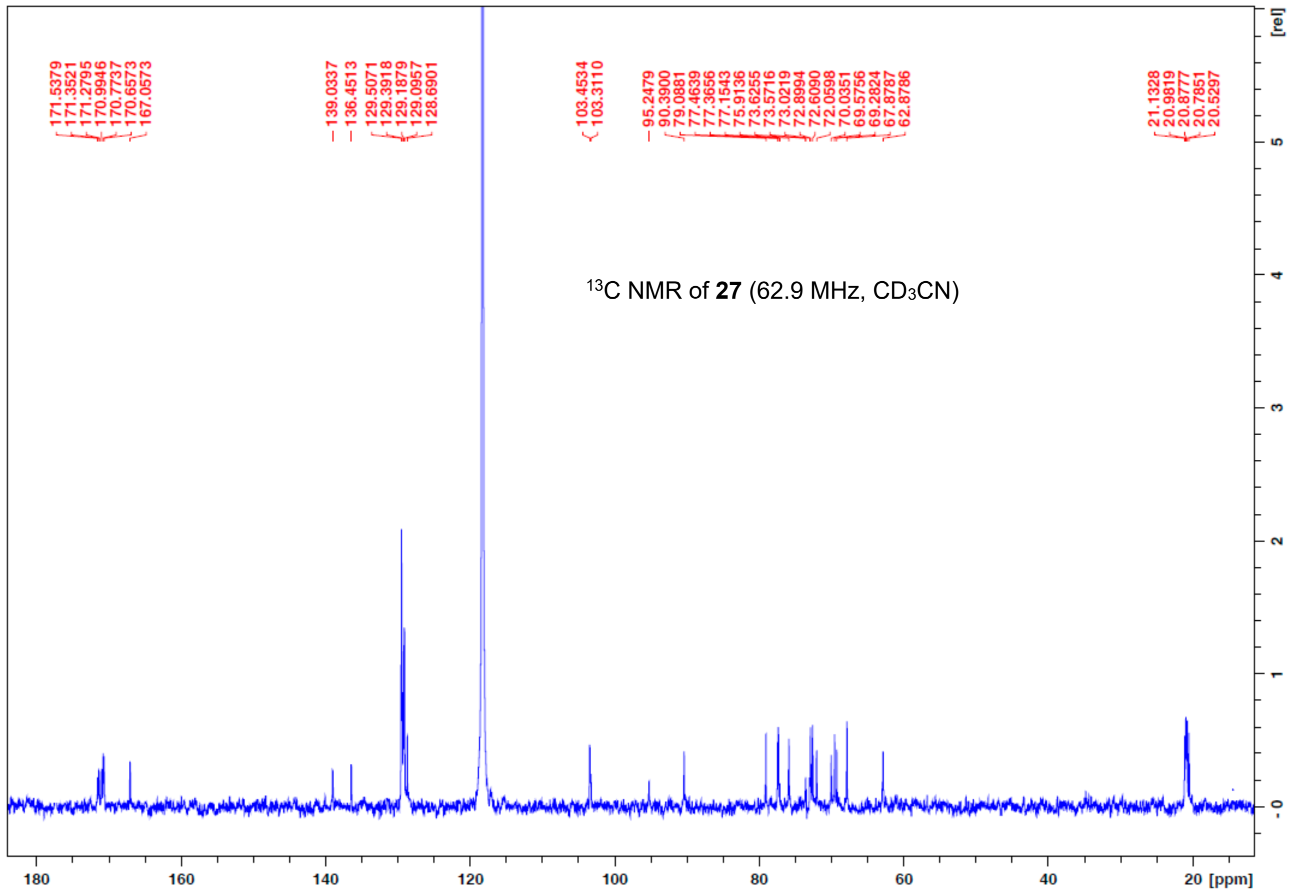

13C NMR of 27 (62.9 MHz, CD3CN)
[ppm]
[rel]

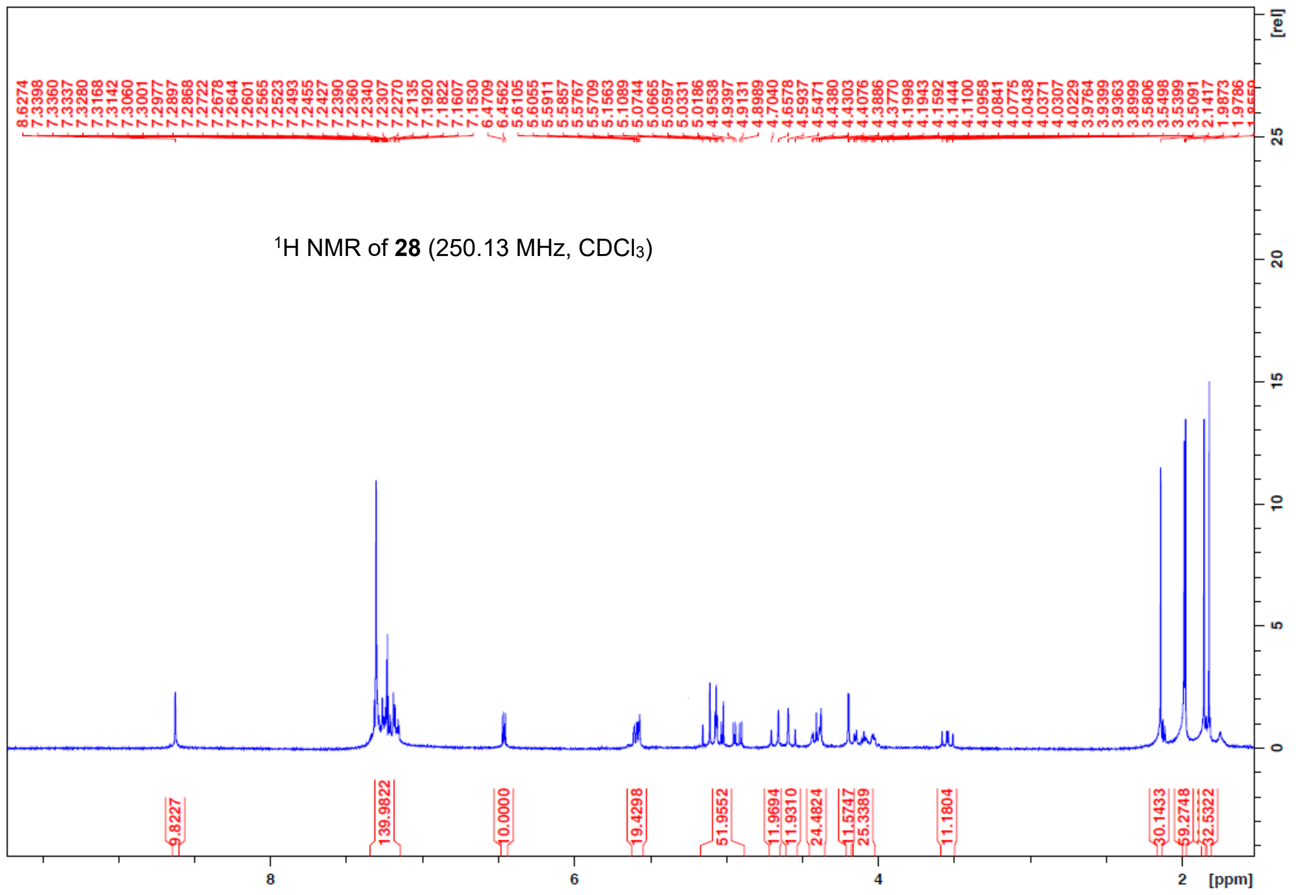

1H NMR of 28 (250.13 MHz, CDCl3)
[ppm]
[rel]

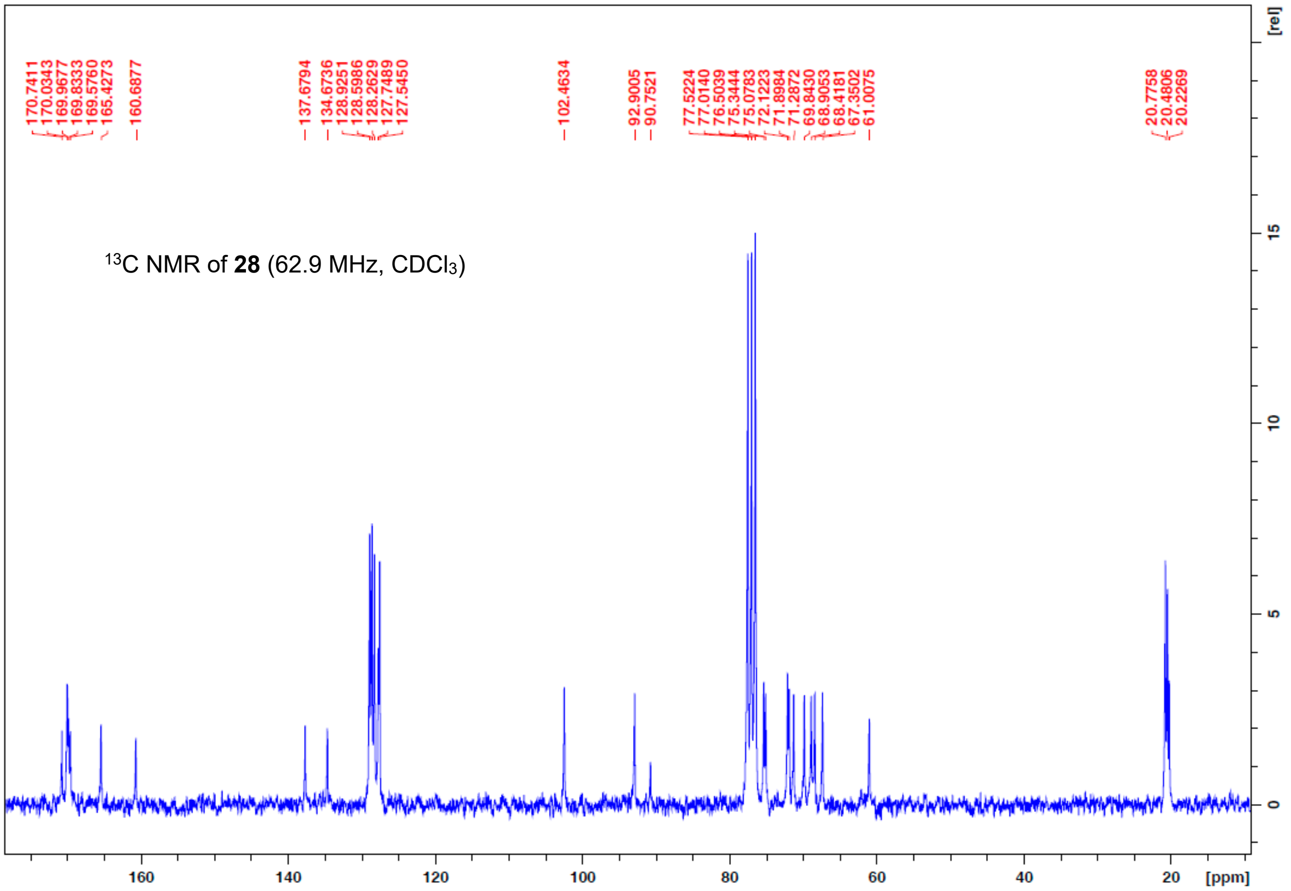

^{13}C NMR of **28** (62.9 MHz, $CDCl_3$)

COSY spectra of **28** (250.13 MHz, $CDCl_3$)

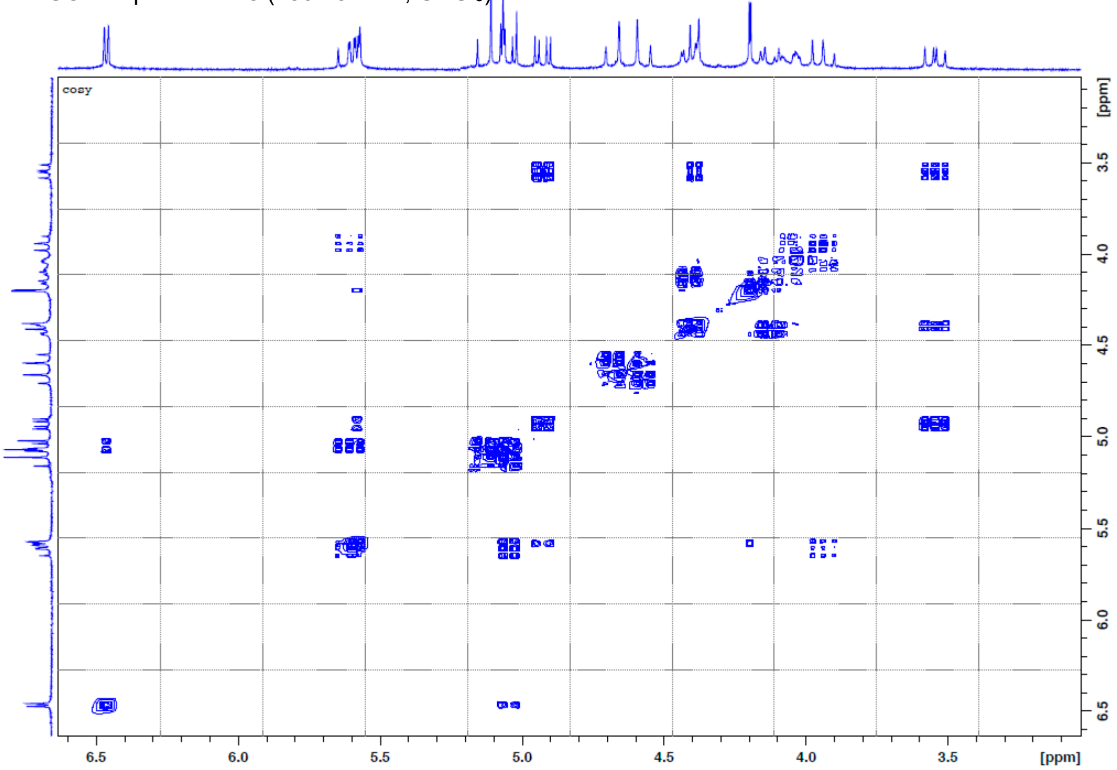

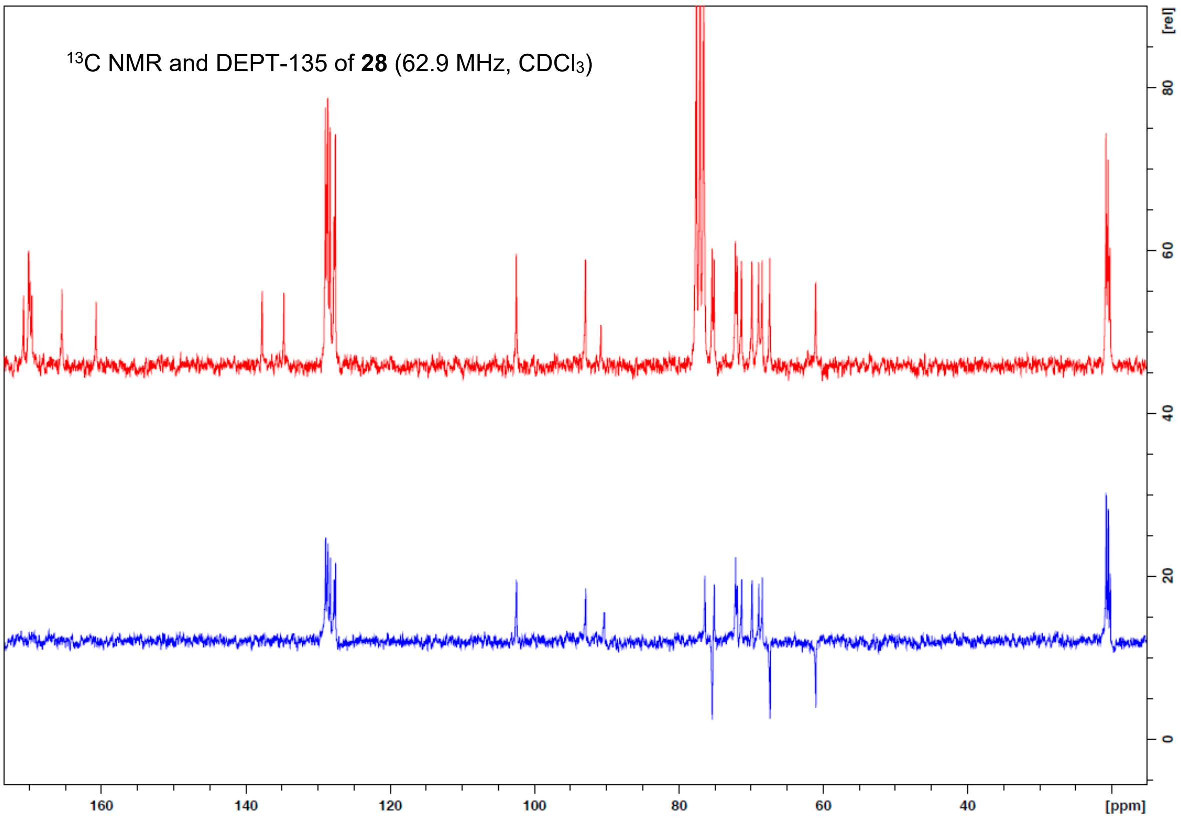

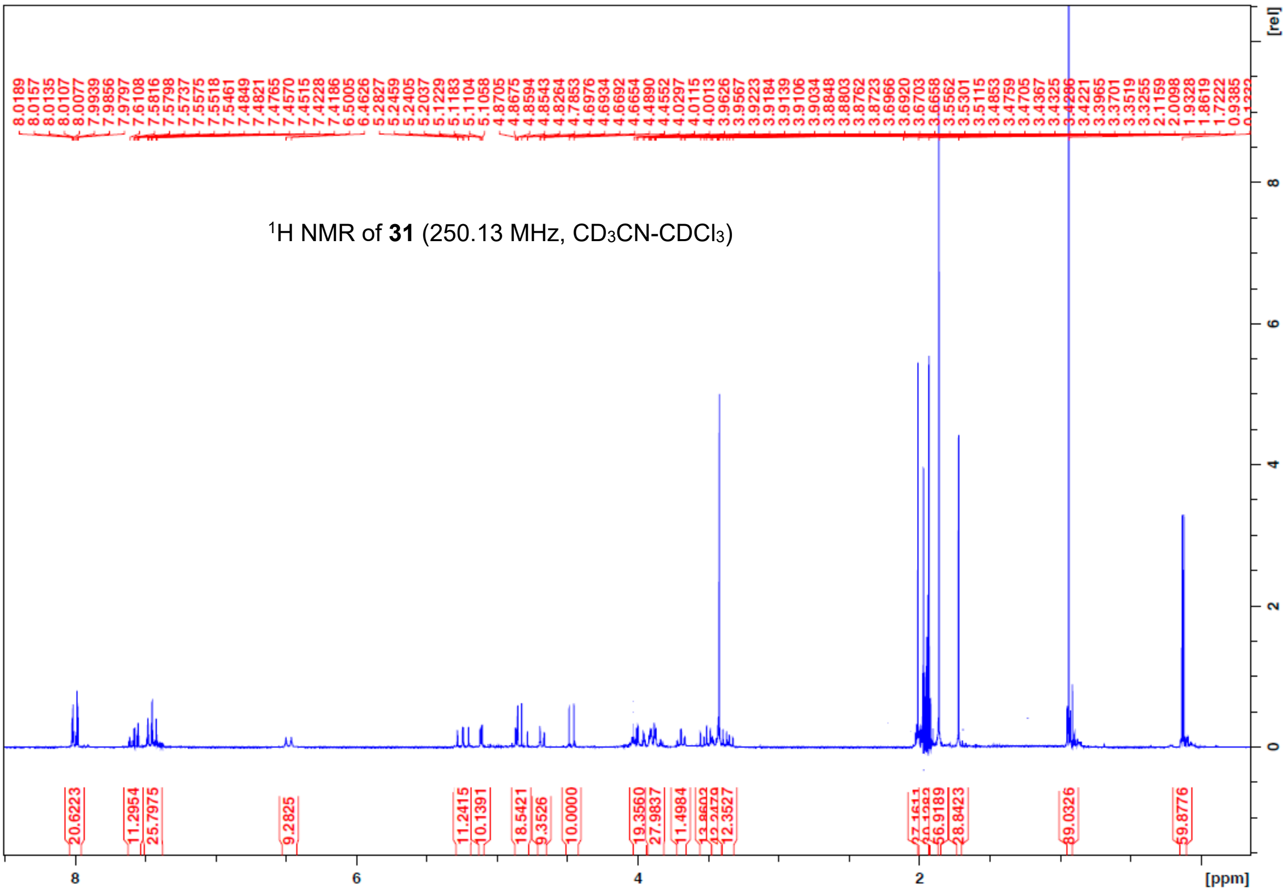

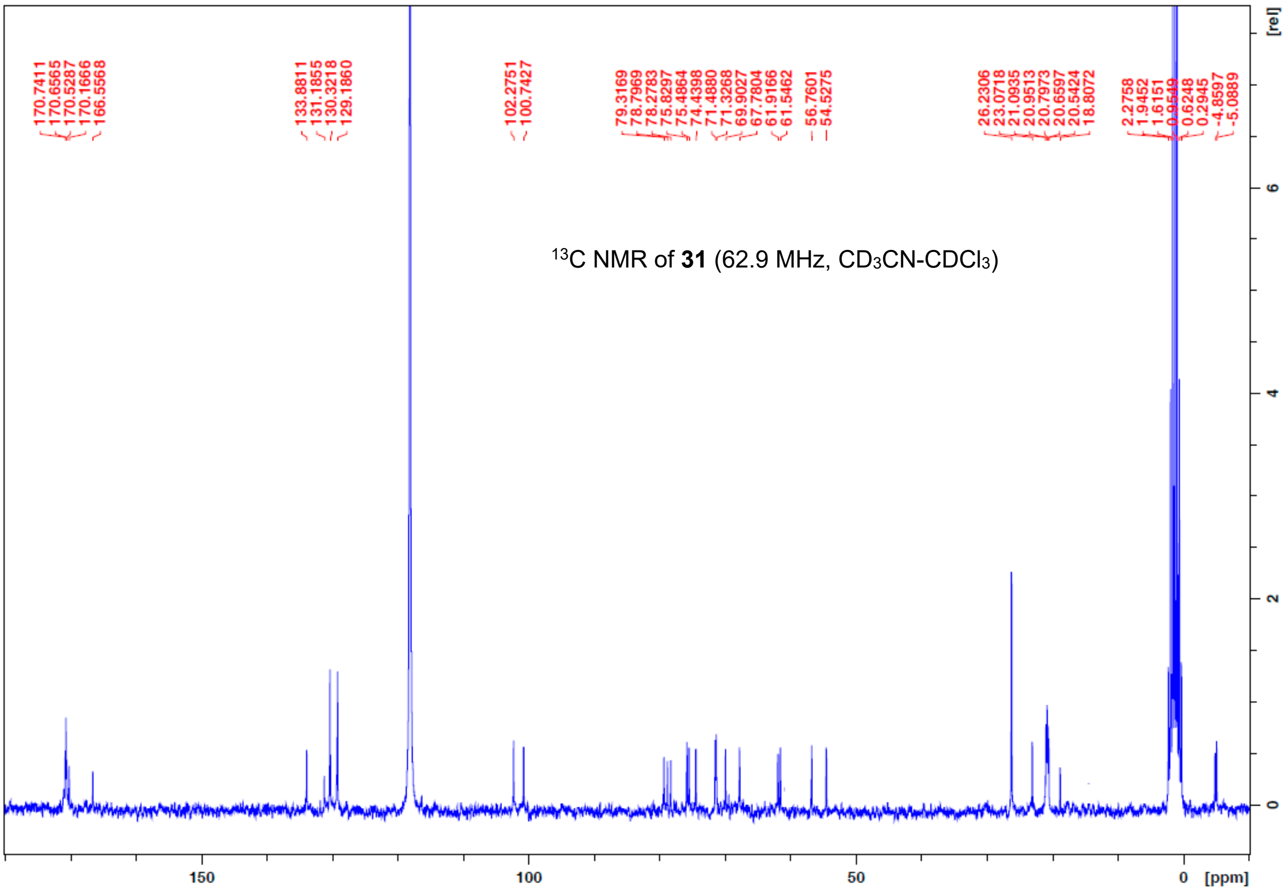

13C NMR of 31 (62.9 MHz, CD3CN-CDCl3)
170.7411
170.6565
170.5287
170.1666
166.5568
133.8811
131.1855
130.3218
129.1860
102.2751
100.7427
79.3169
78.7969
78.2783
75.8297
75.4864
74.4398
71.4880
71.3268
69.9027
67.7804
61.9166
61.5462
56.7601
54.5275
26.2306
23.0718
21.0935
20.9513
20.7973
20.6597
20.5424
18.8072
2.2758
1.9452
1.6151
0.9549
0.6248
0.2945
-4.8597
-5.0889
[rel]
[ppm]

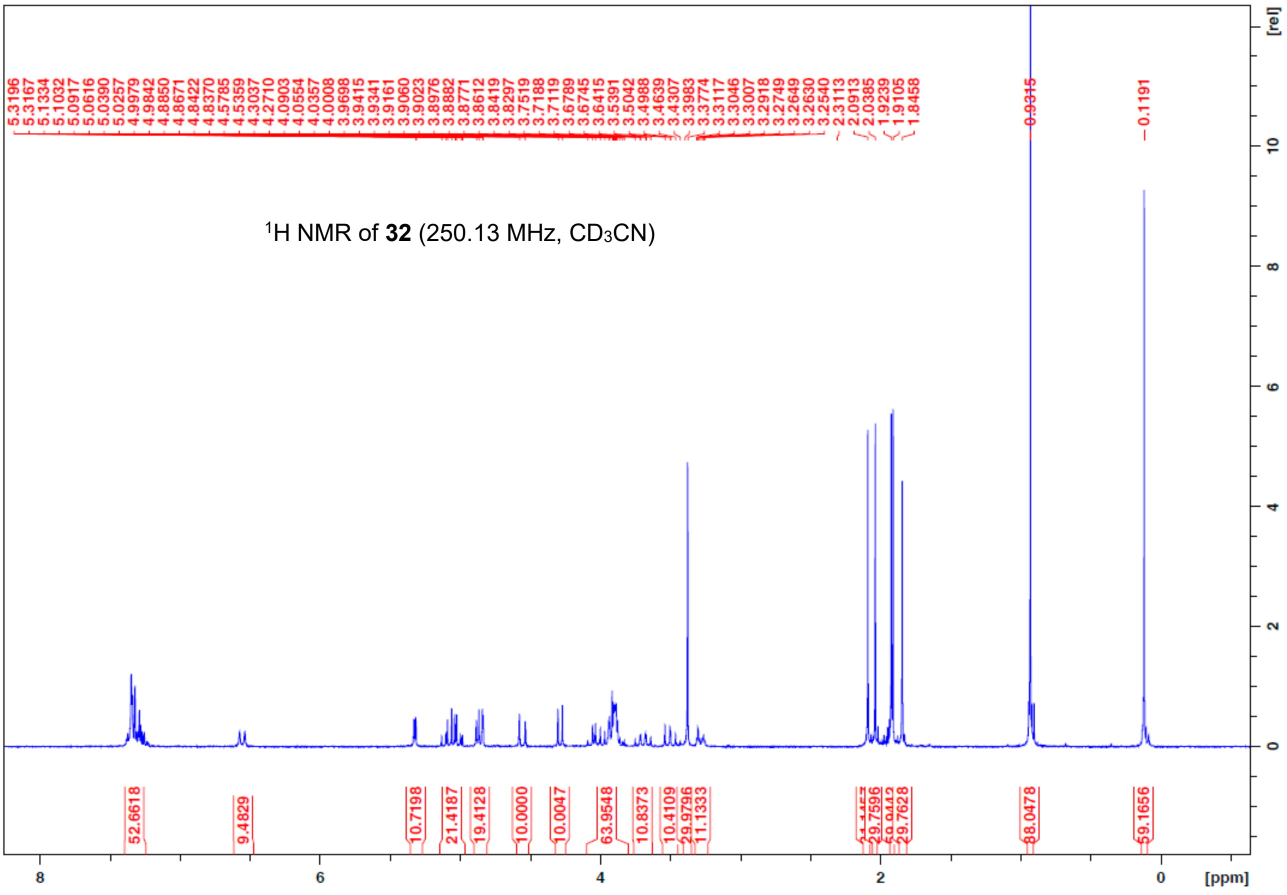

1H NMR of 32 (250.13 MHz, CD3CN)
5.3196
5.3167
5.1334
5.1032
5.0917
5.0616
5.0390
5.0257
4.9979
4.9842
4.8850
4.8671
4.8422
4.8370
4.5785
4.5359
4.3037
4.2710
4.0903
4.0554
4.0357
4.0008
3.9698
3.9415
3.9341
3.9161
3.9060
3.9023
3.8976
3.8882
3.8771
3.8612
3.8419
3.8297
3.7519
3.7188
3.7119
3.6789
3.6745
3.6415
3.5391
3.5042
3.4988
3.4639
3.4307
3.3983
3.3774
3.3117
3.3046
3.3007
3.2918
3.2749
3.2649
3.2630
3.2540
2.3113
2.0913
2.0385
1.9239
1.9105
1.8458
0.9315
0.1191
52.6618
9.4829
10.7198
21.4187
19.4128
10.0000
10.0047
63.9548
10.8373
10.4109
29.9796
11.1333
29.7596
29.7628
88.0478
59.1656
[rel]
[ppm]

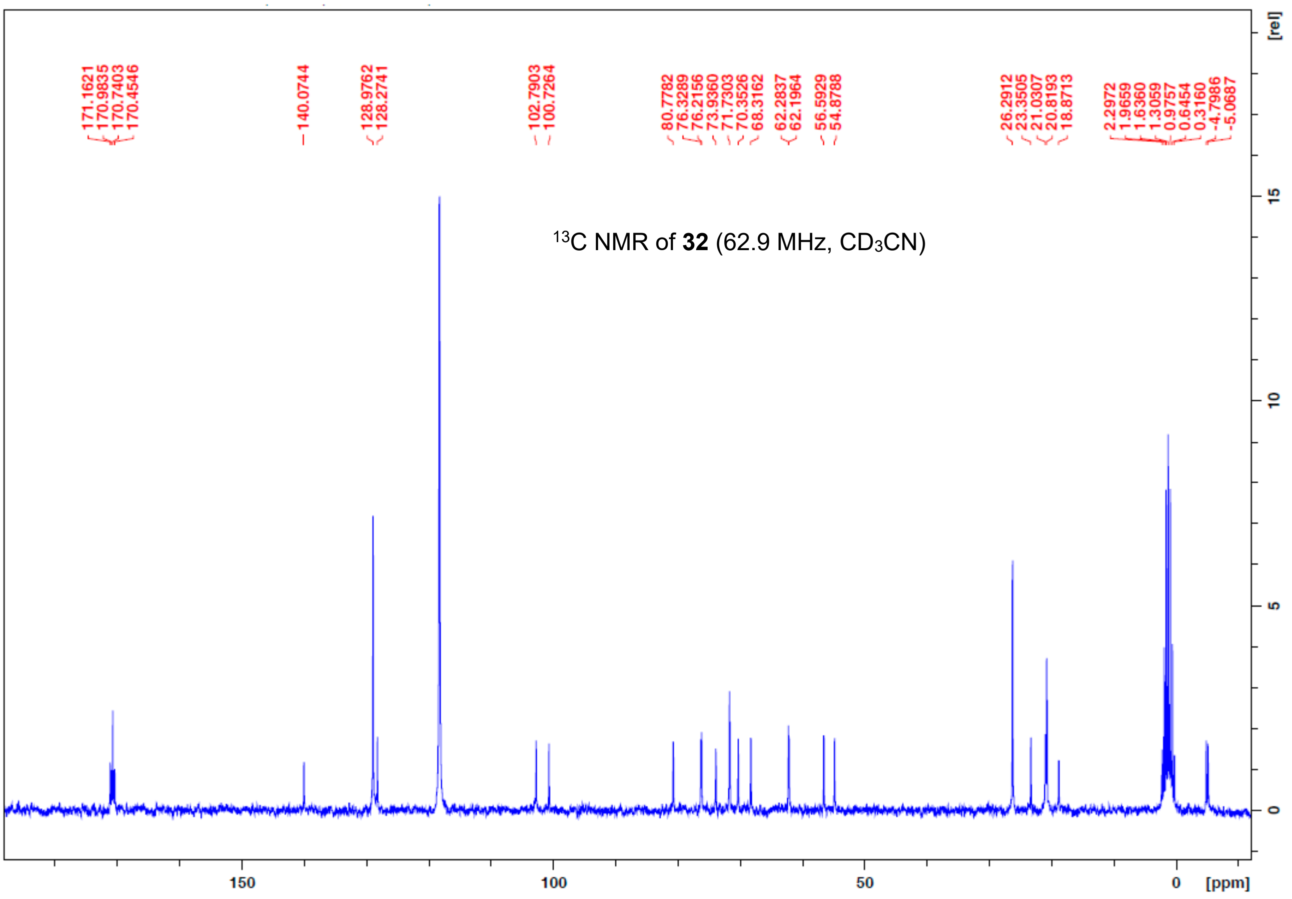

^{13}C NMR of **32** (62.9 MHz, CD3CN)

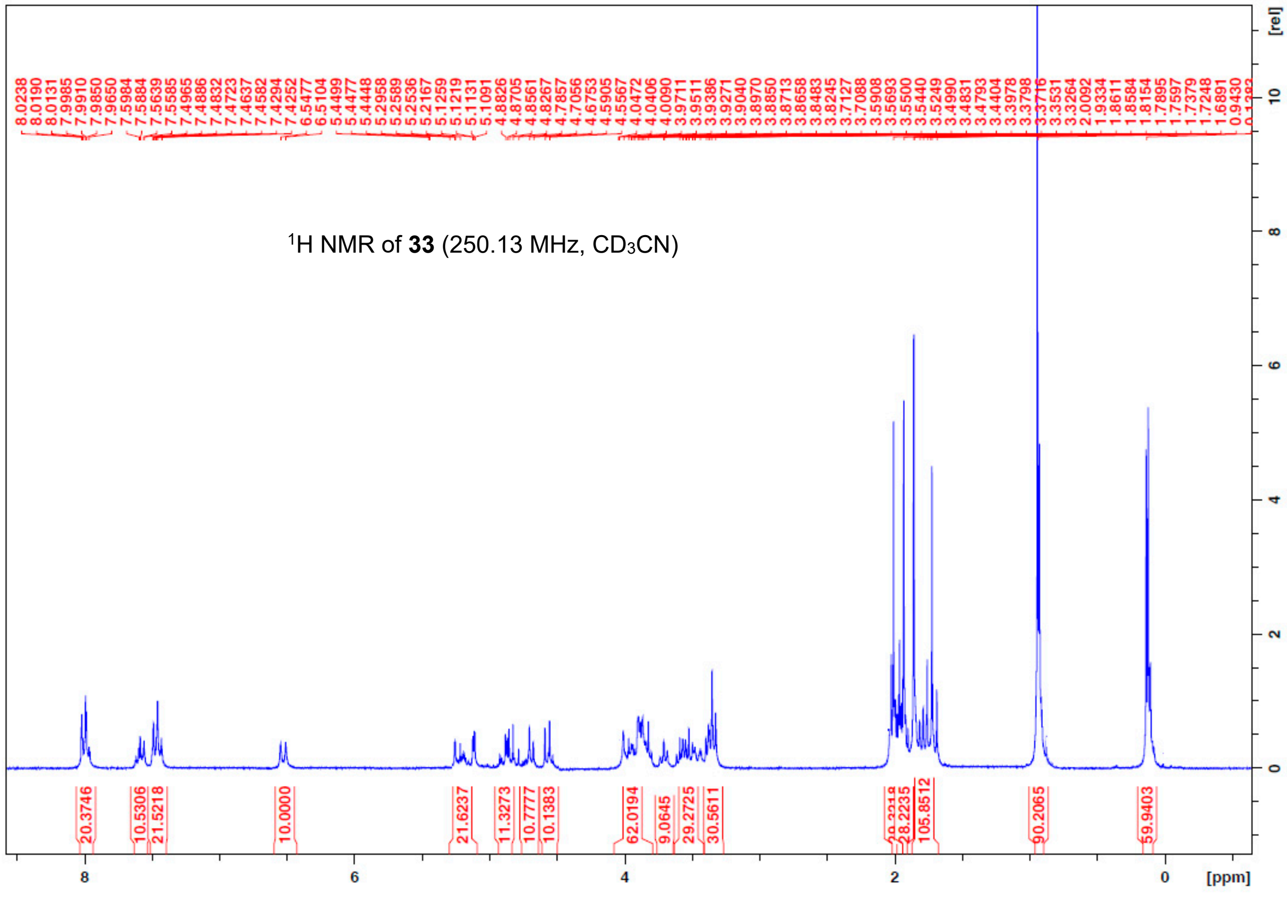

^{1}H NMR of **33** (250.13 MHz, CD3CN)

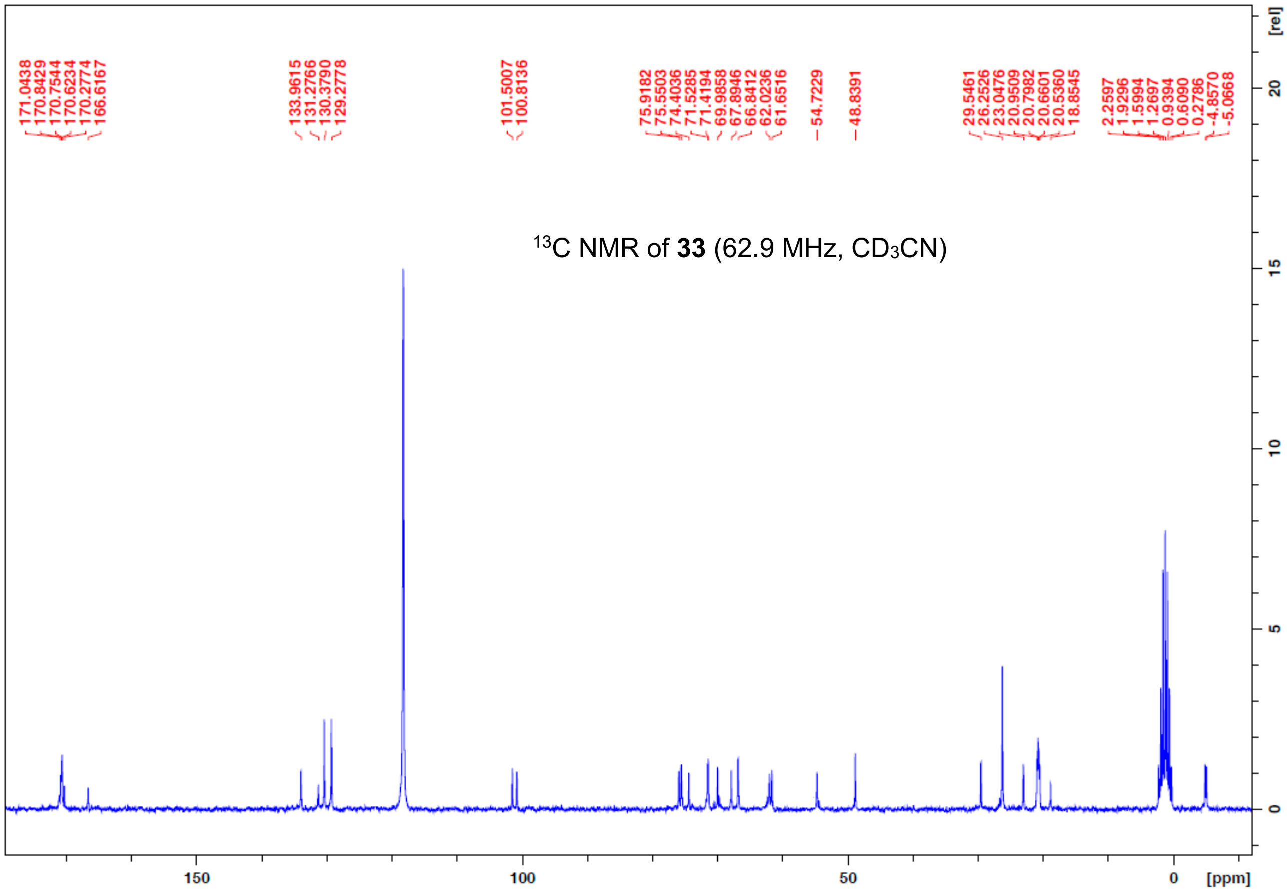
^{13}C NMR of **33** (62.9 MHz, CD_3CN)
171.0438
170.8429
170.7544
170.6234
170.2774
166.6167
133.9615
131.2766
130.3790
129.2778
101.5007
100.8136
75.9182
75.5503
74.4036
71.5285
71.4194
69.9858
67.8946
66.8412
62.0236
61.6516
54.7229
48.8391
29.5461
26.2526
23.0476
20.9509
20.7982
20.6601
20.5360
18.8545
2.2597
1.9296
1.5994
1.2697
0.9394
0.6090
0.2786
-4.8570
-5.0668
150
100
50
0
[ppm]
[rel]
20
15
10
5
0

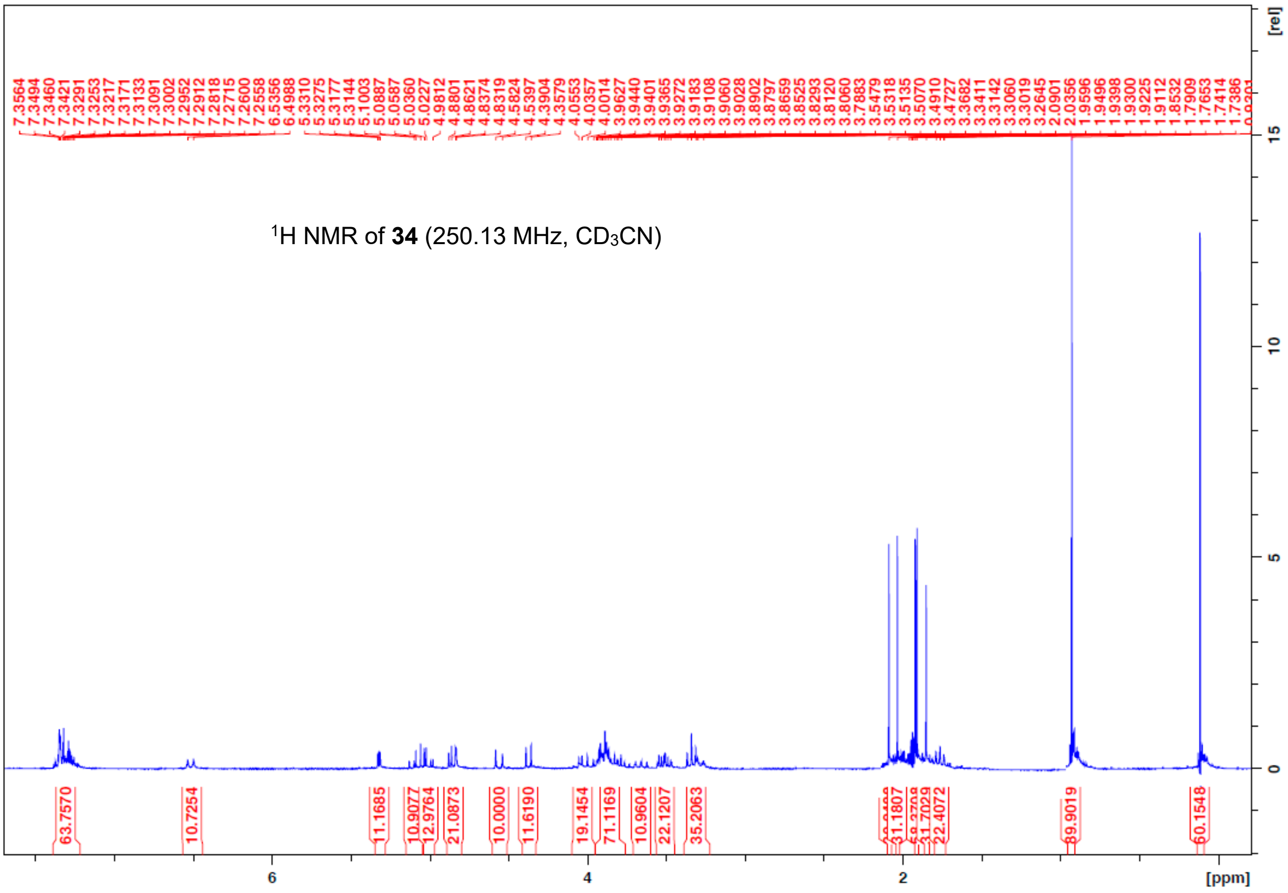
^{1}H NMR of **34** (250.13 MHz, CD_3CN)
6
4
2
[ppm]
[rel]
15
10
5
0

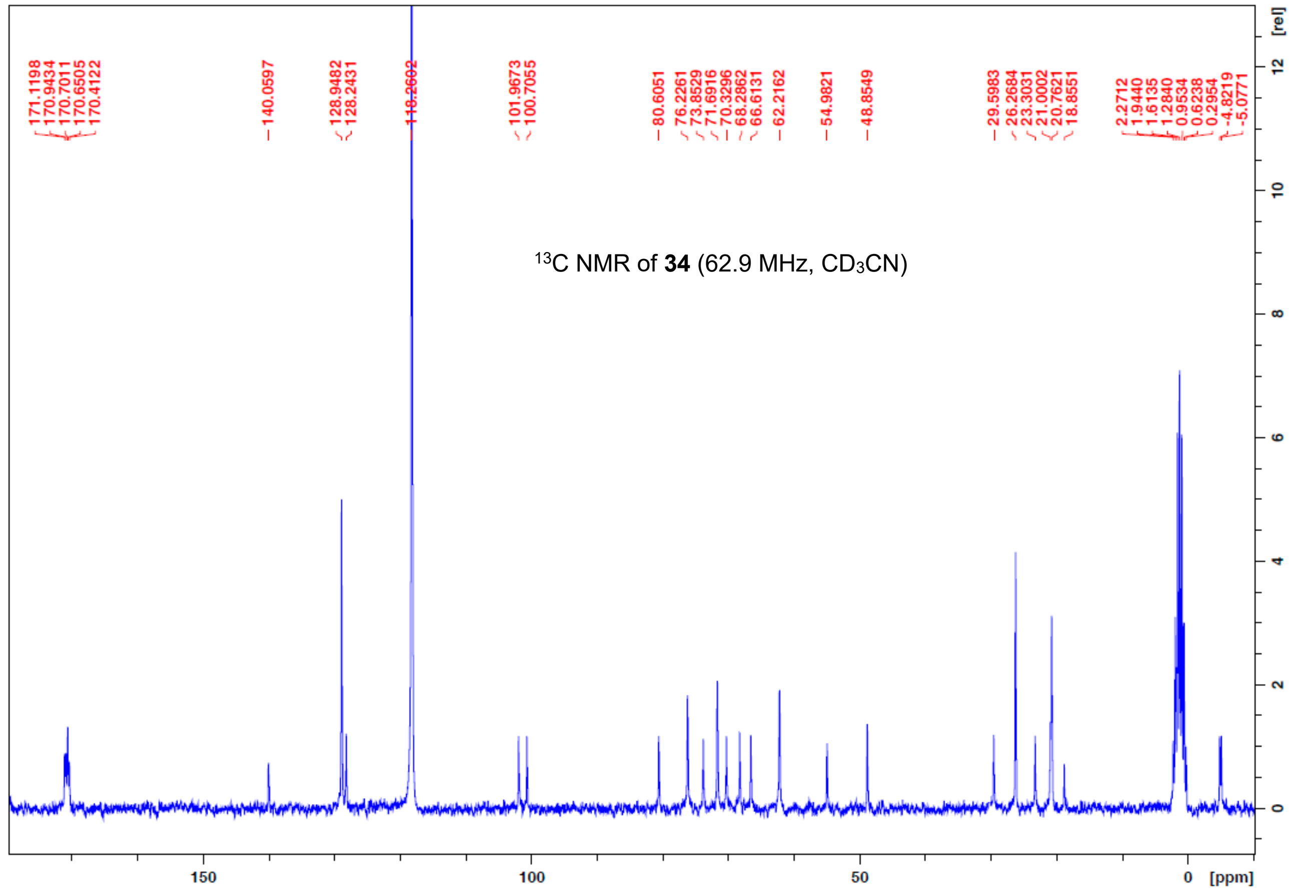
^{13}C NMR of **34** (62.9 MHz, CD_3CN)

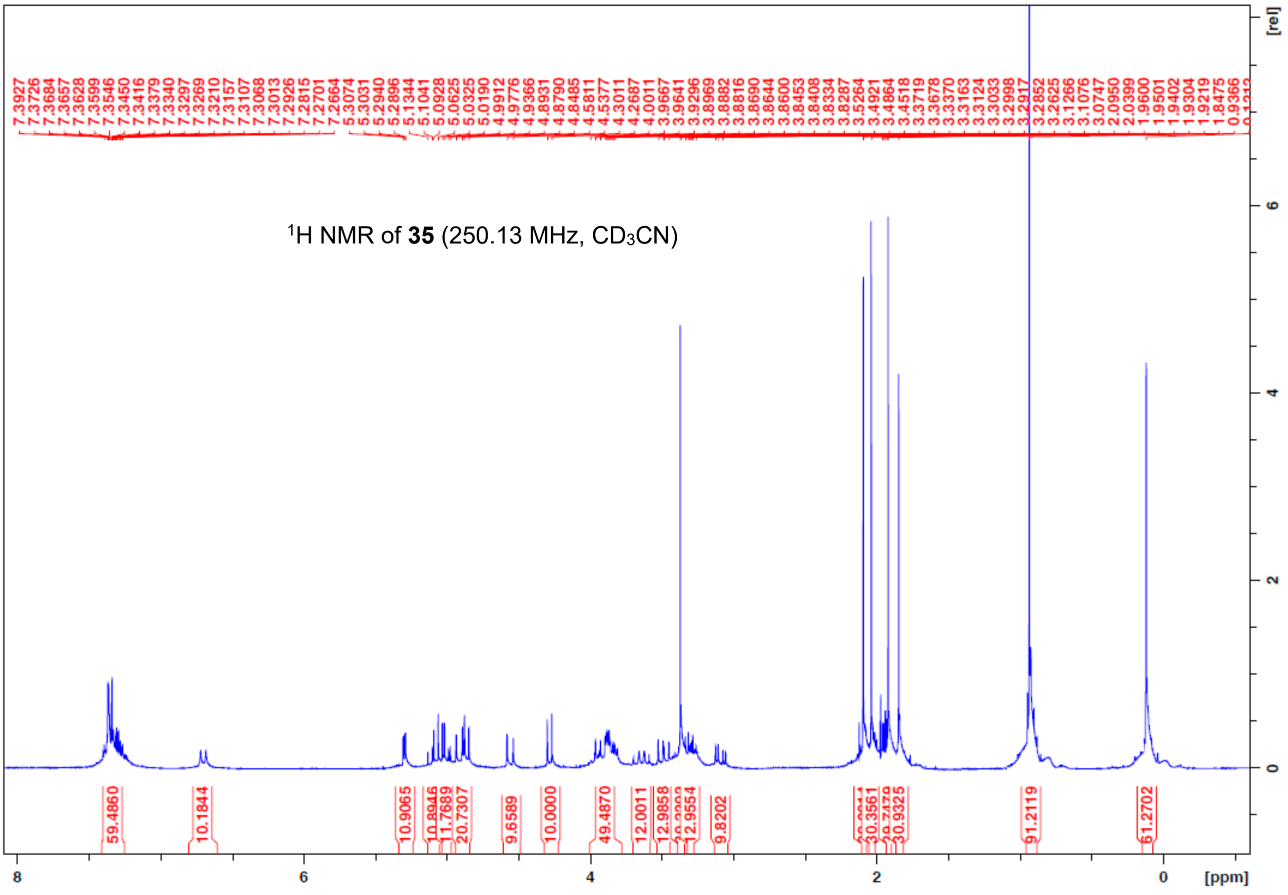
^{1}H NMR of **35** (250.13 MHz, CD_3CN)

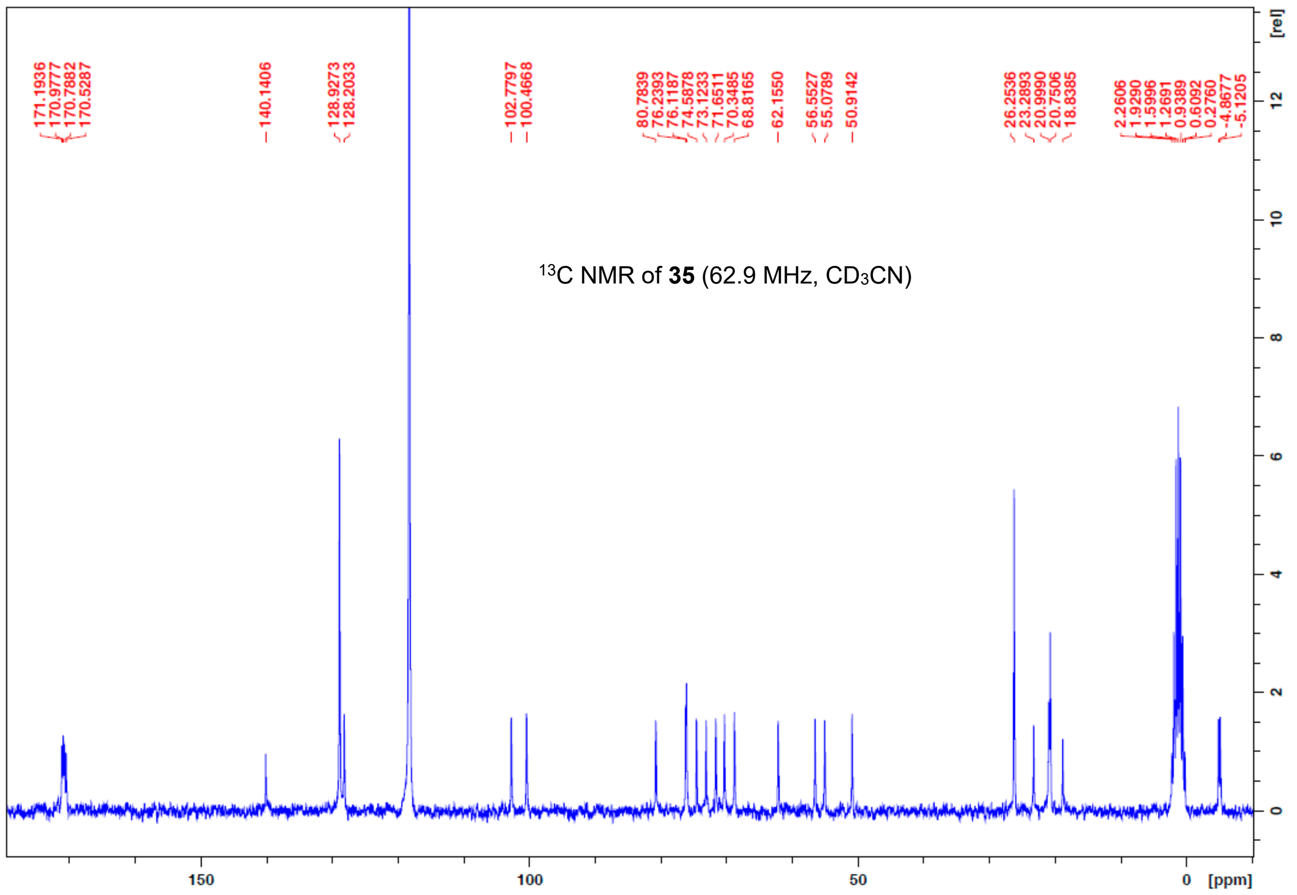

^{13}C NMR of **35** (62.9 MHz, CD_3CN)
[rel]
[ppm]

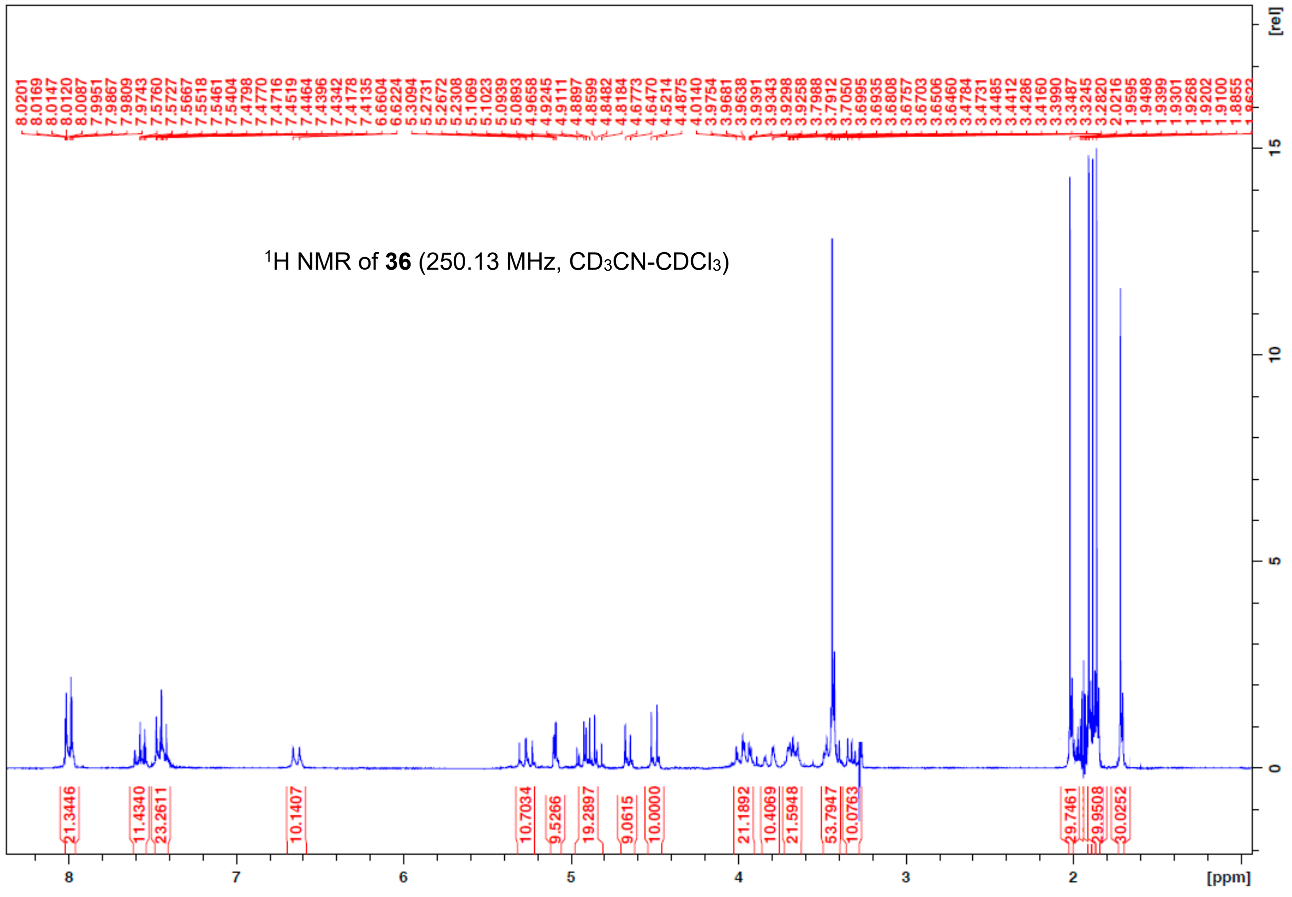

^{1}H NMR of **36** (250.13 MHz, CD_3CN-$CDCl_3$)
[rel]
[ppm]

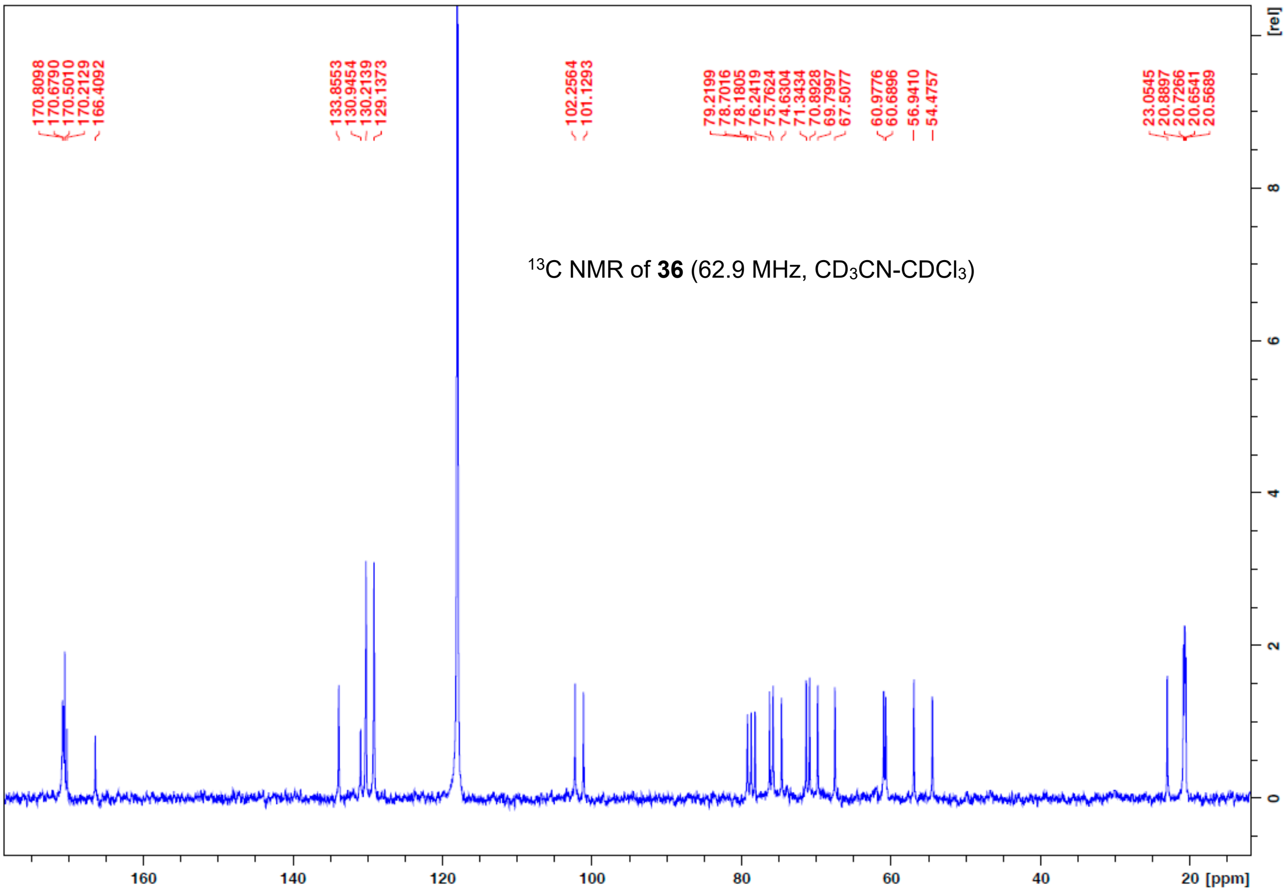
13C NMR of 36 (62.9 MHz, CD3CN-CDCl3)
170.8098
170.6790
170.5010
170.2129
166.4092
133.8553
130.9454
130.2139
129.1373
102.2564
101.1293
79.2199
78.7016
78.1805
76.2419
75.7624
74.6304
71.3434
70.8928
69.7997
67.5077
60.9776
60.6896
56.9410
54.4757
23.0545
20.8897
20.7266
20.6541
20.5689
[rel]
160
140
120
100
80
60
40
20 [ppm]

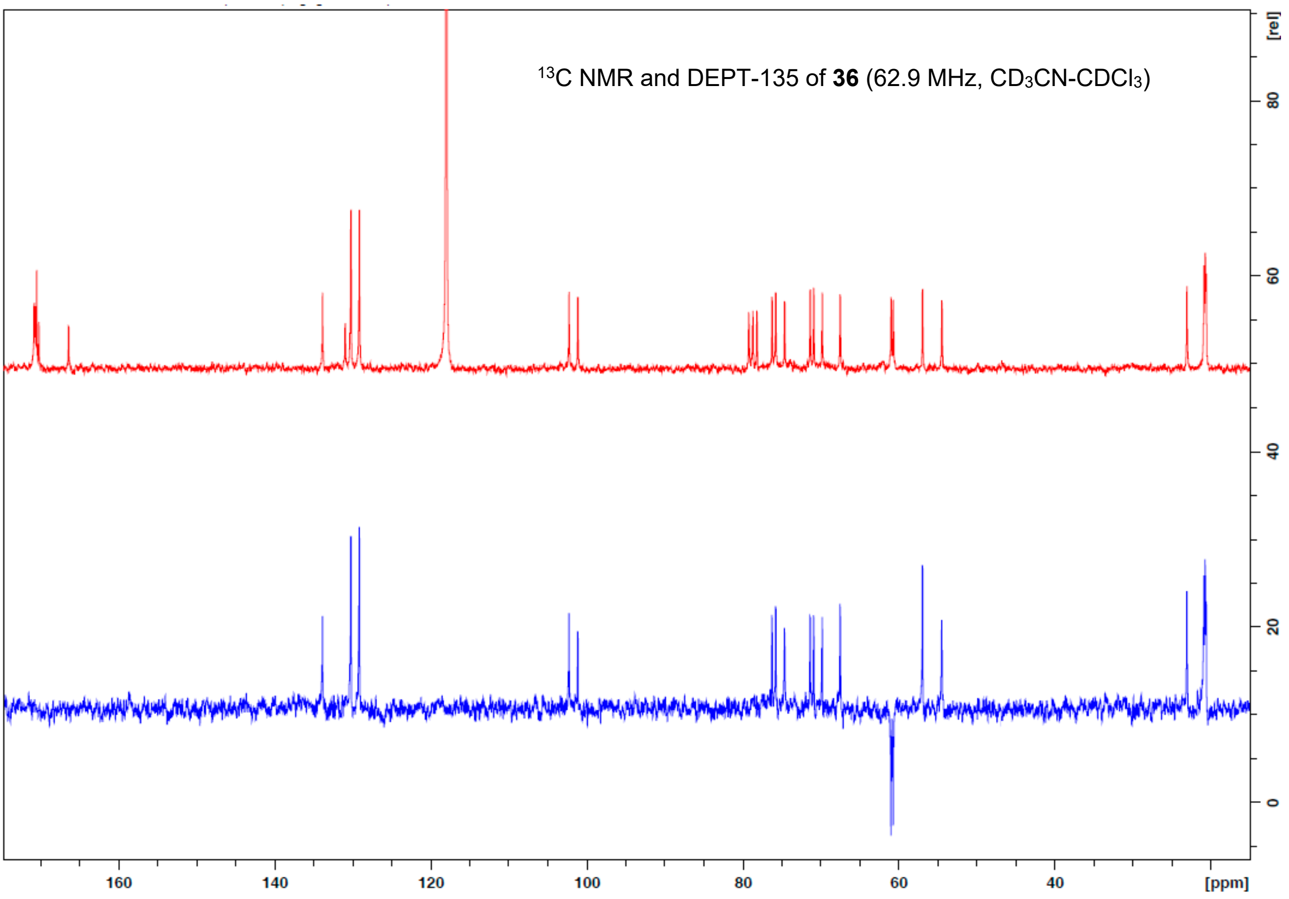
13C NMR and DEPT-135 of 36 (62.9 MHz, CD3CN-CDCl3)
[rel]
160
140
120
100
80
60
40
[ppm]

HETCOR spectra of **36** (62.9 MHz, CD_3CN-$CDCl_3$)

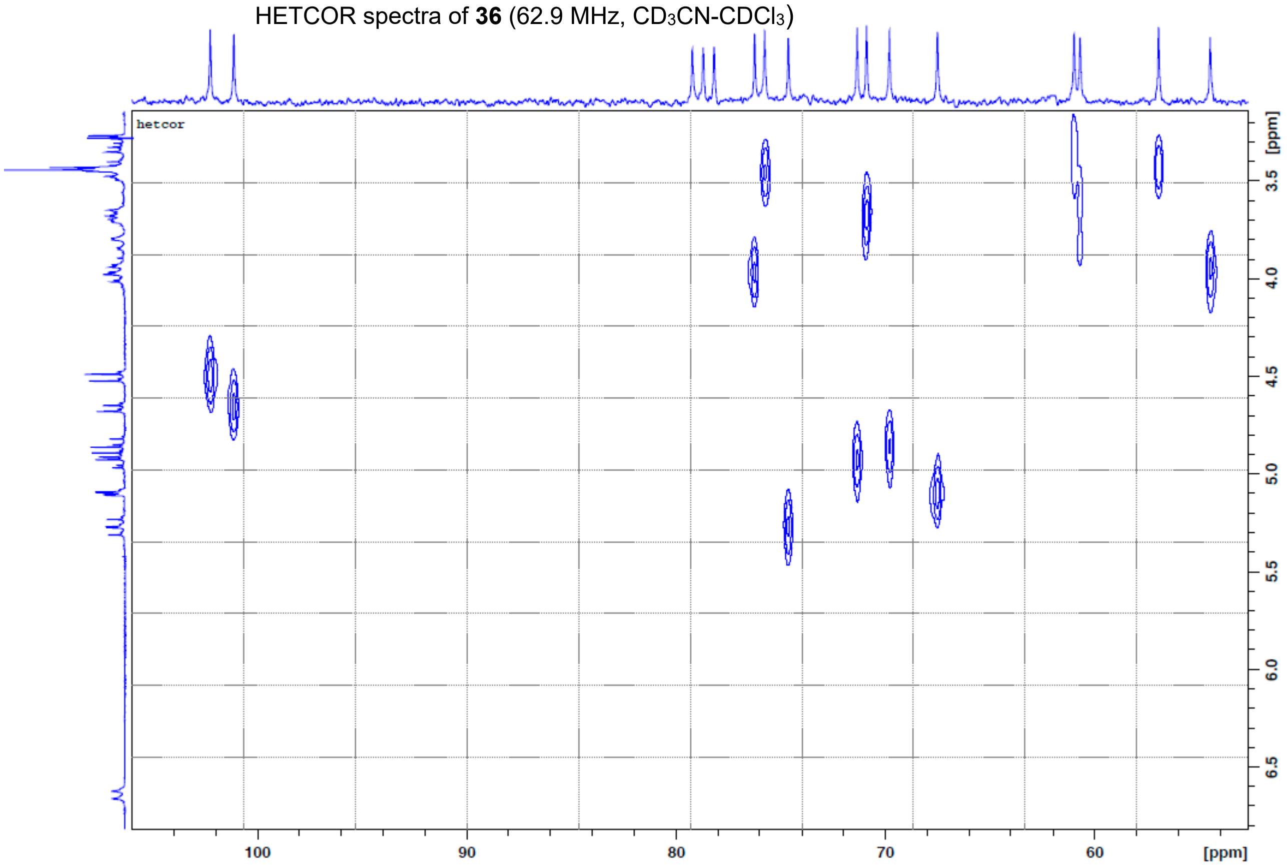

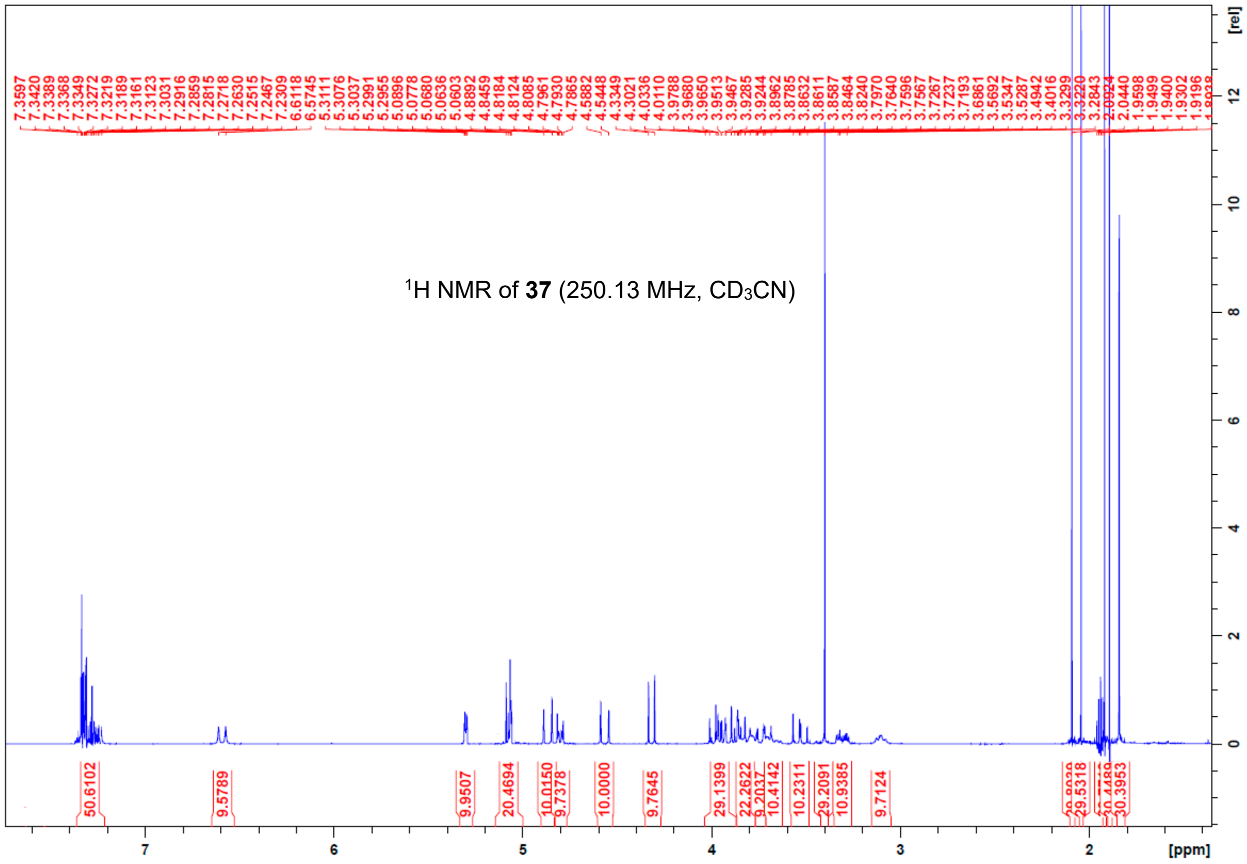

^{1}H NMR of **37** (250.13 MHz, CD_3CN)

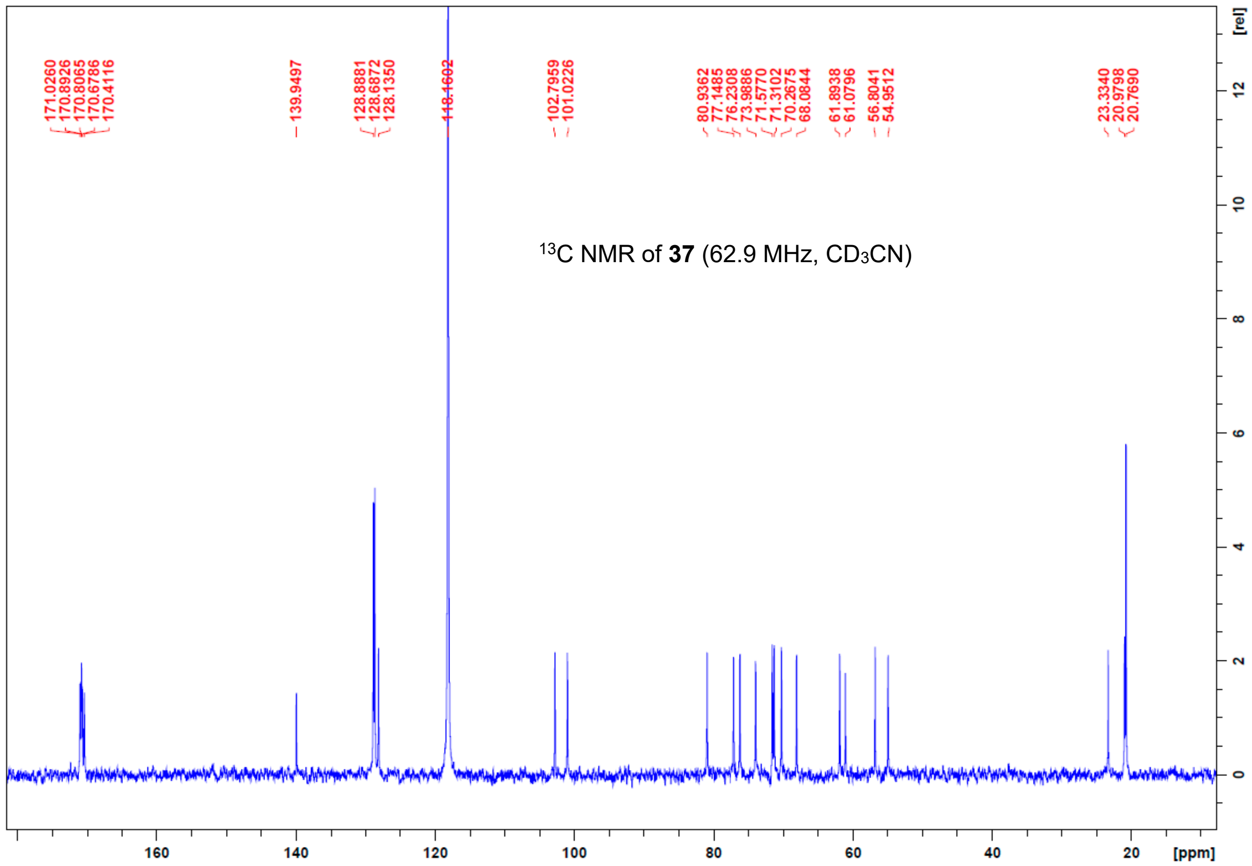

^{13}C NMR of **37** (62.9 MHz, CD_3CN)

COSY spectra of **37** (250.13 MHz, CD_3CN)

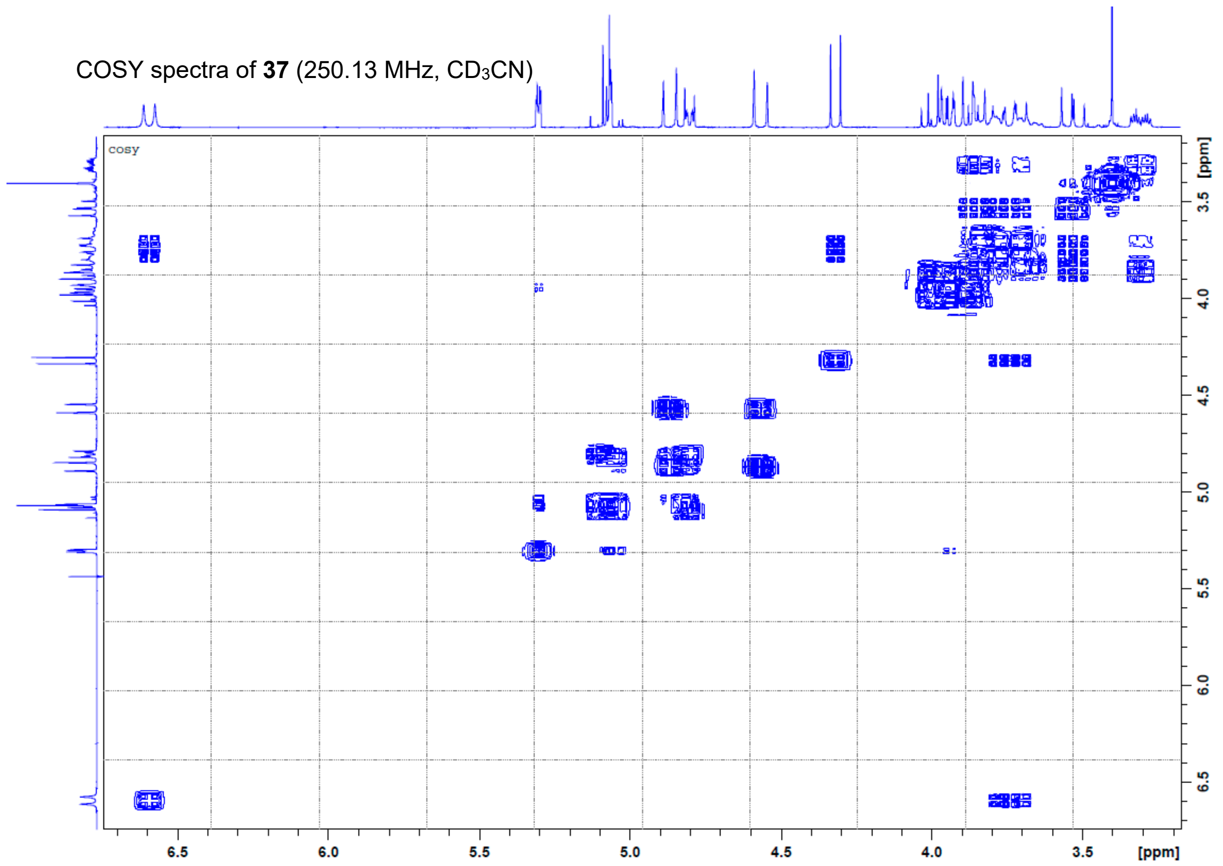

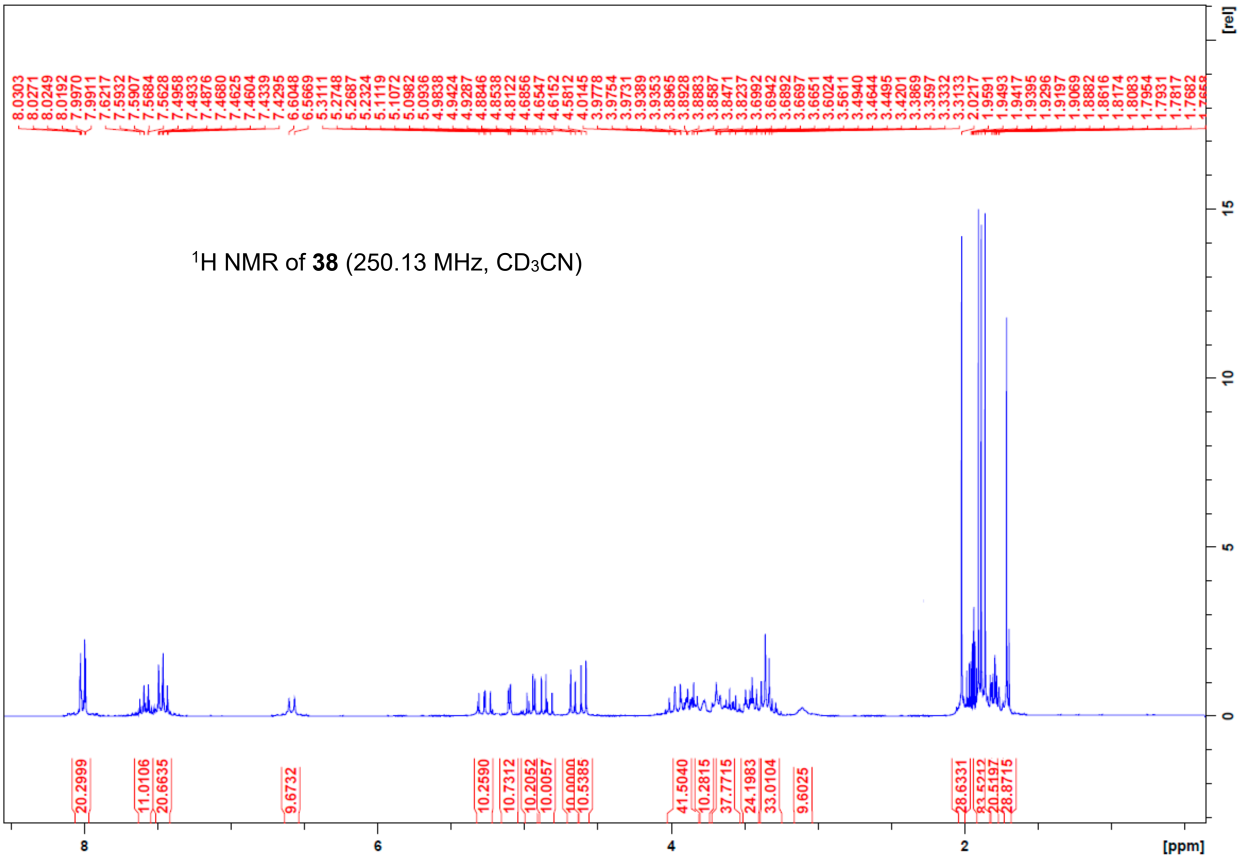
1H NMR of 38 (250.13 MHz, CD3CN)
[ppm]
[rel]

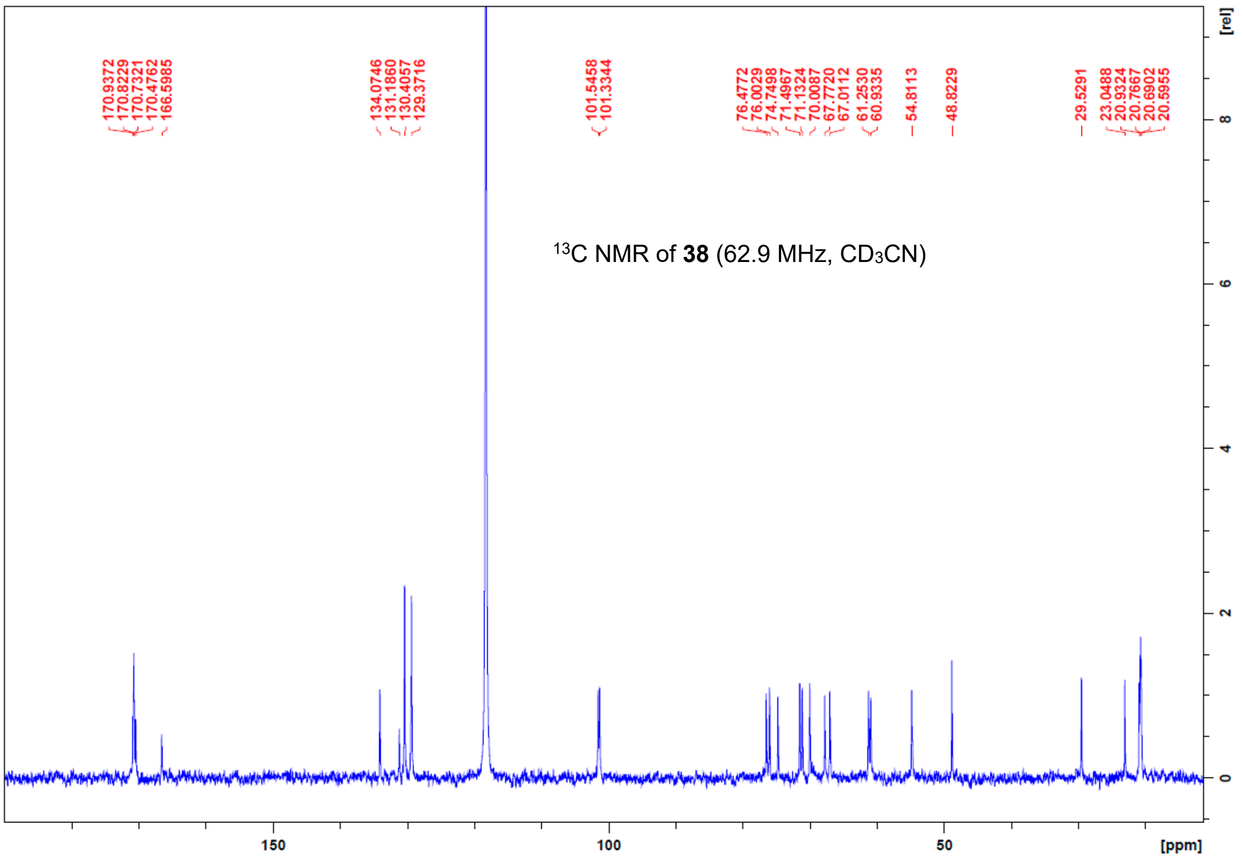
13C NMR of 38 (62.9 MHz, CD3CN)
170.9372
170.8229
170.7321
170.4762
166.5985
134.0746
131.1860
130.4057
129.3716
101.5458
101.3344
76.4772
76.0029
74.7498
71.4967
71.1324
70.0087
67.7720
67.0112
61.2530
60.9335
54.8113
48.8229
29.5291
23.0488
20.9324
20.7667
20.6902
20.5955
[ppm]
[rel]

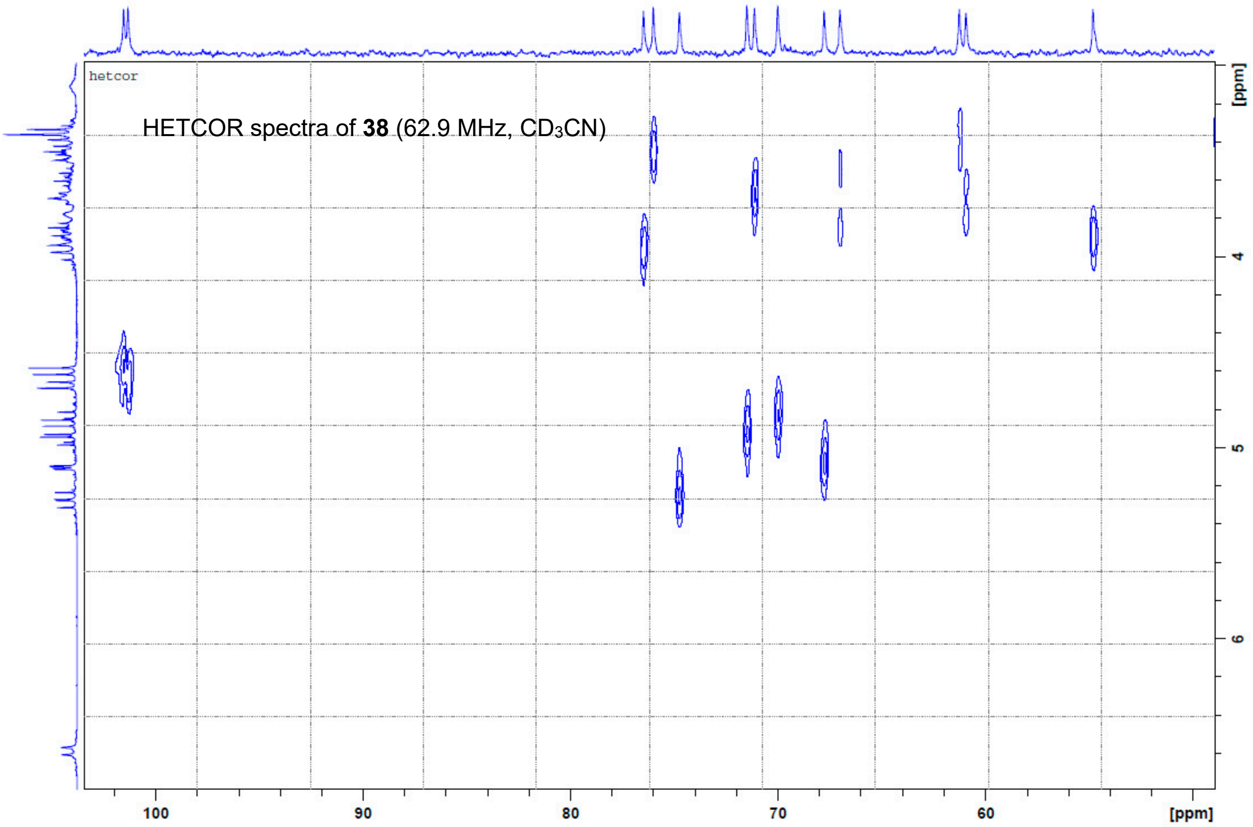
hetcor
HETCOR spectra of 38 (62.9 MHz, CD3CN)

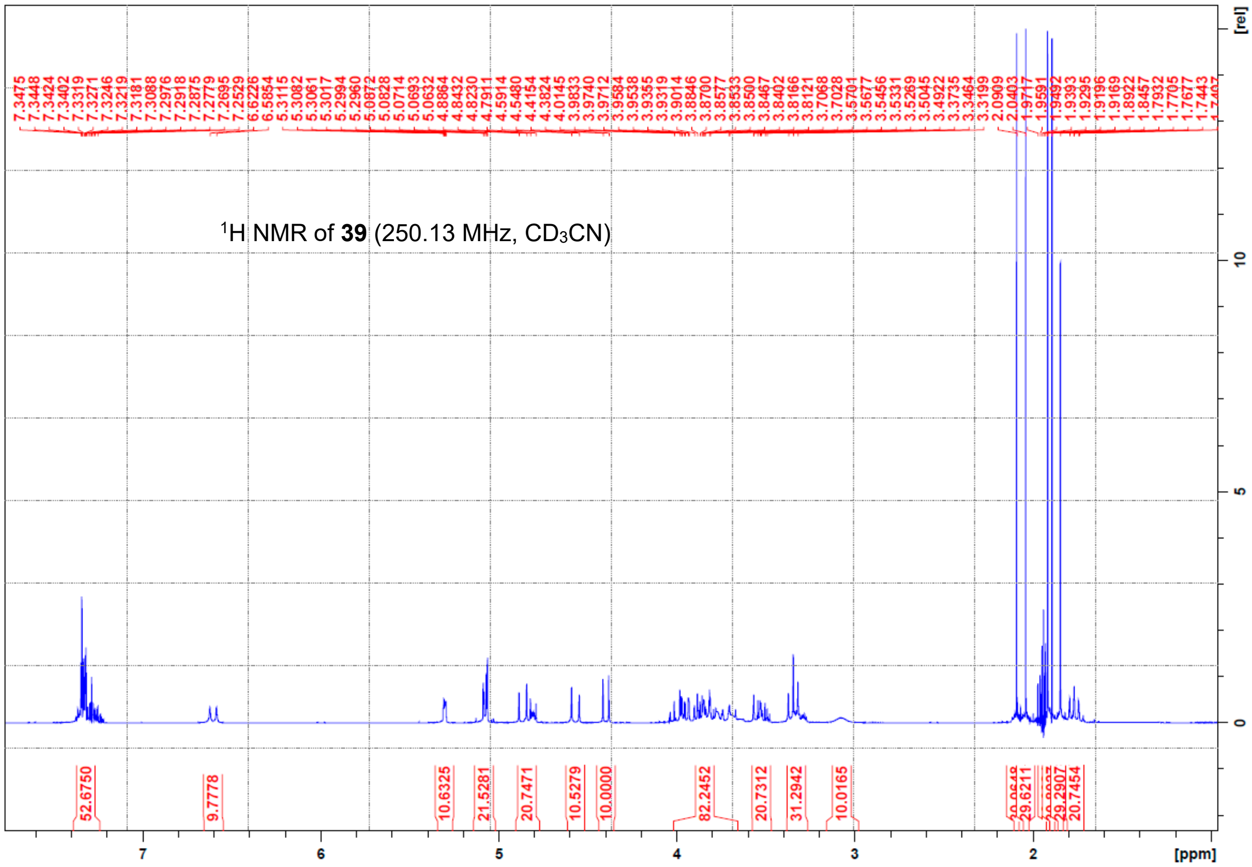
1H NMR of 39 (250.13 MHz, CD3CN)

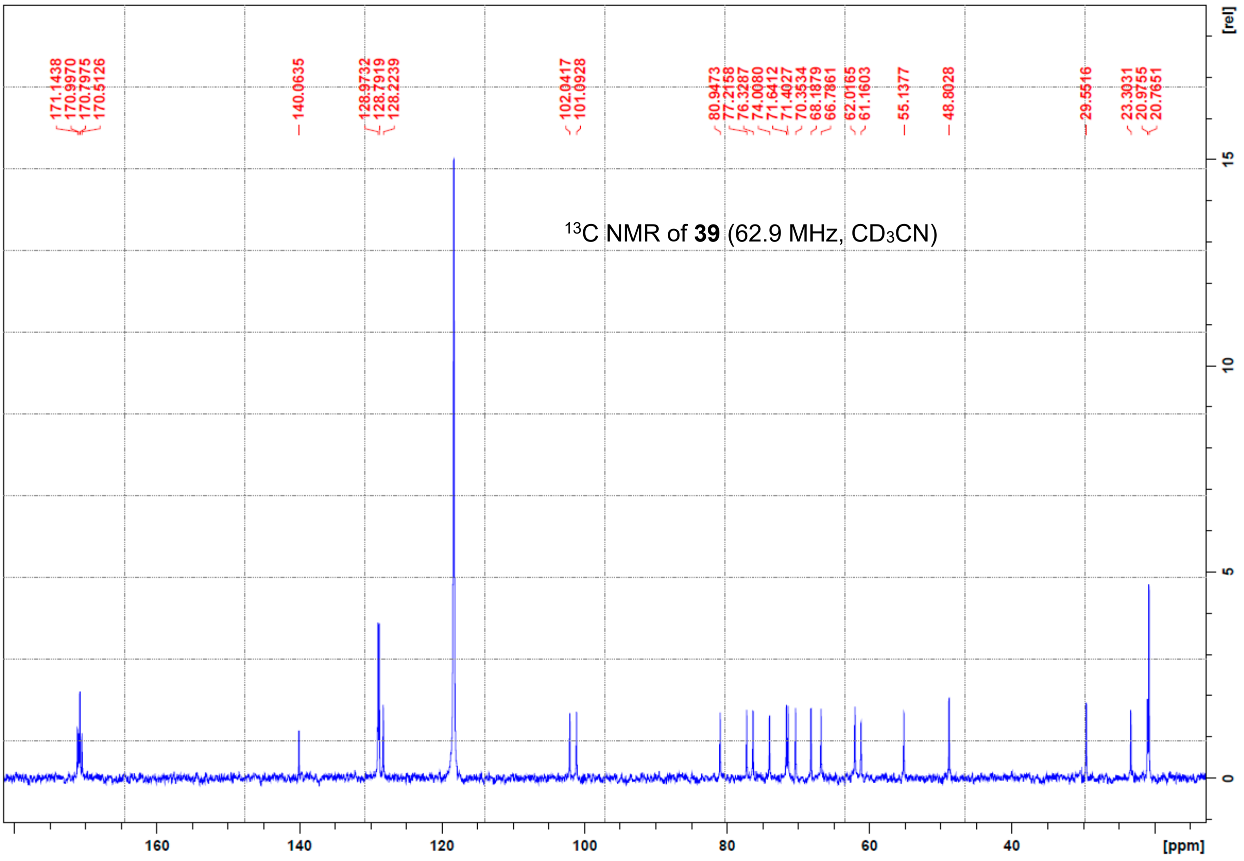
13C NMR of 39 (62.9 MHz, CD3CN)
[ppm]
[rel]

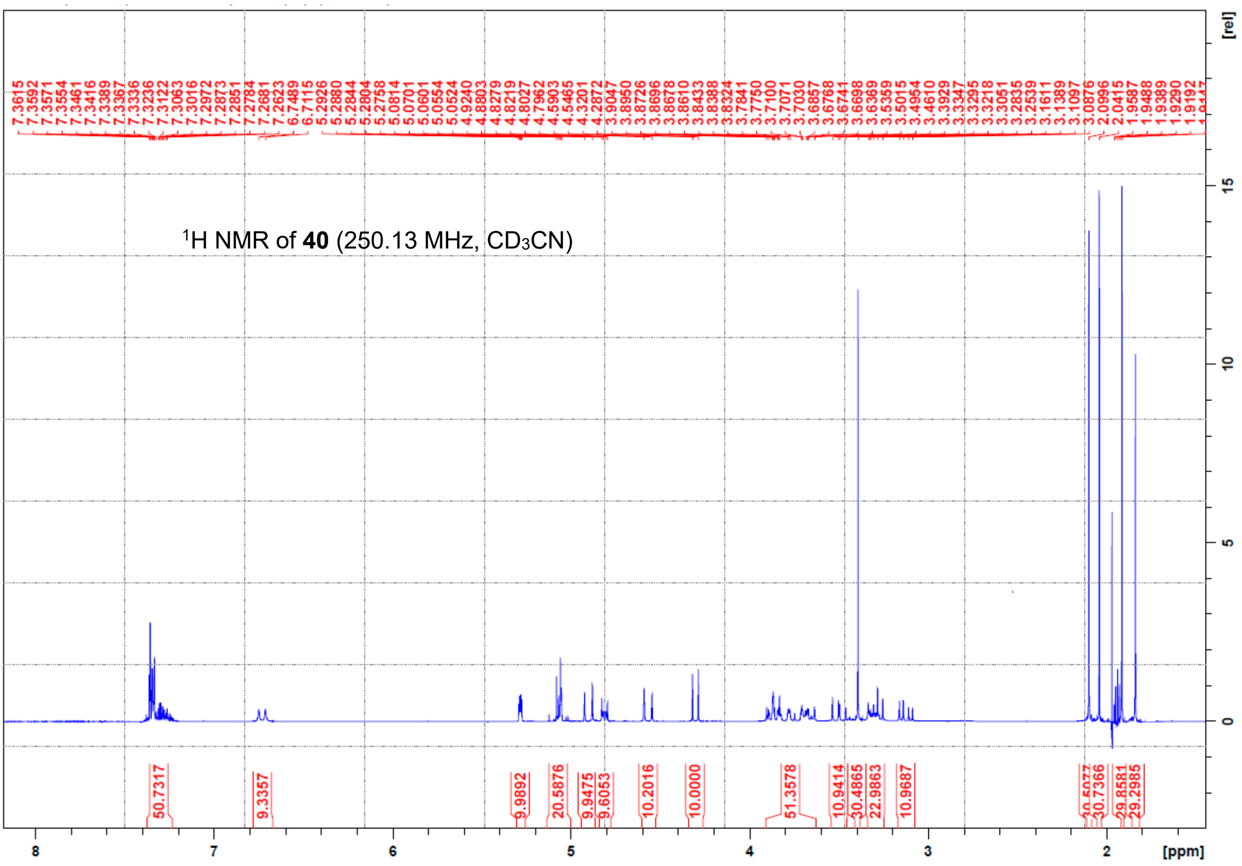
1H NMR of 40 (250.13 MHz, CD3CN)
[ppm]
[rel]

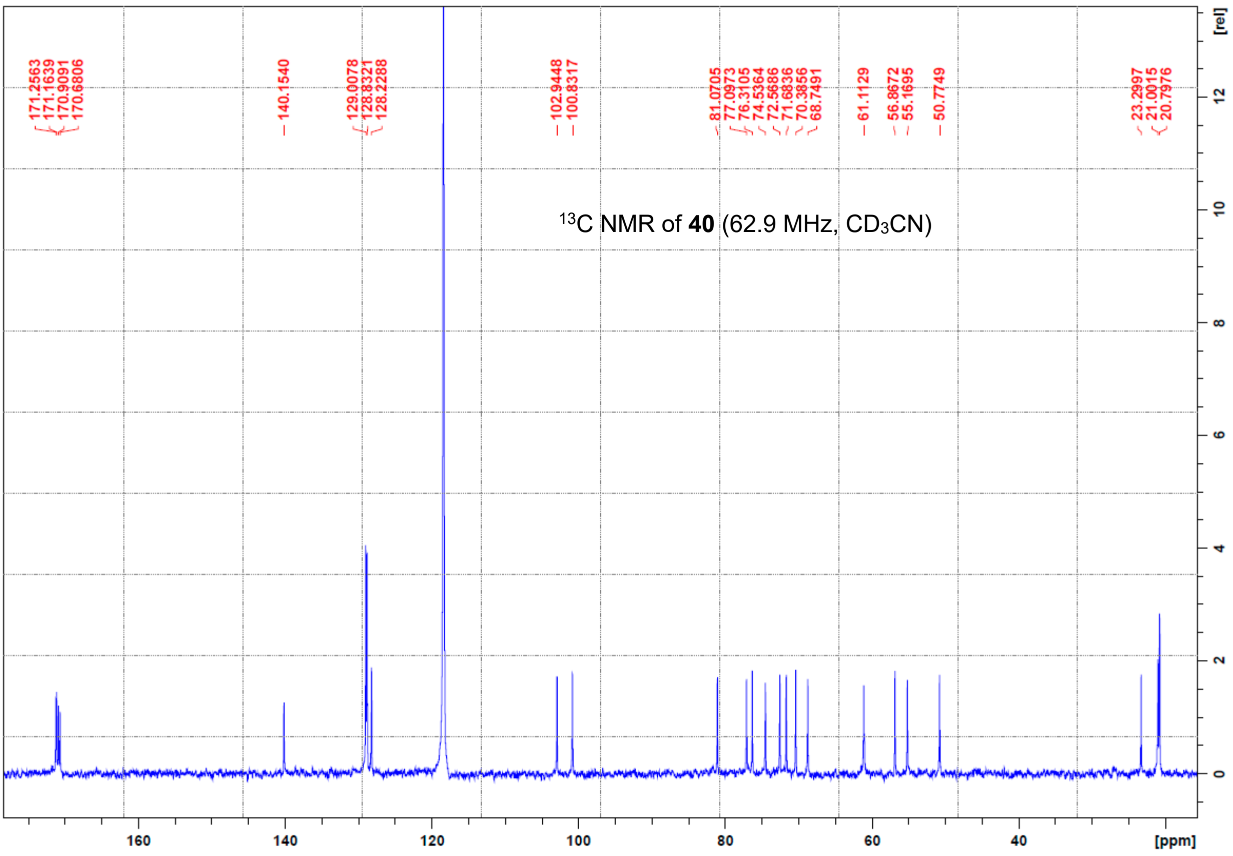
171.2563
171.1639
170.9091
170.6806
140.1540
129.0078
128.8321
128.2288
102.9448
100.8317
81.0705
77.0973
76.3105
74.5364
72.5686
71.6836
70.3856
68.7491
61.1129
56.8672
55.1695
50.7749
23.2997
21.0015
20.7976
13C NMR of 40 (62.9 MHz, CD3CN)
[rel]
[ppm]

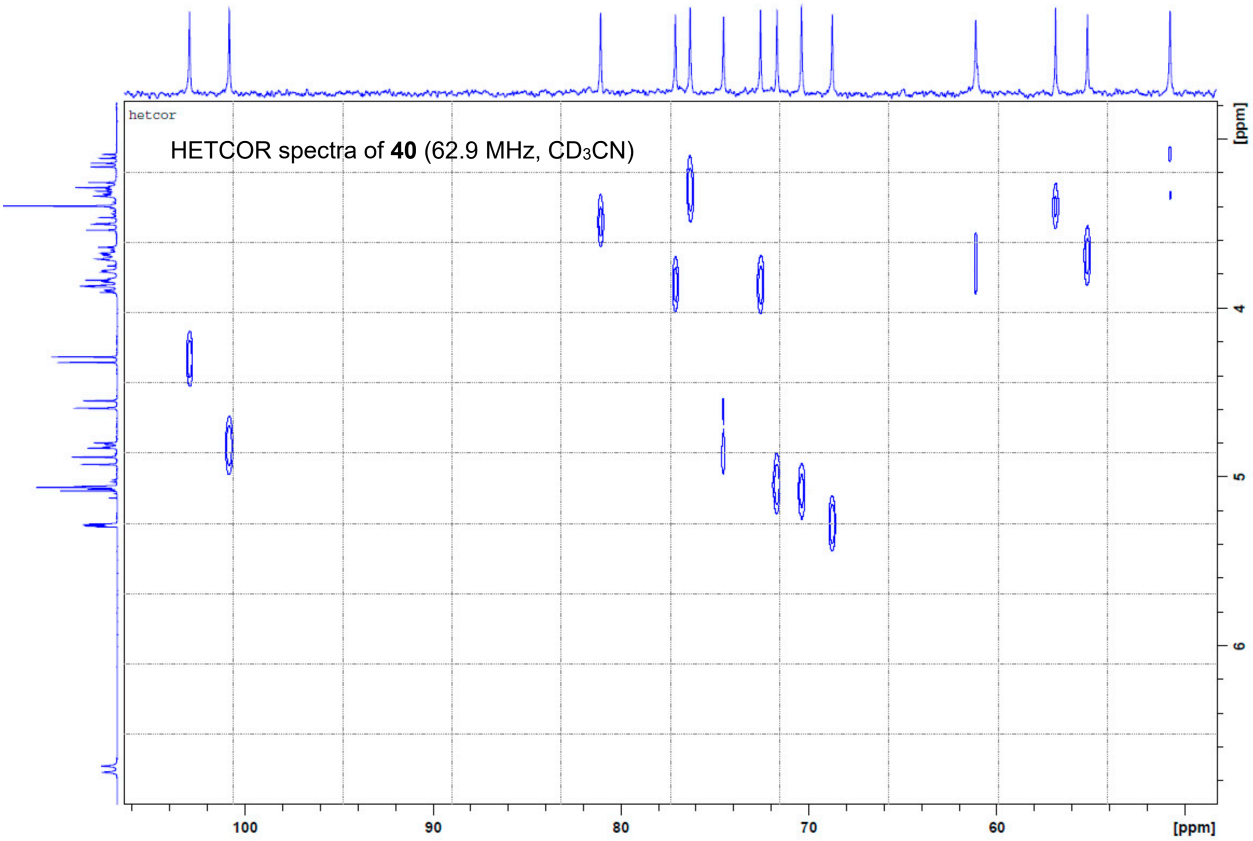
hetcor
HETCOR spectra of 40 (62.9 MHz, CD3CN)
[ppm]
[ppm]

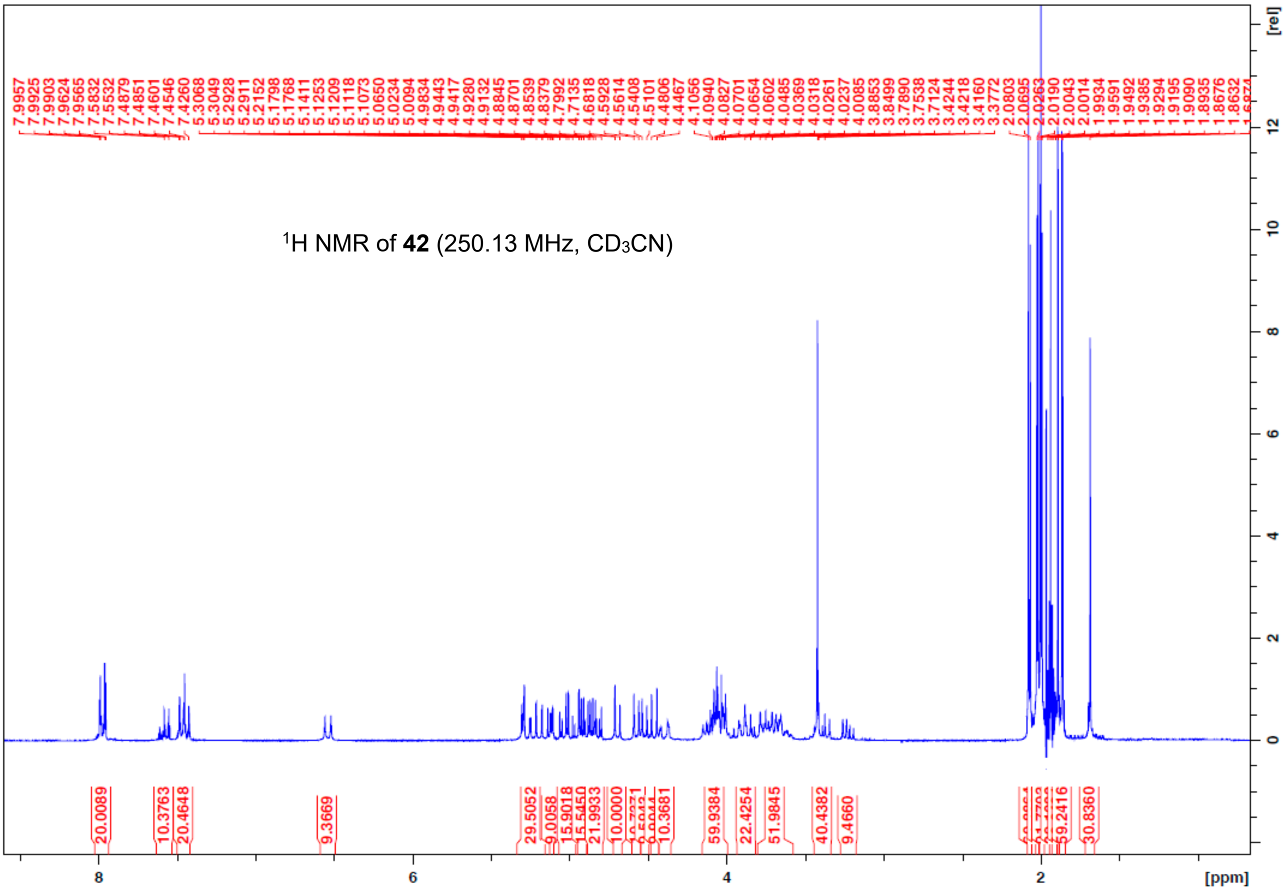

^{1}H NMR of **42** (250.13 MHz, CD_3CN)

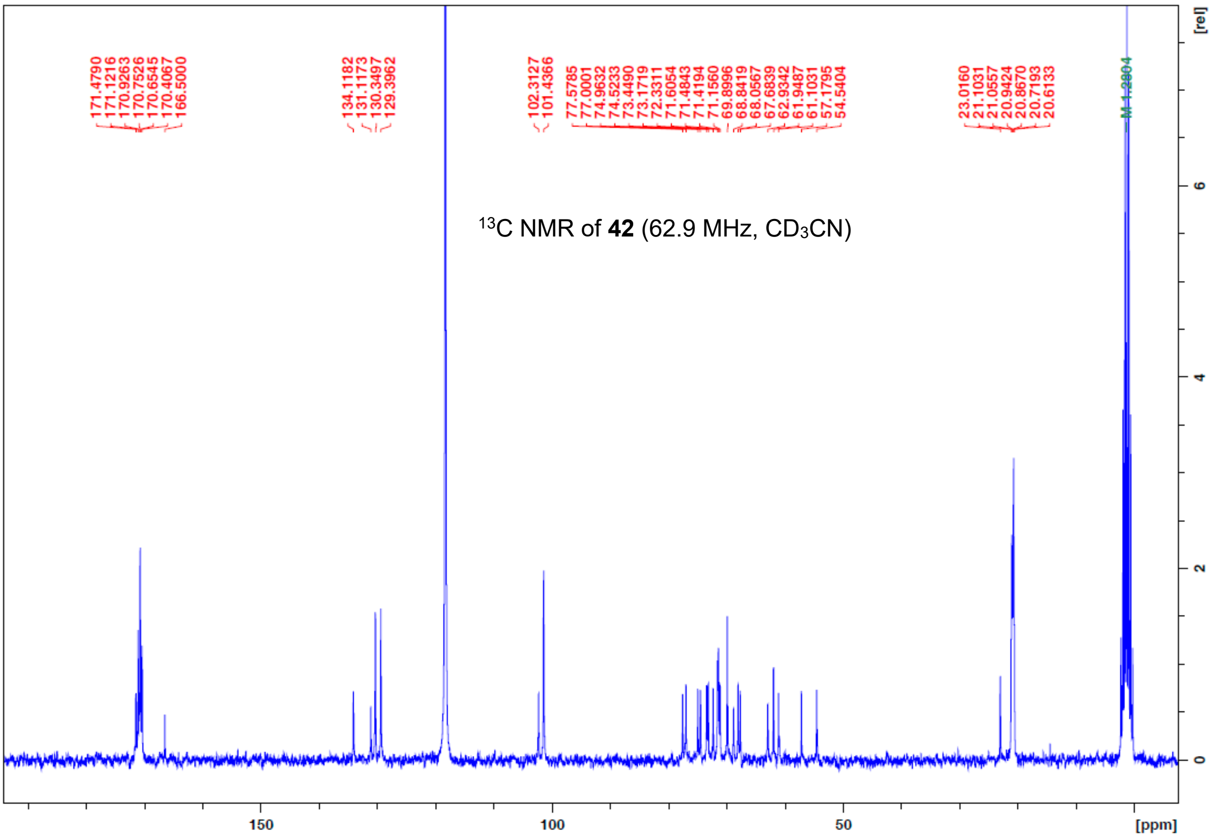

^{13}C NMR of **42** (62.9 MHz, CD_3CN)

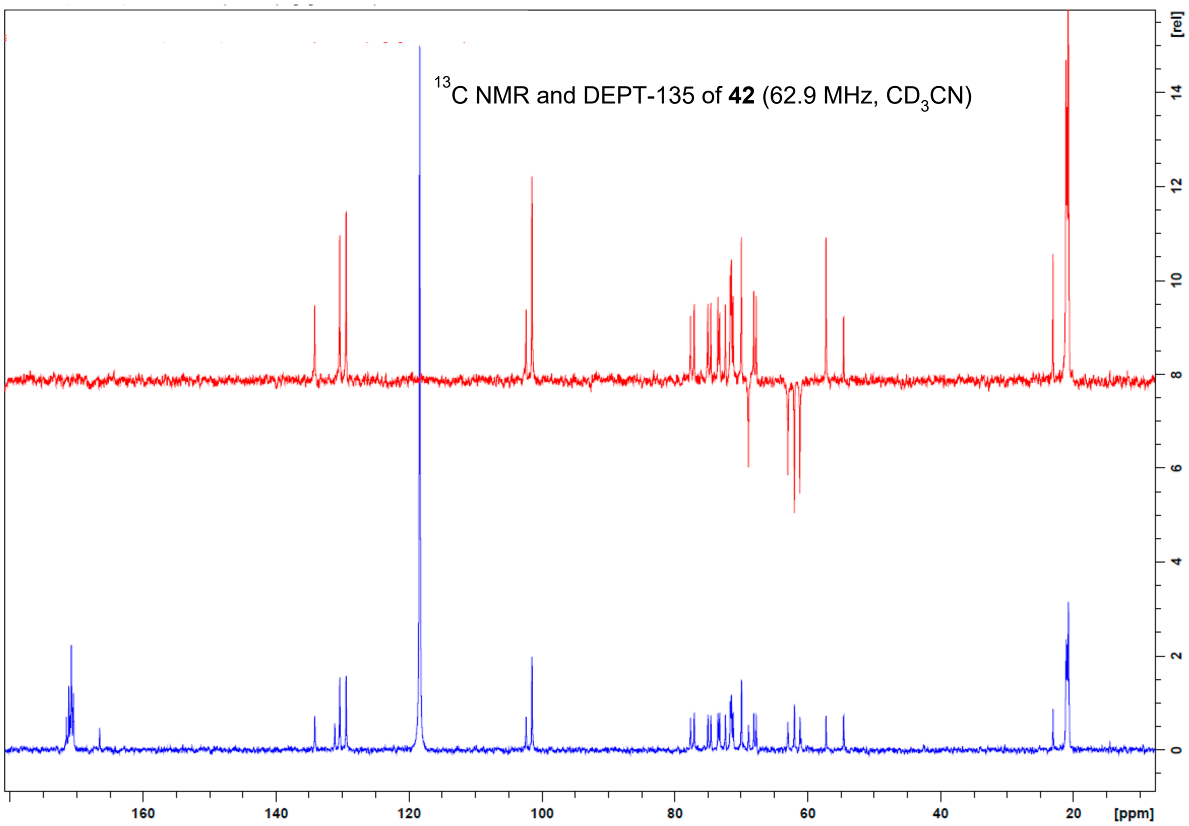

^{13}C NMR and DEPT-135 of **42** (62.9 MHz, CD_3CN)
[rel]
14
12
10
8
6
4
2
0
160
140
120
100
80
60
40
20
[ppm]

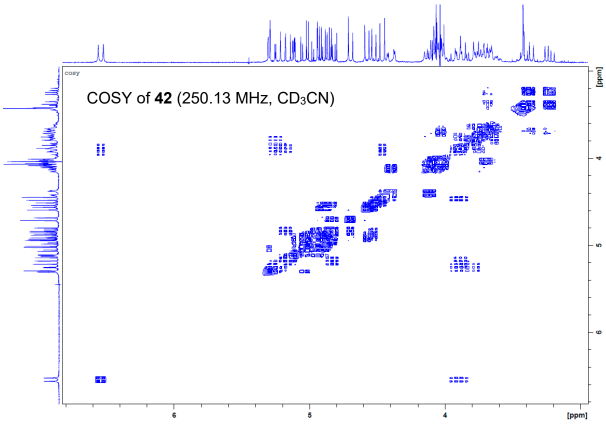

COSY of **42** (250.13 MHz, CD_3CN)
6
5
4
[ppm]

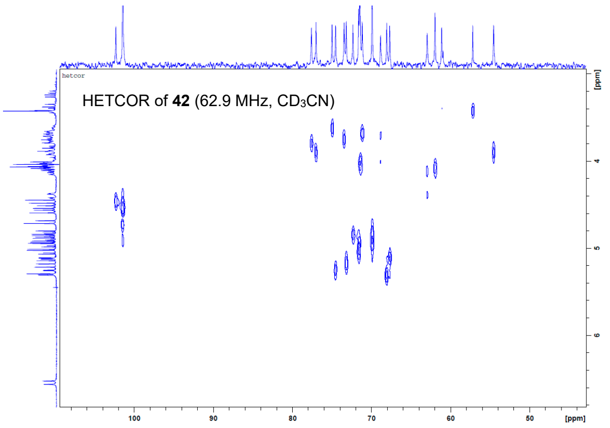

HETCOR of **42** (62.9 MHz, CD_3CN)
100
90
80
70
60
50
[ppm]

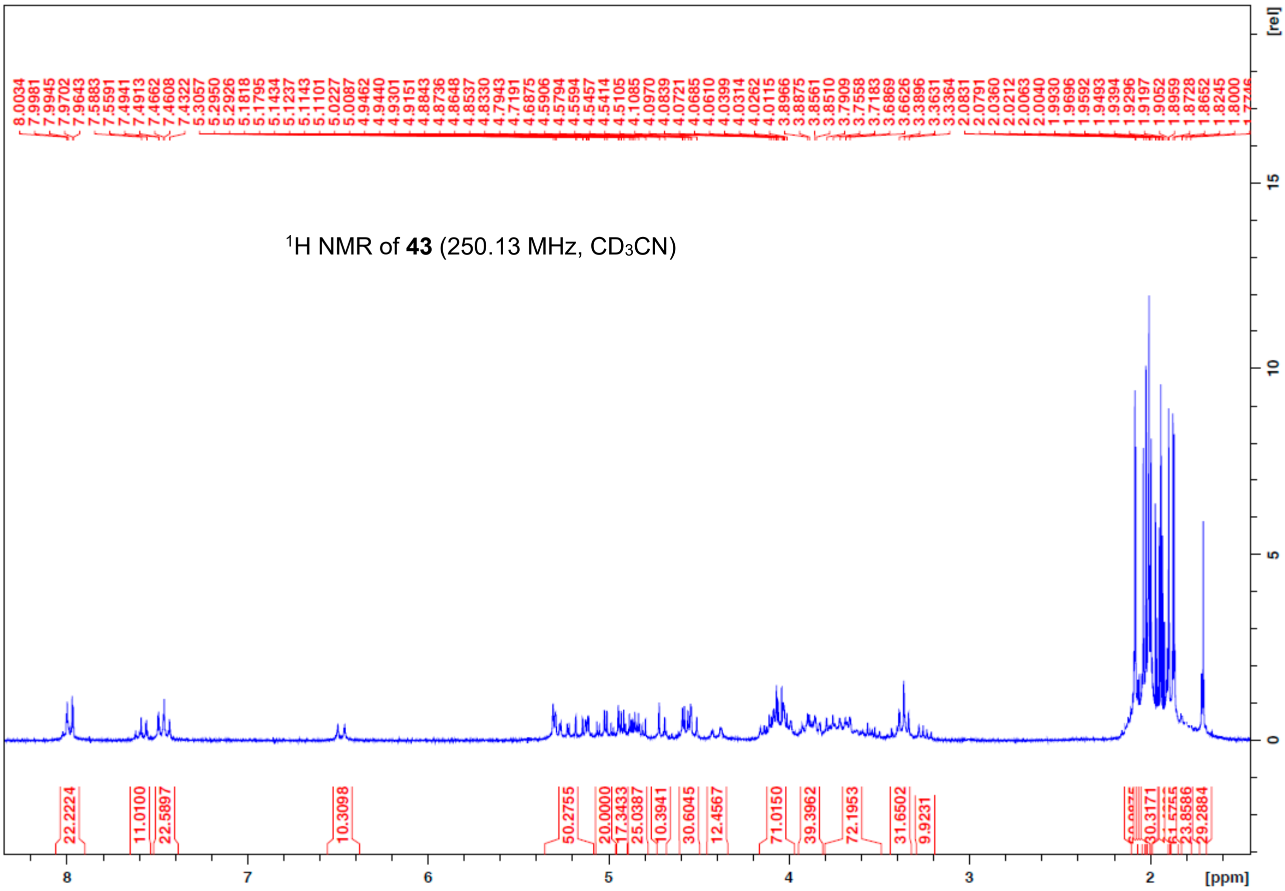

^{1}H NMR of **43** (250.13 MHz, CD_3CN)

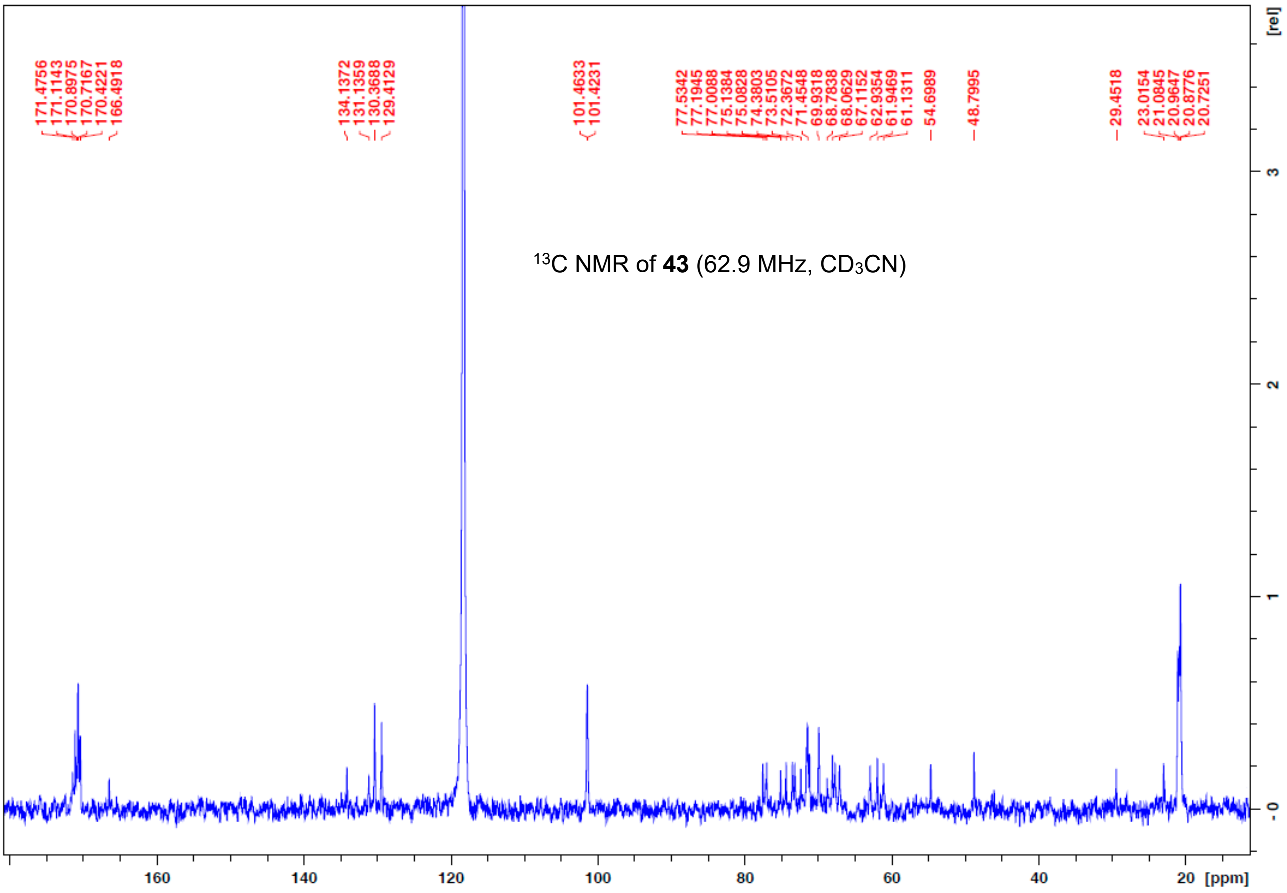

^{13}C NMR of **43** (62.9 MHz, CD_3CN)

^{13}C NMR and DEPT-135 of **43** (62.9 MHz, CD_3CN)

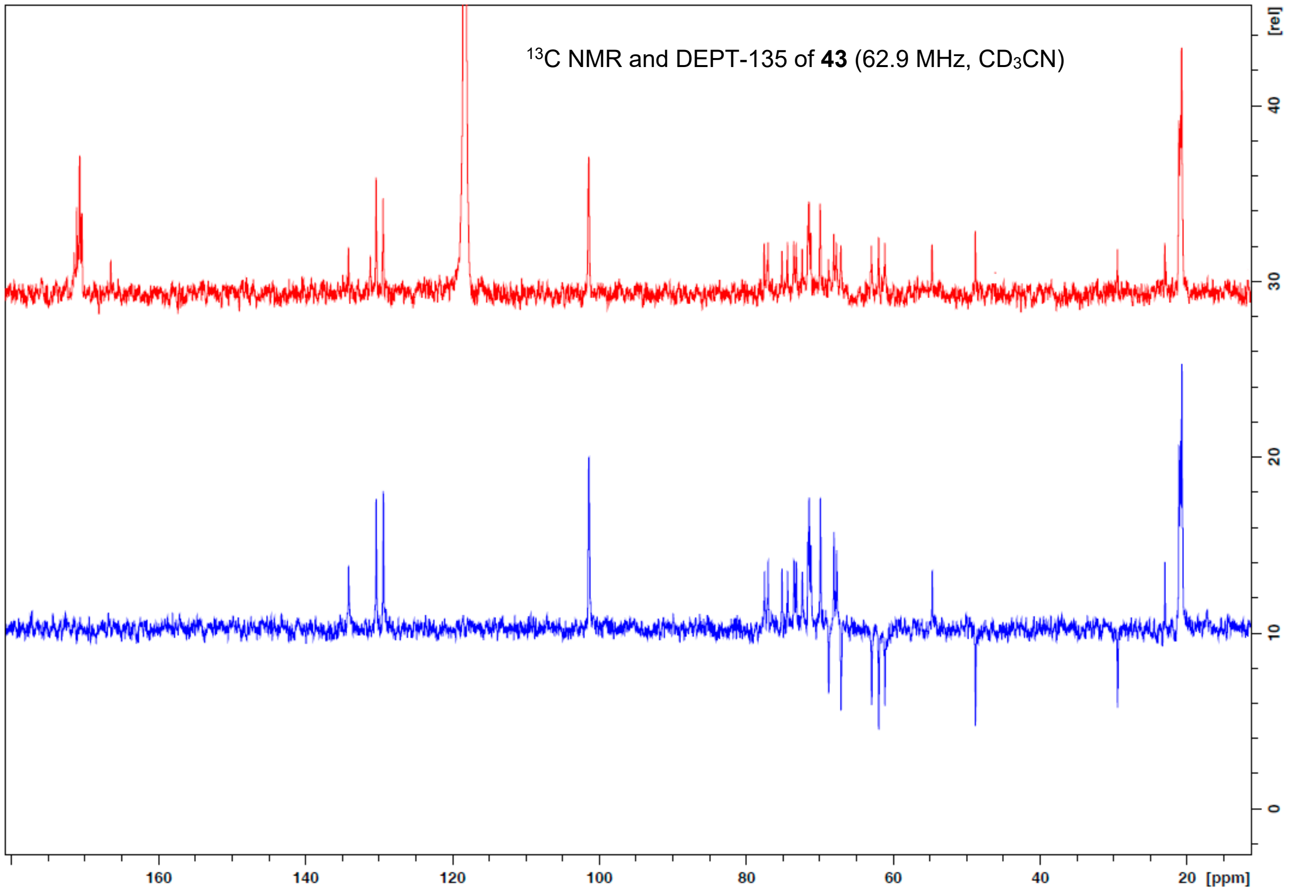

COSY spectra of **43** (250.13 MHz, CD_3CN)

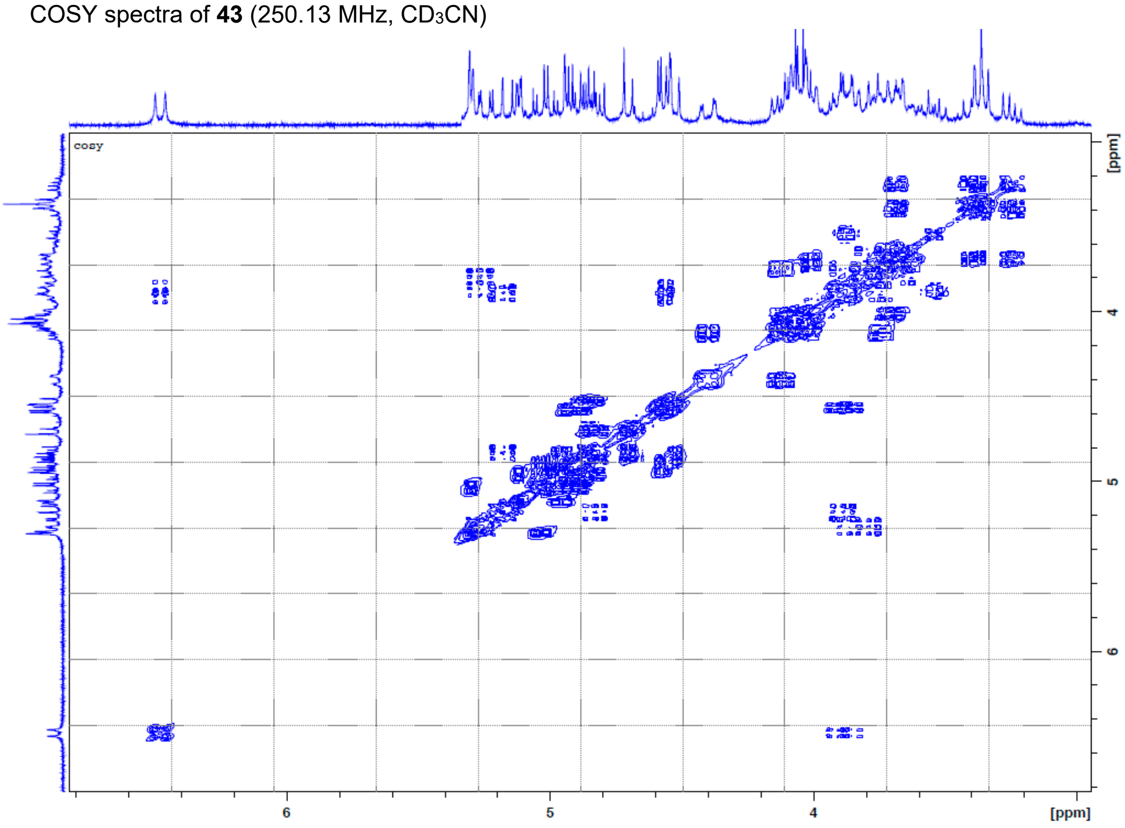

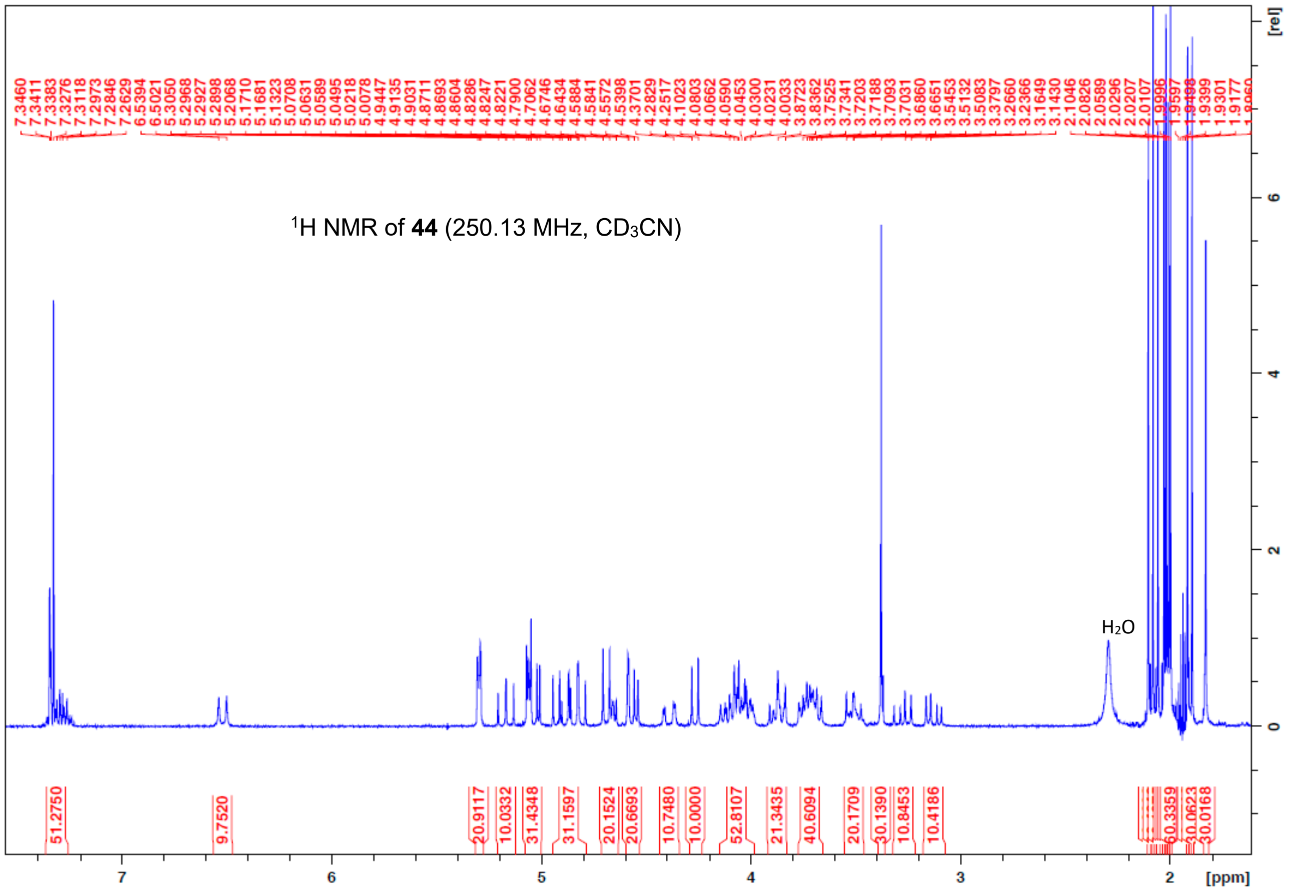
^{1}H NMR of **44** (250.13 MHz, CD_3CN)
H_2O
[ppm]
[rel]

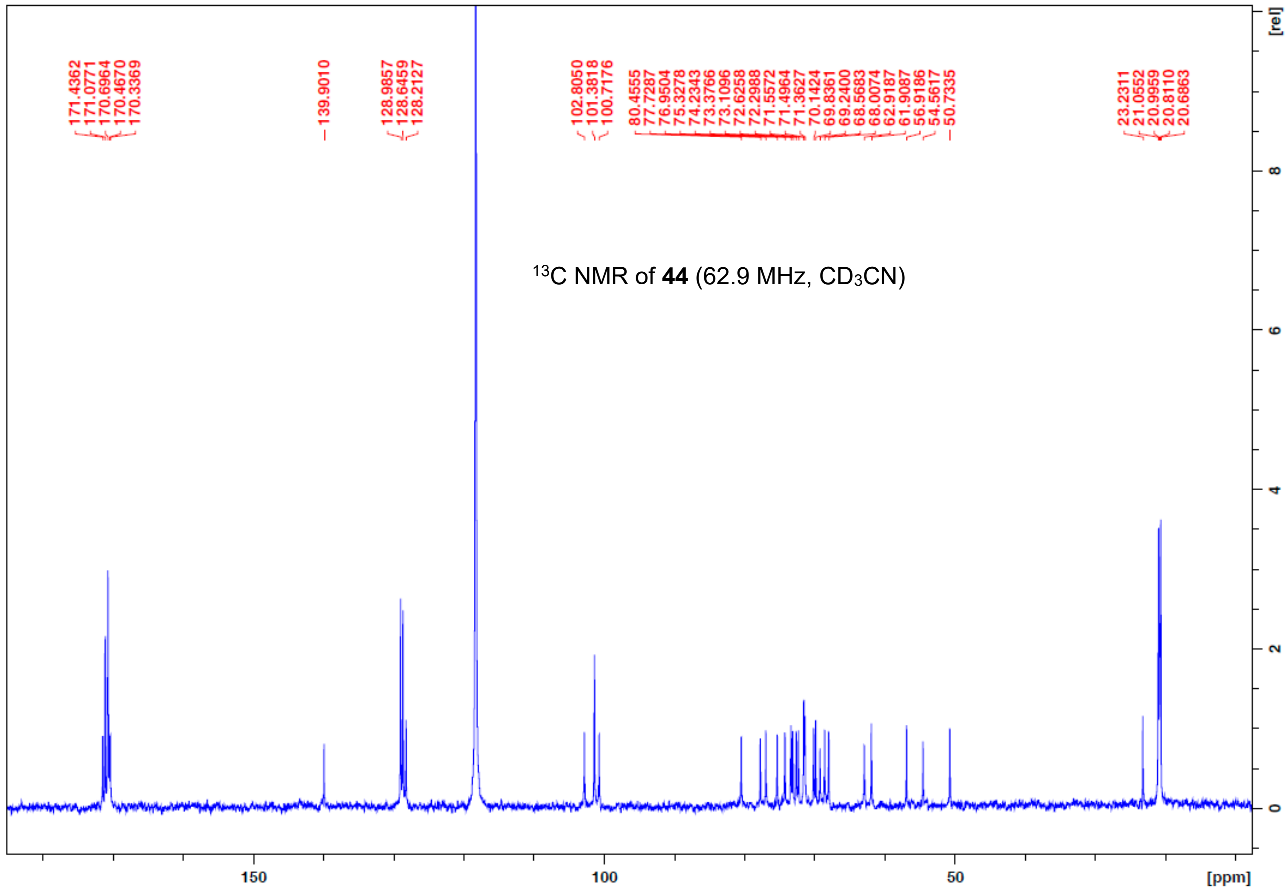
^{13}C NMR of **44** (62.9 MHz, CD_3CN)
[ppm]
[rel]

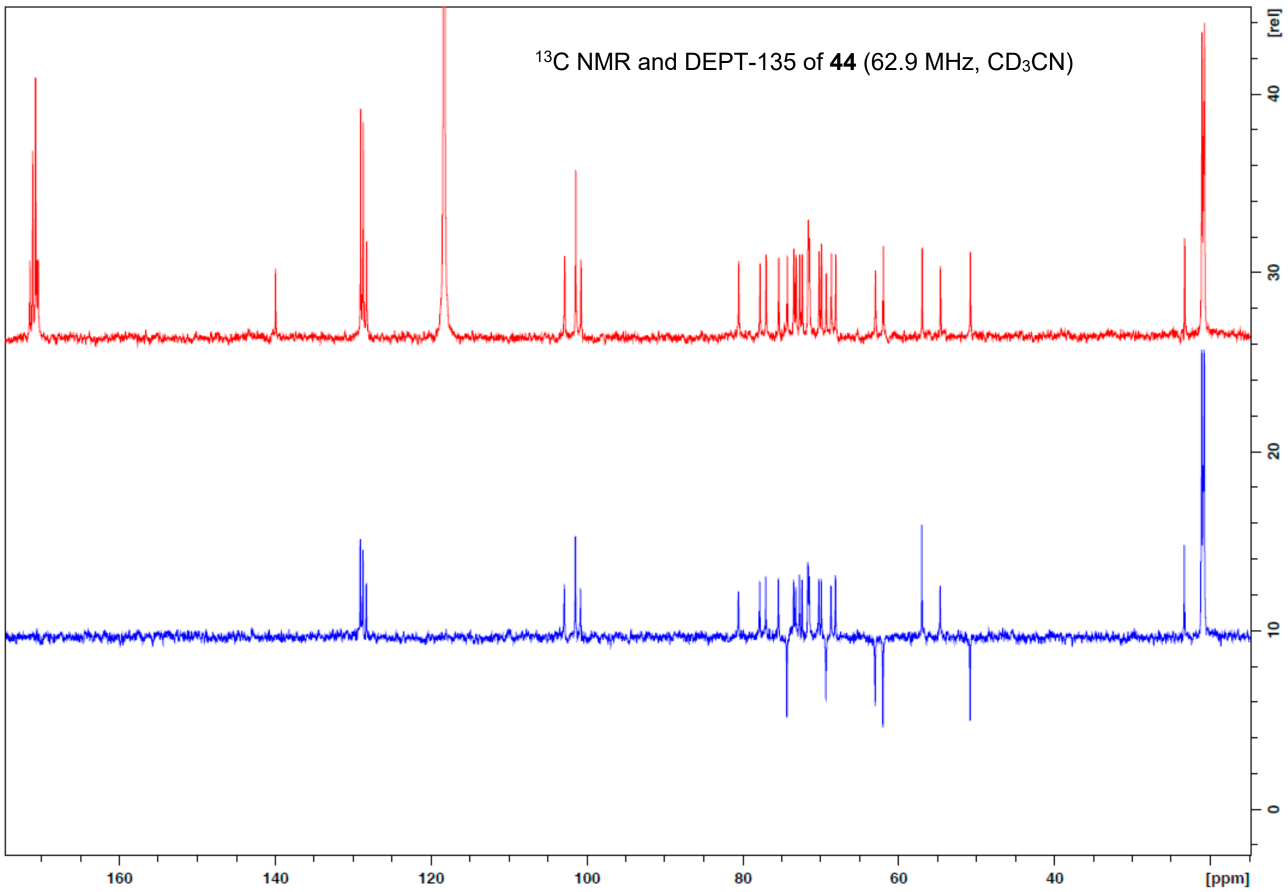

^{13}C NMR and DEPT-135 of **44** (62.9 MHz, CD_3CN)

COSY of **44** (250.13 MHz, CD_3CN)

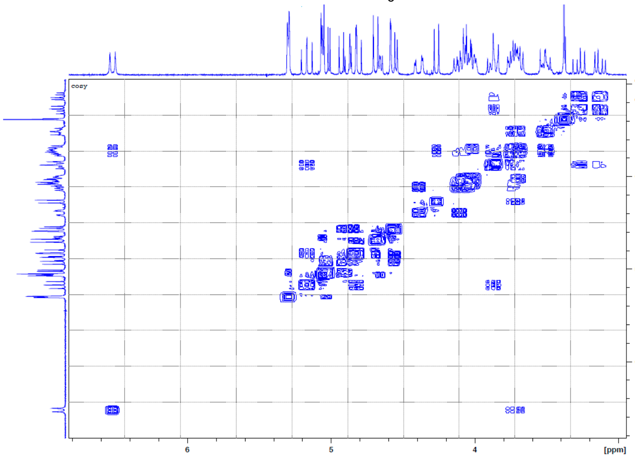

HETCOR of **44** (62.9 MHz, CD_3CN)

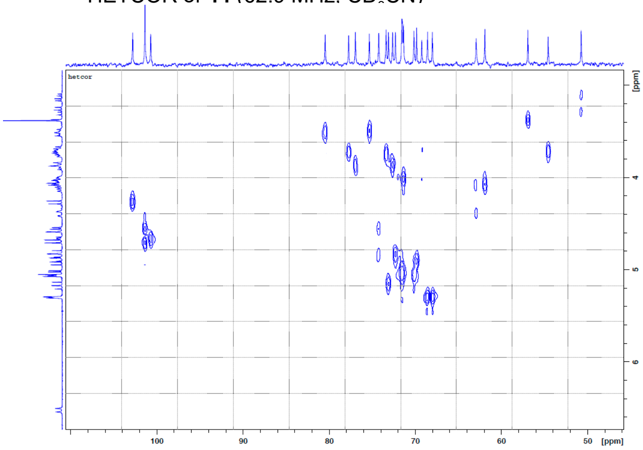

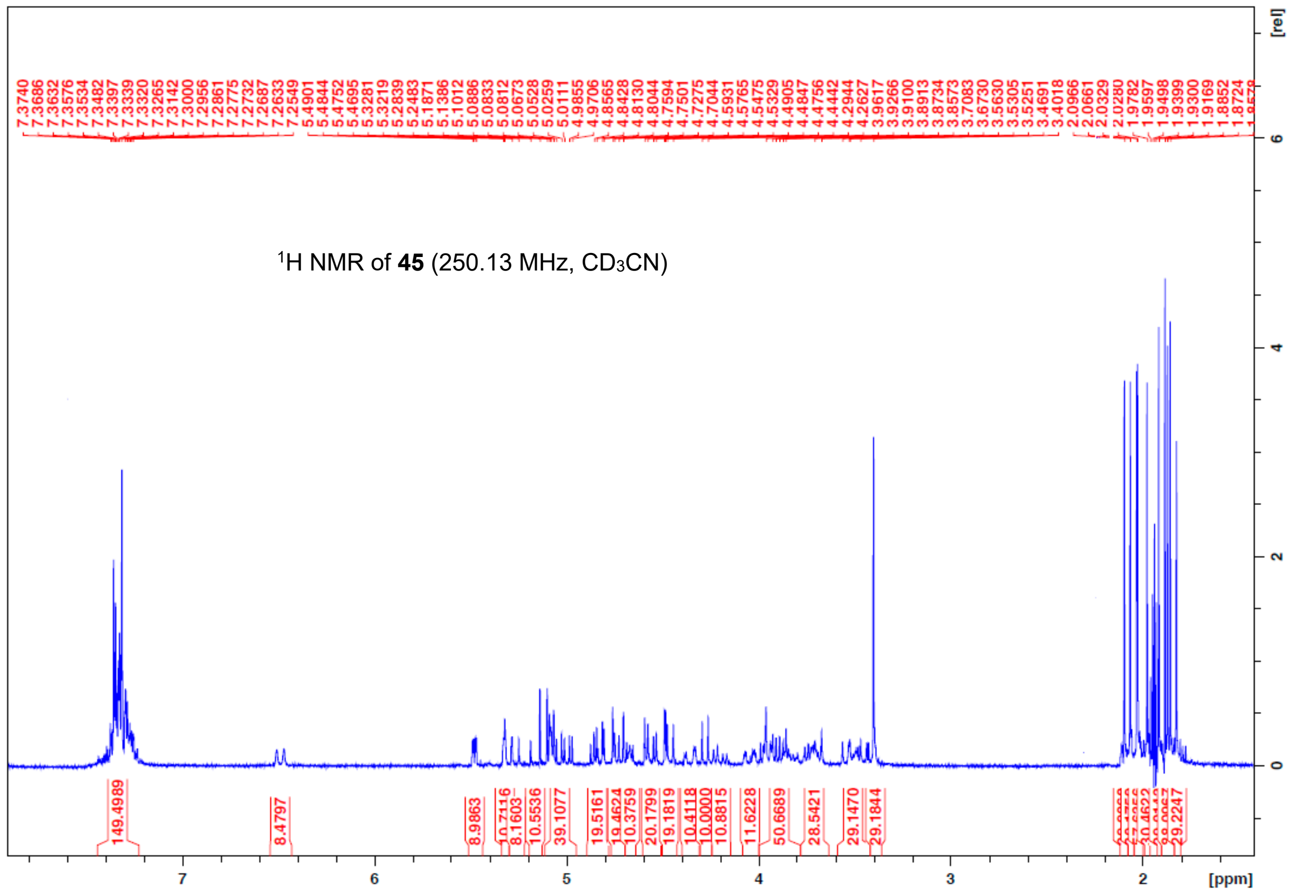

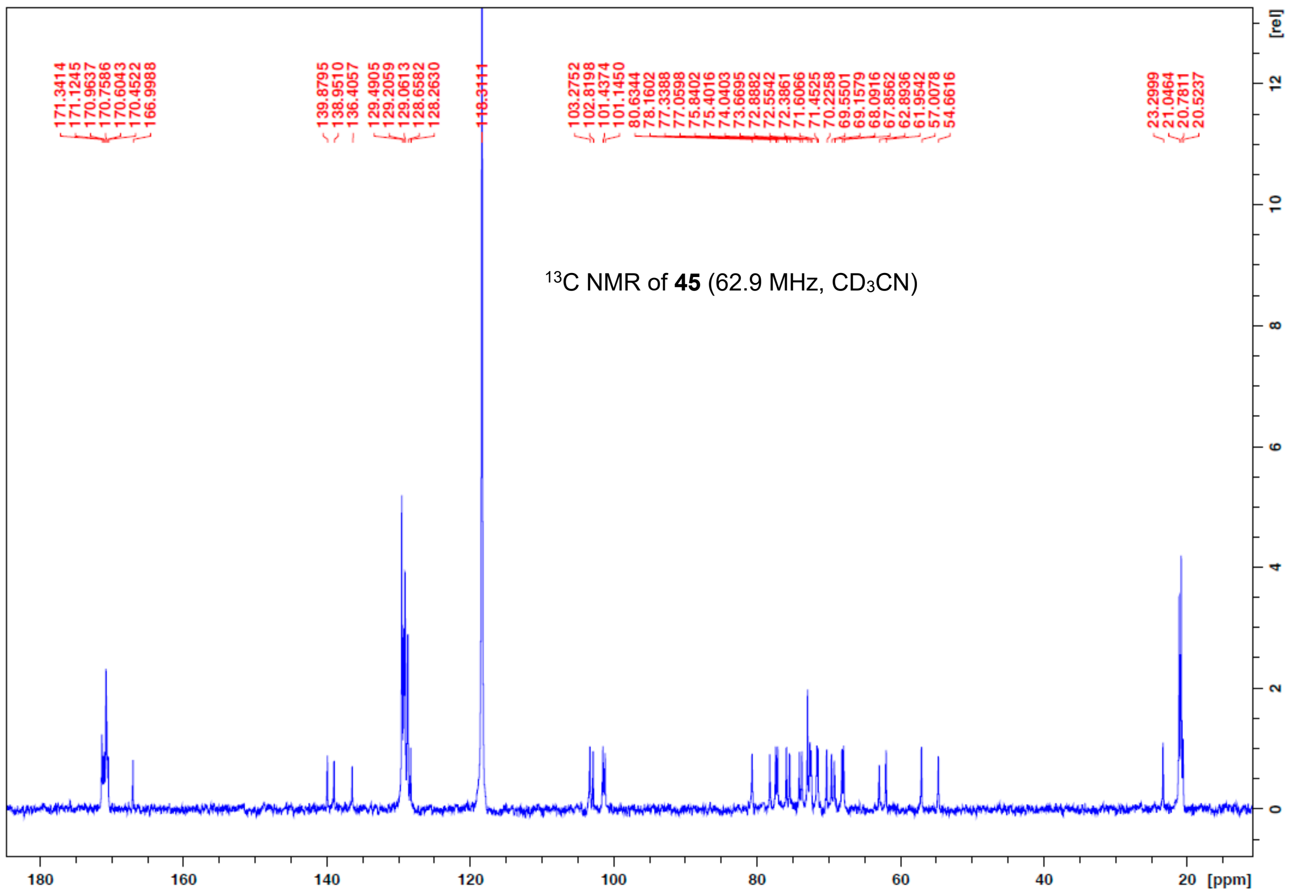

^{13}C NMR and DEPT-135 of **45** (62.9 MHz, CD_3CN)

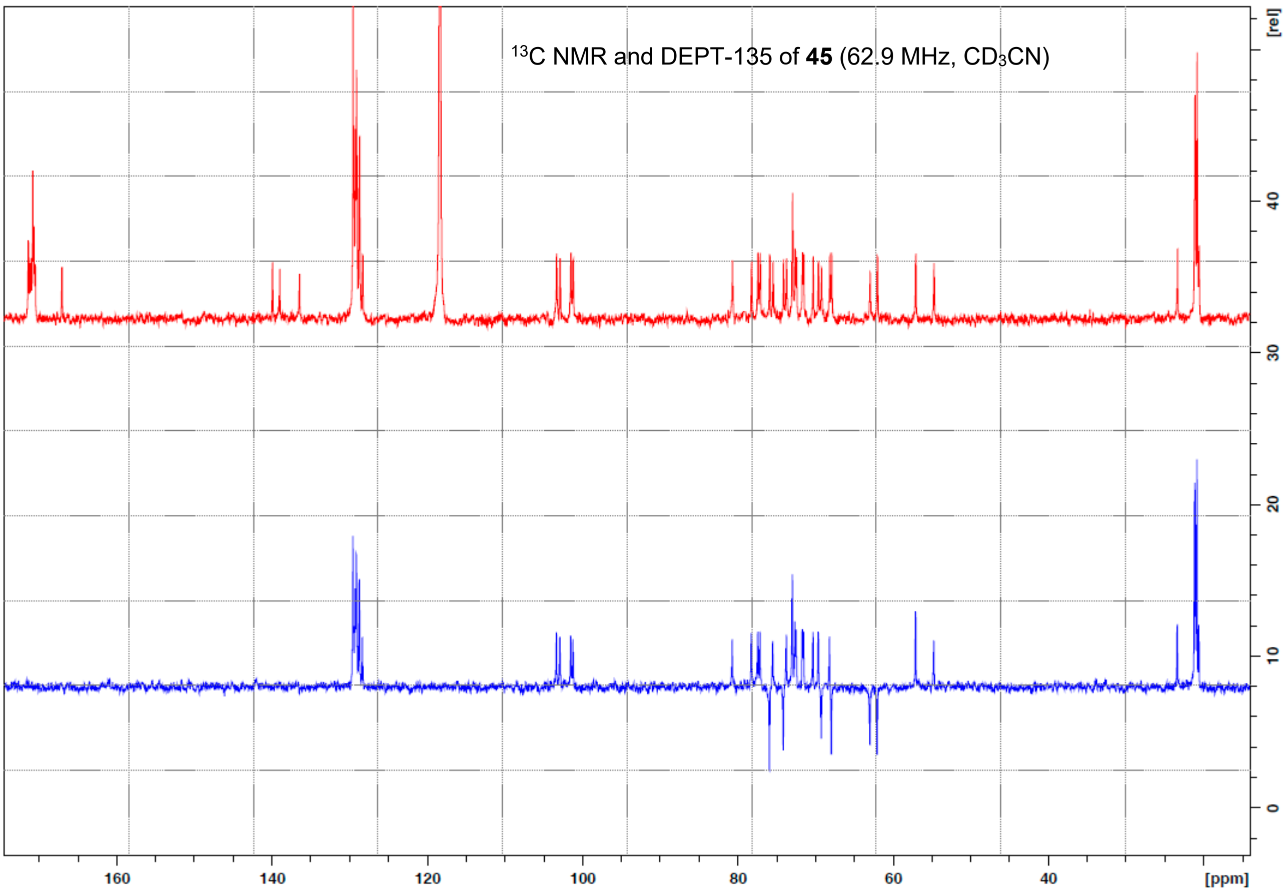

COSY of **45** (250.13 MHz, CD_3CN)

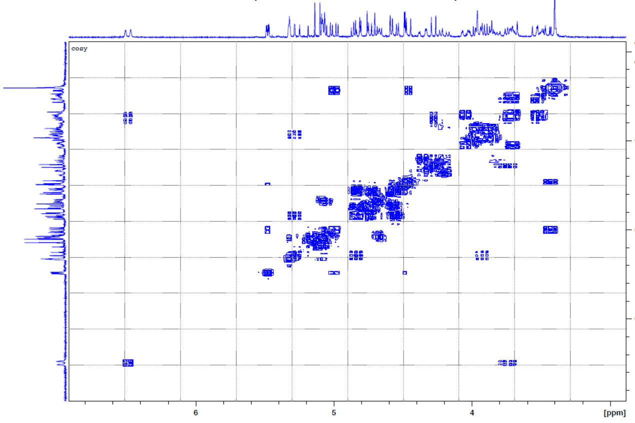

HETCOR of **45** (62.9 MHz, CD_3CN)

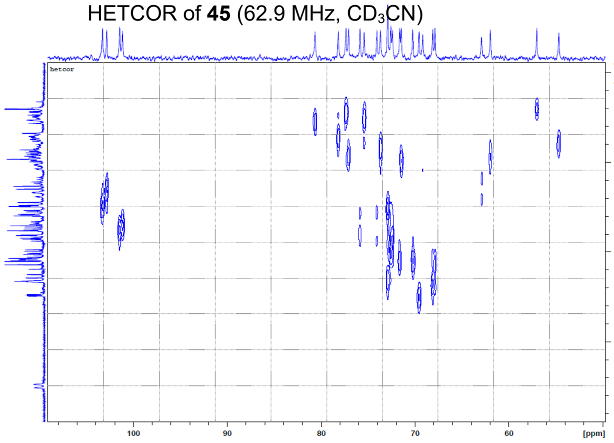

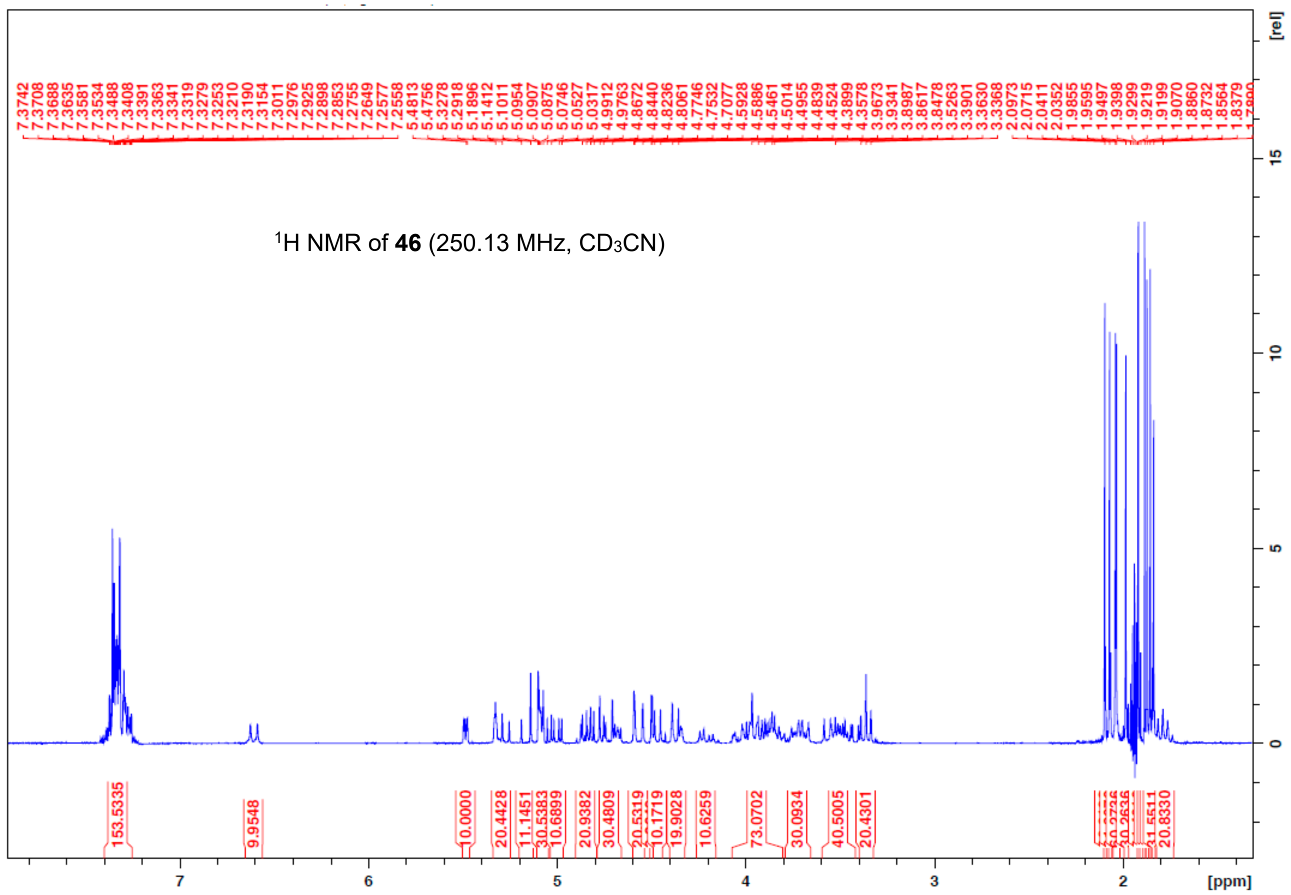
1H NMR of 46 (250.13 MHz, CD3CN)

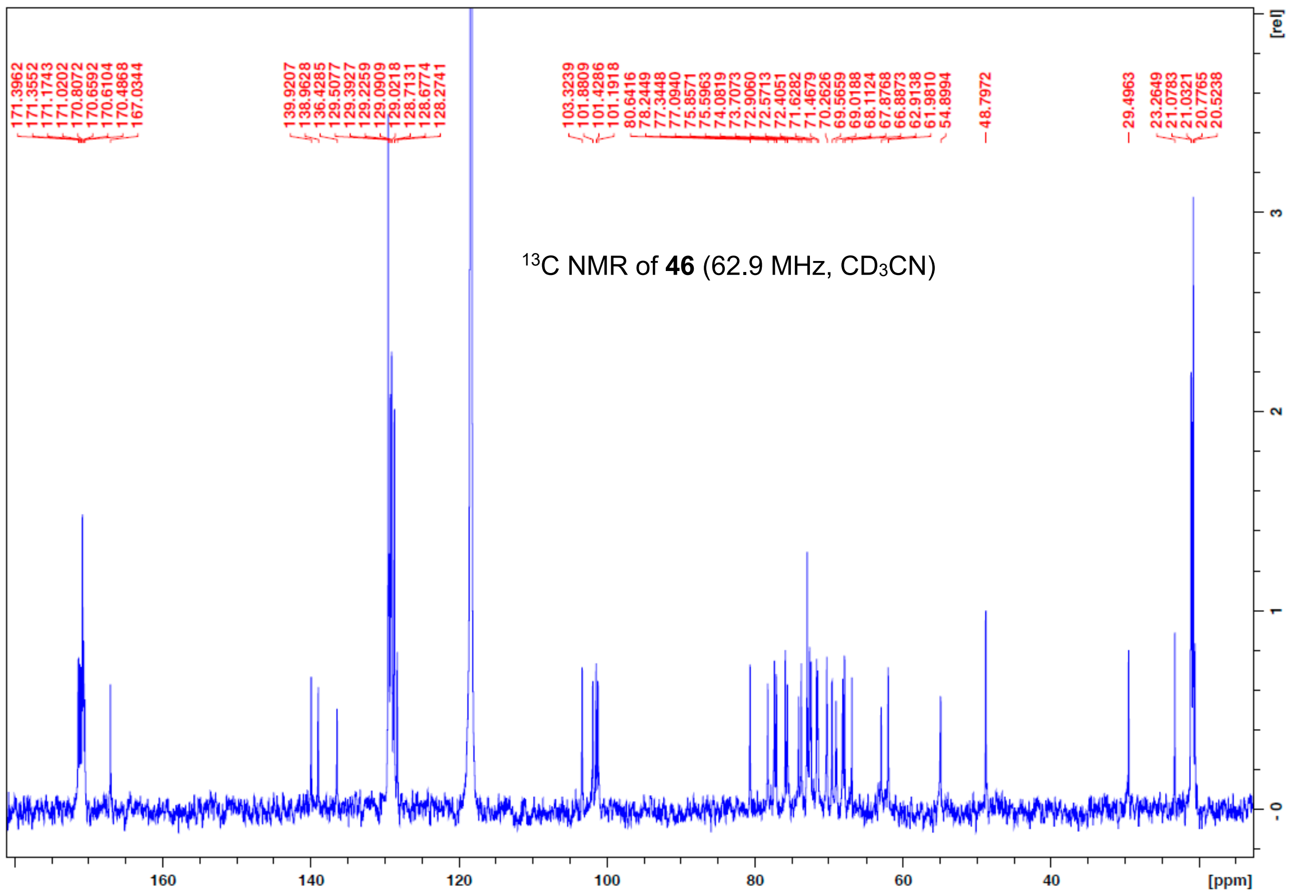
13C NMR of 46 (62.9 MHz, CD3CN)

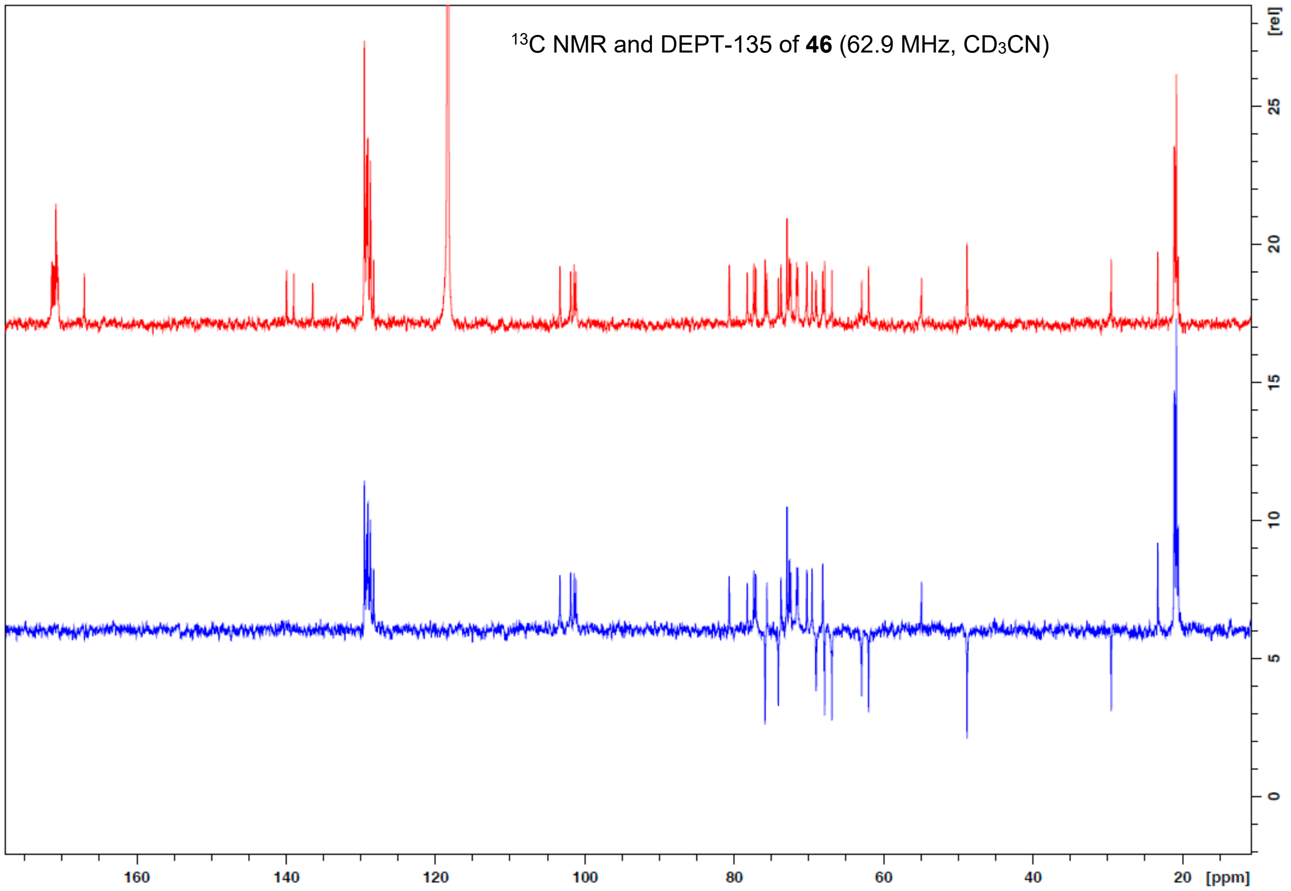

COSY of **46** (250.13 MHz, CD$_3$CN)

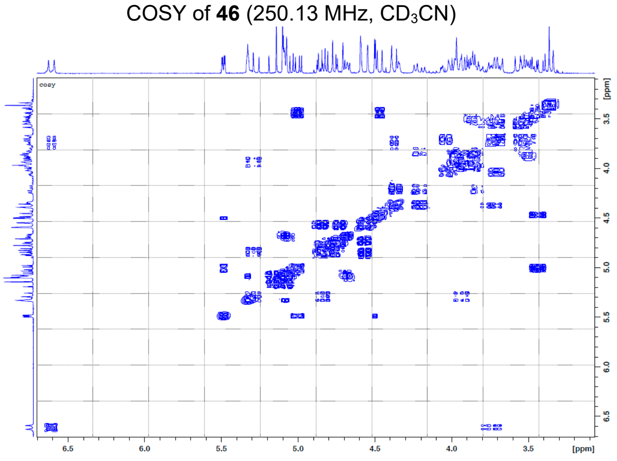

HETCOR of **46** (62.9 MHz, CD$_3$CN)

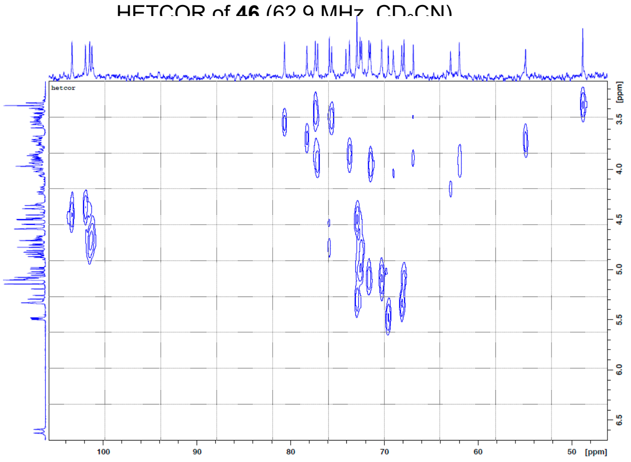

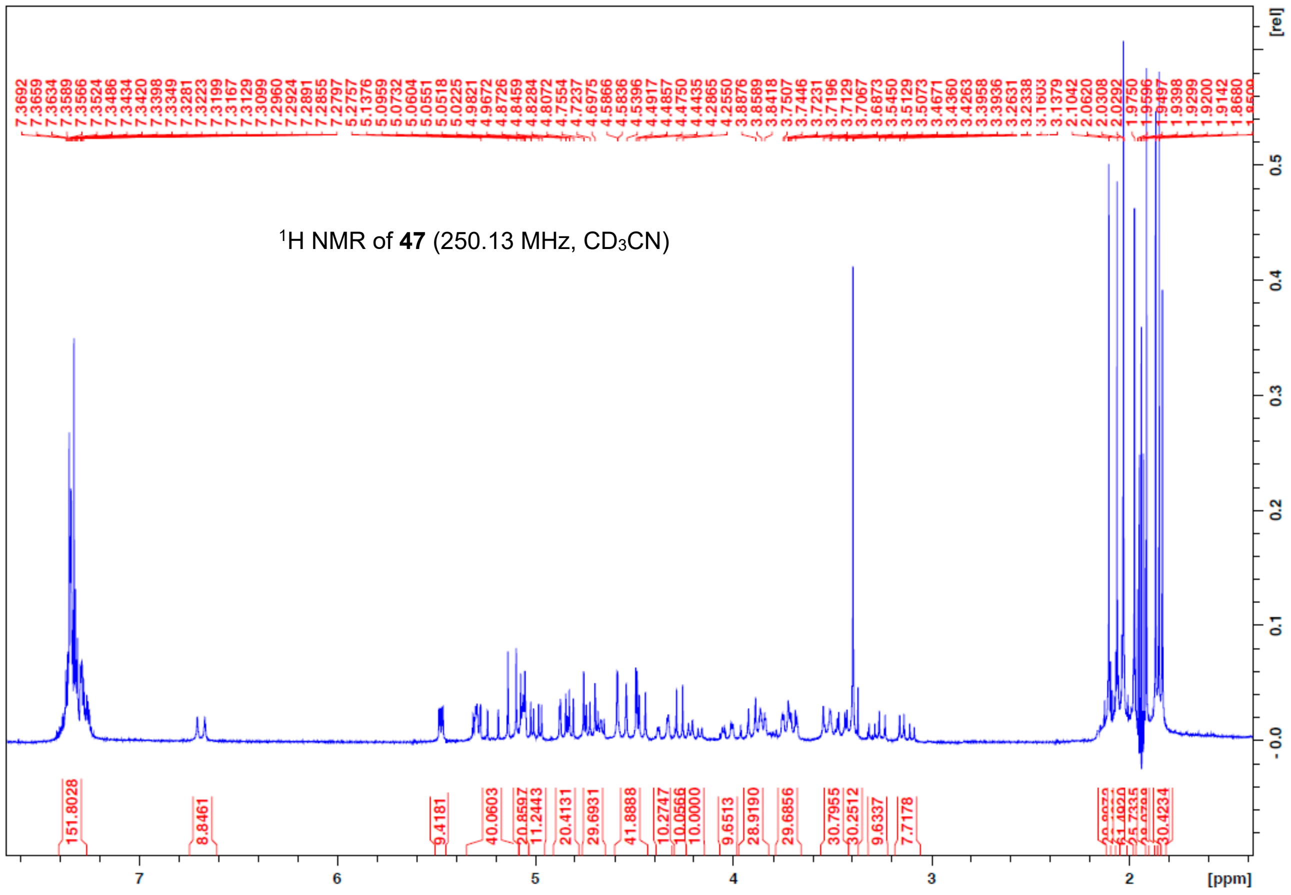

^{1}H NMR of **47** (250.13 MHz, CD3CN)
[rel]
[ppm]

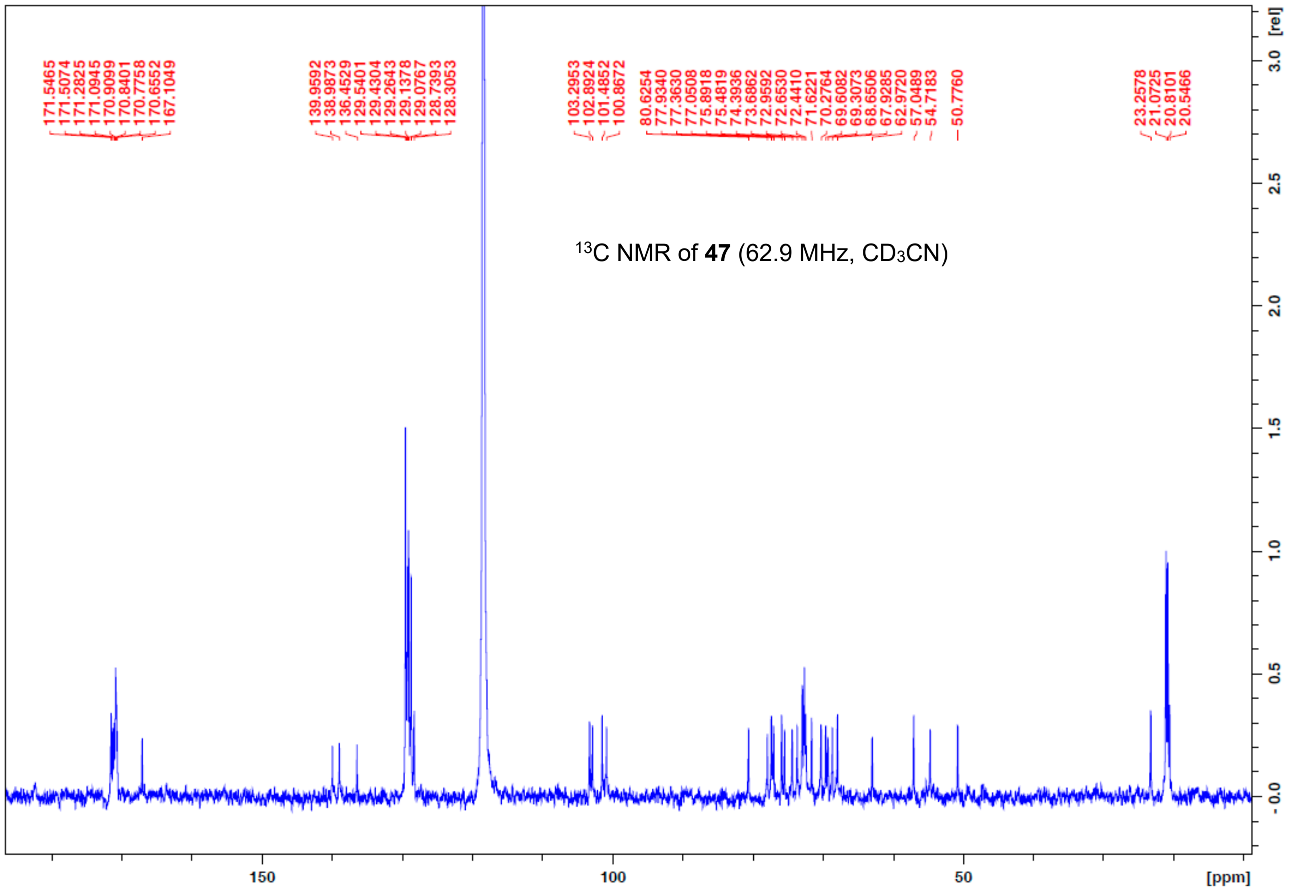

^{13}C NMR of **47** (62.9 MHz, CD3CN)
[rel]
[ppm]

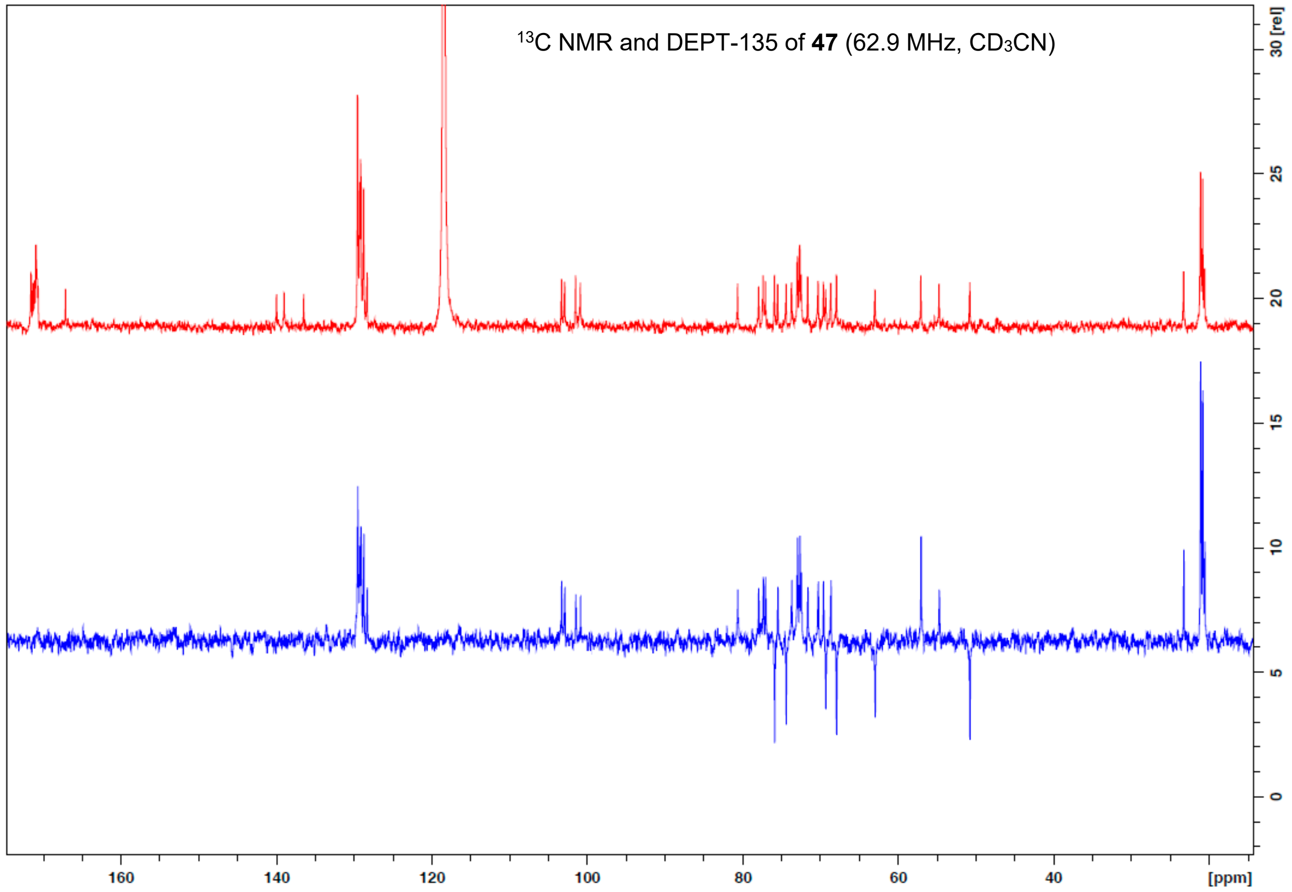

^{13}C NMR and DEPT-135 of **47** (62.9 MHz, CD_3CN)
160
140
120
100
80
60
40
[ppm]
30 [rel]
25
20
15
10
5
0

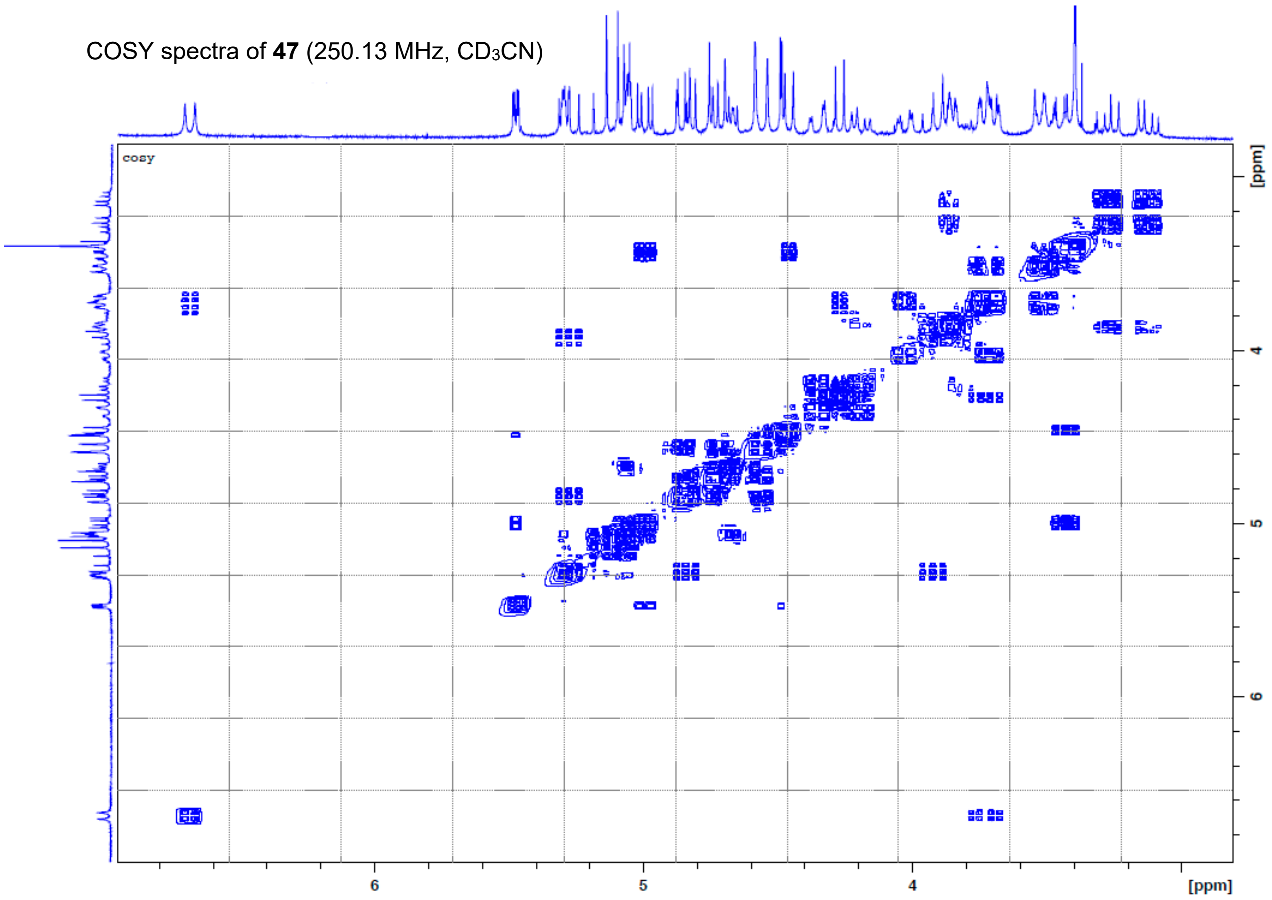

COSY spectra of **47** (250.13 MHz, CD_3CN)
cosy
6
5
4
[ppm]
[ppm]

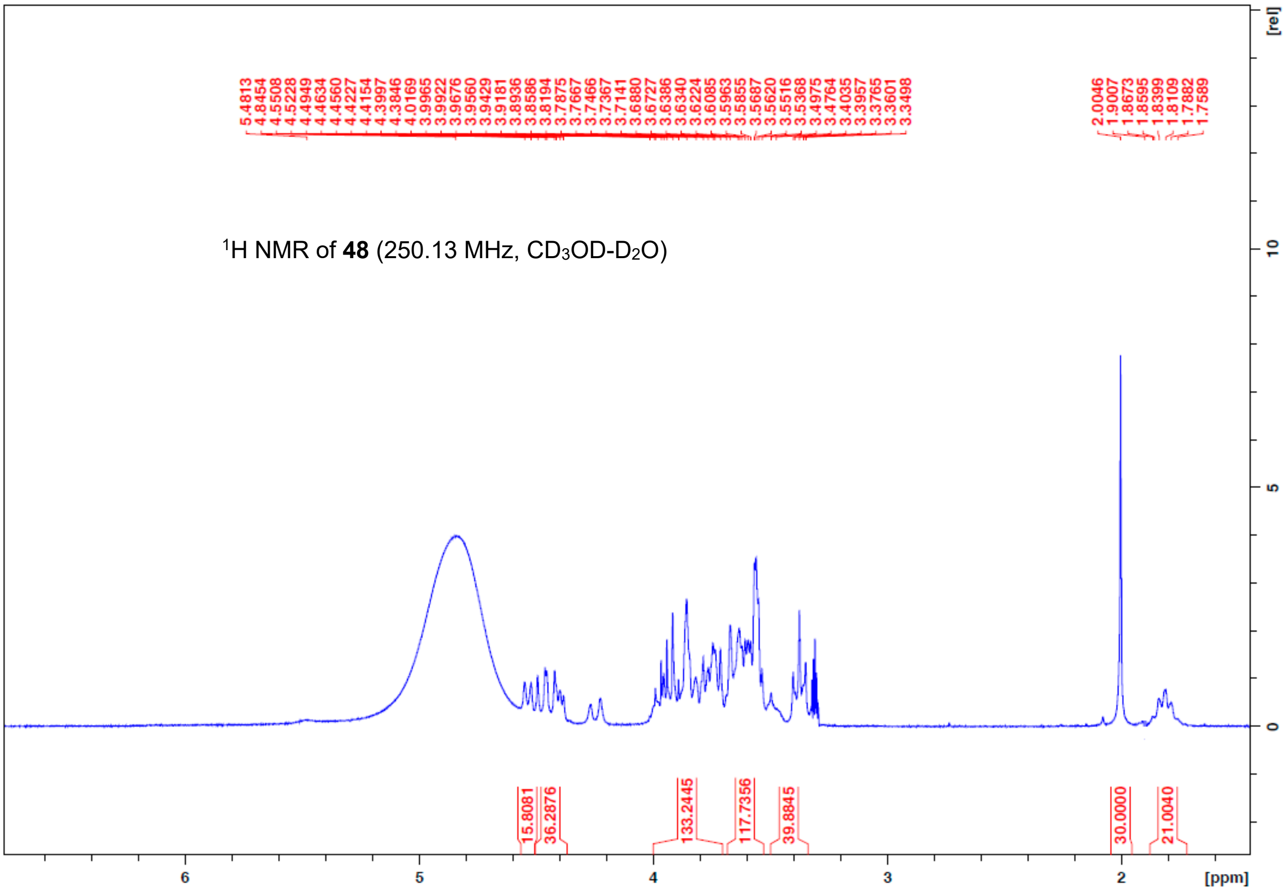

^{1}H NMR of **48** (250.13 MHz, CD_3OD-D_2O)

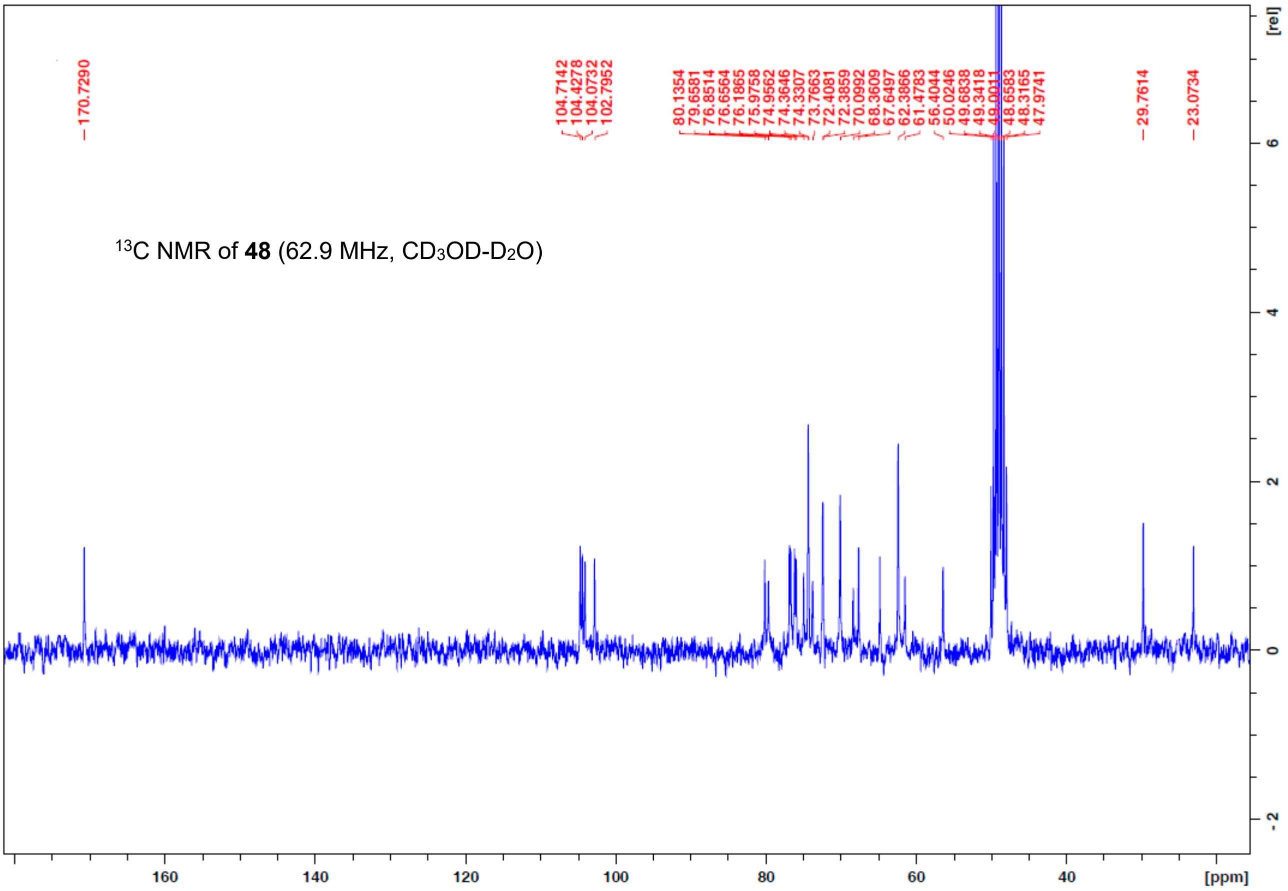

^{13}C NMR of **48** (62.9 MHz, CD_3OD-D_2O)

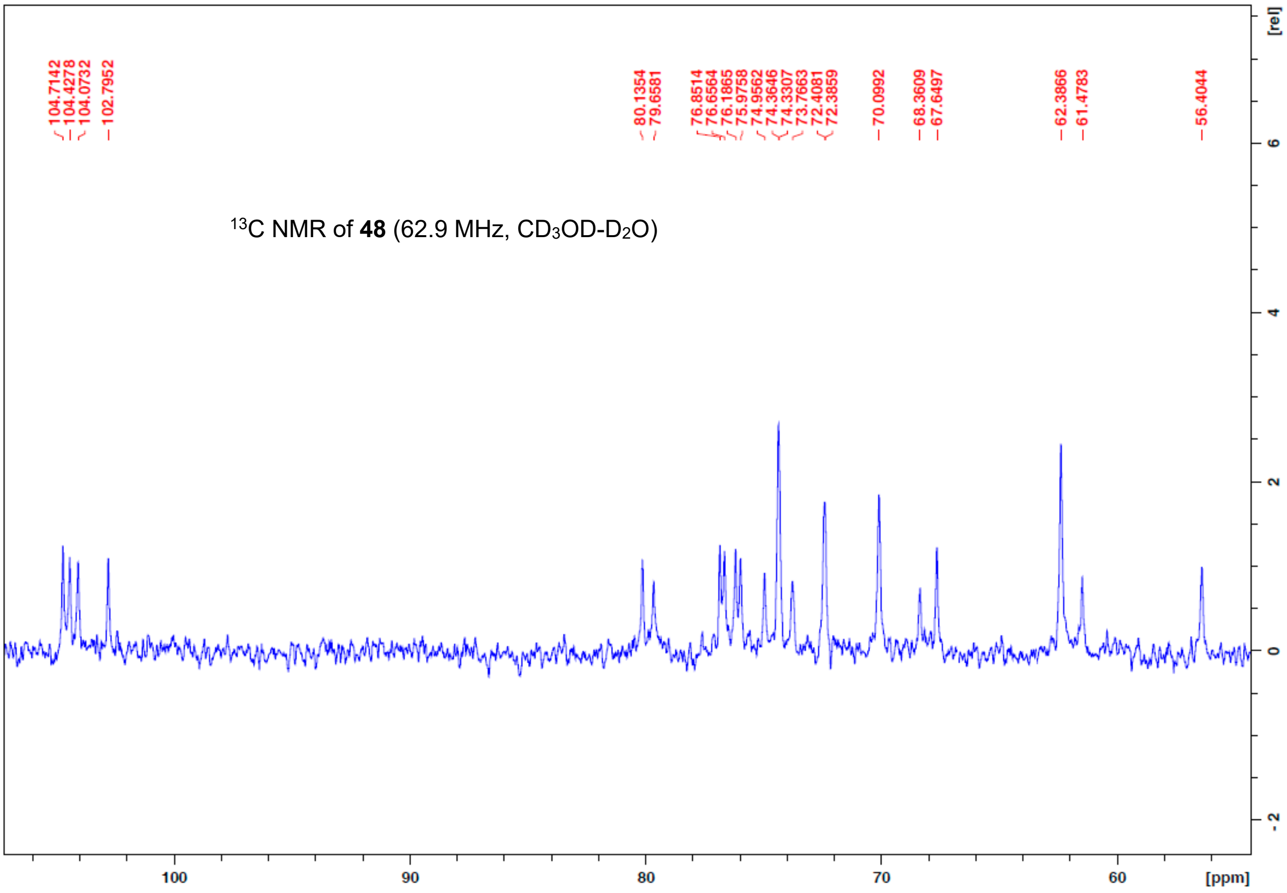

^{13}C NMR of **48** (62.9 MHz, CD_3OD-D_2O)

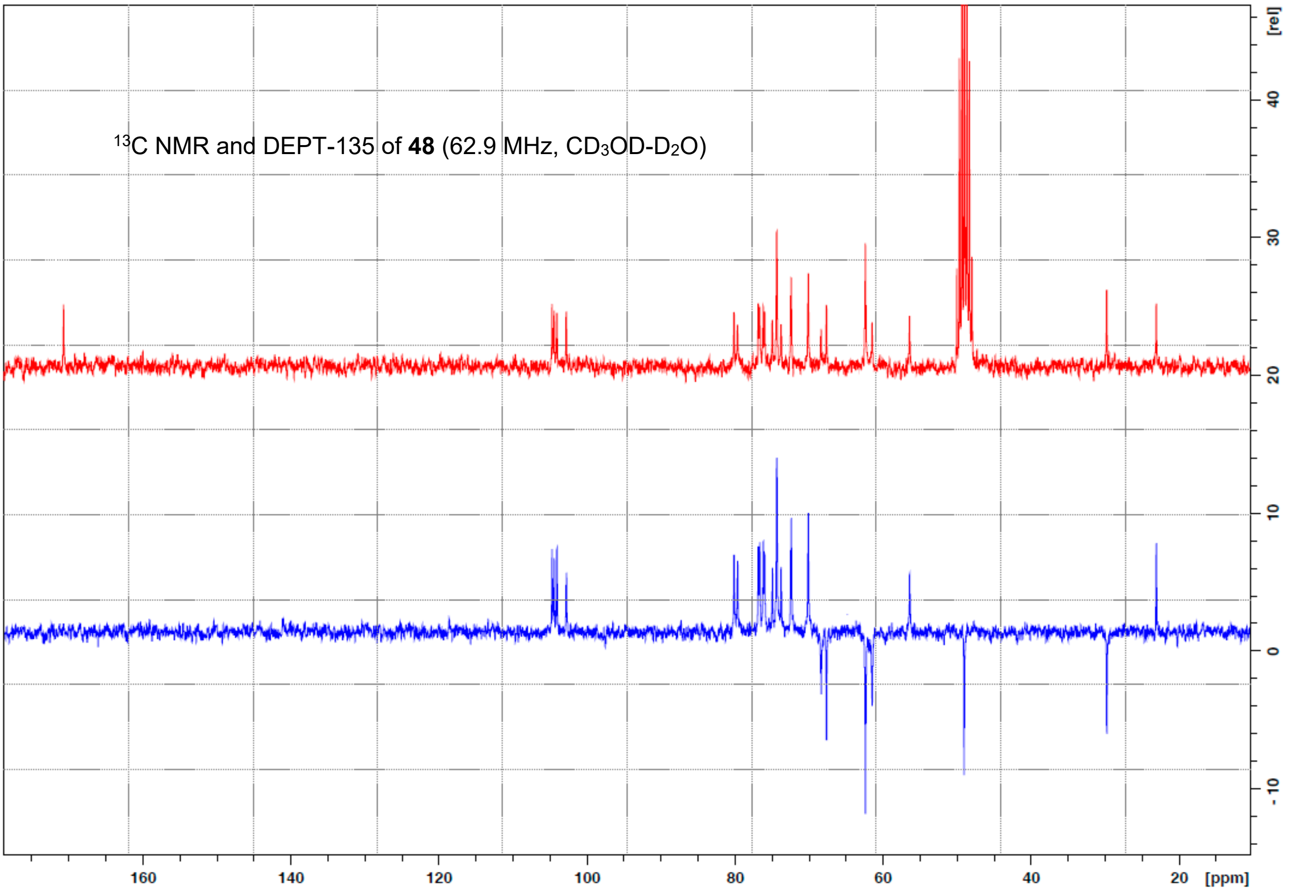

^{13}C NMR and DEPT-135 of **48** (62.9 MHz, CD_3OD-D_2O)

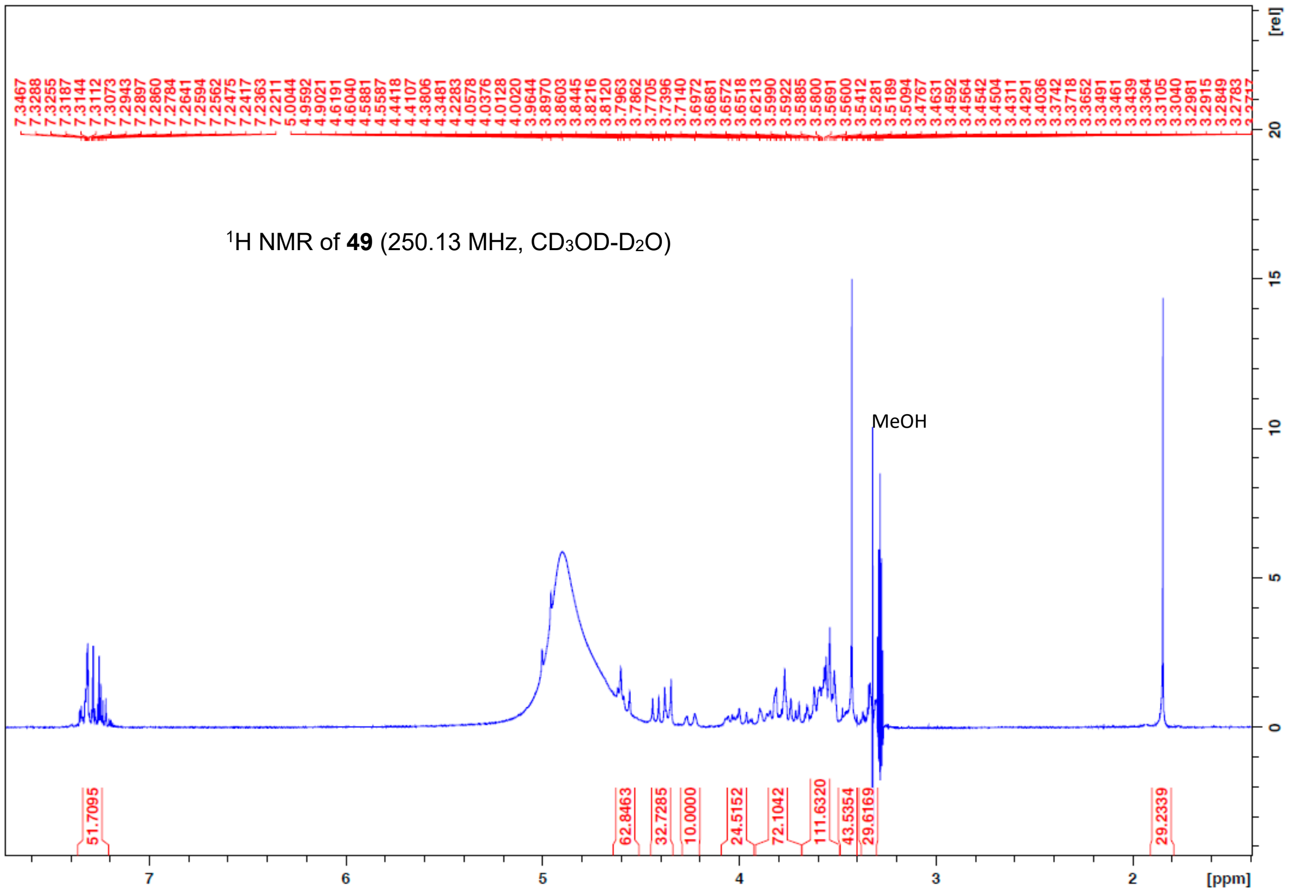

^{1}H NMR of **49** (250.13 MHz, CD_3OD-D_2O)
MeOH

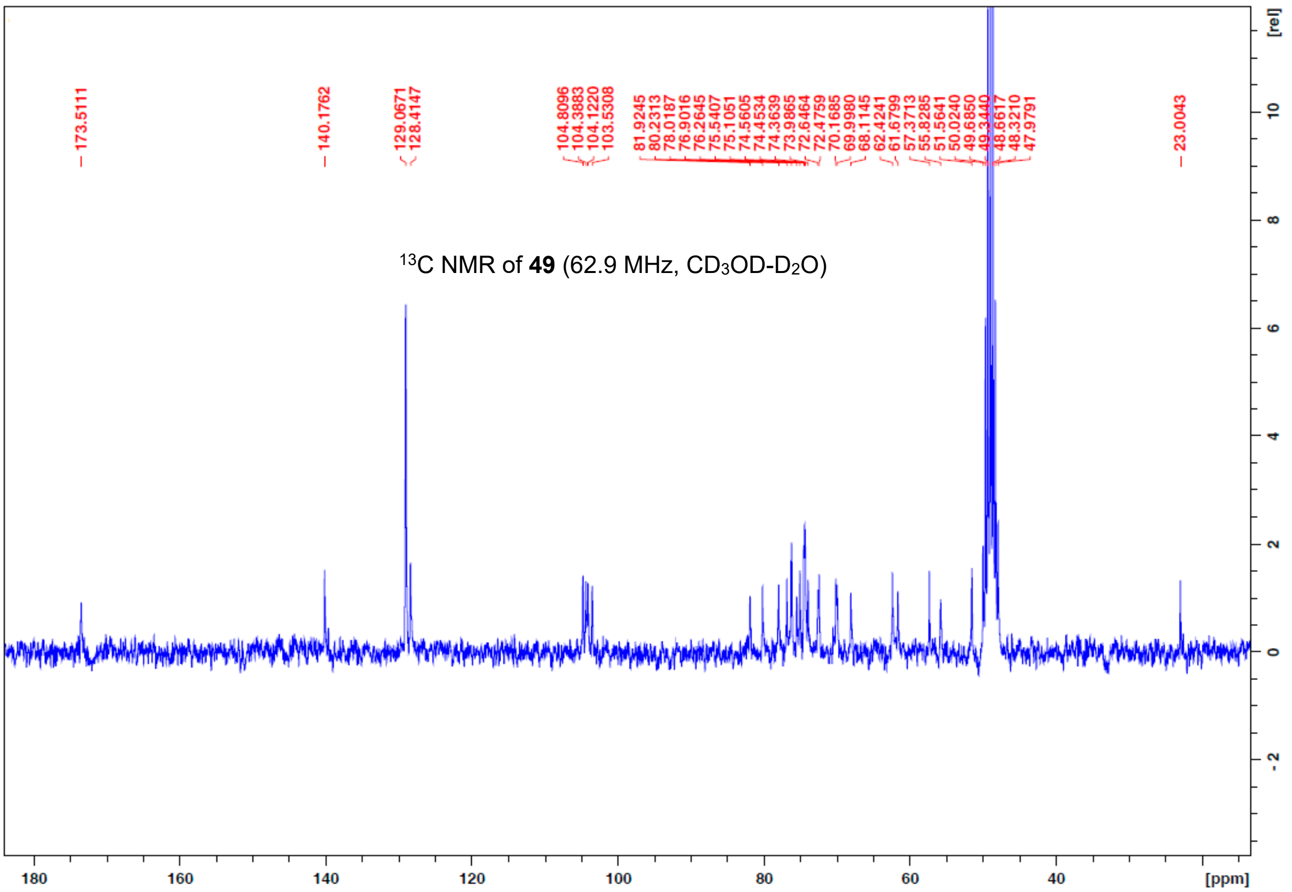

^{13}C NMR of **49** (62.9 MHz, CD_3OD-D_2O)

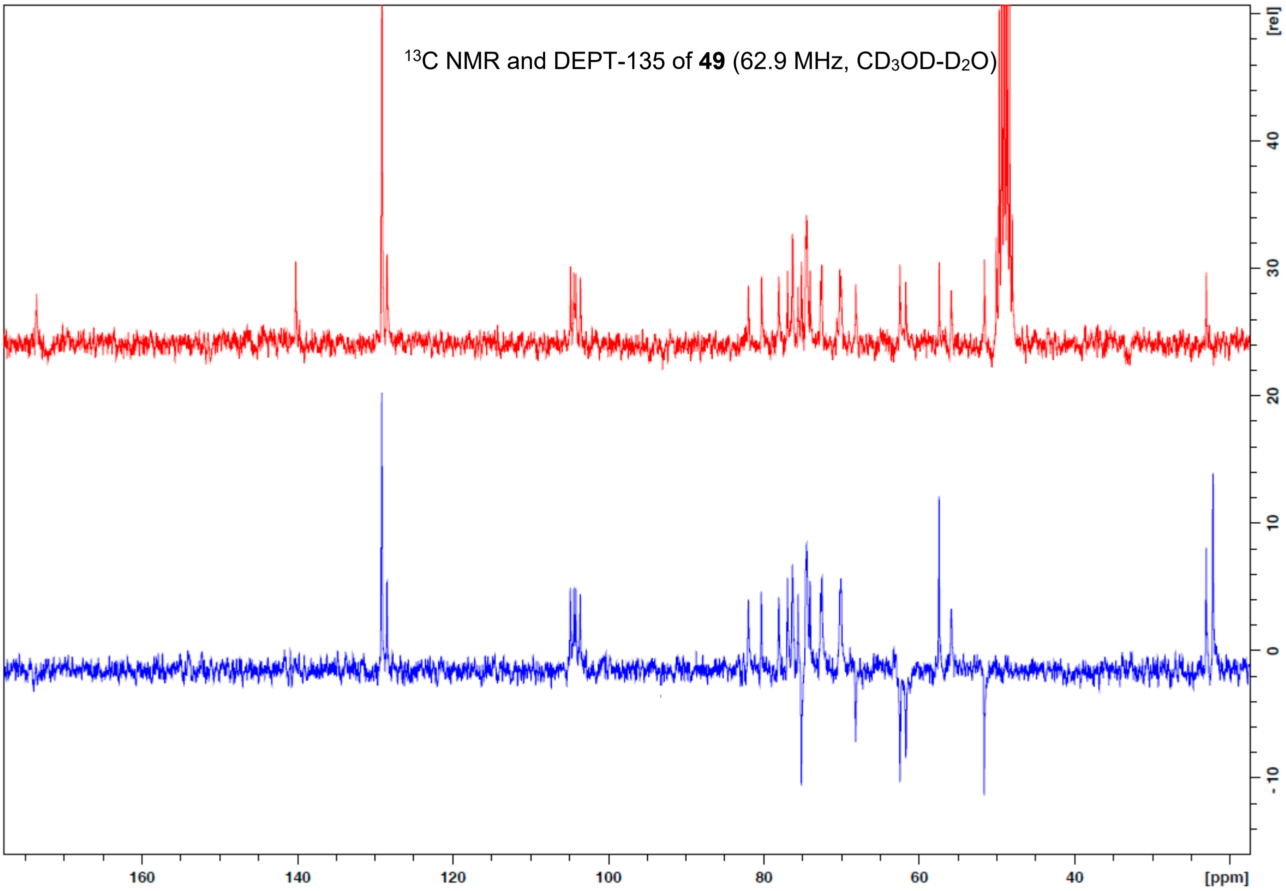

^{13}C NMR and DEPT-135 of **49** (62.9 MHz, CD_3OD-D_2O)

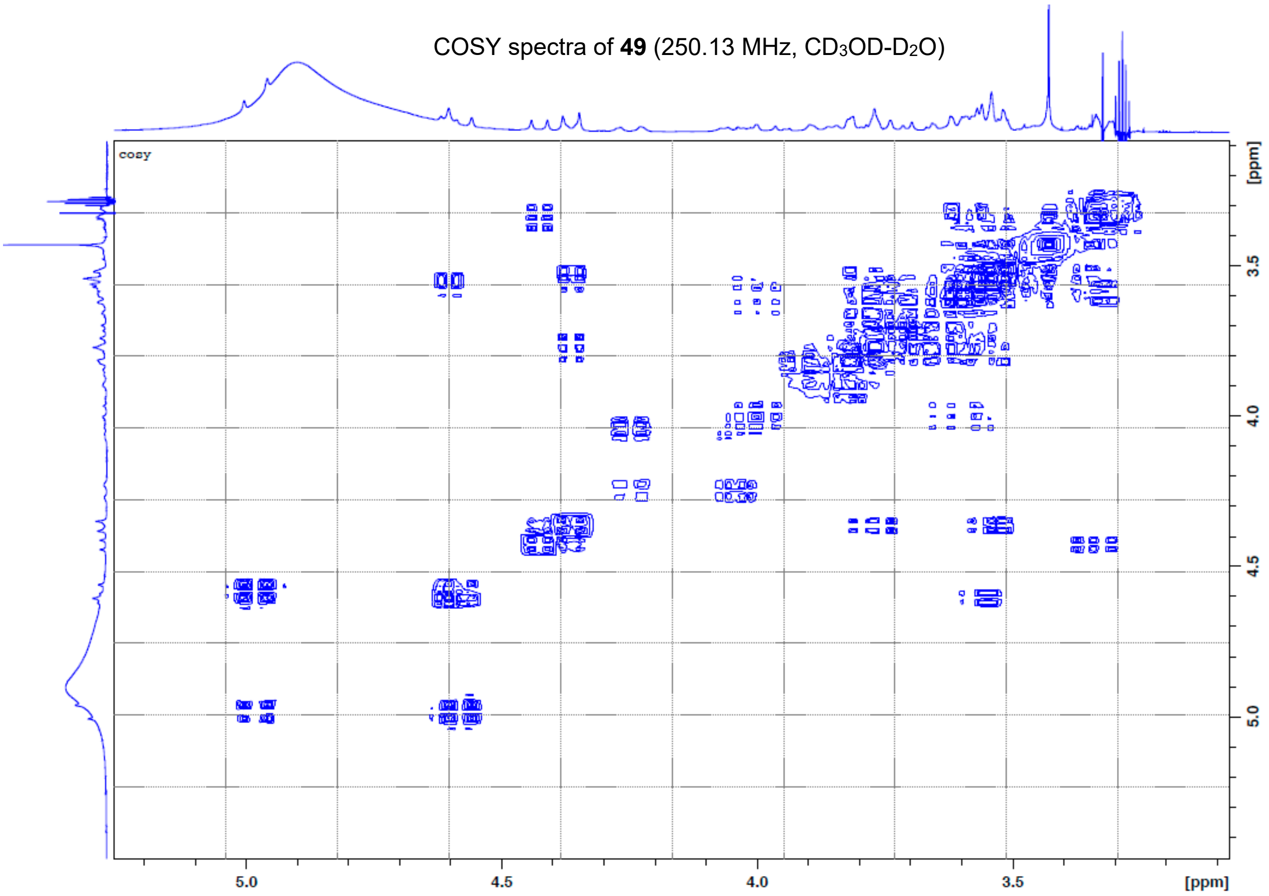

COSY spectra of **49** (250.13 MHz, CD_3OD-D_2O)

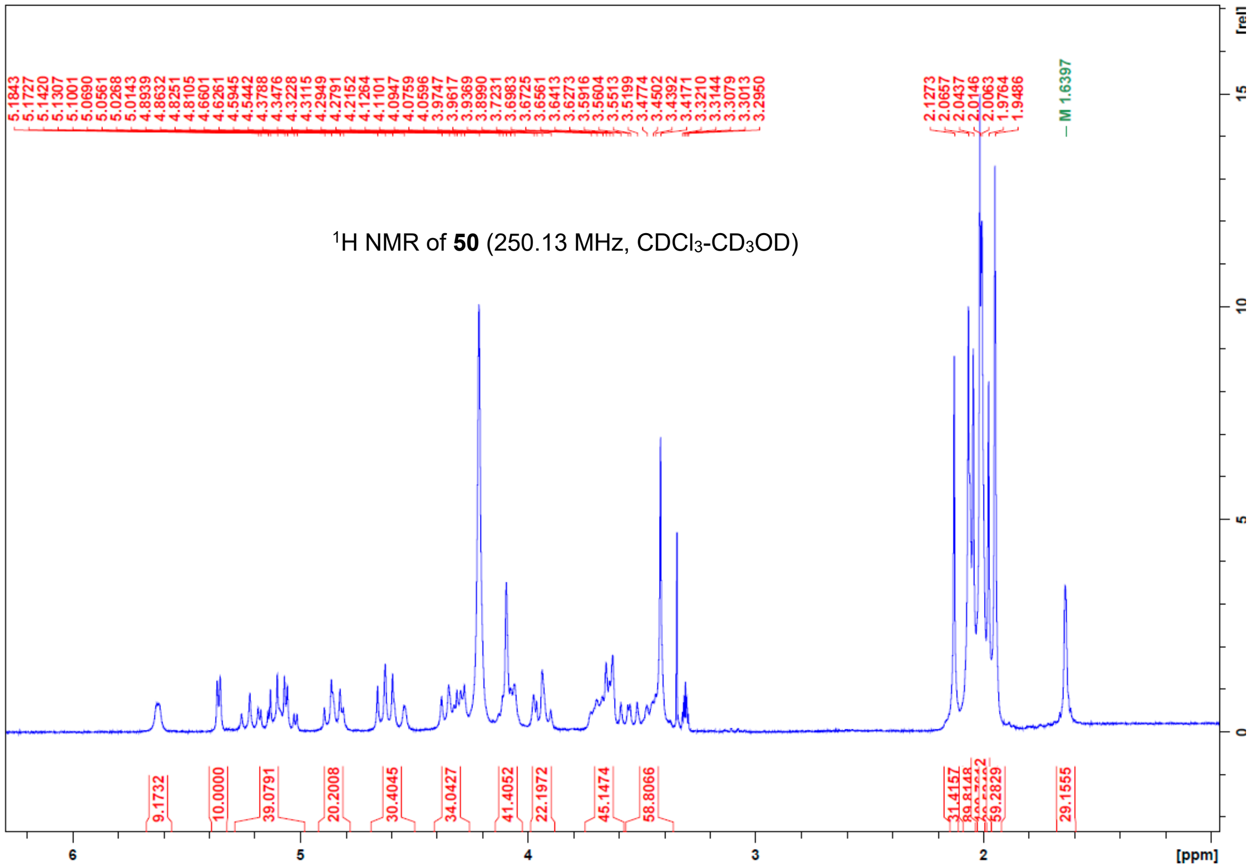

^{1}H NMR of **50** (250.13 MHz, $CDCl_3$-CD_3OD)

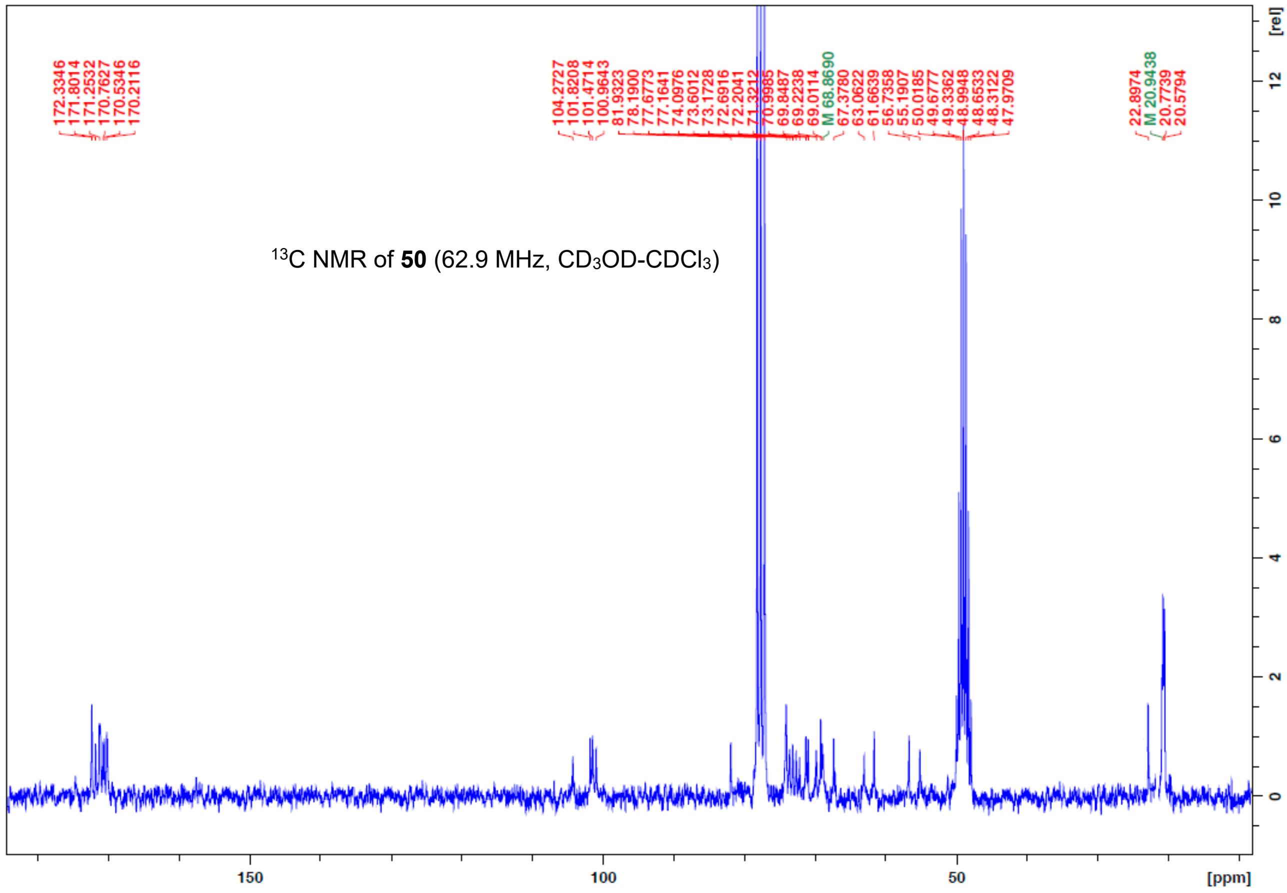

^{13}C NMR of **50** (62.9 MHz, CD_3OD-$CDCl_3$)

COSY spectra of **50** (250.13 MHz, $CDCl_3$-CD_3OD)

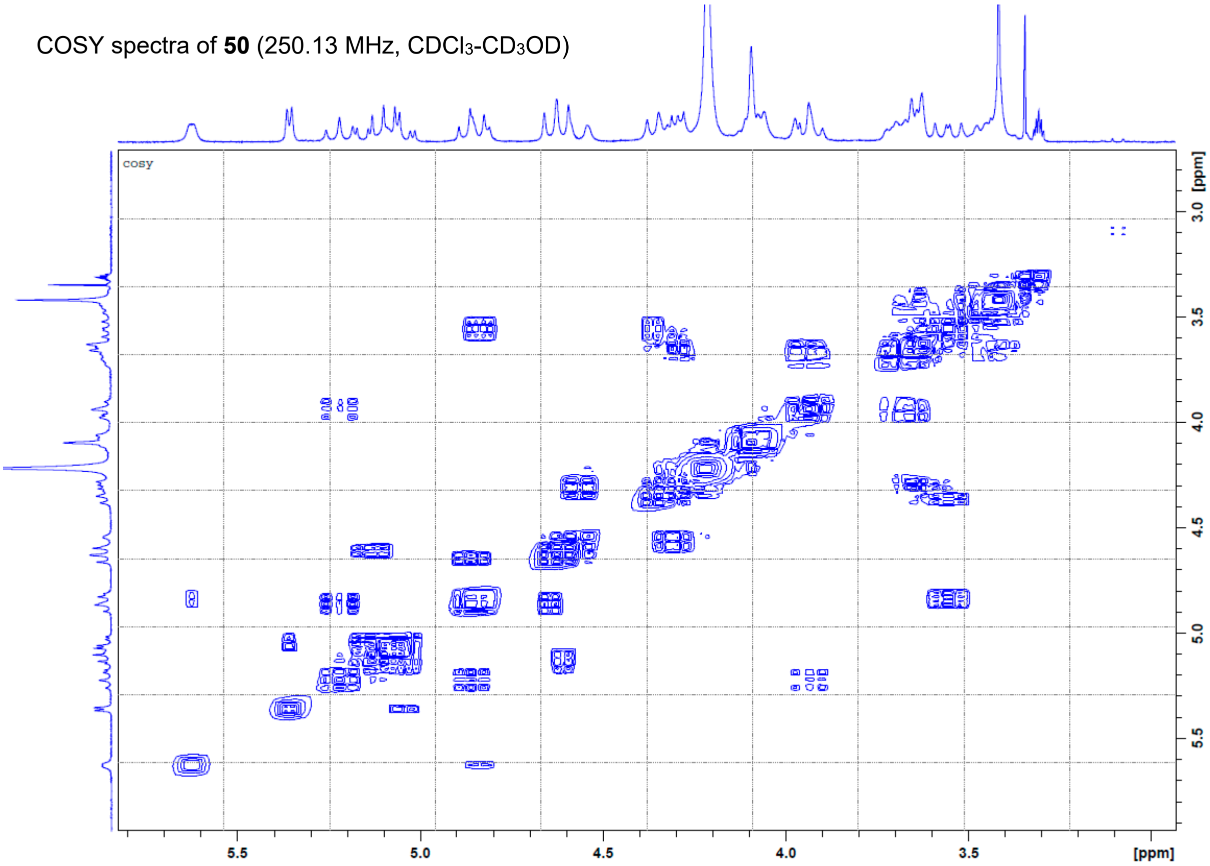

HETCOR spectra of **50** (62.9 MHz, $CDCl_3$-CD_3OD)

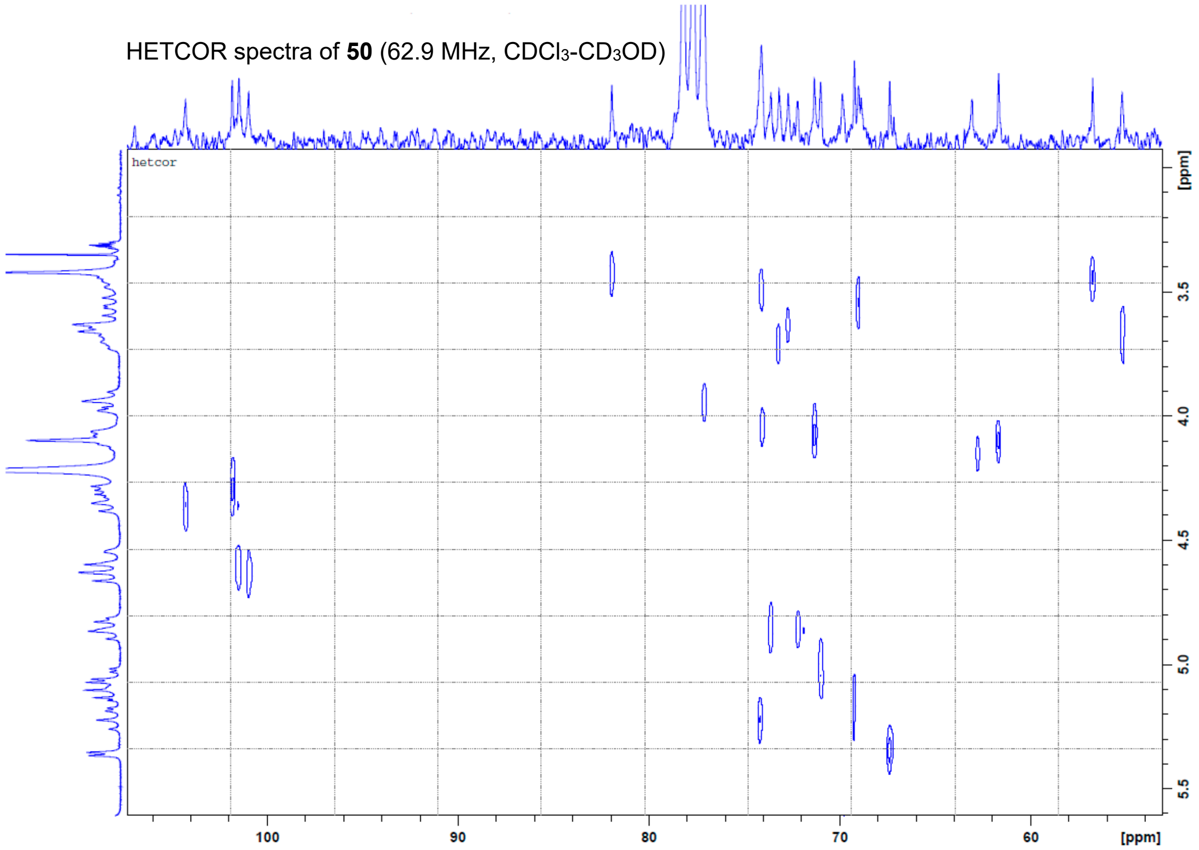

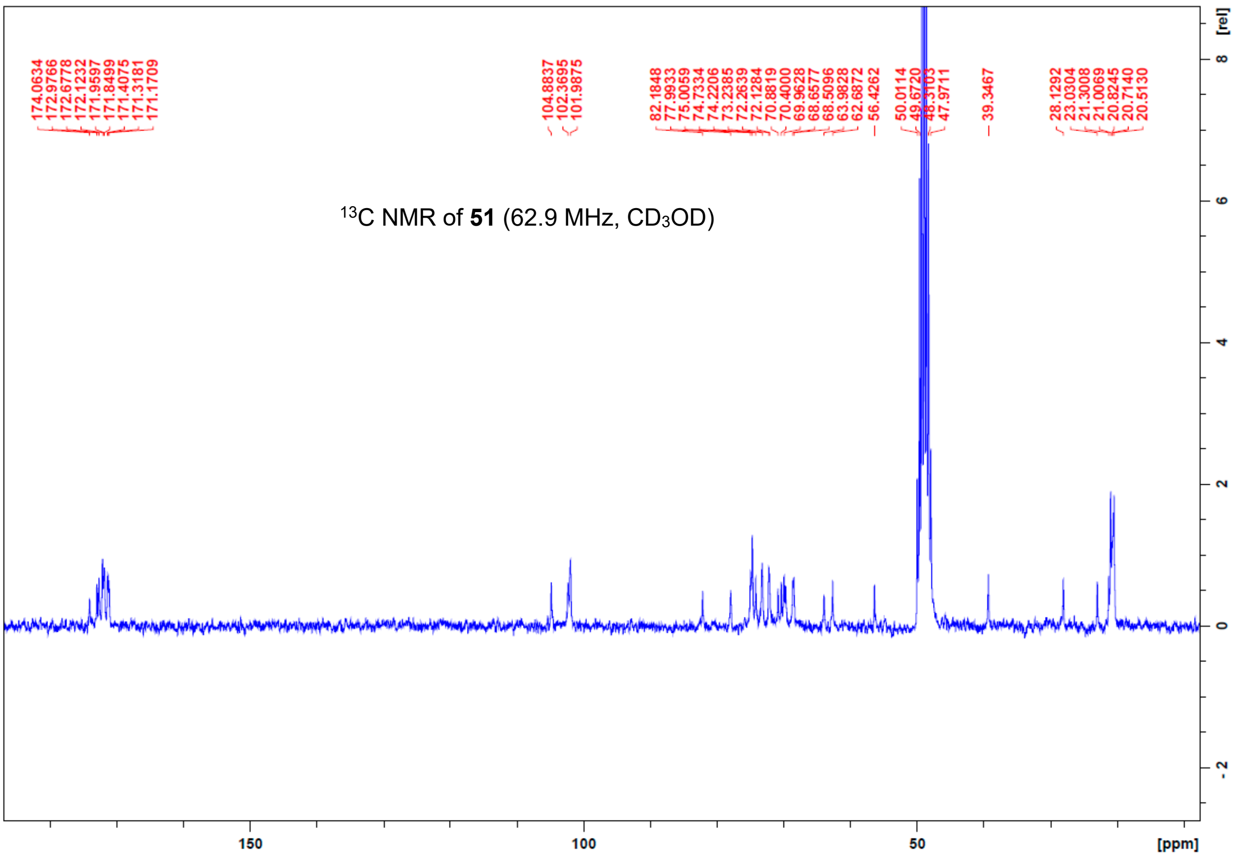
^{13}C NMR of **51** (62.9 MHz, CD_3OD)
174.0634
172.9766
172.6778
172.1232
171.9597
171.8499
171.4075
171.3181
171.1709
104.8837
102.3695
101.9875
82.1848
77.9933
75.0059
74.7334
74.2206
73.2385
72.2639
72.1284
70.8819
70.4000
69.9628
68.6577
68.5096
63.9828
62.6872
56.4262
50.0114
47.9711
39.3467
28.1292
23.0304
21.3008
21.0069
20.8245
20.7140
20.5130
[rel]
[ppm]
150
100
50

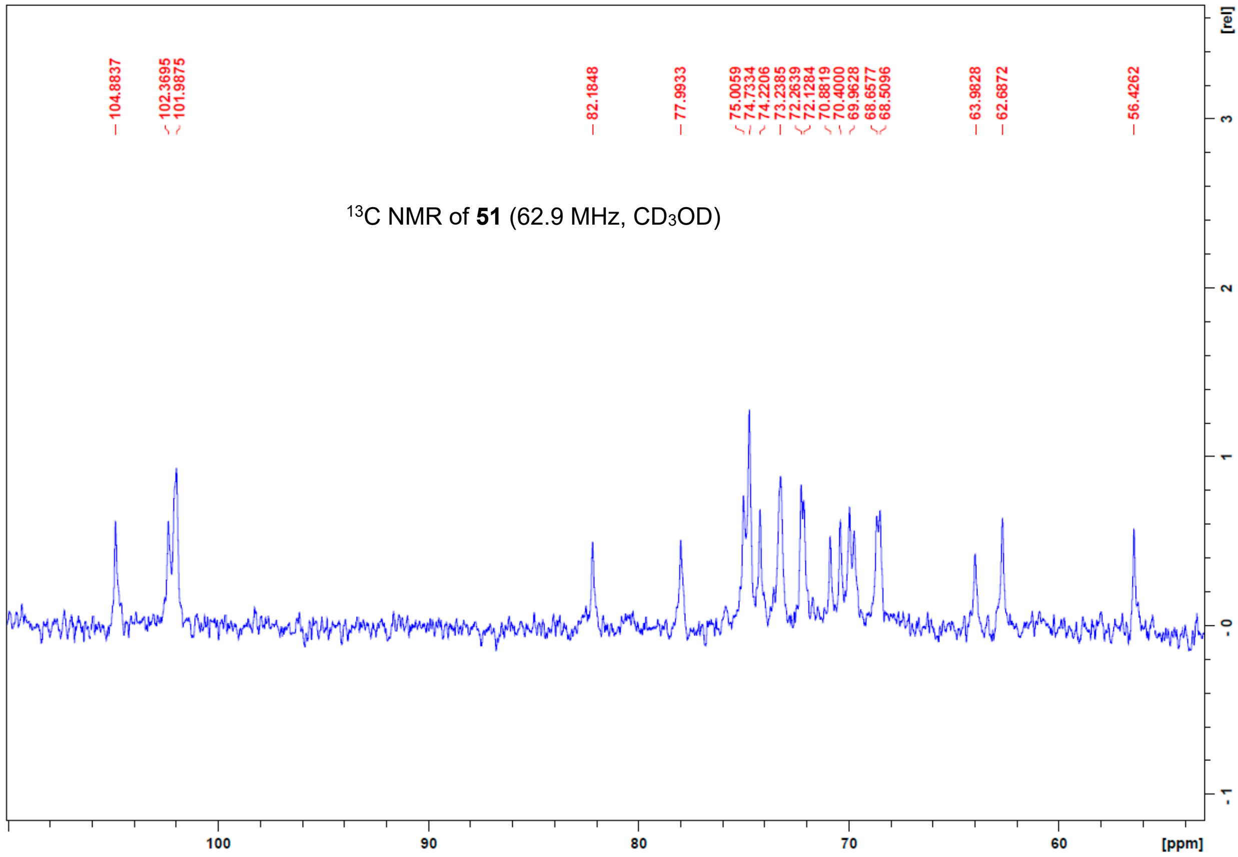
^{13}C NMR of **51** (62.9 MHz, CD_3OD)
104.8837
102.3695
101.9875
82.1848
77.9933
75.0059
74.7334
74.2206
73.2385
72.2639
72.1284
70.8819
70.4000
69.9628
68.6577
68.5096
63.9828
62.6872
56.4262
[rel]
[ppm]
100
90
80
70
60

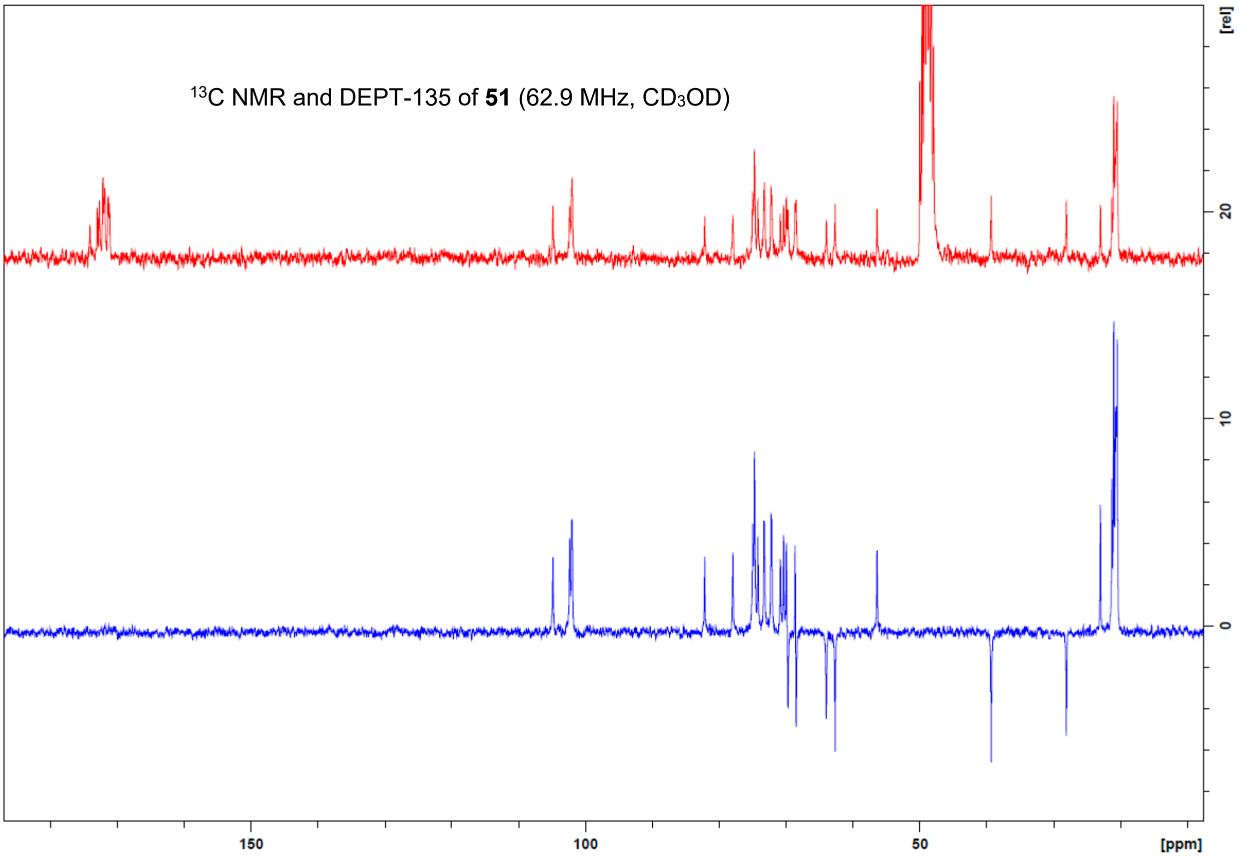

^{13}C NMR and DEPT-135 of **51** (62.9 MHz, CD_3OD)

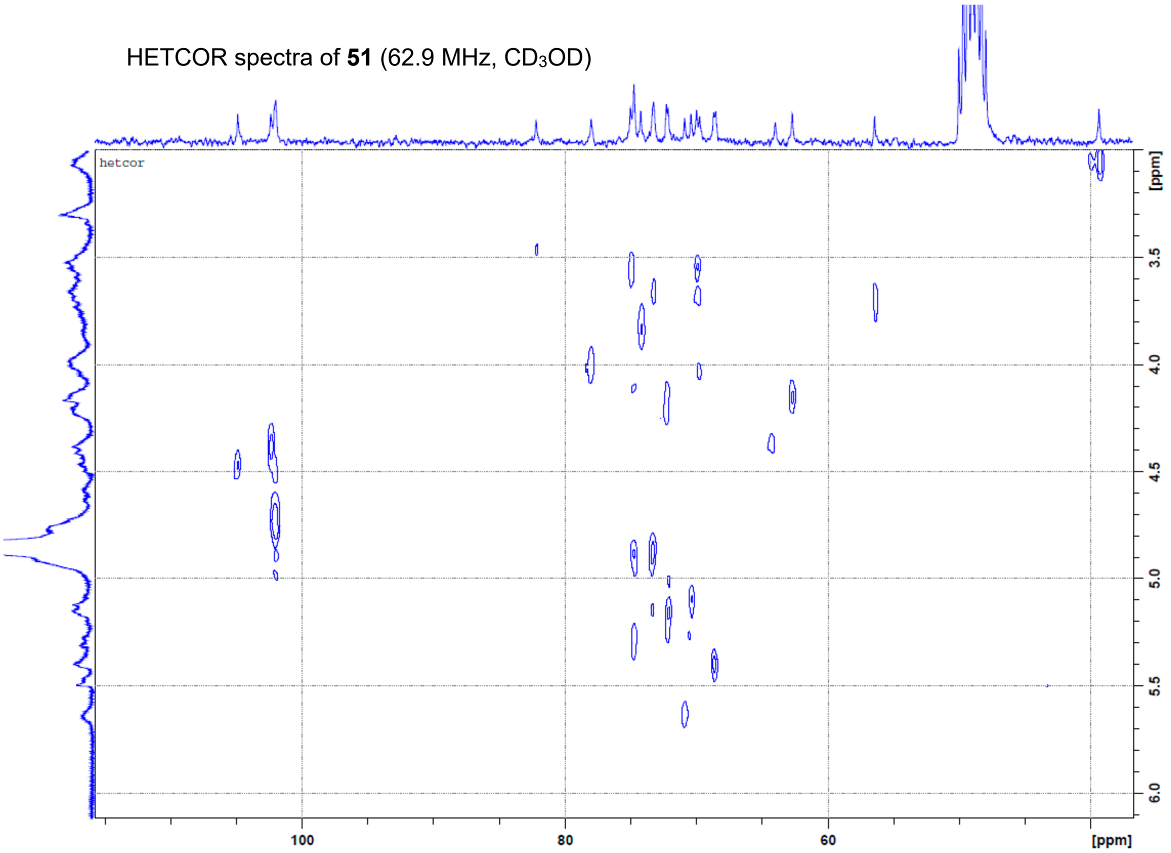

HETCOR spectra of **51** (62.9 MHz, CD_3OD)

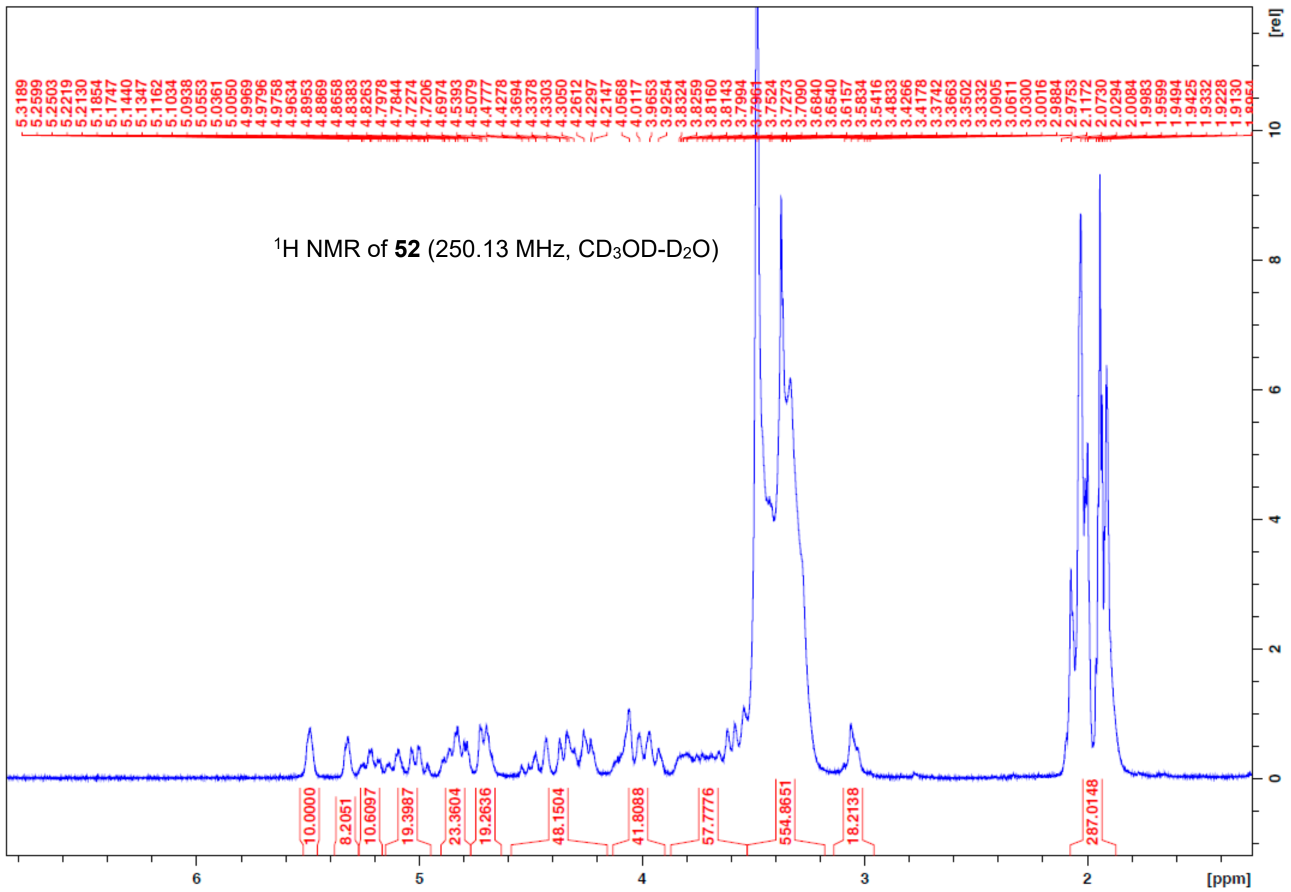

[rel]
1H NMR of **52** (250.13 MHz, CD_3OD-D_2O)
10.0000
8.2051
10.6097
19.3987
23.3604
19.2636
48.1504
41.8088
57.7776
554.8651
18.2138
287.0148
[ppm]

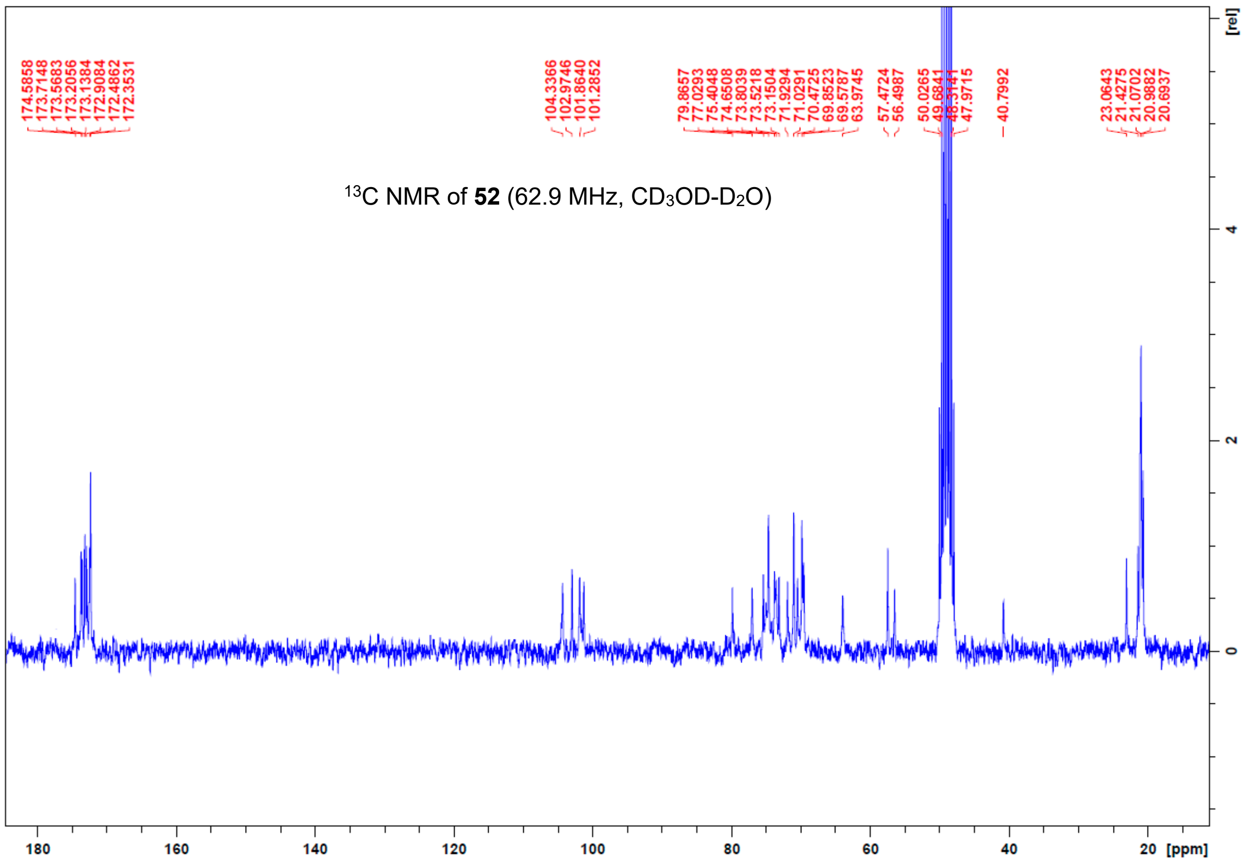

[rel]
13C NMR of **52** (62.9 MHz, CD_3OD-D_2O)
[ppm]

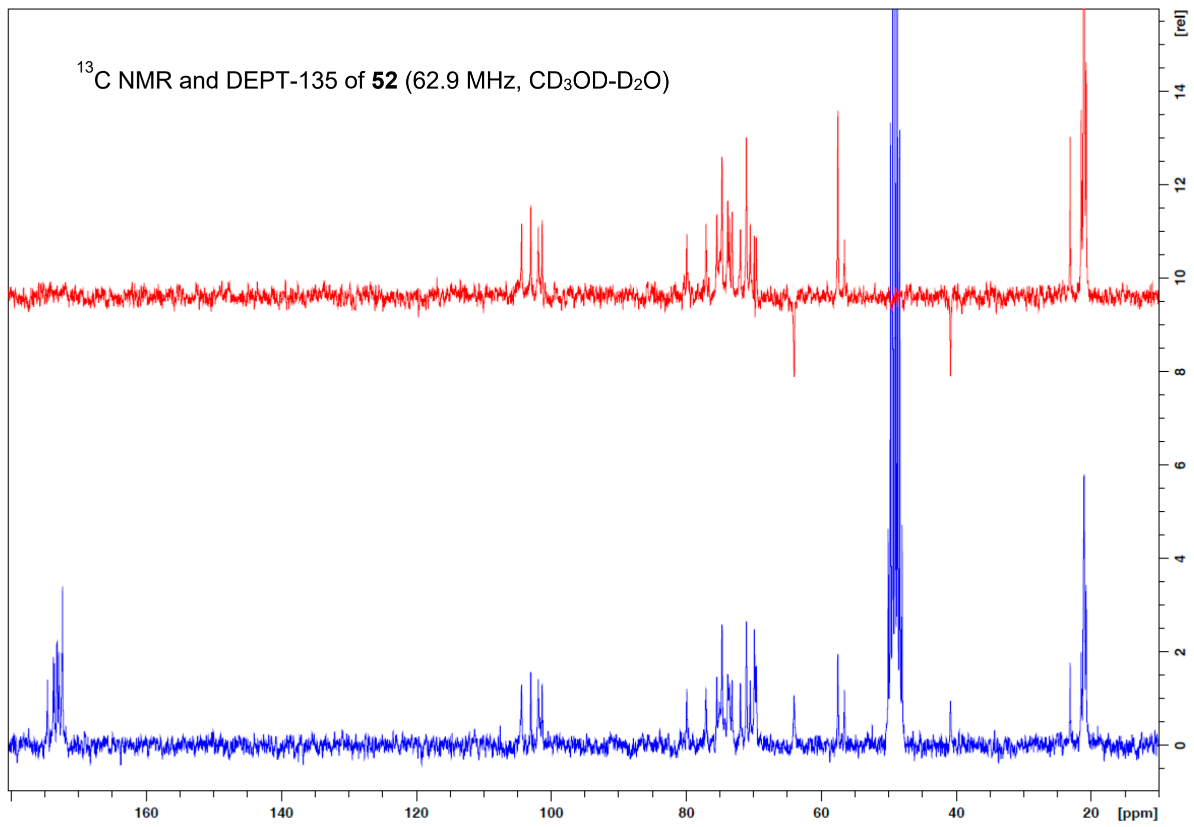

^{13}C NMR and DEPT-135 of **52** (62.9 MHz, CD_3OD-D_2O)
[rel]
14
12
10
8
6
4
2
0
160
140
120
100
80
60
40
20
[ppm]

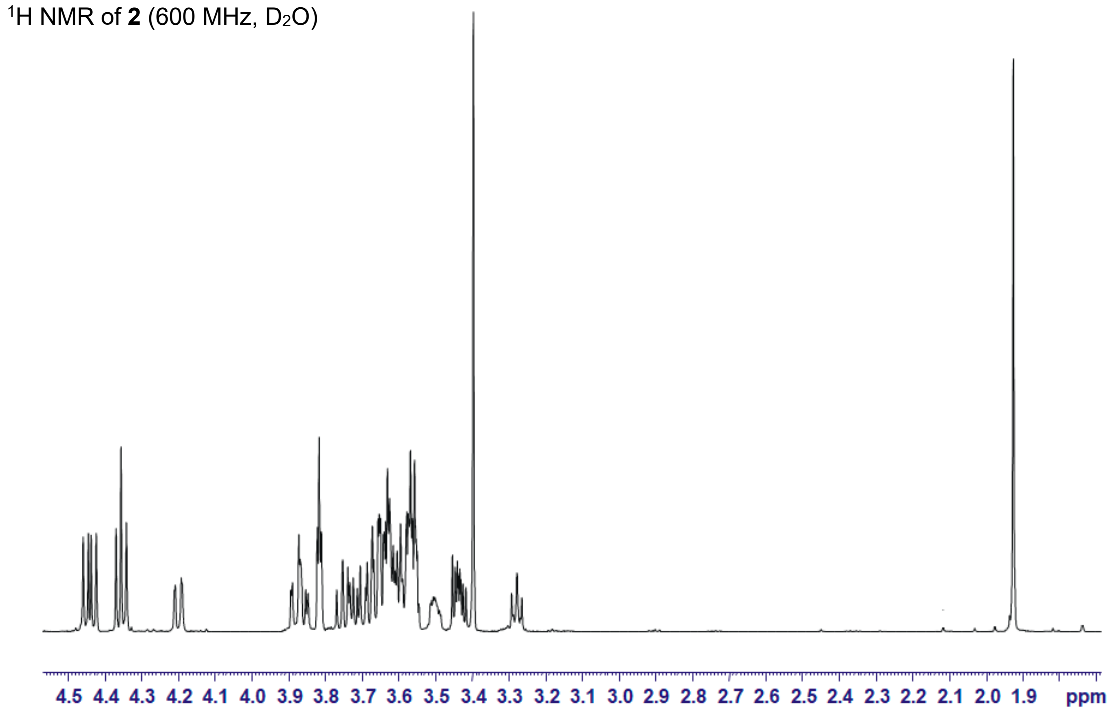

^{1}H NMR of **2** (600 MHz, D_2O)
4.5 4.4 4.3 4.2 4.1 4.0 3.9 3.8 3.7 3.6 3.5 3.4 3.3 3.2 3.1 3.0 2.9 2.8 2.7 2.6 2.5 2.4 2.3 2.2 2.1 2.0 1.9
ppm

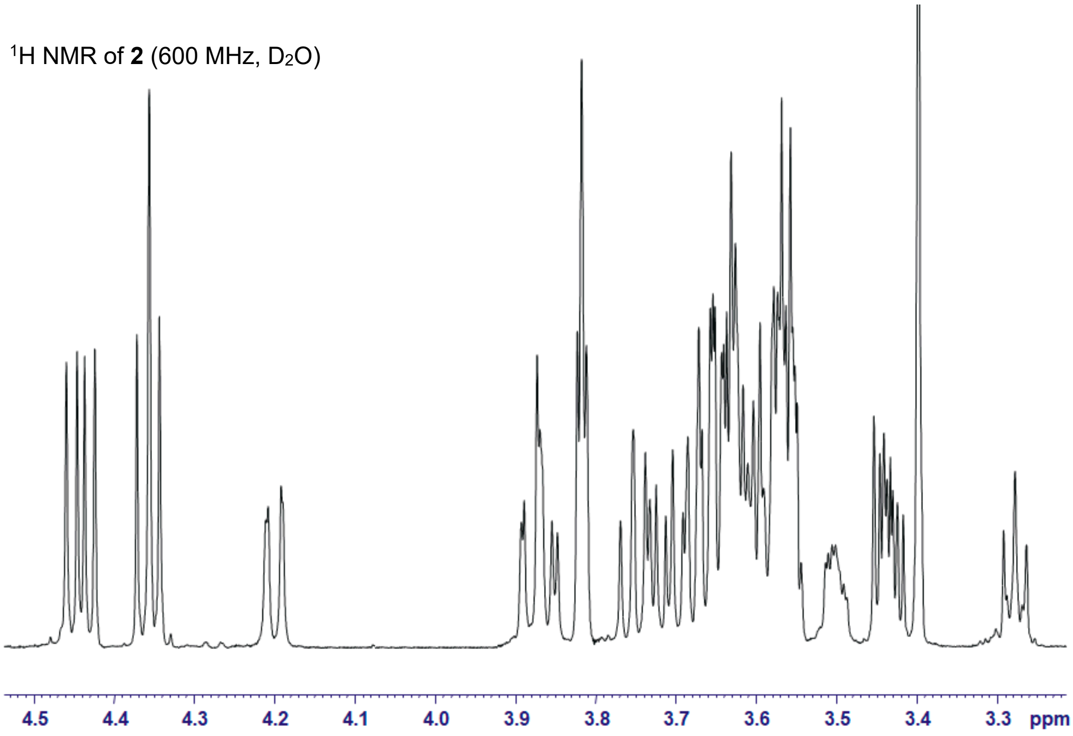

^{1}H NMR of **2** (600 MHz, D_2O)
4.5 4.4 4.3 4.2 4.1 4.0 3.9 3.8 3.7 3.6 3.5 3.4 3.3 ppm

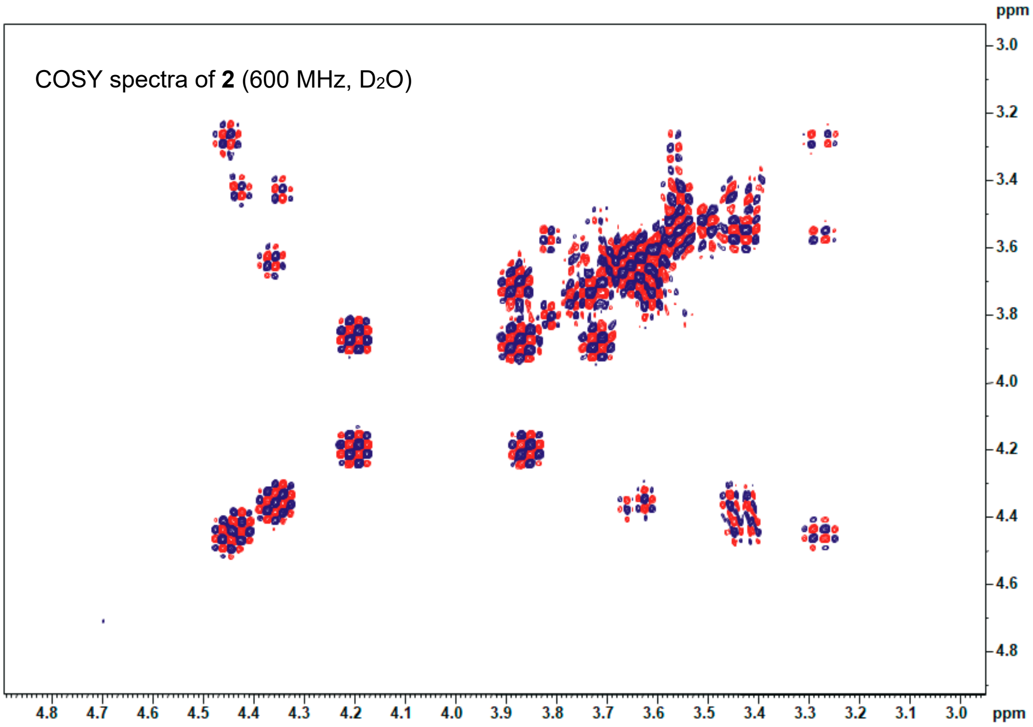

COSY spectra of **2** (600 MHz, D_2O)
ppm
3.0 3.2 3.4 3.6 3.8 4.0 4.2 4.4 4.6 4.8
4.8 4.7 4.6 4.5 4.4 4.3 4.2 4.1 4.0 3.9 3.8 3.7 3.6 3.5 3.4 3.3 3.2 3.1 3.0 ppm

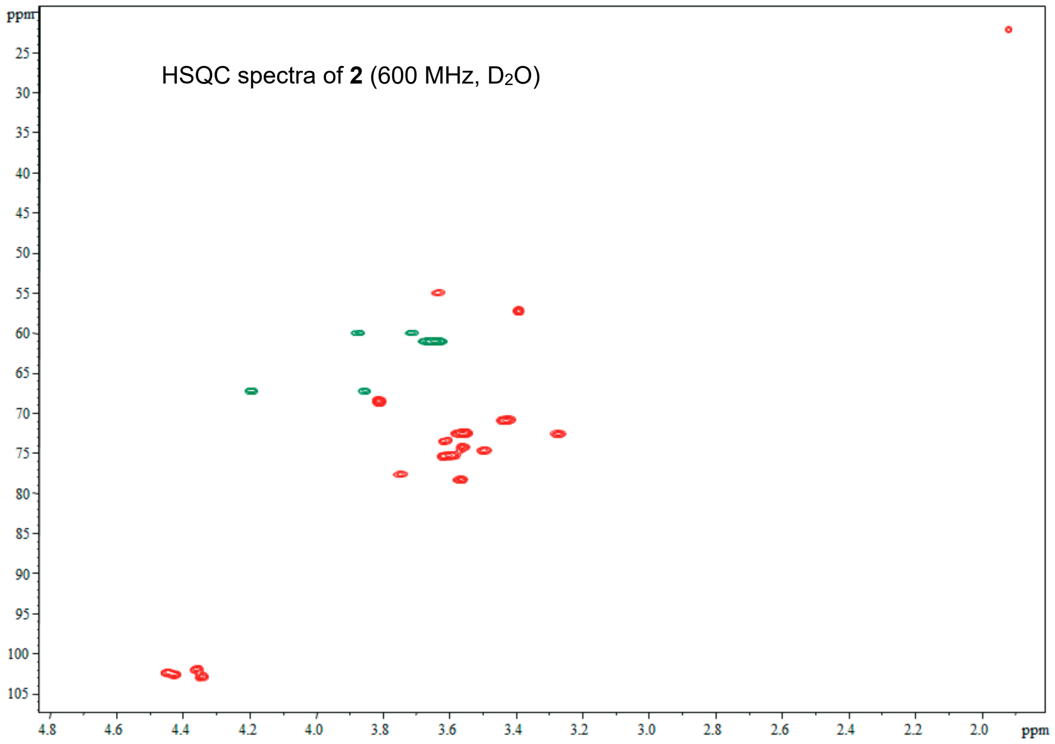

^{1}H NMR of **3** (600 MHz, D_2O)

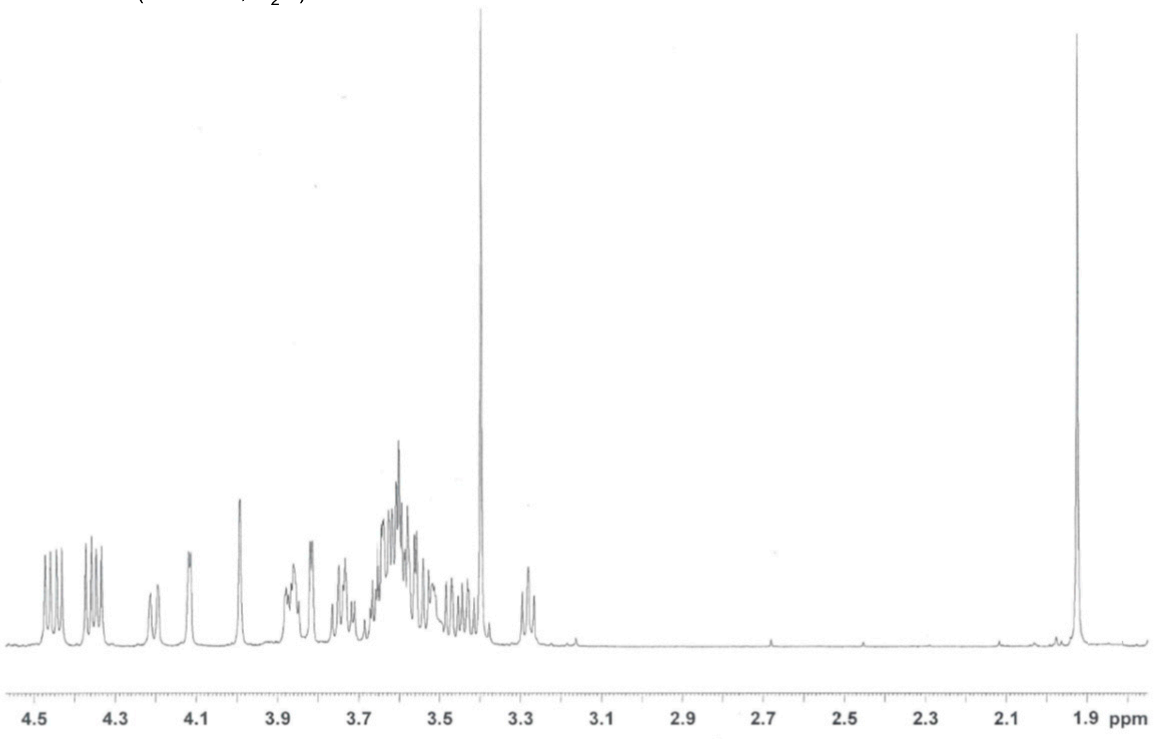

^{1}H NMR of **3** (600 MHz, D_2O)

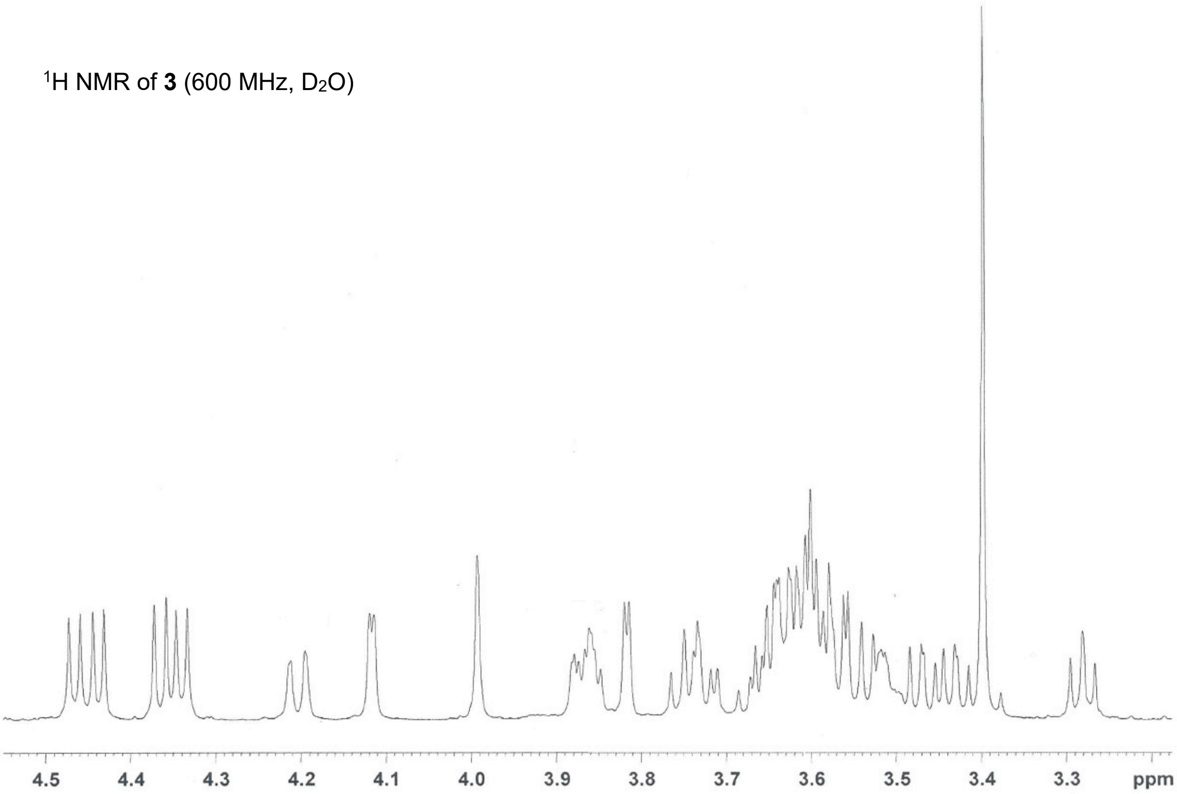

COSY spectra of **3** (600 MHz, D_2O)

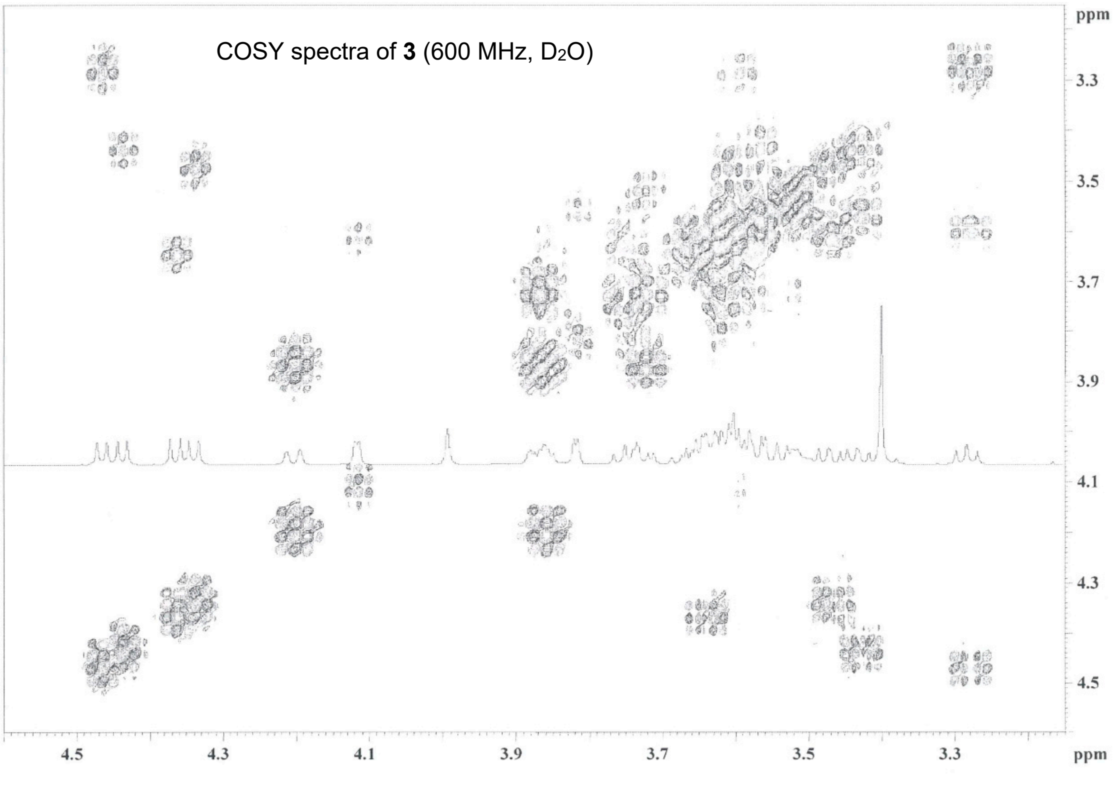

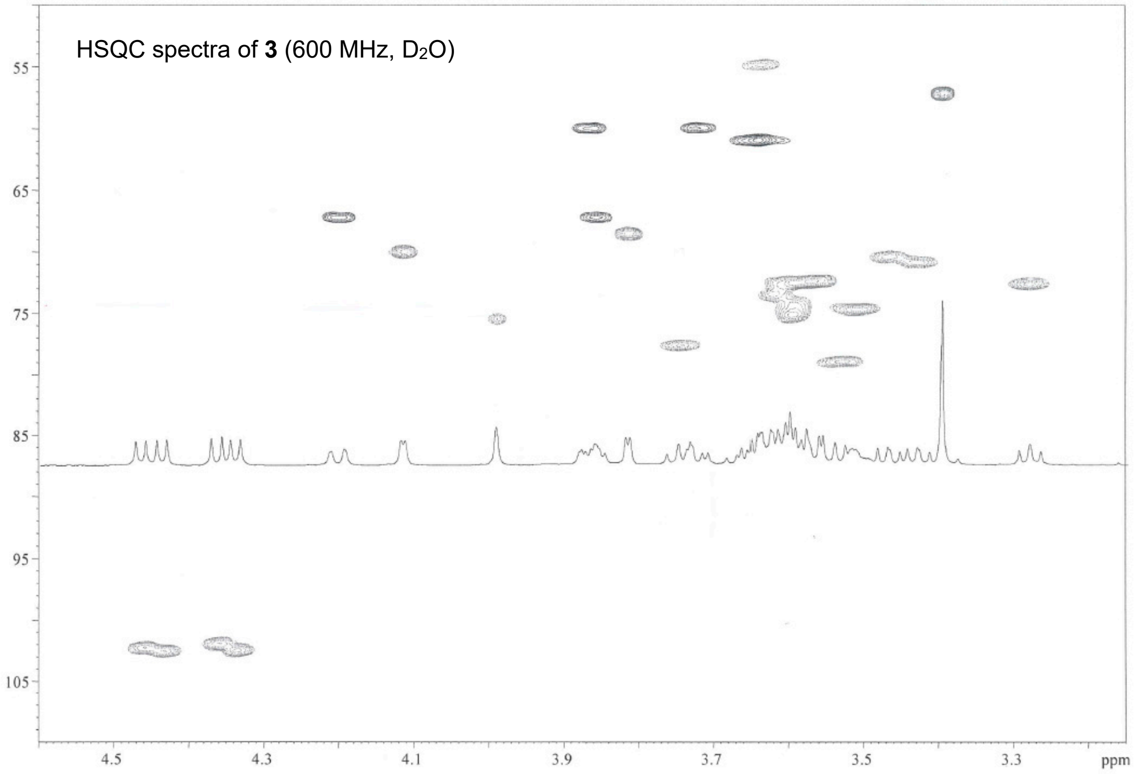
HSQC spectra of **3** (600 MHz, D_2O)
55
65
75
85
95
105
4.5
4.3
4.1
3.9
3.7
3.5
3.3
ppm

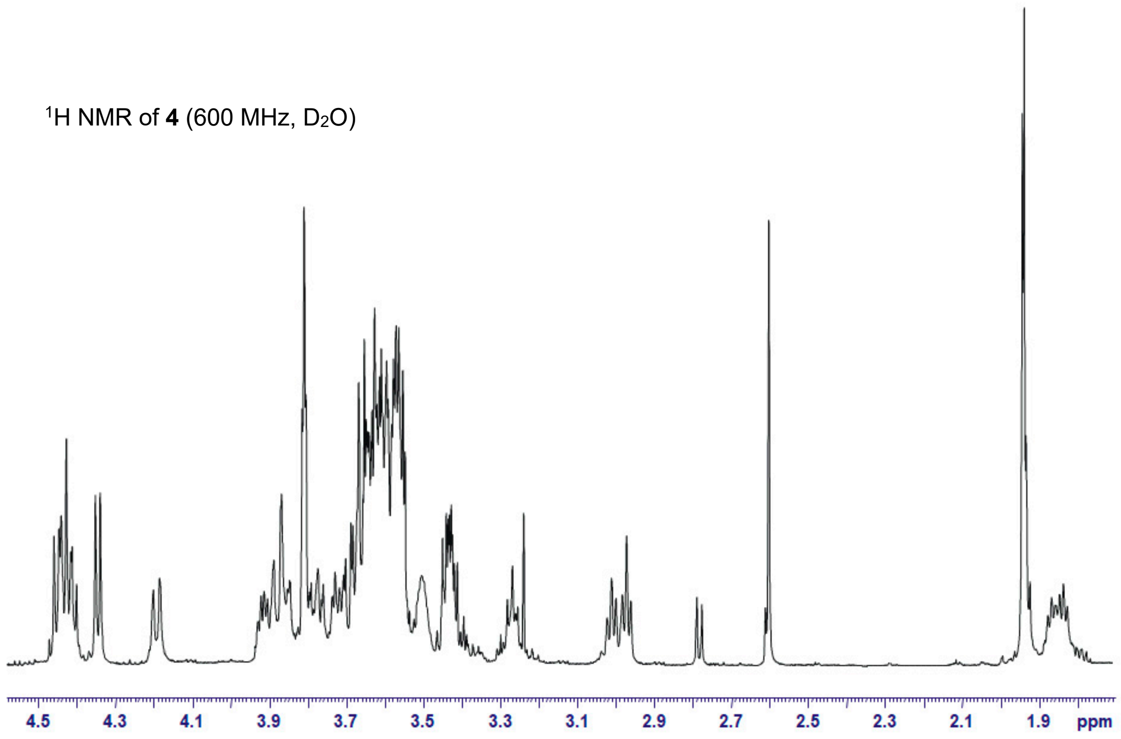
^{1}H NMR of **4** (600 MHz, D_2O)
4.5
4.3
4.1
3.9
3.7
3.5
3.3
3.1
2.9
2.7
2.5
2.3
2.1
1.9
ppm

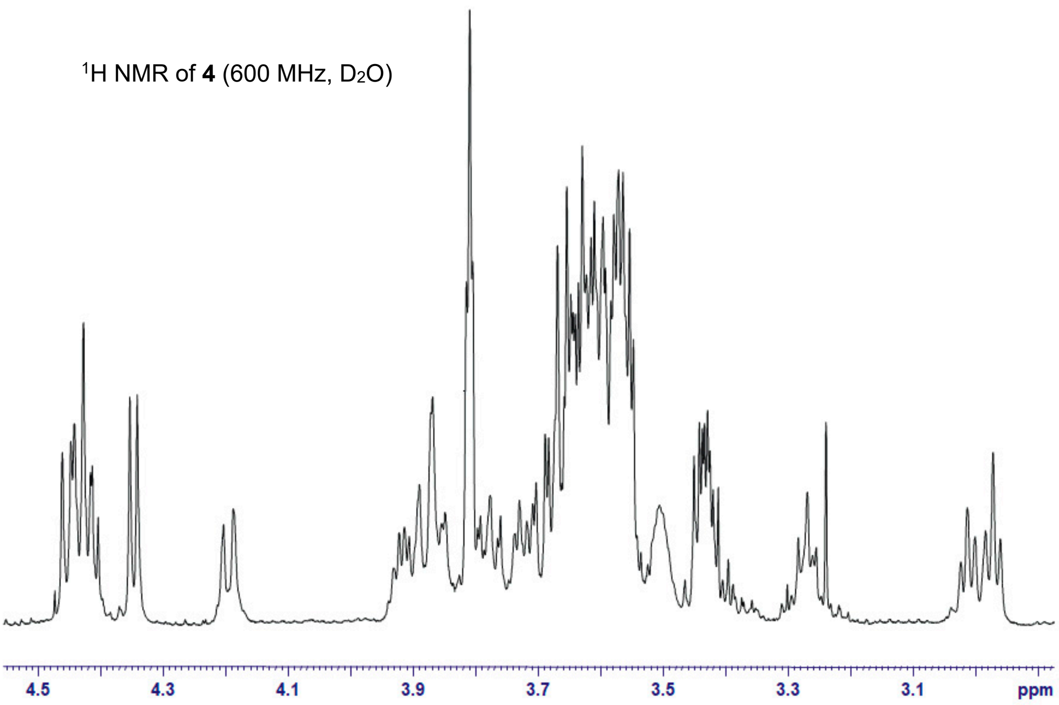
1H NMR of 4 (600 MHz, D2O)
4.5
4.3
4.1
3.9
3.7
3.5
3.3
3.1
ppm

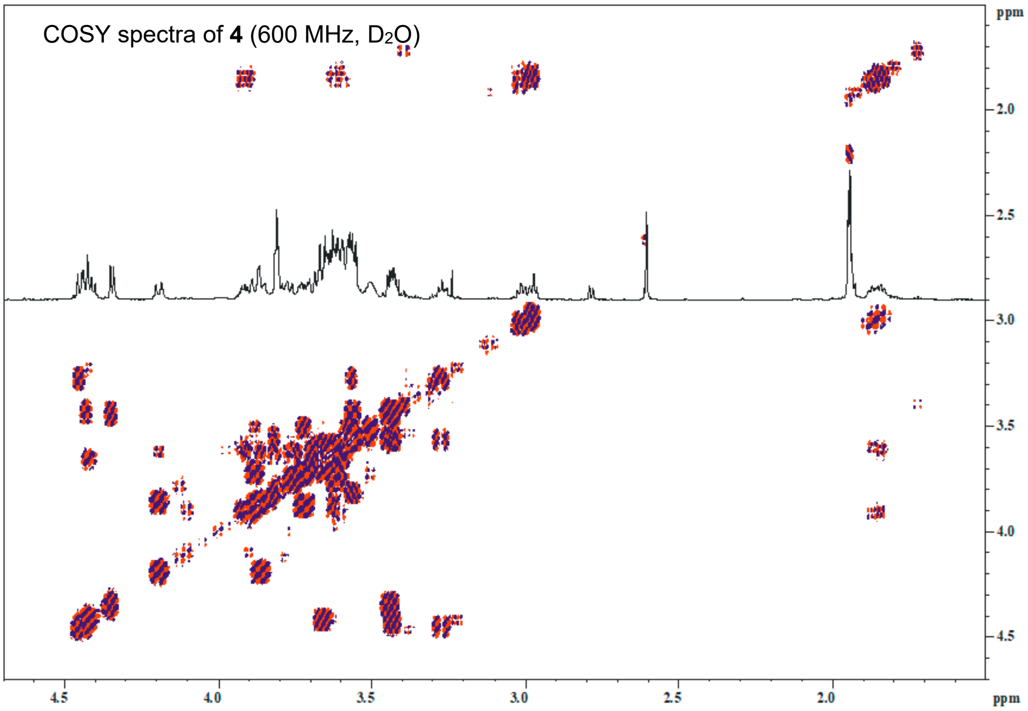
COSY spectra of 4 (600 MHz, D2O)
ppm
2.0
2.5
3.0
3.5
4.0
4.5
4.5
4.0
3.5
3.0
2.5
2.0
ppm

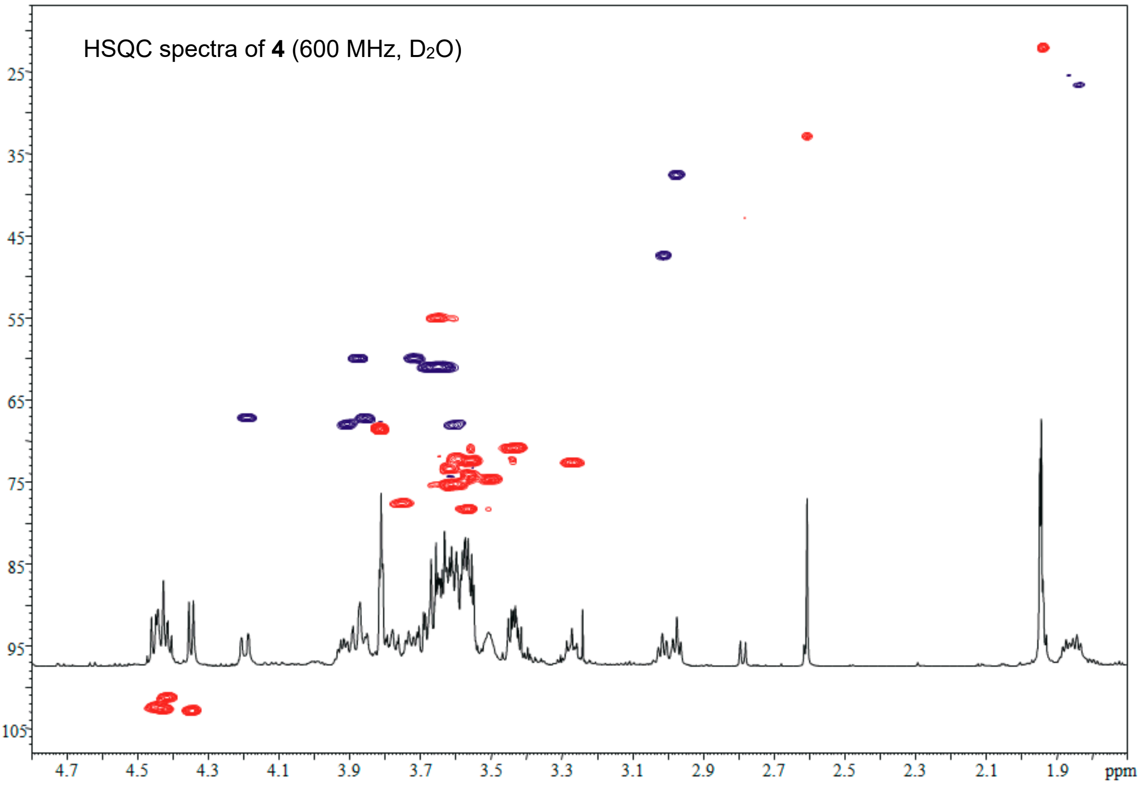

HSQC spectra of **4** (600 MHz, D_2O)
25
35
45
55
65
75
85
95
105
4.7 4.5 4.3 4.1 3.9 3.7 3.5 3.3 3.1 2.9 2.7 2.5 2.3 2.1 1.9 ppm

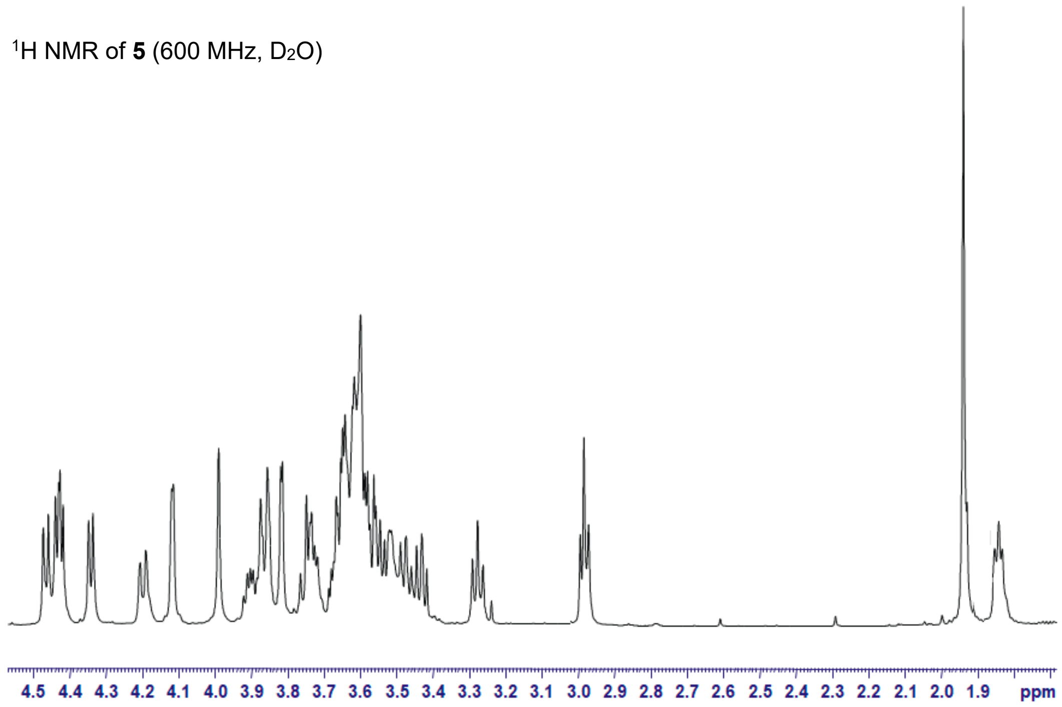

^{1}H NMR of **5** (600 MHz, D_2O)
4.5 4.4 4.3 4.2 4.1 4.0 3.9 3.8 3.7 3.6 3.5 3.4 3.3 3.2 3.1 3.0 2.9 2.8 2.7 2.6 2.5 2.4 2.3 2.2 2.1 2.0 1.9 ppm

^{1}H NMR of **5** (600 MHz, D_2O)

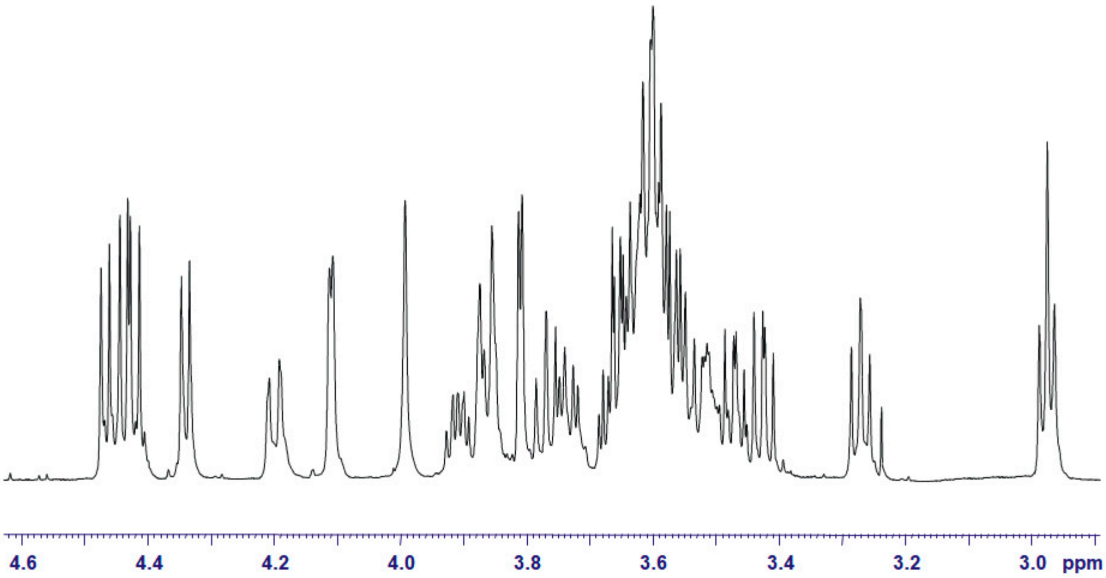

COSY spectra of **5** (600 MHz, D2O)

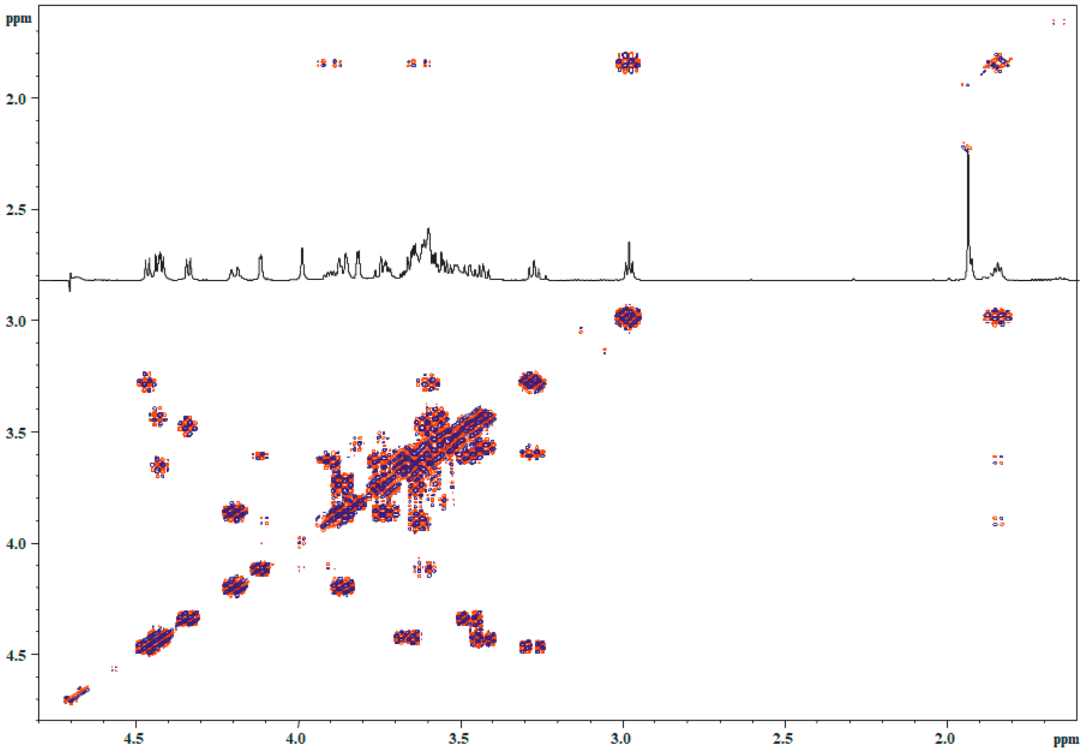

HSQC spectra of **5** (600 MHz, D_2O)

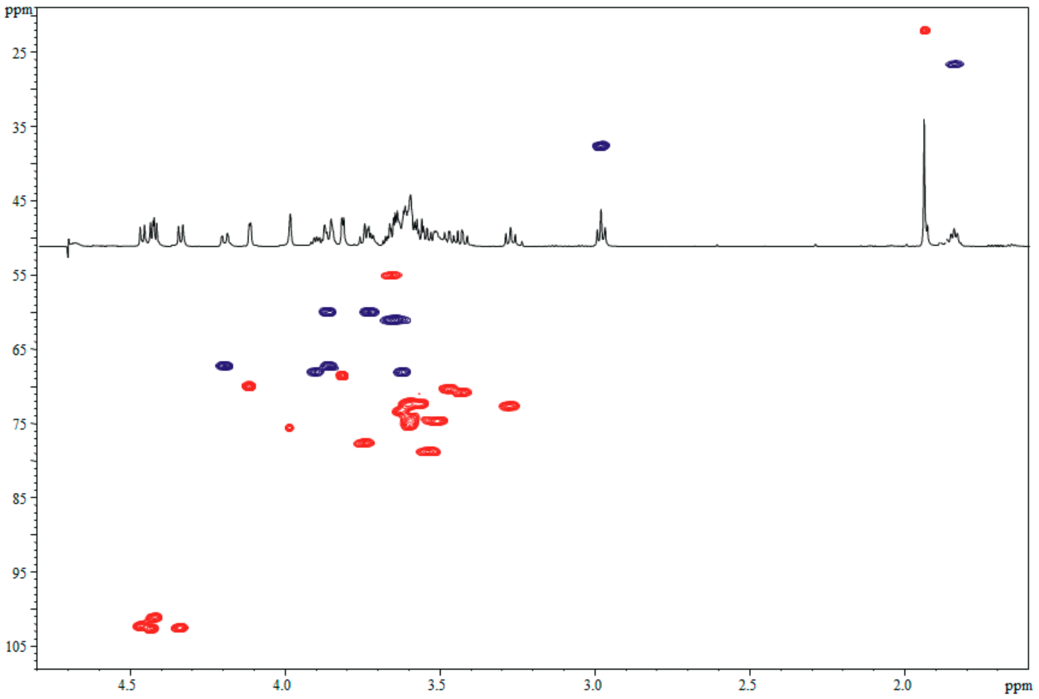

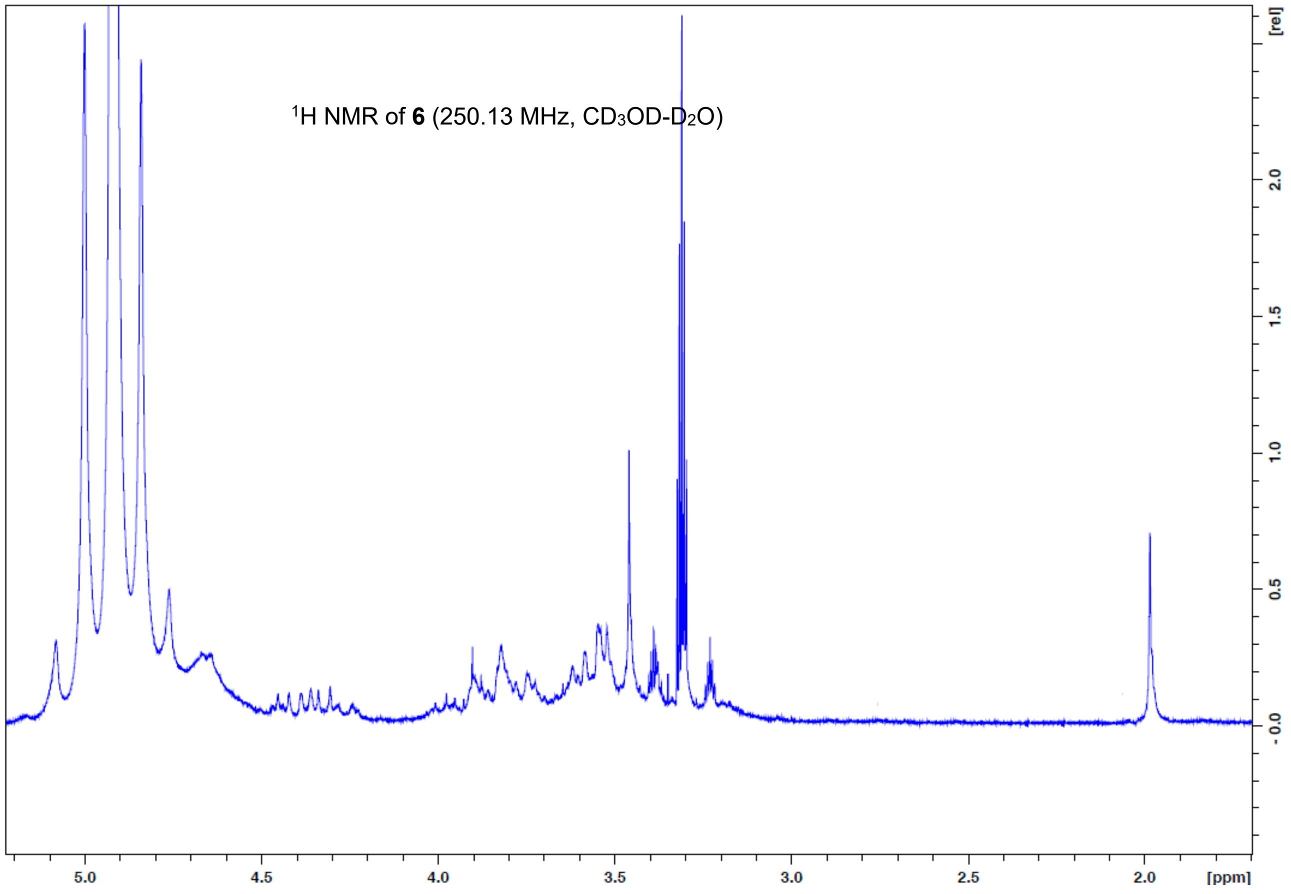

^{13}C NMR of **6** (62.9 MHz, CD_3OD-D_2O)

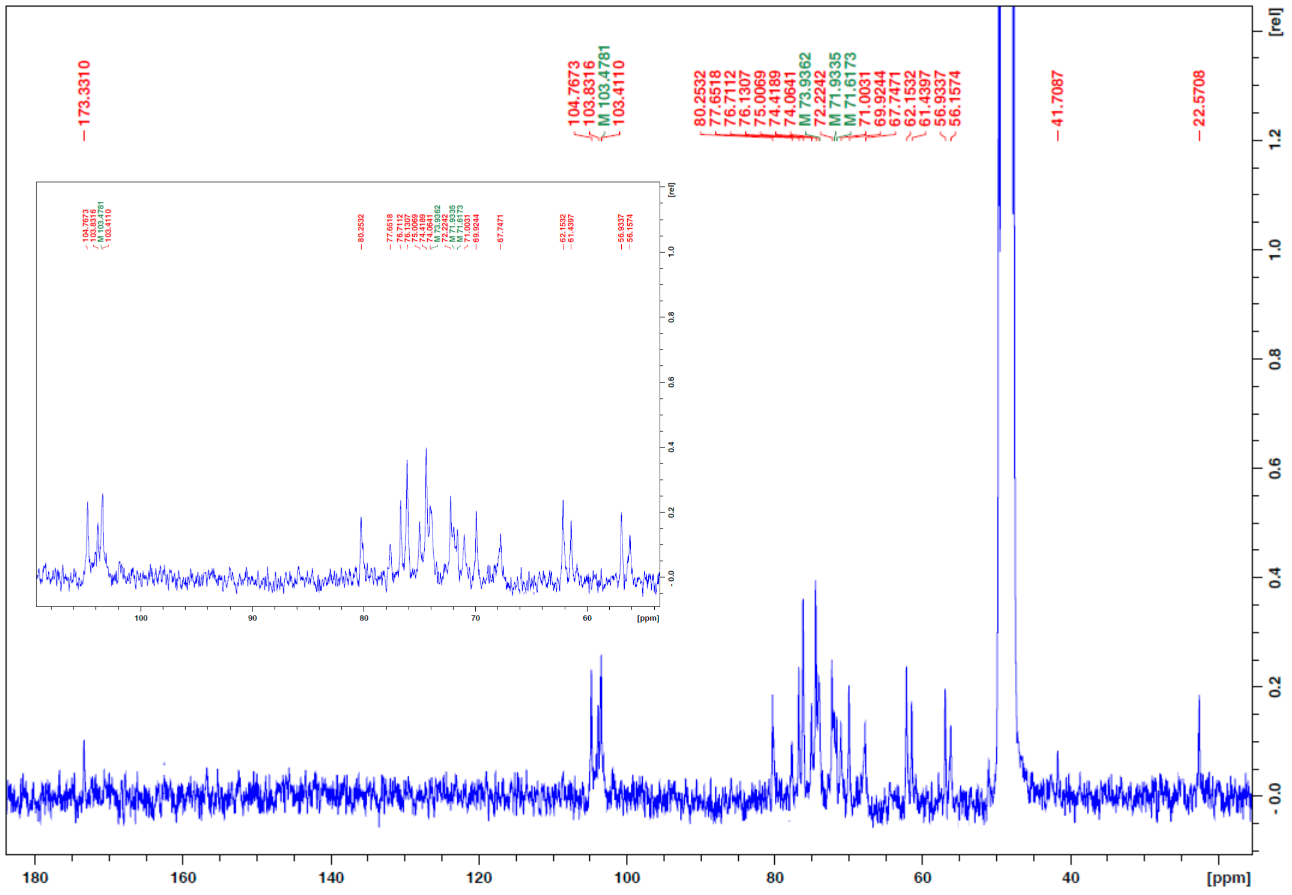

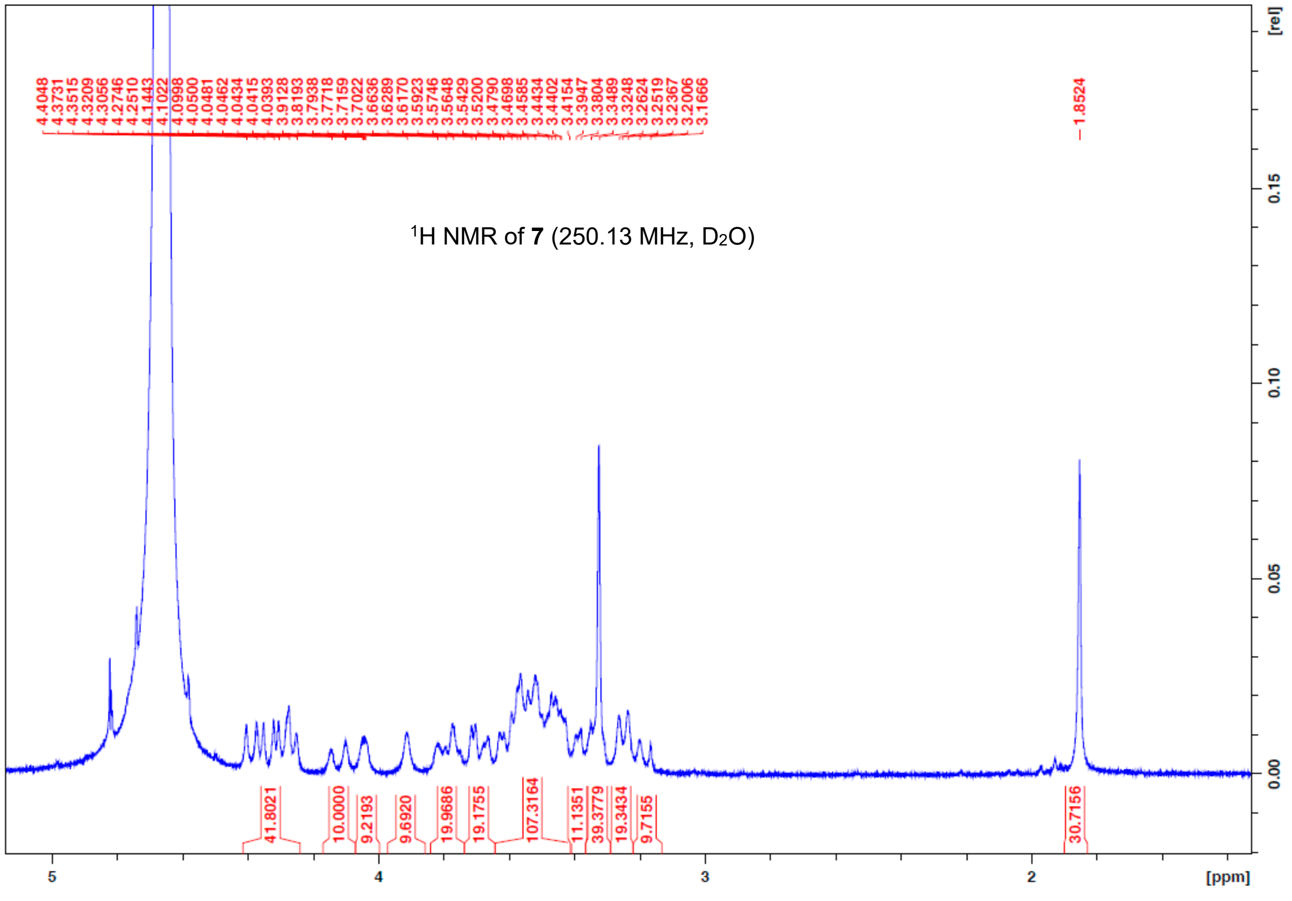

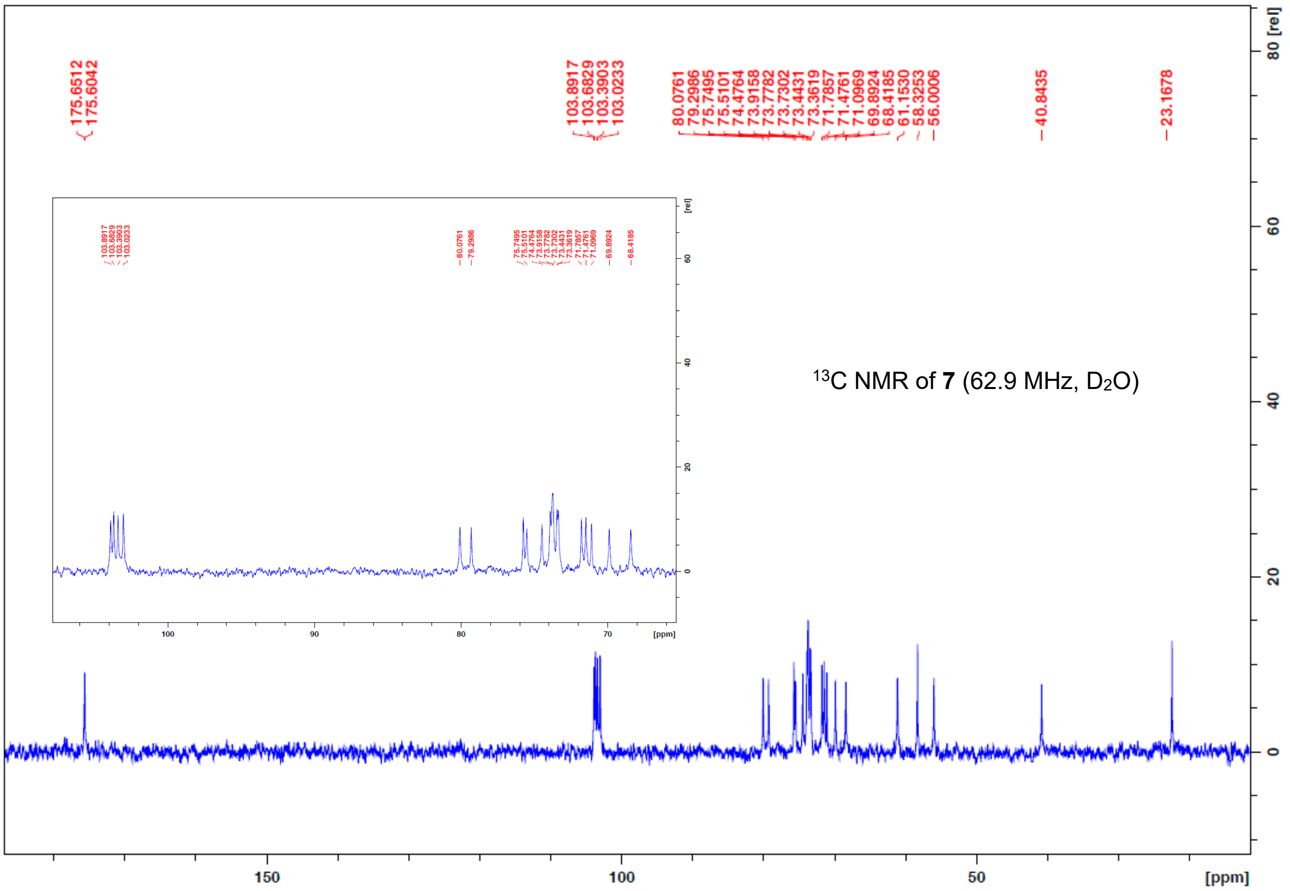
13C NMR of 7 (62.9 MHz, D2O)

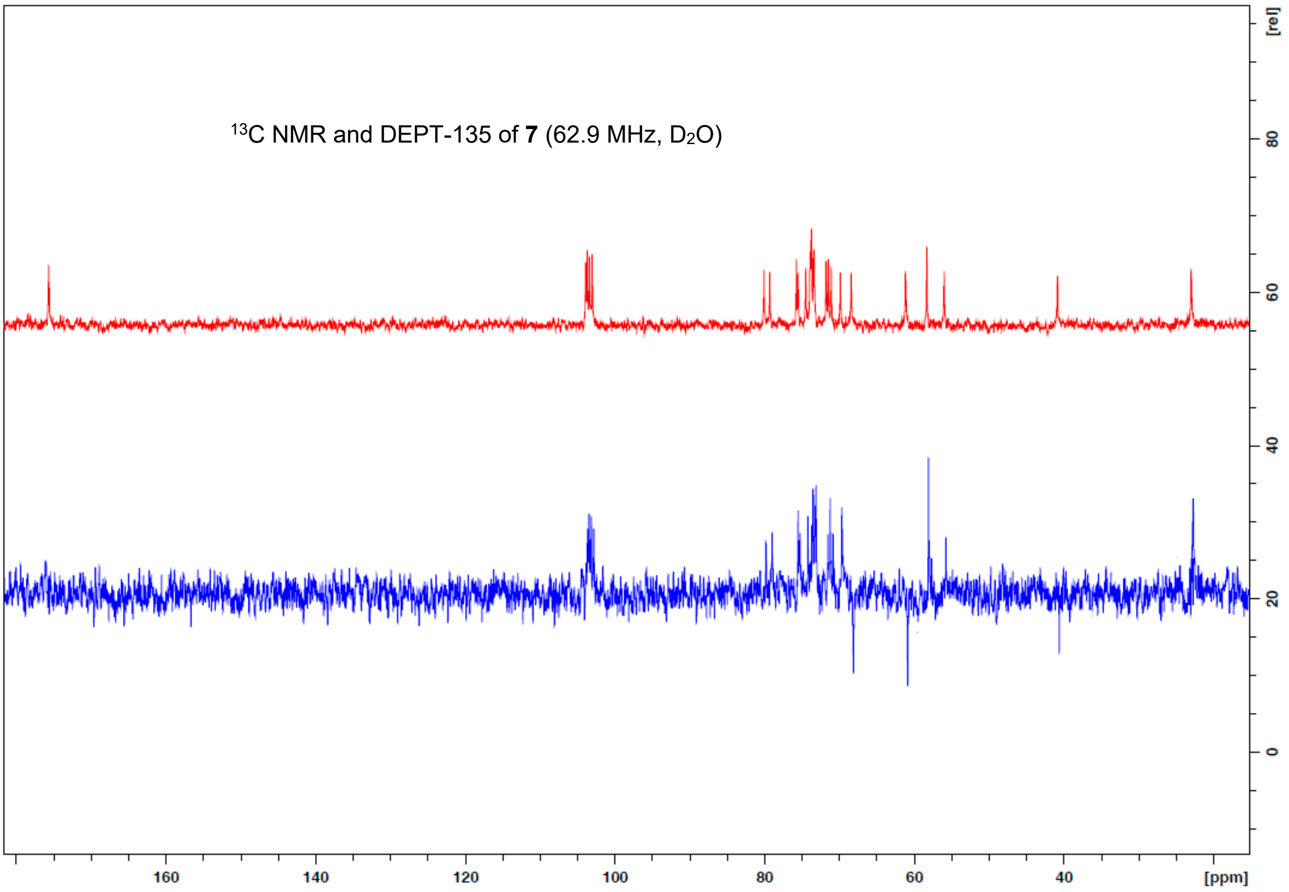
13C NMR and DEPT-135 of 7 (62.9 MHz, D2O)
